# Computational elucidation of nonverbal behavior and body language in music therapy

**DOI:** 10.1093/pnasnexus/pgae475

**Published:** 2024-10-22

**Authors:** Billie Sandak, Avi Gilboa, David Harel

**Affiliations:** Department of Computer Science and Applied Mathematics, Faculty of Mathematics and Computer Science, The Weizmann Institute of Science, Rehovot 7610001, Israel; Department of Music, The Faculty of Humanities, Bar-Ilan University, Ramat-Gan 5290002, Israel; Department of Computer Science and Applied Mathematics, Faculty of Mathematics and Computer Science, The Weizmann Institute of Science, Rehovot 7610001, Israel

**Keywords:** computational technology, computer modeling, AI, music therapy, behavior

## Abstract

Music therapy has shown efficacy in serious and chronic conditions, mental disorders, and disabilities. However, there is still much to explore regarding the mechanisms through which music interventions exert their effects. A typical session involves interactions between the therapist, the client, and the musical work itself, and to help address the challenges of capturing and comprehending its dynamics, we extend our general computational paradigm (CP) for analyzing the expressive and social behavioral processes in arts therapies. The extension includes bodily and nonverbal aspects of the behavior, offering additional insights into the client's emotional states and engagement. We have used this version of the CP, which employs AI pose estimation technology, image processing, and audio analysis, to capture therapy-related psychometrics and their intra- and inter-session analysis. The CP is applied in a real-world proof-of-concept study, and the results enable us to pinpoint meaningful events and emergent properties not captured by the human eye, complementing the therapist's interpretations. The resulting data may also be useful in other scientific and clinical areas.

**Significance Statement**

How can you capture and rigorously analyze occurrences in a therapy session, especially those that are not easily captured and perceived by the human eye? How can you quantify the client's musical and bodily behaviors, and, potentially, also progress or regression? How can you share these data in the scientific and clinical fields? Expanding our computational paradigm to account for the body language and nonverbal behavior of the client and modeling-related psychometrics, we report on the method's application in a real-world music therapy proof-of-concept investigation toward answering these challenges. We also depict sessions’ events and findings in concise, accurate, and conveyable notation that complements the therapist's verbal interpretations and provides empirical and clinical insights.

## Introduction

Music therapy is an arts-based approach used across diverse populations and age groups to address a range of medical situations and enhance well-being. It has been found to be beneficial in various serious and chronic conditions, illnesses, mental disorders, and disabilities. Research has shown its efficacy and potential for inducing therapeutic and psychosocial effects in these contexts (1–19). Moreover, engagement with music has been recognized as a means to improve the quality of life and one's overall well-being, not only for patients but also for healthy individuals. The positive effects of music extend beyond clinical settings, are utilized for research and practice in the social sciences, and offer opportunities to understand and empower individuals, groups, communities, and societies (20–29). Despite the extensive use and positive outcomes associated with music therapy, there is still much to explore and uncover regarding the underlying behavioral mechanisms through which music interventions exert their effects, and on a minute-by-minute micro level (30). Further research is clearly needed in order to gain a deeper understanding of how and why music therapy works in different contexts. This knowledge can help refine and enhance the effectiveness of music interventions (31–33).

The clinical setting in music therapy is a complex environment, where various elements, including the therapist, the client, and the musical work itself, interact dynamically. This interplay involves intricate and simultaneous expressive and social musical, verbal, and gestural behavioral processes that can be challenging to capture and comprehend. Often, these processes are perceived subjectively and interpreted by music therapists, primarily through verbal descriptions, which can influence subsequent analyses and understanding.

To address these challenges and enhance our understanding of arts therapies, a general computational paradigm (CP) has been developed and applied to the art and music modalities (34–37). This CP offers a way to overcome barriers in arts-based fields, by providing a rigorous and quantitative framework for tracking, analyzing, and documenting the actions during the sessions and the underlying dynamic processes. It also enables researchers to conduct exploratory investigations for individuals and collectives, as well as test hypotheses, generate new hypotheses, and discover knowledge grounded in empirical evidence.

Earlier versions of the CP focus on the creation work itself, i.e. the musical work. Here, we expand the CP to address the bodily and nonverbal aspects of the client's behavior. Body language capture and analysis can provide valuable insights into a client's emotional state, engagement, and responses (38–45) during the therapeutic process (46, 47), thus potentially enhancing the understanding of the processes involved in music therapy interventions, and eventually also the effectiveness thereof. For example, a relaxed posture and open gestures may indicate a sense of comfort and receptiveness, while tense muscles, limited movement, or a closed-up posture may suggest anxiety, suspicion, or resistance. All these involve observing and interpreting nonverbal cues such as body movements, gestures, and postures in relation to interventions during the sessions, highly elusive tasks for the human eye (and memory), and hence very difficult to analyze and document.

Here, we focus on the computerized identification, quantification, and notation of the client's bodily and nonverbal states and events of interest and in a real-life situation, that is, the automatic capturing of therapy-related psychometrics, their intra- and inter-session analysis, and the production of concise graphical representation. Related work on computerized analysis of body language in a real-world setting is relatively scarce and mainly focuses on other fields, such as psychotherapy, human–robot interaction, and psychiatry (48–50). Past attempts to represent music therapy sessions graphically were done through manual extraction, transcription, and notation from the session's recording (51–56), where in (54) bodily gestures include hands use and head and torso movements manually identified from a minute-long video.

**Fig. 1.**
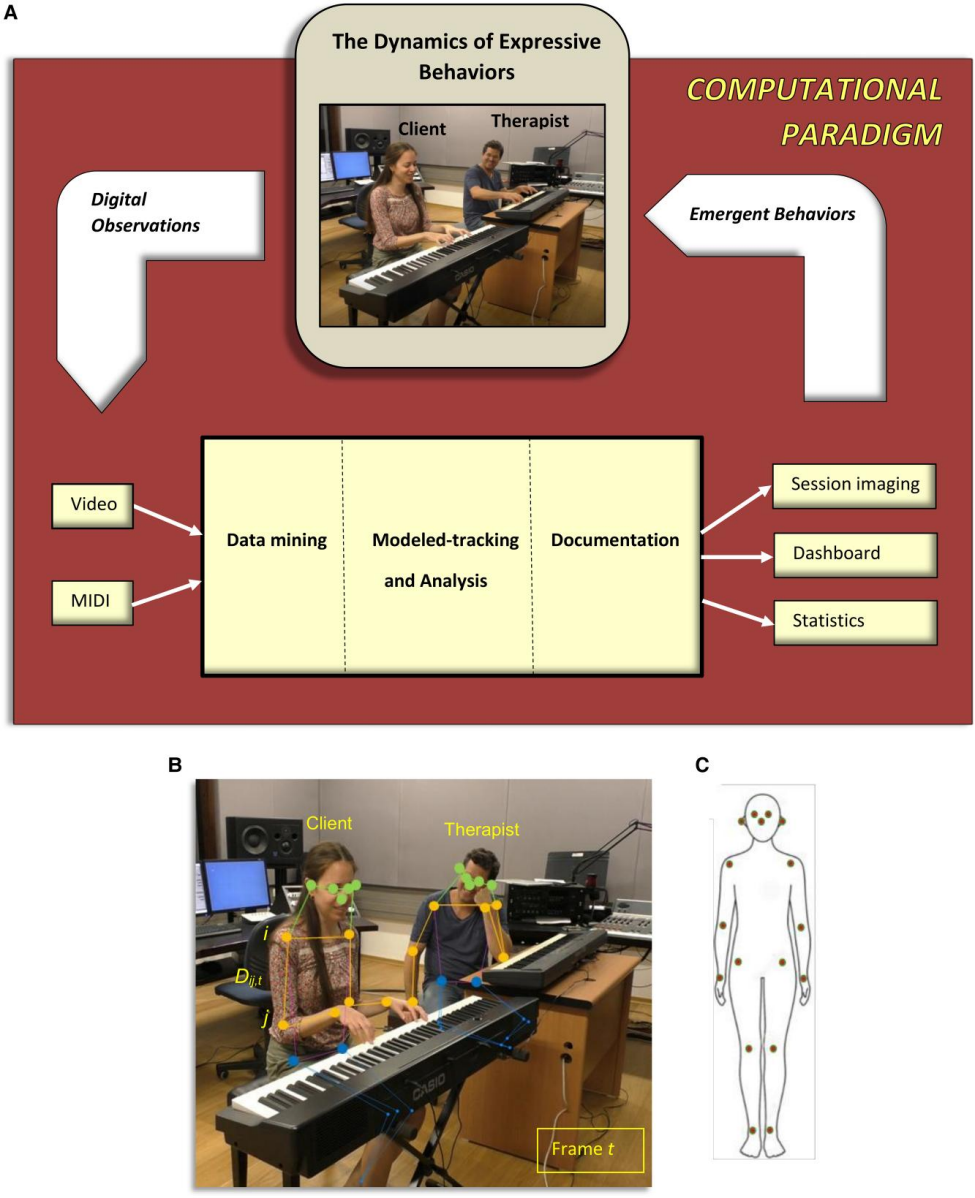
A) The method's components. Input: video and MIDI recordings. Output: graphical imaging of the session (e.g. Figs. 3 and 4), interactive dashboard (e.g. Fig. S1 and Video S1), and summary statistics of the client's behavioral metrics (e.g. Table 2). Process: First, the raw data are processed, selected, and translated. The client's nonverbal behavior is then tracked by the model and is rigorously analyzed and documented thereof. See further details in Fig. 2 and in the text. B) Automatic generation of a multijoint skeleton for each video frame, *t.* The multijoint skeleton is super-positioned on the client (subject) and the therapist. For every two joints, the distance between them, *D_ij_*, is computed. That is, for every two joints *i* and *j*; *x* and *y*, their video frame's pixel coordinates; *t*, a video frame at time *t*; the distance between joints is *D_ij,t_*  *_=_*  *| i_xy,t–_j_xy,t_ |.* C) Seventeen joints constitute the multijoint skeleton: “nose,” “left_eye,” “right_eye,” “left_ear,” “right_ear,” “left_shoulder,” “right_shoulder,” “left_elbow,” “right_elbow,” “left_wrist,” “right_wrist,” “left_hip,” “right_hip,” “left_knee,” “right_knee,” “left_ankle,” “right_ankle.”

With the new method at hand, we conducted a real-world proof-of-concept experiment, where subjects underwent one-on-one hour-long sessions with a music therapist, with the goal of enhancing their improvisational creativity and expressivity on a piano keyboard. See the experimental setup in Fig. 1A. Utilizing the method, we are able to pinpoint unusual and meaningful events and identify their exact time of occurrence during the session. It also enables us to identify emergent properties that signify higher-level behavior, which in general cannot be gleaned from mere examination of low-level events, that is, to discover patterns of behavior within a session or across sessions.

Behaviors of interest include torso positions—notably, shoulder and hand poses, as well as the use of the piano keyboard, which manifest levels of expressiveness and openness (43, 54). For example, when improvising with the hands in the same place on the keyboard the hands are “closed” in the same direction vector, while if the left hand is deep into the low keys and the right hand is on the highest keys, the hands are wide open forming a wide-angle “open” posture. Hands use is also of interest. Playing the piano with one hand or with two can help indicate as to the physical state of the client (e.g. cannot play with one of the hands) or the emotional state (e.g. does not want to with both hands or is intimidated by doing so) (53, 54). Putting focus on this behavior enables in depth understanding of the connection between the client and the instrument. Additional postures of interest are of the head tilt-up, straight, or down, which exposes the neck area to different degrees, signifying various levels of confidence or stiffness, and emotion recognition (38, 57). Body self-touch, that is, contact of one's hands on self's body for a light and brief touch or for an extended duration, e.g. facial touching or arm patting, can indicate levels of discomfort or stress and self-comfort moves (58, 59). Uneasiness may also be manifested by standing and walking away from piano keyboard (47, 60).

The kind of rigorous depiction of the session's occurrences that we make possible could lead to defining a potential domain-specific language for similar dynamic analysis in various other areas.

## Materials and methods

### The computational method

Previous versions of the CP focus on the musical work (34–37), and here, we report on its expansion to handle the body language of the client (see Fig. 1A). We model the system for tracking the psychometrics of interest, which are defined in Table 1. These include body, head and torso poses, self-touch, hands use, and silence and playing epochs (on a piano) all of which have expressive and clinical meaning and importance as summarized in Table 1.

**Table 1.** Behavioral metrics—definition and meaning.

Attribute	Specification (per session)	Clinical importance and meaning
Playing segments	The percentage of time the subject is playing the keyboard.	Indication of how much the client is engaged in music making and in production of sound, as opposed to engaging in verbal communication or in total silence.
Silence segments	The percentage of accumulated silence epochs of length 100 ms or more, also owing to slow playing.	Indication of the tendency to leave open spaces without playing or talking, and when playing, indicates a less dense and slow playing preference.
Body pose Standing Sitting (default)	The percentage of time when the subject is sitting at the keyboard or rising, standing, or walking away from it.	Indication of crucial incidences (43, 60). Standing or walking away from the keyboard can indicate discomfort, or, difficulty with the ideas suggested by the music therapist and/or with playing.
Self-Touch Head and upper torso (while playing) Middle torso (while playing) No self-touch	The percentage of session time when the subject touches his/her specified body parts, while playing or not. 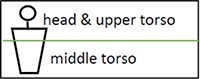	Self-touch is often habitual and automatic and can thus indicate unaware and unconscious material (e.g. stress and uneasiness) (58, 59). Enables to uncover the meaning with the client and act there upon.
Playing hand Both Left Right Not playing	The percentage of time when the subject is playing with the right hand, left hand, or both hands, or not playing at all.	Indication of the physical (e.g. limitation) or emotional connection between the client and the playing instrument (53, 54). Enables to expose playing habits and dispositions, and to see if counteracting these can promote the client.
Head pose Up Down Neutral	The percentage of time the subject's nose tip is above the horizon line (up) or below it (down). All angles in between are defined as neutral. 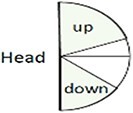	Indication of neck area exposure which may portray levels of confidence (38, 57). When playing, indicates how closely the client watches his/her hands (down pose), or playing freely without restrictions (head up).
Torso pose Closed Open Neutral	The percentage of time the subject is playing with both hands in closed or open forms. If the subject is playing with one hand or not playing at all, the pose is defined as neutral. 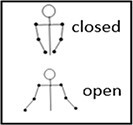	Indication of physical and creative restrictions (closed pose) or physical and creative freedom (open pose) (43, 54).

#### Overview

The inputs to the system are video and musical instrument data interface (MIDI) recordings of the music-based interventions sessions and the musical data therein. Using AI pose estimation algorithms that are deep neural network (DNN) based, we are able to generate a multijoint skeleton of the client and therapist entities from the video recordings (see Fig. 1B and C). Neural network is a broad term referring to a computational model inspired by the human brain's neural structure. It typically consists of layers of interconnected nodes (neurons), including an input layer, one or more hidden layers, and an output layer. A DNN specifically implies a neural network with a more significant number of hidden layers, making it “deep.” It enables the network to capture increasingly complex patterns and enhancing accuracy by modeling intricate relationships, and in tasks like pose estimation, to deduce spatial relationships and positions with advanced pattern recognition.

Here, we employ the bottom-up method, where each body joint is evaluated first and then they are arranged to compose a unique pose, and use the High-Resolution Net (HRNet) neural network (61), which is characterized by maintaining high-resolution representations when estimating postures, trained over the Common Objects in Context dataset (62). HRNet's architecture consists of parallel high-to-low-resolution subnetworks with repeated information exchange across multiresolution subnetworks (see (61) for further details).

**Fig. 2.**
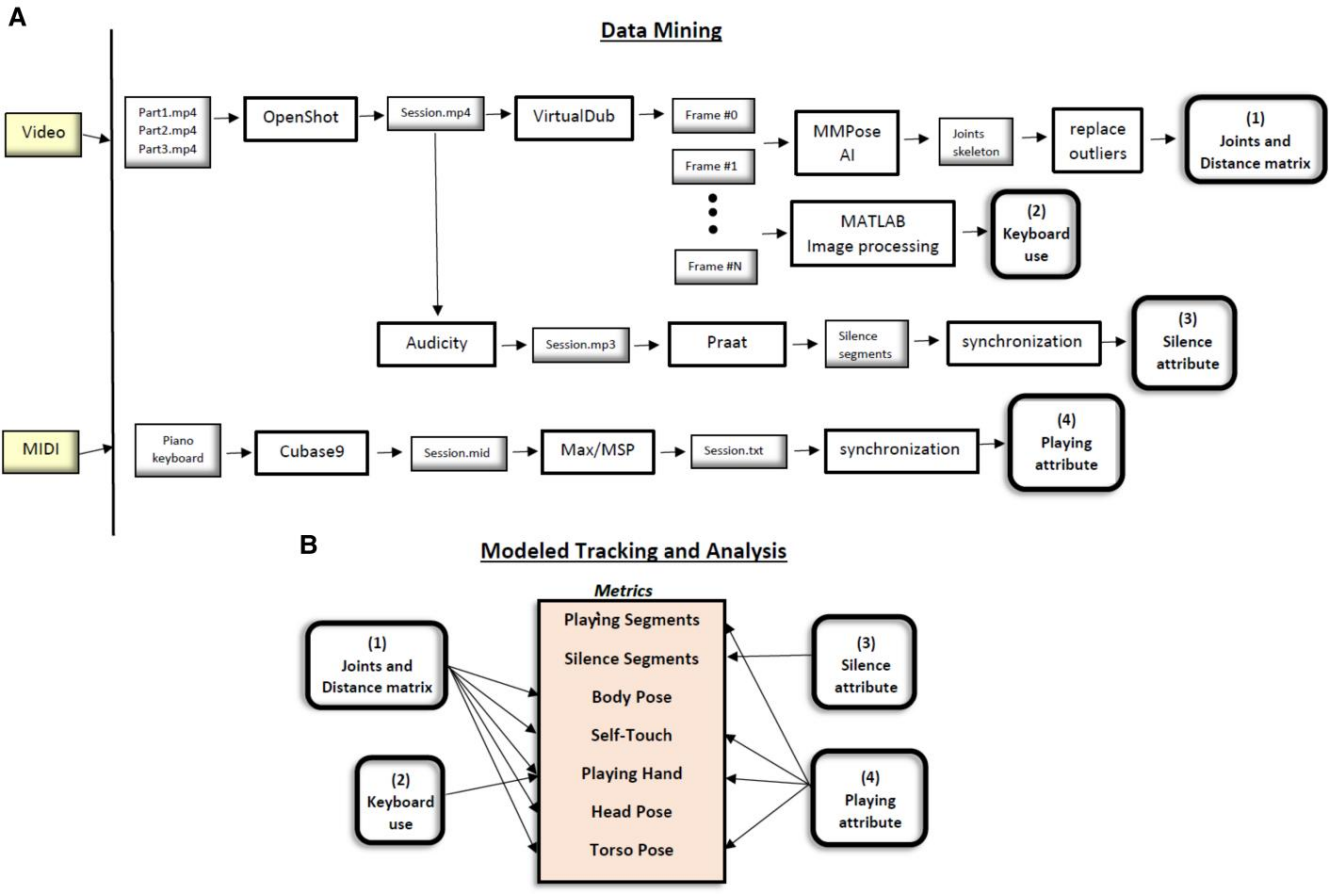
A) A detailed scheme of the data mining procedures to process, select, and translate the raw data. B) The processed data of procedures (1)–(4) are then used by the method's modeled tracking and analysis algorithms and heuristics to identify and track the behavioral metrics and to analyze their values.

**Fig. 3.**
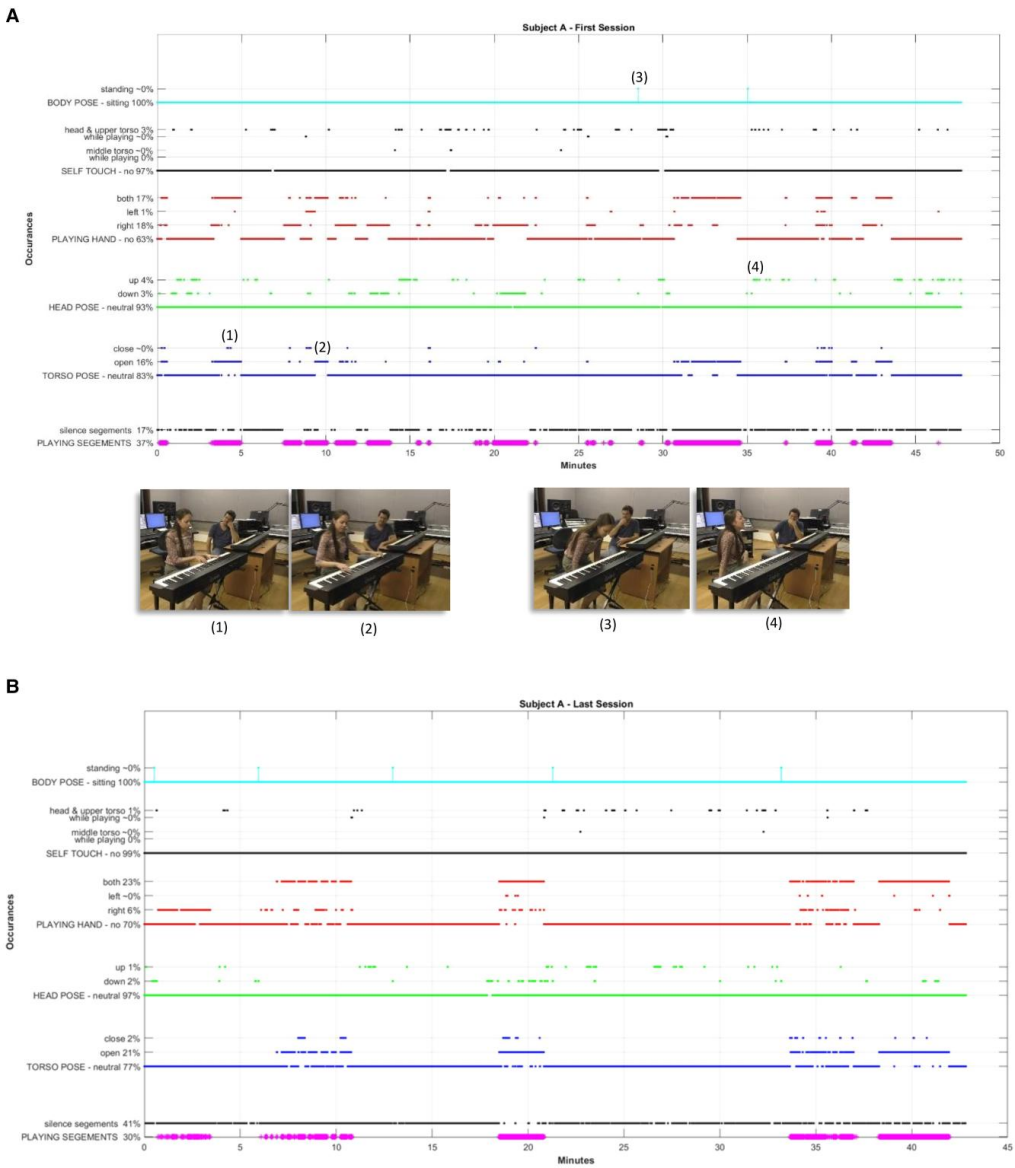
Graphic imaging of Subject A's nonverbal behavior during an entire session. The abscissa is the session's time line in minutes, where the ordinate depicts the metrics and their computed session's statistics. A) The first session of Subject A. The marked occurrences are depicted with their respective video frames. Exemplification of (1) torso pose of an open hands configuration; (2) torso pose of a closed form; (3) rising; and (4) head pose in an open form. B) The last session of Subject A.

**Fig. 4.**
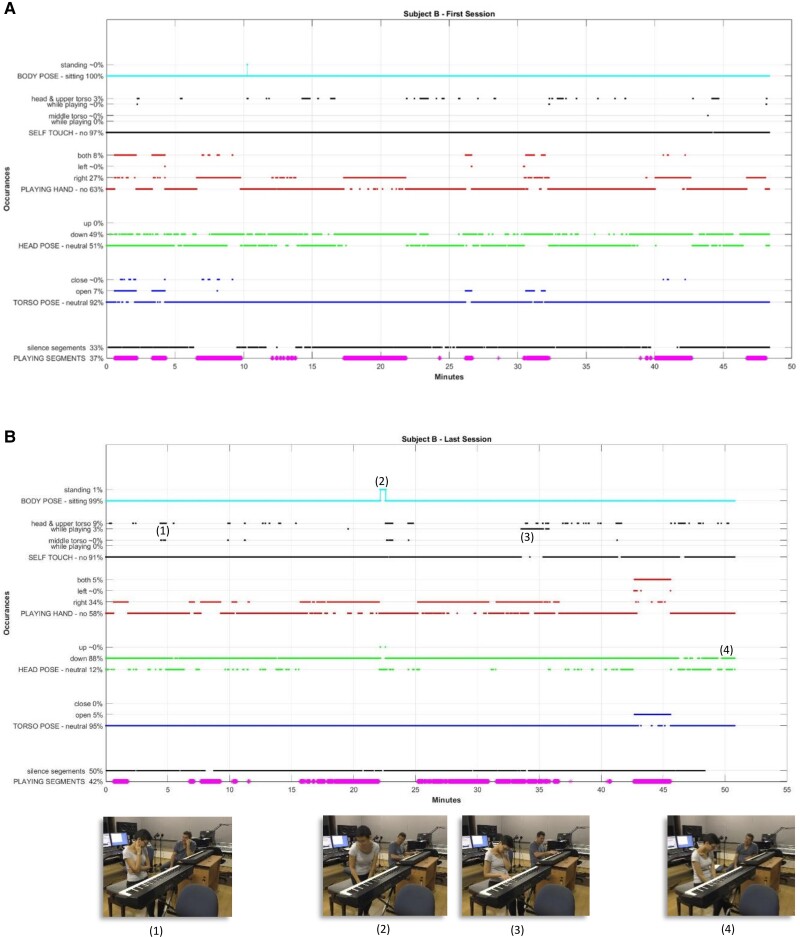
Graphic imaging of Subject B's nonverbal behavior during an entire session. The abscissa is the session's time line in minutes. The ordinate depicts the metrics and their computed session's statistics. A) The first session of Subject B. B) The last session of Subject B. Exemplified occurrences are depicted with their respective video frames. Depiction of (1) self-touch of head and middle torso; (2) standing and turning away from the keyboard; (3) self-touch of head while playing with the right hand; and (4) head pose in the down form.

The video input data are frame-based; that is, around 180k video frames (with a frequency of 50 frames per second) are generated from a video-recorded session of around an hour. The tools we have developed for mining the data include data processing, selection, and translation (see Fig. 2A). We also have synchronized the playing epochs recorded via MIDI with the video's frames, in order to extract the piano-playing segments, as well as the silence segments. As shown in Fig. 2B, we then computationally track, extract, and analyze the client's bodily behavior, e.g. body language and postures of interest, from the data about the joints and by using image processing techniques. For identifying body poses, we have defined measurements that include the many-to-many distances between the joints (from each joint to every other joint) and then document the extracted bodily features in a graphical representation for the full session. See, for example, the graphs in Figs. 3 and 4, which depict the metrics over time. In addition, we have developed a dashboard tool for interactively analyzing the sessions imaged, as displayed in Fig. S1 and Video S1. An example of the metrics quantification is given in Table 2.

**Table 2.** The subjects' behavioral metrics measurements.

Attribute	Subject A First session (%)	Subject A Last session (%)	Subject B First session (%)	Subject B Last session (%)	Subject A average (%)	Subject B average (%)
Playing segments	37	30	37	42	33.5	39.5
Silence segments	17	41	33	50	29	41.5
Body pose						
Standing	∼0	∼0	∼0	1	∼0	0.5
Sitting	∼100	∼100	∼100	99	∼100	99.5
Self-Touch						
Head and upper torso	3	1	3	9	2	6
while playing	∼0	∼0	∼0	3	∼0	1.5
Middle torso	∼0	∼0	∼0	∼0	∼0	∼0
while playing	0	0	0	0	0	0
No self-touch	97	99	97	91	98	94
Playing hand						
Both	17	23	8	5	20	6.5
Left	1	∼0	∼0	∼0	0.5	∼0
Right	18	6	27	34	12	30.5
Not playing	63	70	63	58	66.5	60.5
Head pose						
Up	4	1	0	∼0	2.5	0
Down	3	2	49	88	2.5	68.5
Neutral	93	97	51	12	95	31.5
Torso pose						
Closed	∼0	2	∼0	0	1	0
Open	16	21	7	5	18.5	6
Neutral	83	77	92	95	80	93.5

#### Detailed description

The computational components of the method, as shown in Fig. 2A and B, are now described in detail:

The data mining procedures (see Fig. 2A):

1. *Joints and distance matrix*: The camera (Panasonic full HD HC-V785) stores the video recording of a typical 50-min-long session as three part mp4 files (with a maximum length of 4.2 MB, i.e. 22:40 min recorded time enabled for each file). These are merged into a single file using the OpenShot (63) software. This file in an input file to VirtualDub (64), which outputs the video as individual frames with a frequency of 50 frames per second, that is, one video frame every 20 ms, resulting in 150K frames for the typical 50-min session (depending, of course, on its actual length). These frames are the input to the MMPose (65) package, which outputs the estimation of the client and therapist's pose, as skeleton joint structures for each frame (see Fig. 1B). The skeleton's joints that are scarcely and illogically displaced by MMPose, named here as outliers, are replaced by the preceding 20 milli frame's joints. From these joints, the many-to-many matrix of the distances between the 17 joints is then computed for each frame: nose, eyes, ears, shoulders, elbows, wrists, hips, knees, and ankles (see Fig. 1C for the joint scheme).
2. *Keyboard use*: An indication of the client's keyboard use for each frame is estimated using MATLAB's image processing package (66) for computing the percentage of the “white” keys’ pixels (white and its adjacent red, green and blue tones) and in which part of the keyboard. When the keyboard is played on or being touched, the number of identified white pixels decreases as the client's hand(s) occlude the keys.
3. *Silence attribute*: The silence epochs are extracted by first translating the video recording into mp3 audio file (using the Audacity software (67)) and then using the Praat package (68) to identify the silences. The silence segments are synchronized with the video frames timeline, marking those where silence is detected.
4. Playi*ng attribute*: Actual playing is identified by first recording the session using Cubase (69) and then using the Max/MSP software package (70) to translate the MIDI file format into a regular text file for further manipulation. These data are synchronized temporally with the video frames, marking the frames in which the client was actually using the keyboard and playing.

The modeled tracking and analysis procedure (see Fig. 2B and Tables 1 and 2):

- Playing segments—determined by procedure (4).
- Silence segments—determined by procedure (3).
- Body pose—determined by computing joints distance from sitting position.
- Self-touch—determined by computing the spatial position of the wrists in relation to the body parts, derived from the distance matrix (1) and playing attribute (4) (this is necessary since there can be states where the client in engaged both in playing and self-touch).
- Playing hand—determined by computing the spatial wrist position in relation to the keyboard, as derived from the distance matrix (1) and playing attribute (4).
- Head pose—determined by the computation of angles of the head's joints in space (nose, ears), and evaluating the head tilt.
- Torso pose—determined by the distances between the shoulders and the elbows from the distance matrix (1), as well as whether the client is playing (4) and whether the playing is with both hands.

### Experimental design

The one-on-one study included a music therapist playing on a piano keyboard and a participant playing on a separate keyboard. Each one of the four subjects participated in six sessions of ∼50 min long, each of which began and ended with a free improvisation. In between, the subjects were given exercises and tasks by the therapist, to execute alone or accompanied by him, with the aim of improving their creativity and expressivity.

An open discussion was held after each improvisatory exercise, in order to gain insight into the creative and expressive processes that took place during the improvisation. See the detailed and formal protocol in Fig. S2.

The sessions were not defined to the subjects as therapeutic. However, they did carry a flow similar to music therapy sessions and thus served as a good model to simulate the musical, verbal, and gestural occurrences that are typical in musical therapy sessions. The participants were healthy/normal subjects, 22- to 35-y-old females, having had college-level musical education as well as several years of piano training, and modest experience in improvisation. The musical instrument used by the participants was a Casio MIDI piano keyboard controller (PX-160) and a pedal (sp-3).

### Ethics declaration

The research protocols were reviewed and approved by the Weizmann Institute's Bioethics and Embryonic Stem Cell research Oversight Committee and Bar-Ilan University's Ethics Committee. All participants signed a written informed consent. Informed consent was obtained to publish the information/image(s) in an online open access publication. All methods were performed in accordance with the relevant guidelines and regulations.

## Results

Our extended CP is applied in a proof-of-concept study showing its feasibility on two subjects, referred to as Subject A and Subject B. The body language of a client relevant to music therapy (46, 47, 53, 54) is carefully considered and learned idiosyncratically and is then used as a point of reference to other sets of information (e.g. the music that the client makes, the themes that s/he brings up), and in comparison between different points of time during the clinical process. We demonstrate how we obtain new empirical insights, compare the quantitative results with the therapist's notes, and generate hypotheses relating to the emotional states of the client, for which further clinical validation is described in the Discussion section. We first exemplify the analysis of the subjects’ body positions in single specific sessions that can be clinically indicative and useful to the music therapist (intra-session analysis), and then demonstrate how the method can be used to compare the body positions between sessions and between subjects (inter-session analysis).

### Intra-session analysis (micro-analysis)

The occurrences captured and analyzed for Subjects A and B in several sessions are depicted in Figs. 3 and 4, respectively, and the total statistics are summarized in Table 2. The first session of Subject A was characterized by openness in playing with both hands in open torso form, during a significant part of the session, i.e. 16%. Adding to this was the head pose, in an up pose for 4% of the time, and talking or playing (not silent) around 83% of the session. Such data are useful for music therapists to understand in what emotional state the client was in this session, to use it as baseline for further sessions, or to cross examine it with his or her subjective impressions. The music therapist noted on this session that he felt the client was “afraid of how her music will sound” and “that it won't turn out well” (see Fig. S3). The body position data, on the contrary, indicated openness, which can challenge the music therapist's subjective feelings and enable other competing clinical hypotheses to surface.

Subject B's last session was in a down head pose in an extremely significant part of the session (88%) and playing mostly with the right hand. She was also engaged in self-touch 9% of the time, touching either her head, upper, or middle torso, with about a third of this time carried out while playing. A notable occurrence is the Subject B standing and walking away from the keyboard for a period of around a minute (as was also revealed by the interactive dashboard tool exemplified in Fig. S1 and Video S1). The subject played with both hands during her last improvisation, at time 42 min in an open torso form. Following that, and proceeding until the end of the session, she was engaged in a conversation. Put together, these data portrays the possible uneasiness of the client was during the session. This hypothesis can then be examined in comparison with the same measures in other sessions, to see whether they are consistent, notably with the first baseline session. The music therapist can use this information to explicitly ask the client if she felt uneasy for any reason, and if so to see whether this could, or should, be discussed and in future sessions, and possibly take appropriate steps to alleviate it.

### Inter-session analysis (macro-analysis)

As shown in Fig. 3, Subject A rose five times during the last session. Playing was carried out with both hands in an open torso pose for about 20% of the session time. The significant silence during these periods manifested relative slow playing. These numbers are different from the baseline measures in the first session and could allow the music therapist to examine whether goals such as increasing vitality and boldness were achieved in the sessions. In his notes on the last session (Fig. S3), the music therapist indeed refers to some improvement that the client reported of, though with a great deal of reservation. Had he shown her the data from her body position analysis, it could have added impetus and validity to her feeling.

Subject B's last session shows an increase in some metrical measures as compared to her first (Fig. 4). In the first session, self-touch was present to a lesser extent than in the last session, as well as the down head pose, playing with a single hand, and silence segments. This could alert the music therapist to a regression in the uneasiness of the client that was noted in the first session. It should also be interpreted in the context of other measures that might have changed during the following sessions, such as increases in creative explorations, which might, at first, affect one's (bodily) comfort (71, 72). Music therapists could use such information as a basis for a dialogue with the client, in which such issues can be clarified.

In addition to inter-session comparison, some behavioral attributes repeating themselves may point to personal patterns and characteristics (73, 74), as given in the last two columns of Table 2, averaging the emerged metrics. Subject B was less talkative than Subject A, as manifested from both the silence and playing segments. Notable bodily occurrences that were more intensive for Subject B and less so for Subject A, are self-touch, down head pose, and playing with one hand. Such personal patterns can be reflected to the client, who might not be aware of them, and thereafter, decided whether they should be treated (increased, diminished) as part of the process.

## Discussion

To address the challenges that the dynamic environment of music therapy imposes on understanding its effects, in this paper we have expanded our CP (34–37) to track, analyze, and document the bodily and nonverbal behavior of the client. Adding to these is imaging the session in a graphical format and gearing its notated and concise reporting toward the development of a domain-specific language, aimed at conveying and disseminating these understandings. That is, when a commonly accepted language is adopted within a specific area of expertise, it opens up abundant avenues for communication and comprehension among experts and communities within the pertinent fields of that domain.

Comparing the model's results with the written notes of the therapist (Fig. S3) shows that the method makes it possible to capture and analyze phenomena missed by the human eye (e.g. self-touch and head pose). Whereas the therapist's evaluation of the sessions is usually qualitative, we enable an objective, quantitative measurement of psychometrics, which complements the therapist's notes and thus provides additional and often novel insights. In way of extending our current reporting, we describe further validation steps for the behavioral metrics in studies described in detail in Experiment S1, which mainly upscale the number of clients, music therapists, and sessions investigated. Upon their further validation, the metrics may facilitate a quantitative assessment of progress or regression of a client and may be considered as idiosyncratic “behavioral markers,” e.g. in identifying turning points (75–77).

We have illustrated the feasibility and advantages of our method. Nevertheless, issues that further need to be addressed are the acceleration of some computational aspects of the technology, since the AI component of pose estimation is still time- and space-consuming. In addition, for applying the approach in a real-world clinical setting, the therapist may need to assimilate it as part of the session and the client's evaluation process, and the client, to consent to be videoed.

The system is flexible, in that in enables different therapists to ask it to focus and analyze those bodily expressions they find important, thus tailoring it for different clinical scenarios and therapeutic approaches. This causes us to believe that the method can be utilized in other therapy fields, such as art therapy and psychotherapy.

Capturing bodily and nonverbal occurrences, in addition to the rigorous enablement of tracking and documenting the art and musical work itself (34–37), meets the challenge of investigating the first two components in the triangular relations of “client—musical work—therapist.” Potential avenues for future work include the interaction between the therapist and client, e.g. their bodily synchronization, additional nonverbal metrics, e.g. facial expressions, and the expansion of the CP to be used in more complex music therapy settings that involve choice of various playing instruments.

## Conclusion

Our method provides a useful framework for understanding and analyzing the complex dynamics within the clinical setting. It offers a means to quantitatively track and document expressive and social behavioral processes, facilitating empirical investigations and knowledge discovery. Through intra-/local-/micro-analysis and inter-/global-/macro-analysis, researchers can gain a deeper understanding of the therapeutic process and its underlying mechanisms, with possible applicability in other relevant fields too.

## Supplementary Material

pgae475_Supplementary_Data

## Data Availability

Due to ethical restrictions imposed by the institutional review board, the authors cannot deposit the data publicly. However, all approved data and materials are within the manuscript and the Supplementary Material file.

## References

[1] Bruscia KE . 2014. Defining music therapy. 3rd ed. Barcelona Publishers.

[2] Bunt L, Stige B. 2004. Music therapy: an art beyond words. 2nd ed. Routledge.

[3] Dileo EC . 2006. Effects of music and music therapy on medical patients: a meta-analysis of the research and implications for the future. J Soc Integr Oncol. 4:67–70. 19442338 10.2310/7200.2006.002

[4] Burns S, Harbuz M, Hucklebridge F, Bunt AA. 2001. Pilot study into the therapeutic effects of music therapy at a cancer help center. Altern Ther Health Med. 7:48–57. 11191042

[5] Guzzetta C . 1989. Effects of relaxation and music therapy in a coronary care unit with presumptive acute myocardial infaction. Heart Lung. 18:609–616. 2684920

[6] Pacchetti C, et al 2009. Active music therapy in Parkinson's disease: an integrative method for motor and emotional rehabilitation. Psychosom Med. 62:386–393. 10.1097/00006842-200005000-00012 10845352

[7] Hilliard R . 2003. The effects of music therapy on the quality and length of life of people diagnosed with terminal cancer. J Music Ther. 40:113–137. 14505443 10.1093/jmt/40.2.113

[8] Gold C, Solli H, Krüger V, Lie S. 2009. Dose-response relationship in music therapy for people with serious mental disorders: systematic review and meta-analysis. Clin Psychol Rev. 29:193–207. 19269725 10.1016/j.cpr.2009.01.001

[9] Hense C, McFerran KS. 2017. Promoting young people's musical identities to facilitate recovery from mental illness. J Youth Stud. 20:997–1012.

[10] Skeja E . 2014. The impact of cognitive intervention program and music therapy in learning disabilities. Procedia Soc Behav Sci. 159:605–609.

[11] Chanda ML, Levitin DJ. 2013. The neurochemistry of music. Trends Cogn Sci. 17:179–193. 23541122 10.1016/j.tics.2013.02.007

[12] Lindblad F, Hogmark Å, Theorell T. 2007. Music intervention for 5th and 6th graders—effects on development and cortisol secretion. Stress Health. 23:9–14.

[13] Smolen D, Topp R, Singer L. 2002. The effect of self-selected music during colonoscopy on anxiety, heart rate, and blood pressure. Appl Nurs Res. 15:126–136. 12173164 10.1053/apnr.2002.34140

[14] Bleibel M, El Cheikh A, Sadier NS, Abou-Abbas L. 2023. The effect of music therapy on cognitive functions in patients with Alzheimer's disease: a systematic review of randomized controlled trials. Alzheimers Res Ther. 27:65. 10.1186/s13195-023-01214-9 PMC10041788 36973733

[15] Zhao K, Bai ZG, Bo A, Chi IA. 2016. Systematic review and meta-analysis of music therapy for the older adults with depression. Int J Geriatr Psychiatry. 31:1188–1198. 27094452 10.1002/gps.4494

[16] Chang Y, et al 2015. The efficacy of music therapy for people with dementia: a meta-analysis of randomized controlled trials. J Clin Nurs. 24:3425–3440. 26299594 10.1111/jocn.12976

[17] Ghai S . 2023. Does music therapy improve gait after traumatic brain injury and spinal cord injury? A mini systematic review and meta-analysis. Brain Sci. 13:522. 36979332 10.3390/brainsci13030522 PMC10046548

[18] Nuria N, et al 2023. Music therapy to reduce pain intensity in post fracture surgery patients: systematic review. Int J Biomed Nurs Rev. 1:101–124.

[19] Wang C, Sun Y, Zang H. 2014. Music therapy improves sleep quality in acute and chronic sleep disorders: a meta-analysis of 10 randomized studies. Int J Nurs Stud. 51:51–62. 23582682 10.1016/j.ijnurstu.2013.03.008

[20] Ansdell G, Stige B. 2016. Community music therapy. In: Edwards J, editor. The Oxford handbook of music therapy. Oxford University Press. p. 595–621.

[21] Tuastad L, Stige B. 2015. The revenge of me and THE BAND’its: a narrative inquiry of identity constructions in a rock band of ex-inmates. Nord J Music Ther. 24:252–275.

[22] Kuusea A, Paulanderb A, Eulau L. 2023. Characteristics and impacts of live music interventions on health and wellbeing for children, families, and health care professionals in paediatric hospitals: a scoping review. Int J Qual Stud Health Well-being. 18:2180859. 36880806 10.1080/17482631.2023.2180859 PMC10013212

[23] Gilboa A, Yehuda N, Amir D. 2019. Let's talk music: a musical-communal project for enhancing communication among students of multi-cultural origin. J Music Ther. 18:3–31.

[24] Gilboa A, Salman B. 2018. The roles of music in Let's talk music, a model for enhancing communication between Arabs and Jews in Israel. J Music Ther. 18:1–13.

[25] Pavlicevic M, Ansdell G. 2004. Community music therapy. Jessica Kingsley Publishers.

[26] Carnovalini F, Rodà A, Caneva P. 2023. A rhythm-aware serious game for social interaction. Multimed Tools Appl. 82:4749–4771.

[27] Stige B . 2011. Elaborations toward a notion of community music therapy. Barcelona Publishers.

[28] Stige B, Aaro LE. 2011. Invitation to community music therapy. Routledge.

[29] Stige B, Ansdell B, Elefant C, Pavlicevic M. 2010. Where music helps: community music therapy in action and reflection. Ashgate Publishing Company.

[30] Wosch T, Wigram T. 2007. Microanalysis in music therapy. Jessica Kingsley Publishers.

[31] Wheeler B . 2015. Handbook of music therapy. Guilford Publications.

[32] Greenberg LS . 1994. The investigation of change: its measurement and explanation. In: Russell RL, editor. Reassessing psychotherapy research. The Guilford Press. p. 114–143.

[33] Juslin P, Sloboda J. 2001. Handbook of music and emotion—theory, research, applications. Oxford University Press.

[34] Sandak B, Huss E, Sarid O, Harel D. 2015. Computational paradigm to elucidate the effects of arts-based approaches and interventions: individual and collective emerging behaviors in artwork construction. PLoS One. 10(6):e0126467. 26061736 10.1371/journal.pone.0126467 PMC4489499

[35] Sandak B, Cohen S, Gilboa A, Harel D. 2019. Computational elucidation of the effects induced by music making. PLoS One. 14(3):e0213247. 30845183 10.1371/journal.pone.0213247 PMC6405055

[36] Sandak B, Mazor A, Asis A, Gilboa A, Harel D. 2019. Computational music therapy. In: Montiel M, Gomez-Martin F, Agustin-Aquino O, editors. Mathematics and computation in music. Cham: Springer. p. 359–368.

[37] Sandak B, Gilboa A, Harel D. 2020. Computational paradigm to elucidate the effects of arts-based approaches and interventions: art and music studies and implication for research and therapy. Front Psychol. 11:1200. 32595563 10.3389/fpsyg.2020.01200 PMC7300292

[38] Wallbott HG . 1998. Bodily expression of emotion. Eur J Soc Psychol. 28:879–896.

[39] Atkinson AP, Tunstall ML, Dittrich WH. 2007. Evidence for distinct contributions of form and motion information to the recognition of emotions from body gestures. Cognition. 104(1):59–72. 16831411 10.1016/j.cognition.2006.05.005

[40] De Gelder B . 2009. Why bodies? Twelve reasons for including bodily expressions in affective neuroscience. Philos Trans R Soc Lond B Biol Sci. 364(1535):3475–3484. 19884142 10.1098/rstb.2009.0190 PMC2781896

[41] De Gelder B, Van den Stock J. 2011. The bodily expressive action stimulus test (BEAST). construction and validation of a stimulus basis for measuring perception of whole body expression of emotions. Front Psychol. 2:181. 21886632 10.3389/fpsyg.2011.00181 PMC3152787

[42] Aviezer H, Trope Y, Todorov A. 2012. Body cues, not facial expressions, discriminate between intense positive and negative emotions. Science. 338:1225–1229. 23197536 10.1126/science.1224313

[43] Carney DR, Hall JA, LeBeau LS. 2005. Beliefs about the nonverbal expression of social power. J Nonverbal Behav. 29:105–123.

[44] Calbi M, Langiulli N, Siri F, Umiltà MA, Gallese V. 2021. Visual exploration of emotional body language: a behavioural and eye-tracking study. Psychol Res. 85:2326–2339. 32920675 10.1007/s00426-020-01416-y

[45] Hall JA, Horgan TG, Murphy NA. 2019. Nonverbal communication. Ann Rev Psychol. 70:271–294. 30256720 10.1146/annurev-psych-010418-103145

[46] Dvir T, Lotan N, Viderman R, Elefant C. 2020. The body communicates: movement synchrony during music therapy with children diagnosed with ASD. Arts Psychother. 69:101658.

[47] Hibben JK . 1984. Movement as musical expression in a music therapy setting. Music Ther. 4(1):91–97.

[48] Ramsey F, Tschacher W. 2014. Nonverbal synchrony of head- and body-movement in psychotherapy: different signals have different associations with outcome. Front Psychol. 5:979. 25249994 10.3389/fpsyg.2014.00979 PMC4155778

[49] McColl D, Nejat G. 2014. Determining the affective body language of older adults during socially assistive HRI. IEEE Int Conf Intell Robots Syst. 2633–2638.

[50] Yang Z, Kay A, Li Y, Cross W, Luo J. 2020. Pose-based body language recognition for emotion and psychiatric symptom interpretation. Proc Int Conf Patt Rec. 294–301.

[51] Gilboa A, Bensimon M. 2007. Putting clinical process into image: a method for visual representation of music therapy sessions. Music Ther Perspect. 25:32–42.

[52] Gilboa A . 2007. Testing the MAP: a graphic method for describing and analyzing music therapy sessions. Arts Psychother. 34:304–320.

[53] Bergstrom-Nielsen C . 1993. Graphic notation as a tool in describing and analyzing music therapy improvisations. Music Therapy. 12:40–58.

[54] Bergstrom-Nielsen C . 2009. Graphic notation in music therapy: a discussion of what to notate in graphic notation and how. Approaches Music Ther Spec Music Educ. 1:72–92.

[55] Cohen S, Gilboa A, Bergstrøm-Nielsen C, Leder R, Milstein Y. 2012. A multiple-perspective approach to graphical notation. Nord J Music Ther. 21:153–175.

[56] Gilboa A . 2012. Developments in the MAP: a method for describing and analyzing music therapy sessions. Nord J Music Ther. 21:57–79.

[57] Giannakakis G, Manousos D, Chaniotakis V, Tsiknakis M. 2018. Evaluation of head pose features for stress detection and classification. IEEE EMBS International Conference on Biomedical & Health Informatics (BHI); Las Vegas, NV, USA. p. 406–409.

[58] Barroso F, Feld JK. 1986. Self-touching and attentional processes: the role of task difficulty, selection stage, and sex differences. J Nonverbal Behav. 10:51–64.

[59] Grunwald M, Weiss T, Mueller S, Rall L. 2014. EEG changes caused by spontaneous facial self-touch may represent emotion regulating processes and working memory maintenance. Brain Res. 1557:111–126. 24530432 10.1016/j.brainres.2014.02.002

[60] Takayama A, Sekiya H. 2023. Effects of various sitting and standing postures on arousal and valence. PLoS One. 18(6):e0286720. 37267405 10.1371/journal.pone.0286720 PMC10237498

[61] Sun K, Xiao B, Liu D, Wang J. 25 February 2019. Deep high-resolution representation learning for human pose estimation. arXiv 09212. 10.48550/arXiv.1902.09212, preprint: not peer reviewed.

[62] Lin TY, et al July 2024. Microsoft COCO: Common objects in context. arXiv 0312. 10.48550/arXiv.1405.0312, preprint: not peer reviewed.

[63] OpenShot . Video editor. [accessed 2024 Oct 26]. https://www.openshot.org.

[64] VirtualDub . Video processing utility. [accessed 2024 Oct 26]. https://virtualdub.org/.

[65] MMPose . Toolbox for pose estimation. [accessed 2024 Oct 26]. https://github.com/open-mmlab/mmpose.

[66] MATLAB (Mathworks) . The language of technical computing / image processing toolbox. [accessed 2024 Oct 26]. https://www.mathworks.com/products/image.html.

[67] Audicity . Cross-platform audio software. [accessed 2024 Oct 26]. https://www.audacityteam.org/.

[68] Praat . Speech analysis software. [accessed 2024 Oct 26]. https://www.fon.hum.uva.nl/praat/.

[69] Cubase9 . Digital audio workstation. [accessed 2024 Oct 26]. https://www.steinberg.net/cubase/.

[70] Max/MSP . Visual programming language for music and multimedia. [accessed 2024 Oct 26]. https://cycling74.com/products/max.

[71] Daker RJ, Cortes RA, Lyons IM, Green AE. 2020. Creativity anxiety: evidence for anxiety that is specific to creative thinking, from STEM to the arts. J Exp Psychol Gen. 149:42–57. 31219299 10.1037/xge0000630

[72] Daker RJ, Viskontas IV, Porter GF, et al 2023. Investigating links between creativity anxiety, creative performance, and state-level anxiety and effort during creative thinking. Sci Rep. 13:17095. 37816728 10.1038/s41598-023-39188-1 PMC10564955

[73] Costa PT, McCrae RR. 1992. Four ways five factors are basic. Pers Individ Dif. 13(6):653–665.

[74] Matthews G, Deary IJ, Whiteman MC. 2003. Personality traits. 2nd ed. Cambridge: Cambridge University Press.

[75] Bell B . 2002. Moments of change in art therapy process. Arts Psychother. 29:79–92.

[76] McLean CL . 2014. Creative arts in humane medicine. Brush Education, University of Toronto Press.

[77] Jones P . 2020. The arts therapies: a revolution in healthcare. 2nd ed. Brunner- Routledge.

