# Supplementary material for: Computational elucidation of nonverbal behavior and body language in music therapy
Source: PNAS Nexus. 2024 Oct 22;3(11):pgae475. doi: 10.1093/pnasnexus/pgae475 (PMC11586667; doi:10.1093/pnasnexus/pgae475)
Supplement: pgae475_Supplementary_Data [file pgae475_supplementary_data.zip › PNASNEXUS-PNASNEXUS-2024-00393R-s01.pdf]

# Supplementary Material

# Computational Elucidation of Non-Verbal Behavior and Body Language in Music Therapy

Billie Sandak[1,*], Avi Gilboa[2], David Harel[1]

[1] Department of Computer Science and Applied Mathematics, Faculty of Mathematics and Computer Science, The Weizmann Institute of Science, Rehovot, Israel

[2] Department of Music, The Faculty of Humanities, Bar-Ilan University, Ramat-Gan, Israel

[*] **Correspondence:**
Billie Sandak
billie.sandak@alumni.weizmann.ac.il

## Content

**Figure S1 - The interactive interface of the behavioral analysis dashboard**.

**Video S1 - Behavioral analysis dashboard in action.**

**Figure S2 – The music therapy sessions' protocol.**

**Figure S3 – The music therapist's evaluation notes – summary part.**

**Experiments S1 – Further Clinical Validation of Behavioral Metrics (Table 1).**

**Datasets S1 – MIDI recordings of Subjects A and B.**

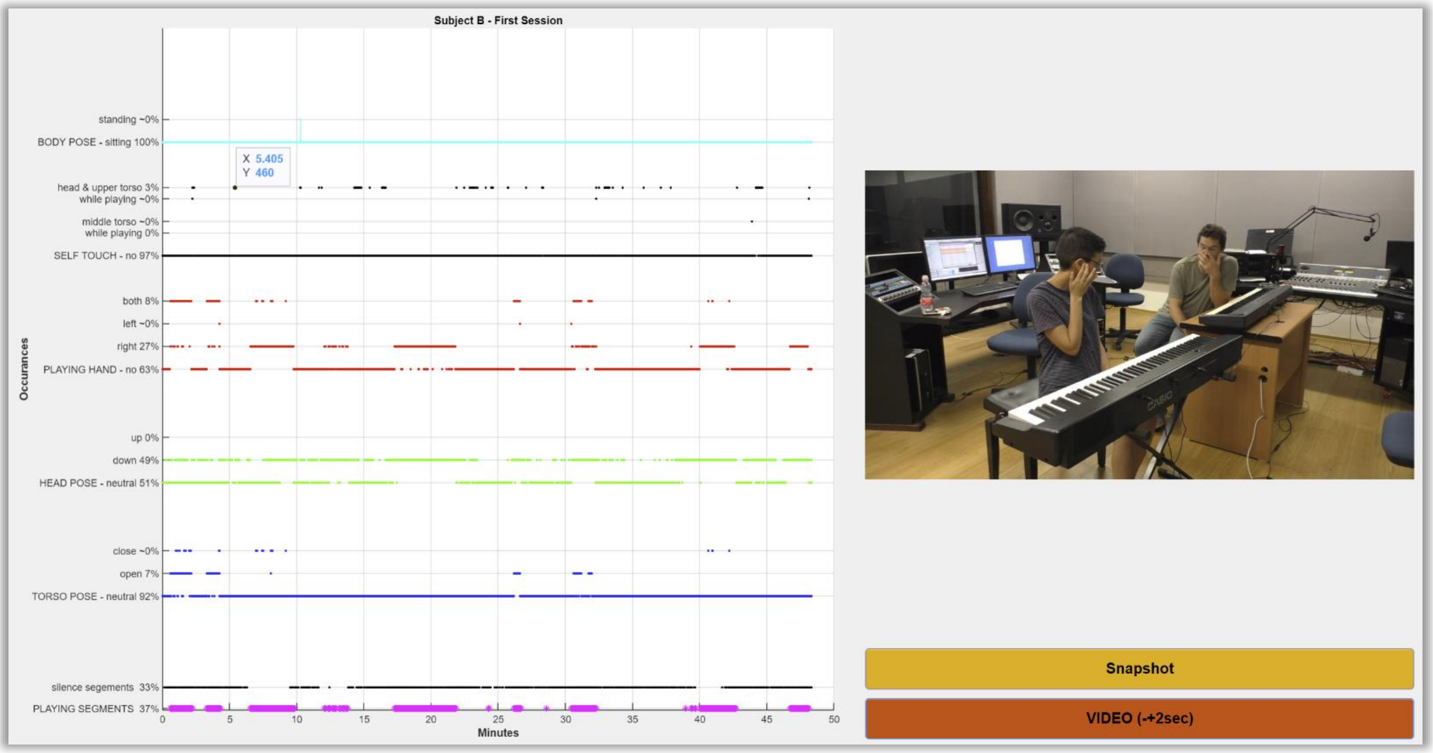


**Figure S1. The interactive interface of the behavioral analysis dashboard**. On the left hand side of screen is the graphic representation of an entire session, with a chosen data point shown, corresponding to the video frame depicted on the right. The frame is generated by pressing the "Snapshot" button. Pressing the "VIDEO" button will generate a 4-second video clip, from 2 sec before the data point event and until 2 sec after.

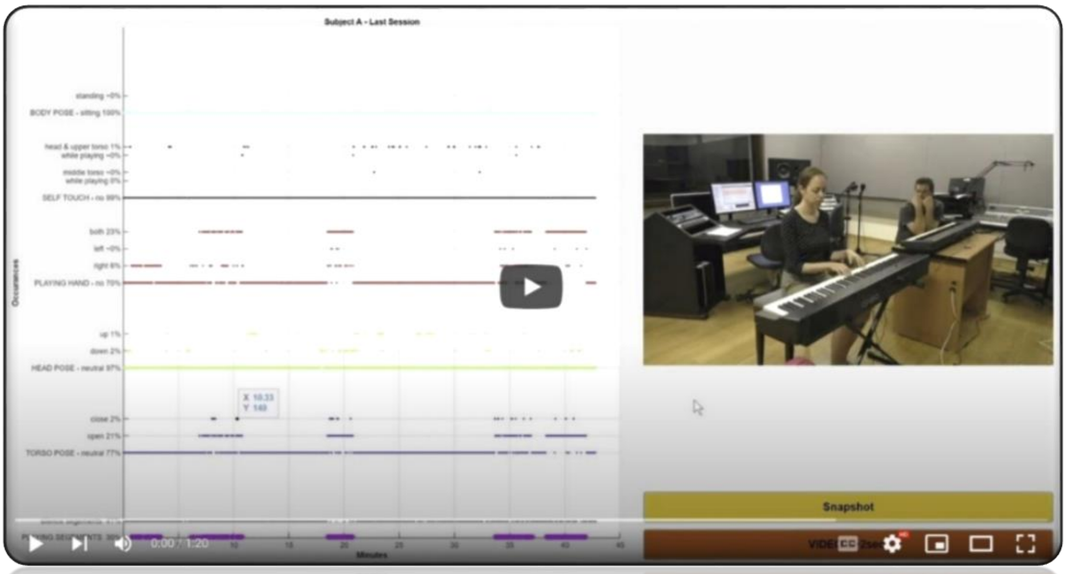


**Video S1. Behavioral analysis dashboard in action**. https://youtu.be/VM9FlZtcCK4

**Protocol**

**First session - acquaintance and evaluation**

1. Free Improvisation - “Opening” (5 minutes)
2. Improvisation on the black keys only - "blues to reduce anxiety” in Eb minor
3. Improvisation on the white keys only - C major / D Dorian (if necessary)
4. “Drumming on the keyboard” - play on the piano like percussion instrument
5. “Rain drops” improvisation [fixed bass (“organ-point”) on left hand and very slow playing on the right hand with sustain pedal]
6. Emotional improvisation - emotional words as stimulation (for evaluation)
7. Free Improvisation - “Ending” (5 minutes)

**Second session - work on musical parameters**

1. Free Improvisation - “Opening” (5 minutes)
2. Using the keyboard - “Drumming on the keyboard”, “Rain-drops” improvisation, playing just with seconds (minor or major)
3. Dynamics - play with different dynamics
4. Tempo - play with varying tempi
5. Articulation - play with Legato and Staccato
6. Use the entire range of the keyboard (low, high, middle)
7. Atonal improvisation – play with no tonality
8. Free Improvisation - “Ending” (5 minutes)

**Third session “Alone and Together” – interpersonal interaction and inwards (musician meditative mode)**

1. Free Improvisation - “Opening” (5 minutes)
2. Joint exercises (leader-follower, solo-companion, question-answer, low-high, one track the other)
3. Individual playing while trying to reach a meditative state (no planning, no thoughts, no awareness)
4. Free Improvisation - “Ending” (5 minutes)

**Fourth session - improvisation around artistic/visual stimuli**

1. Free Improvisation - “Opening” (5 minutes)
2. Photo / painting improvisations (improvising while looking at a picture or a painting)
3. Free Improvisation - “Ending” (5 minutes)

**Fifth session - work on emotional content**

1. Free Improvisation - “Opening” (5 minutes)
2. Improvisation on random words (“birds”, “sea”, “forest”, “rain”, “wind”, “storm”, and so on’)
3. Improvisation on emotional words (״joy”, “sadness”, “home”, “family", “faith”, and so on’)
4. Improvisation on emotional experience (an emotional event that comes up through guided imagery)
5. Character improvisation (improvisation of a character - friend, or family member)
6. Free Improvisation - “Ending” (5 minutes)

**Sixth session - – summary meeting**

1. Free Improvisation - “Opening” (5 minutes)
2. A feedback conversation with the client
3. At the request of the client – come back and repeat meaningful exercises
4. Improvisation on the words: “ending” or “separation”
5. Free Improvisation - “Ending” (5 minutes)

**Alternative exercises - improvisation with limitations**

1. Improvisation on one note, two notes, three notes, and four notes
2. Improvisation with octave doubling (melody with two hands)
3. Improvisation in minor or major seconds
4. Improvisation of harmonic “blocks” on one modus only
5. A hand on the black keys and a hand on the White keys
6. Atonal improvisation

**Figure S2 – The sessions’ protocol.**

**Subject A – First Session**

I feel that Subject A "is afraid of how her music will sound", "that it won't turn out well" (she said this after the last improvisation). On the one hand, she is afraid that the listener will think that the improvisation is not beautiful or good and then she is fixed, on the other hand, she is afraid to bore the listener when she plays simple or repetitive things. It seems that with a slight "push" she is able to reach very interesting places in her improvisations. She has difficulty connecting emotionally, has difficulty experiencing a "vibe" or a deep connection with the material, has difficulty detaching from critical thoughts, but on the other hand allows herself to experiment in new places, and is not fixed even when anxious (in the beginning she chose a fixed structure but within it she immediately felt great freedom).

**Subject A – Last Session**

She says that the sessions helped her a lot, but still finds it difficult to feel free, and every time she improvises, she experiences some kind of fear.

---

**Subject B – First Session**

Very blocked and very restrained. Lots of self-criticism and judgment that does not allow for playfulness. She states that she cannot "express" herself. Has difficulty with emotional expression even in other places.

**Subject B – Last Session**

In the end, she says that the sessions gave her a lot even if she could not always implement the ideas. Says mainly that she had a good time with "dressing up" in different “costumes”. I point out that it is possible that in music she should not really connect with herself because it is too heavy and too overwhelming, and she probably needs a lot of playfulness and "dressing up" in order to be able to develop her music and her ability to improvise. She needs to adopt identities from the “outside”.

**Figure S3 – The music therapist’s evaluation notes – summary part.**

**Experiments S1 – Further clinical validation of behavioral metrics (defined in Table 1).**

To further clinically validate the behavioral metrics defined in Table 1, and the system's clinical operability, we suggest the following steps (MT is the abbreviation for Music Therapist):

(1) Pilot Study.

In this experiment, ten MTs will be asked to use the system with five of their clients.
Each treatment at least ten sessions long.

Data will be collected as to the metrics and system features that the MTs uses most, those that they did not use, the ways in which the system most promoted the clinical work, and possibly the drawbacks of the system, as reported by the MTs. Data will be collected mainly by self-reports (free expression of the MTs).

Based on this pilot study, recurring statements will be pinpointed (e.g., "the system helps to uncover body gestures"; "the system is intuitive"), and a closed questionnaire will be devised for the use of the second experiment.

(2) Experiment 2.

Here, 100 MTs will try out the system with five of their clients, ten sessions each.

After each session, they will fill out the questionnaire, and add any comments they have about the system and its operationally. Analyzing this data will provide indications as to its usefulness to the MTs.

If required, this experiment can be re-iterated after revisions in the system are made, to comply with the MTs comments and suggestions.

## Datasets S1 – MIDI recordings of Subjects A and B.

10036 176 88 0;
10036 176 88 72;
10036 144 21 52;
10593 128 21 64;
11213 176 88 72;
11213 176 88 90;
11213 144 21 48;
11828 128 21 64;
12369 176 88 90;
12369 176 88 2;
12369 144 21 45;
13442 128 21 64;
288625 176 88 2;
288625 176 88 113;
288625 144 68 23;
289093 176 88 113;
289093 176 88 33;
289093 144 80 36;
289223 128 68 64;
289270 128 80 64;
296151 176 88 33;
296151 176 88 51;
296151 144 68 36;
296494 176 64 0;
296494 176 64 127;
296572 176 88 51;
296572 176 88 104;
296572 144 80 37;
296583 176 88 104;
296583 176 88 80;
296583 144 48 37;
296630 128 68 64;
296661 128 48 64;
296947 176 88 80;
296947 176 88 96;
296947 128 80 64;
296947 144 81 37;
297197 176 88 96;
297197 176 88 3;
297197 128 81 64;
297197 144 80 43;
297213 176 88 3;
297213 176 88 94;
297213 144 63 24;
297250 176 88 94;
297250 176 88 109;
297250 144 56 12;
297302 128 63 64;
297317 128 56 64;
297463 176 88 109;
297463 176 88 106;
297463 144 78 38;
297541 128 80 64;
297682 176 88 106;
297682 176 88 105;
297682 144 63 32;
297718 176 88 105;
297718 176 88 45;
297718 144 75 39;
297755 128 63 64;
297791 128 78 64;
297911 176 88 45;
297911 176 88 43;
297911 144 68 46;
298088 176 64 127;
298088 176 64 0;
298130 176 88 43;
298130 176 88 8;
298130 144 49 50;
298151 128 68 64;
298161 176 88 8;
298161 176 88 112;
298161 144 78 43;
298177 128 75 64;
298218 176 64 0;
298218 176 64 127;
298270 128 49 64;
298359 176 88 112;
298359 176 88 92;
298359 144 80 45;
298375 128 78 64;
298552 176 88 92;
298552 176 88 73;
298552 144 78 44;
298567 176 88 73;
298567 176 88 22;
298567 144 56 29;
298645 128 56 64;
298744 176 88 22;
298744 176 88 74;
298744 144 76 51;
298765 128 80 64;
298932 176 88 74;
298932 176 88 15;
298932 144 56 45;
298932 144 73 45;
298942 128 78 64;
298963 128 76 64;
298994 128 56 64;
299104 176 88 15;
299104 176 88 95;
299104 144 68 46;
299125 128 73 64;
299239 176 64 127;
299239 176 64 0;
299281 176 88 95;
299281 176 88 88;
299281 144 44 70;
299317 176 88 88;
299317 176 88 39;
299317 144 76 43;
299338 128 68 64;
299375 176 64 0;
299375 176 64 127;
299416 128 44 64;
299473 128 76 64;
299479 176 88 39;
299479 176 88 60;
299479 144 78 45;
299656 176 88 60;
299656 176 88 45;
299656 144 60 37;
299666 176 88 45;
299666 176 88 116;
299666 144 54 26;
299677 176 88 116;
299677 176 88 10;
299677 144 76 56;
299729 128 60 64;
299744 128 54 64;
299833 128 78 64;
299843 176 88 10;
299843 176 88 76;
299843 144 75 51;
299890 128 76 64;
300041 176 88 76;
300041 176 88 72;
300041 144 54 50;
300057 176 88 72;
300057 176 88 1;
300057 144 72 59;
300098 128 54 64;
300140 128 75 64;
300213 176 88 1;
300213 176 88 109;
300213 144 66 54;
300369 176 64 127;
300369 176 64 0;
300421 176 88 109;
300421 176 88 30;
300421 144 45 73;
300442 176 88 30;
300442 176 88 79;
300442 128 72 64;
300442 144 75 62;
300479 128 45 64;
300489 176 64 0;
300489 176 64 127;
300520 128 66 64;
300651 176 88 79;
300651 176 88 92;
300651 144 76 61;
300677 128 75 64;
300854 176 88 92;
300854 176 88 119;
300854 144 52 43;
300875 176 88 119;
300875 176 88 83;
300875 144 75 50;
300958 128 52 64;
301088 176 88 83;
301088 176 88 28;
301088 144 73 53;
301140 128 76 64;
301182 128 75 64;
301359 176 88 28;
301359 176 88 4;
301359 144 52 42;
301380 176 88 4;
301380 176 88 63;
301380 144 69 45;
301380 176 88 63;
301380 176 88 53;
301380 144 61 16;
301427 128 52 64;
301453 128 61 64;
301567 128 73 64;
301619 176 88 53;
301619 176 88 40;
301619 144 64 45;
301812 128 64 64;
301833 176 64 127;
301833 176 64 0;
301848 176 88 40;
301848 176 88 79;
301848 144 42 53;
301885 128 69 64;
301885 176 88 79;
301885 176 88 67;
301885 144 73 43;
301953 176 64 0;
301953 176 64 127;
301984 128 42 64;
302098 176 88 67;
302098 176 88 39;
302098 144 75 47;
302114 128 73 64;
302307 176 88 39;
302307 176 88 22;
302307 144 61 32;
302307 144 73 51;
302338 176 88 22;
302338 176 88 99;
302338 144 54 27;
302395 128 61 64;
302421 128 54 64;
302500 176 88 99;
302500 176 88 79;
302500 144 69 56;
302536 128 75 64;
302661 128 73 64;
302687 176 88 79;
302687 176 88 44;
302687 144 63 59;
302864 176 88 44;
302864 176 88 109;
302864 144 73 52;
302895 128 69 64;
302927 128 63 64;
302937 128 73 64;
303020 176 64 127;
303020 176 64 0;
303020 176 88 109;
303020 176 88 58;
303020 144 44 61;
303041 176 88 58;
303041 176 88 35;
303041 144 73 50;
303109 128 44 64;
303119 176 64 0;
303119 176 64 127;
303208 176 88 35;
303208 176 88 59;
303208 144 75 55;
303218 128 73 64;
303380 176 88 59;
303380 176 88 7;
303380 144 56 43;
303385 176 88 7;
303385 176 88 58;
303385 144 52 52;
303390 176 88 58;
303390 176 88 105;
303390 144 73 51;
303442 128 56 64;
303458 128 52 64;
303489 128 75 64;
303567 176 88 105;
303567 176 88 113;
303567 144 68 51;
303630 128 73 64;
303734 176 88 113;
303734 176 88 31;
303734 144 61 52;
303776 128 68 64;
303921 176 88 31;
303921 176 88 20;
303921 144 68 34;
303989 128 61 64;
303989 128 68 64;
304041 176 64 127;
304041 176 64 0;
304052 176 88 20;
304052 176 88 107;
304052 144 68 57;
304067 176 88 107;
304067 176 88 41;
304067 144 32 73;
304125 128 32 64;
304171 176 64 0;
304171 176 64 127;
304244 176 88 41;
304244 176 88 28;
304244 144 56 57;
304265 128 68 64;
304416 128 56 64;
304447 176 88 28;
304447 176 88 89;
304447 144 63 34;
304578 128 63 64;
304578 176 88 89;
304578 176 88 42;
304578 144 68 41;
304734 176 88 42;
304734 176 88 65;
304734 128 68 64;
304734 144 69 52;
304744 176 88 65;
304744 176 88 100;
304744 144 60 51;
304781 176 88 100;
304781 176 88 54;
304786 144 54 31;
304916 176 88 54;
304916 176 88 17;
304916 144 68 46;
304973 128 69 64;
305015 176 64 127;
305015 176 64 0;
305041 128 60 64;
305072 176 88 17;
305072 176 88 104;
305072 144 67 67;
305083 176 88 104;
305083 176 88 45;
305083 128 68 64;
305083 144 49 66;
305109 128 54 64;
305135 176 64 0;
305135 176 64 127;
305213 128 49 64;
305265 176 88 45;
305265 176 88 21;
305265 144 68 66;
305343 128 67 64;
305458 176 88 21;
305458 176 88 100;
305458 144 56 49;
305479 176 88 100;
305479 176 88 77;
305479 144 72 70;
305500 128 68 64;
305500 176 88 77;
305500 176 88 70;
305500 144 64 35;
305541 128 72 64;

305552 128 56 64;
305572 128 64 64;
305640 176 88 70;
305640 176 88 93;
305640 144 73 85;
305770 128 73 64;
305921 176 88 93;
305921 176 88 70;
305921 144 76 62;
305932 176 88 70;
305932 176 88 6;
305932 144 56 27;
306020 128 56 64;
306088 128 76 64;
306151 176 88 6;
306151 176 88 42;
306151 144 78 49;
306453 176 88 42;
306453 176 88 44;
306453 144 48 49;
306458 176 88 44;
306458 176 88 17;
306458 144 80 56;
306463 176 64 127;
306463 176 64 0;
306510 128 78 64;
306562 176 64 0;
306562 176 64 127;
306630 128 48 64;
306687 176 88 17;
306687 176 88 55;
306687 144 81 64;
306718 128 80 64;
306916 176 88 55;
306916 176 88 84;
306916 144 80 51;
306932 176 88 84;
306932 176 88 112;
306932 144 63 36;
306979 176 88 112;
306979 176 88 13;
306979 144 56 24;
307026 128 63 64;
307067 128 81 64;
307072 128 56 64;
307125 176 88 13;
307125 176 88 48;
307125 144 78 59;
307307 176 88 48;
307307 176 88 80;
307307 144 75 52;
307312 176 88 80;
307312 176 88 73;
307312 128 80 64;
307312 144 56 42;
307312 144 63 39;
307375 128 63 64;
307380 128 56 64;
307411 128 78 64;
307447 176 88 73;
307447 176 88 115;
307447 144 68 52;
307546 128 75 64;
307619 176 64 127;
307619 176 64 0;
307630 176 88 115;
307630 176 88 95;
307630 144 49 64;
307651 176 88 95;
307651 176 88 79;
307651 144 78 63;
307661 128 68 64;
307723 176 64 0;
307723 176 64 127;
307770 128 49 64;
307796 176 88 79;
307796 176 88 5;
307796 144 80 57;
307802 128 78 64;
307916 176 88 5;
307916 176 88 19;
307916 144 78 40;
307953 176 88 19;
307953 176 88 6;
307953 144 64 42;
307968 176 88 6;
307968 176 88 11;
307968 144 56 30;
308026 128 64 64;
308041 128 56 64;
308098 176 88 11;
308098 176 88 124;
308098 144 76 53;
308114 128 80 64;
308239 176 88 124;
308239 176 88 127;
308239 144 56 58;
308250 176 88 127;
308250 176 88 101;
308250 144 73 58;
308265 128 78 64;
308307 128 76 64;
308317 128 56 64;
308364 176 88 101;
308364 176 88 57;
308364 144 68 60;
308395 128 73 64;
308479 176 64 127;
308479 176 64 0;
308510 176 88 57;
308510 176 88 20;
308510 144 44 86;
308515 176 88 20;
308515 176 88 51;
308515 144 76 47;
308567 128 68 64;
308598 176 64 0;
308598 176 64 127;
308635 128 44 64;
308651 176 88 51;
308651 176 88 76;
308651 144 78 62;
308661 128 76 64;
308812 176 88 76;
308812 176 88 48;
308812 144 76 50;
308817 176 88 48;
308817 176 88 58;
308817 144 60 60;
308828 176 88 58;
308828 176 88 74;
308828 144 54 41;
308869 128 60 64;
308885 128 54 64;
308968 128 78 64;
308973 176 88 74;
308973 176 88 127;
308973 144 75 58;
309052 128 76 64;
309062 176 88 127;
309062 176 88 119;
309062 144 60 57;
309072 176 88 119;
309072 176 88 62;
309072 144 54 61;
309114 176 88 62;
309114 176 88 121;
309114 128 60 64;
309114 144 72 55;
309135 128 54 64;
309208 128 75 64;
309223 176 88 121;
309223 176 88 89;
309223 144 66 81;
309333 176 64 127;
309333 176 64 0;
309380 176 88 89;
309380 176 88 52;
309380 144 45 88;
309395 176 88 52;
309395 176 88 118;
309395 144 75 76;
309421 128 72 64;
309447 128 45 64;
309468 128 66 64;
309479 176 64 0;
309479 176 64 127;
309572 176 88 118;
309572 176 88 7;
309572 128 75 64;
309572 144 76 29;
309578 176 88 7;
309578 176 88 58;
309578 144 77 38;
309750 176 88 58;
309750 176 88 8;
309750 144 75 74;
309760 176 88 8;
309760 176 88 27;
309760 144 52 63;
309807 128 77 64;
309833 128 52 64;
309848 128 76 64;
309927 176 88 27;
309927 176 88 1;
309927 144 73 76;
310026 128 75 64;
310145 176 88 1;
310145 176 88 96;
310145 144 69 66;
310151 176 88 96;
310151 176 88 68;
310151 144 52 53;
310151 144 61 45;
310213 128 61 64;
310218 128 52 64;
310223 128 73 64;
310390 176 88 68;
310390 176 88 121;
310390 144 64 77;
310395 128 69 64;
310625 176 88 121;
310625 176 88 13;
310625 144 42 65;
310630 176 64 127;
310630 176 64 0;
310656 176 88 13;
310656 176 88 20;
310656 144 69 67;
310723 176 64 0;
310723 176 64 127;
310760 128 64 64;
310770 128 42 64;
310822 176 88 20;
310822 176 88 73;
310822 144 71 55;
310942 128 69 64;
310979 176 88 73;
310979 176 88 108;
310979 144 73 62;
311015 128 71 64;
311140 128 73 64;
311151 176 88 108;
311151 176 88 57;
311151 144 74 74;
311260 176 88 57;
311260 176 88 11;
311260 144 57 41;
311281 176 88 11;
311281 176 88 53;
311281 144 62 42;
311296 176 88 53;
311296 176 88 4;
311296 144 69 38;
311328 176 64 127;
311328 176 64 0;
311333 176 88 4;
311333 176 88 60;
311333 144 76 68;
311343 128 74 64;
311390 176 64 0;
311390 176 64 127;
311505 176 88 60;
311505 176 88 27;
311505 144 78 61;
311515 128 76 64;
311645 128 78 64;
311656 176 88 27;
311656 176 88 8;
311656 144 79 77;
311786 176 88 8;
311786 176 88 104;
311786 144 80 65;
311802 128 79 64;
311864 128 80 64;
311901 176 88 104;
311901 176 88 62;
311901 144 81 85;
311911 128 69 64;
311989 128 62 64;
312005 128 57 64;
312041 176 88 62;
312041 176 88 116;
312041 144 83 69;
312057 128 81 64;
312187 176 88 116;
312187 176 88 63;
312187 128 83 64;
312187 144 85 49;
312286 128 85 64;
312307 176 88 63;
312307 176 88 8;
312307 144 86 77;
312411 176 64 127;
312411 176 64 0;
312437 176 88 8;
312437 176 88 49;
312437 144 44 62;
312473 176 64 0;
312473 176 64 127;
312479 176 88 49;
312479 176 88 125;
312479 144 87 70;
312515 128 44 64;
312520 128 86 64;
312656 128 87 64;
312666 176 88 125;
312666 176 88 71;
312666 144 88 69;
312854 176 88 71;
312854 176 88 115;
312854 144 66 41;
312859 176 88 115;
312859 176 88 40;
312859 144 56 33;
312864 176 88 40;
312864 176 88 99;
312864 144 89 75;
312895 128 88 64;
312901 176 88 99;
312901 176 88 100;
312901 144 60 32;
312927 128 66 64;
312937 128 56 64;
312973 128 60 64;
313052 176 88 100;
313052 176 88 65;
313052 144 90 60;
313072 128 89 64;
313239 176 88 65;
313239 176 88 72;
313239 144 66 50;
313244 176 88 72;
313244 176 88 63;
313244 144 56 39;
313255 176 88 63;
313255 176 88 94;
313255 144 91 73;
313276 128 90 64;
313302 128 66 64;
313317 128 56 64;
313458 176 88 94;
313458 176 88 34;
313458 144 92 55;
313473 128 91 64;
313708 176 64 127;
313708 176 64 0;
313713 128 92 64;
313760 176 88 34;
313760 176 88 5;
313760 144 49 57;
313776 176 88 5;
313776 176 88 56;
313776 144 97 50;
313822 176 64 0;
313822 176 64 127;
313838 128 49 64;
314359 176 88 56;
314359 176 88 8;
314359 144 61 42;
314385 176 88 8;
314385 176 88 85;

314385 144 56 25;
314421 128 61 64;
314468 128 56 64;
314536 128 97 64;
315911 176 64 127;
315911 176 64 0;
473630 176 64 0;
473630 176 64 127;
473942 176 64 127;
473942 176 64 0;
482041 176 64 0;
482041 176 64 127;
484671 176 64 127;
484671 176 64 0;
485942 176 64 0;
485942 176 64 127;
485963 176 88 85;
485963 176 88 98;
485963 144 45 36;
485973 176 88 98;
485973 176 88 90;
485973 144 69 36;
486010 176 88 90;
486010 176 88 2;
486010 144 72 33;
486255 128 45 64;
486494 128 69 64;
486817 176 88 2;
486817 176 88 92;
486817 144 52 27;
486916 128 72 64;
487156 176 88 92;
487156 176 88 78;
487156 144 57 32;
487359 128 52 64;
487427 176 88 78;
487427 176 88 28;
487427 144 76 49;
487703 176 88 28;
487703 176 88 65;
487703 144 69 48;
487708 128 57 64;
487744 128 76 64;
487979 176 88 65;
487979 176 88 103;
487979 144 81 89;
488109 128 69 64;
488234 176 88 103;
488234 176 88 29;
488234 144 69 49;
488281 176 88 29;
488281 176 88 91;
488281 144 57 31;
488364 128 57 64;
488536 176 88 91;
488536 176 88 32;
488536 144 79 72;
488541 176 88 32;
488541 176 88 120;
488541 144 45 52;
488557 176 64 127;
488557 176 64 0;
488562 128 81 64;
488609 176 64 0;
488609 176 64 127;
488854 176 88 120;
488854 176 88 48;
488854 144 52 36;
488942 128 52 64;
489125 176 88 48;
489125 176 88 118;
489125 144 57 50;
489437 176 88 118;
489437 176 88 92;
489437 144 77 54;
489473 128 69 64;
489500 128 79 64;
489526 128 57 64;
489838 176 88 92;
489838 176 88 26;
489838 144 57 28;
489921 128 45 64;
490171 128 57 64;
490208 176 88 26;
490208 176 88 65;
490208 144 76 53;
490229 128 77 64;
490713 128 76 64;
490776 176 88 65;
490776 176 88 22;
490776 144 77 29;
490901 128 77 64;
490927 176 88 22;
490927 176 88 60;
490927 144 76 43;
491000 128 76 64;
491041 176 88 60;
491041 176 88 33;
491041 144 77 31;
491125 128 77 64;
491140 176 88 33;
491140 176 88 124;
491140 144 76 53;
491177 176 88 124;
491177 176 88 9;
491177 144 50 52;
491218 176 88 9;
491218 176 88 1;
491218 144 77 25;
491281 128 77 64;
491484 176 88 1;
491484 176 88 11;
491484 144 74 60;
491494 128 76 64;
491604 128 50 64;
491708 176 88 11;
491708 176 88 93;
491708 144 57 50;
491781 128 57 64;
491786 176 88 93;
491786 176 88 81;
491786 144 72 63;
491843 128 74 64;
492015 176 88 81;
492015 176 88 4;
492015 144 71 84;
492015 176 88 4;
492015 176 88 93;
492015 144 62 57;
492067 128 72 64;
492171 128 62 64;
492208 176 64 127;
492208 176 64 0;
492270 176 88 93;
492270 176 88 39;
492270 144 69 77;
492281 128 71 64;
492354 176 64 0;
492354 176 64 127;
492526 176 88 39;
492526 176 88 37;
492526 144 71 89;
492557 176 88 37;
492557 176 88 5;
492557 144 57 49;
492572 128 69 64;
492625 128 57 64;
492796 176 88 5;
492796 176 88 116;
492796 144 72 82;
492812 128 71 64;
493041 128 72 64;
493057 176 88 116;
493057 176 88 118;
493057 144 74 81;
493062 176 88 118;
493062 176 88 27;
493062 144 62 34;
493151 128 62 64;
493364 176 88 27;
493364 176 88 81;
493364 144 76 74;
493380 176 88 81;
493380 176 88 111;
493380 144 50 45;
493411 128 74 64;
493432 176 64 127;
493432 176 64 0;
493526 176 64 0;
493526 176 64 127;
493677 176 88 111;
493677 176 88 121;
493677 144 57 46;
493765 128 57 64;
494046 176 88 121;
494046 176 88 96;
494046 144 62 32;
494151 128 62 64;
494348 176 88 96;
494348 176 88 16;
494348 144 74 35;
494739 128 50 64;
494765 176 88 16;
494765 176 88 59;
494765 128 74 64;
494765 144 81 36;
494770 128 76 64;
495057 128 81 64;
495088 176 88 59;
495088 176 88 16;
495088 144 74 53;
495609 176 88 16;
495609 176 88 105;
495609 144 84 35;
496031 128 84 64;
496052 176 88 105;
496052 176 88 45;
496052 144 83 50;
496260 128 74 64;
496463 176 88 45;
496463 176 88 127;
496463 144 80 50;
496494 176 88 127;
496494 176 88 5;
496494 144 52 52;
496541 128 83 64;
496598 176 64 127;
496598 176 64 0;
496687 176 64 0;
496687 176 64 127;
496802 176 88 5;
496802 176 88 54;
496802 144 56 33;
497010 128 56 64;
497057 176 88 54;
497057 176 88 37;
497057 144 59 57;
497125 128 59 64;
497296 176 88 37;
497296 176 88 60;
497296 144 76 68;
497302 176 88 60;
497302 176 88 37;
497302 144 64 54;
497307 128 80 64;
497479 176 88 37;
497479 176 88 99;
497479 128 64 64;
497479 144 64 14;
497562 176 88 99;
497562 176 88 111;
497562 144 88 78;
497666 128 64 64;
497755 128 76 64;
497822 176 88 111;
497822 176 88 46;
497822 144 76 69;
497989 128 88 64;
498083 176 88 46;
498083 176 88 94;
498083 144 86 75;
498093 176 88 94;
498093 176 88 11;
498093 144 64 41;
498166 128 76 64;
498197 128 64 64;
498338 176 88 11;
498338 176 88 53;
498338 144 76 38;
498588 176 88 53;
498588 176 88 8;
498588 144 84 64;
498656 128 76 64;
498760 128 86 64;
498812 176 88 8;
498812 176 88 5;
498812 144 76 47;
499093 176 88 5;
499093 176 88 91;
499093 144 83 42;
499104 128 76 64;
499114 128 84 64;
499291 128 83 64;
499322 176 88 91;
499322 176 88 25;
499322 144 76 40;
499619 176 88 25;
499619 176 88 127;
499619 144 81 45;
499656 128 76 64;
499916 176 88 127;
499916 176 88 97;
499916 144 83 49;
499984 128 81 64;
500052 128 52 64;
500229 128 83 64;
500244 144 84 37;
500526 128 84 64;
500541 176 88 97;
500541 176 88 19;
500541 144 81 39;
500796 128 81 64;
500838 176 88 19;
500838 176 88 85;
500838 144 84 41;
500875 176 88 85;
500875 176 88 29;
500875 144 45 25;
501088 176 88 29;
501088 176 88 73;
501088 144 81 36;
501098 176 88 73;
501098 176 88 1;
501098 144 52 37;
501125 128 84 64;
501177 128 52 64;
501395 176 88 1;
501395 176 88 36;
501395 144 57 39;
501468 128 81 64;
501484 128 57 64;
501630 176 88 36;
501630 176 88 117;
501630 144 72 40;
501854 128 72 64;
501895 176 88 117;
501895 176 88 98;
501895 144 71 41;
502151 176 88 98;
502151 176 88 43;
502151 144 69 45;
502171 128 71 64;
502333 128 45 64;
502354 128 69 64;
502411 176 88 43;
502411 176 88 72;
502411 144 64 38;
502442 176 88 72;
502442 176 88 58;
502442 144 57 17;
502515 128 57 64;
502562 128 64 64;
502661 176 88 58;
502661 176 88 99;
502661 144 62 30;
502708 176 88 99;
502708 176 88 52;
502708 144 45 24;
502916 176 88 52;
502916 176 88 119;
502916 144 60 47;
502947 176 64 127;
502947 176 64 0;
502947 128 62 64;
503156 176 88 119;
503156 176 88 71;
503156 144 59 44;
503197 128 60 64;
503380 128 59 64;
503390 176 88 71;
503390 176 88 20;
503390 144 57 50;

503442 176 64 0;
503442 176 64 127;
503630 128 57 64;
503635 176 88 20;
503635 176 88 28;
503635 144 59 47;
503880 128 59 64;
503890 176 88 28;
503890 176 88 52;
503890 144 60 52;
503953 176 88 52;
503953 176 88 79;
503953 144 52 23;
503958 128 60 64;
504072 128 52 64;
504166 176 88 79;
504166 176 88 10;
504166 144 64 37;
504500 176 88 10;
504500 176 88 119;
504500 144 69 31;
504598 128 45 64;
504750 128 64 64;
504854 128 69 64;
504854 176 88 119;
504854 176 88 108;
504854 144 72 30;
505010 128 72 64;
505109 176 88 108;
505109 176 88 63;
505109 144 64 24;
505244 128 64 64;
505291 176 88 63;
505291 176 88 27;
505291 144 45 63;
505317 176 88 27;
505317 176 88 11;
505317 144 72 57;
505322 176 88 11;
505322 176 88 39;
505322 144 69 52;
505328 176 88 39;
505328 176 88 108;
505328 144 64 26;
505421 128 64 64;
505625 176 88 108;
505625 176 88 42;
505625 144 64 42;
505640 128 69 64;
505661 128 72 64;
505895 176 88 42;
505895 176 88 91;
505895 144 69 44;
506036 128 64 64;
506166 176 88 91;
506166 176 88 34;
506166 144 72 42;
506192 128 45 64;
506260 128 69 64;
506390 176 88 34;
506390 176 88 88;
506390 144 45 56;
506390 144 69 48;
506390 176 88 88;
506390 176 88 91;
506390 128 72 64;
506390 144 72 25;
506682 176 88 91;
506682 176 88 11;
506682 144 64 41;
506739 128 69 64;
506885 128 72 64;
506942 176 88 11;
506942 176 88 20;
506942 144 69 67;
507072 128 45 64;
507192 176 88 20;
507192 176 88 98;
507192 144 72 80;
507244 128 64 64;
507281 128 69 64;
507364 128 72 64;
507437 176 88 98;
507437 176 88 87;
507437 144 72 67;
507468 176 88 87;
507468 176 88 127;
507468 144 69 54;
507473 176 88 127;
507473 176 88 81;
507473 144 41 53;
507531 176 64 127;
507531 176 64 0;
507593 176 64 0;
507593 176 64 127;
507739 176 88 81;
507739 176 88 19;
507739 144 65 41;
507770 128 69 64;
508020 176 88 19;
508020 176 88 22;
508020 144 69 75;
508083 128 72 64;
508302 176 88 22;
508302 176 88 31;
508302 144 72 65;
508312 176 88 31;
508312 176 88 62;
508312 128 65 64;
508312 144 65 16;
508328 128 41 64;
508406 128 65 64;
508468 128 72 64;
508494 128 69 64;
508557 176 88 62;
508557 176 88 83;
508557 144 72 65;
508588 176 88 83;
508588 176 88 47;
508588 144 41 48;
508880 176 88 47;
508880 176 88 54;
508880 144 65 41;
509088 128 41 64;
509234 176 88 54;
509234 176 88 10;
509234 144 69 40;
509276 128 72 64;
509593 176 88 10;
509593 176 88 41;
509593 144 72 48;
509630 128 69 64;
509656 128 65 64;
509901 128 72 64;
509901 176 88 41;
509901 176 88 124;
509901 144 74 57;
509932 176 88 124;
509932 176 88 68;
509932 144 38 37;
509968 176 64 127;
509968 176 64 0;
510005 176 64 0;
510005 176 64 127;
510151 176 88 68;
510151 176 88 40;
510151 144 45 52;
510182 176 88 40;
510182 176 88 107;
510182 144 72 45;
510218 128 45 64;
510416 128 74 64;
510427 176 88 107;
510427 176 88 119;
510427 144 71 57;
510614 128 72 64;
510682 176 88 119;
510682 176 88 122;
510682 144 65 77;
510692 176 88 122;
510692 176 88 39;
510692 144 50 36;
510703 128 71 64;
510953 176 88 39;
510953 176 88 33;
510953 144 77 83;
510963 128 50 64;
511197 176 88 33;
511197 176 88 80;
511197 144 76 67;
511442 176 88 80;
511442 176 88 72;
511442 144 50 40;
511453 176 88 72;
511453 176 88 120;
511453 128 38 64;
511453 144 74 77;
511468 128 77 64;
511546 128 50 64;
511828 128 74 64;
511864 128 76 64;
512026 176 88 120;
512026 176 88 118;
512026 144 40 45;
512083 176 64 127;
512083 176 64 0;
512104 176 64 0;
512104 176 64 127;
512296 176 88 118;
512296 176 88 16;
512296 144 47 32;
512307 128 65 64;
512375 128 47 64;
512635 176 88 16;
512635 176 88 14;
512635 144 52 34;
512927 176 88 14;
512927 176 88 7;
512927 144 74 51;
513010 128 74 64;
513067 128 52 64;
513260 176 88 7;
513260 176 88 51;
513260 144 80 48;
513270 176 88 51;
513270 176 88 108;
513270 144 76 48;
513276 176 88 108;
513276 176 88 13;
513276 144 74 40;
513359 128 74 64;
513520 176 88 13;
513520 176 88 75;
513520 144 74 73;
513552 176 88 75;
513552 176 88 14;
513552 144 52 34;
513812 128 76 64;
513828 128 52 64;
513833 128 80 64;
513880 176 88 14;
513880 176 88 105;
513880 144 83 85;
513911 128 40 64;
514109 128 74 64;
514255 176 88 105;
514255 176 88 97;
514255 144 81 77;
514406 128 83 64;
514473 128 81 64;
514614 176 88 97;
514614 176 88 35;
514614 144 81 67;
514625 176 88 35;
514625 176 88 14;
514625 144 45 52;
514661 176 64 127;
514661 176 64 0;
514734 176 64 0;
514734 176 64 127;
514880 176 88 14;
514880 176 88 31;
514880 144 52 44;
514947 128 52 64;
515119 176 88 31;
515119 176 88 53;
515119 144 57 56;
515369 176 88 53;
515369 176 88 89;
515369 144 76 59;
515390 128 81 64;
515598 128 57 64;
515661 176 88 89;
515661 176 88 15;
515661 144 88 81;
515885 176 88 15;
515885 176 88 82;
515885 144 57 35;
515916 176 88 82;
515916 176 88 3;
515916 144 86 61;
515973 128 57 64;
516223 176 88 3;
516223 176 88 74;
516223 144 84 34;
516270 128 88 64;
516442 128 45 64;
516489 176 88 74;
516489 176 88 26;
516489 144 57 28;
516500 176 88 26;
516500 176 88 120;
516500 144 83 55;
516546 128 86 64;
516546 128 84 64;
516593 128 57 64;
516880 128 83 64;
516906 176 88 120;
516906 176 88 8;
516906 144 41 55;
516906 144 84 64;
516979 176 64 127;
516979 176 64 0;
517000 176 64 0;
517000 176 64 127;
517098 176 88 8;
517098 176 88 84;
517098 128 41 64;
517098 144 41 11;
517140 128 76 64;
517161 176 88 84;
517161 176 88 15;
517161 144 48 38;
517270 128 48 64;
517458 176 88 15;
517458 176 88 99;
517458 144 53 66;
517718 176 88 99;
517718 176 88 63;
517718 144 77 67;
517791 128 84 64;
518026 176 88 63;
518026 176 88 19;
518026 144 88 56;
518052 128 53 64;
518296 176 88 19;
518296 176 88 8;
518296 128 88 64;
518296 144 86 77;
518338 176 88 8;
518338 176 88 58;
518338 144 53 34;
518421 128 53 64;
518588 176 88 58;
518588 176 88 113;
518588 144 84 74;
518593 128 41 64;
518609 176 88 113;
518609 176 88 23;
518609 144 48 48;
518645 128 86 64;
518692 128 48 64;
518901 176 88 23;
518901 176 88 62;
518901 144 83 78;
518911 176 88 62;
518911 176 88 17;
518911 144 53 42;
518942 128 84 64;
518984 128 53 64;
519213 176 88 17;
519213 176 88 89;
519213 144 84 91;
519244 128 83 64;
519385 176 88 89;
519385 176 88 15;
519385 144 38 42;
519401 176 88 15;
519401 176 88 47;
519401 144 36 40;
519401 176 64 127;
519401 176 64 0;

519473 128 36 64;
519546 176 64 0;
519546 176 64 127;
519640 176 88 47;
519640 176 88 100;
519640 144 45 60;
519708 128 77 64;
519713 128 45 64;
519927 176 88 100;
519927 176 88 52;
519927 144 50 68;
520218 176 88 52;
520218 176 88 24;
520218 144 81 70;
520515 176 88 24;
520515 176 88 104;
520515 144 89 94;
520526 128 50 64;
520552 128 84 64;
520744 128 81 64;
520812 176 88 104;
520812 176 88 90;
520812 144 81 72;
520822 176 88 90;
520822 176 88 101;
520822 144 50 22;
520968 128 50 64;
521145 176 88 101;
521145 176 88 10;
521145 144 88 91;
521171 128 89 64;
521380 128 81 64;
521453 176 88 10;
521453 176 88 63;
521453 144 81 68;
521484 128 38 64;
521494 176 88 63;
521494 176 88 23;
521494 144 50 37;
521838 128 50 64;
521869 176 88 23;
521869 176 88 49;
521869 144 86 67;
521875 176 88 49;
521875 176 88 75;
521875 144 40 49;
521927 128 81 64;
522062 128 88 64;
522197 176 88 75;
522197 176 88 27;
522197 144 47 32;
522302 128 47 64;
522505 176 88 27;
522505 176 88 92;
522505 144 52 54;
522515 128 86 64;
522869 176 88 92;
522869 176 88 26;
522869 144 74 39;
522869 144 76 45;
522869 144 83 39;
522869 176 88 26;
522869 176 88 123;
522869 144 80 30;
522932 128 76 64;
522937 128 74 64;
522947 128 80 64;
522973 128 83 64;
523026 128 52 64;
523260 176 88 123;
523260 176 88 22;
523260 144 83 51;
523265 176 88 22;
523265 176 88 33;
523265 144 80 40;
523286 176 88 33;
523286 176 88 25;
523286 144 76 29;
523291 176 88 25;
523291 176 88 12;
523291 144 74 28;
523416 128 74 64;
523427 128 76 64;
523588 176 88 12;
523588 176 88 83;
523588 144 52 33;
523677 128 80 64;
523692 176 88 83;
523692 176 88 89;
523692 144 74 47;
523708 128 83 64;
523973 128 52 64;
524046 176 88 89;
524046 176 88 96;
524046 144 76 47;
524057 128 74 64;
524338 128 76 64;
524348 176 88 96;
524348 176 88 112;
524348 144 74 42;
524416 176 88 112;
524416 176 88 95;
524416 144 52 16;
524572 128 52 64;
524661 176 88 95;
524661 176 88 43;
524661 144 80 45;
524687 128 74 64;
524979 176 88 43;
524979 176 88 98;
524979 144 74 36;
525000 128 80 64;
525005 176 88 98;
525005 176 88 94;
525005 144 52 21;
525171 128 52 64;
525307 176 88 94;
525307 176 88 71;
525307 144 83 42;
525359 128 74 64;
525640 128 83 64;
525677 176 88 71;
525677 176 88 107;
525677 144 74 45;
525760 128 74 64;
526010 176 88 107;
526010 176 88 85;
526010 144 71 73;
526046 176 88 85;
526046 176 88 95;
526046 144 52 23;
526119 128 71 64;
526130 128 52 64;
526359 176 88 95;
526359 176 88 85;
526359 144 68 62;
526416 128 68 64;
526744 176 88 85;
526744 176 88 120;
526744 144 64 52;
526786 176 88 120;
526786 176 88 56;
526786 144 52 22;
526817 128 64 64;
526901 128 52 64;
527062 176 88 56;
527062 176 88 93;
527062 144 62 57;
527125 128 62 64;
527354 176 88 93;
527354 176 88 107;
527354 144 59 52;
527416 128 59 64;
527604 176 88 107;
527604 176 88 58;
527604 144 56 41;
527703 128 56 64;
527859 176 88 58;
527859 176 88 97;
527859 144 52 48;
528041 128 40 64;
528114 176 88 97;
528114 176 88 83;
528114 128 52 64;
528114 144 56 39;
528364 128 56 64;
528395 176 88 83;
528395 176 88 29;
528395 144 52 37;
528411 176 88 29;
528411 176 88 47;
528411 144 40 41;
528682 176 88 47;
528682 176 88 89;
528682 144 59 59;
528729 128 52 64;
528911 128 59 64;
528963 176 88 89;
528963 176 88 51;
528963 144 52 44;
528979 176 88 51;
528979 176 88 116;
528979 144 47 30;
529057 128 47 64;
529265 176 88 116;
529265 176 88 101;
529265 144 60 49;
529296 128 52 64;
529494 128 60 64;
529572 176 88 101;
529572 176 88 33;
529572 144 52 50;
529750 176 88 33;
529750 176 88 116;
529750 128 52 64;
529750 144 52 9;
529901 176 88 116;
529901 176 88 86;
529901 144 62 51;
529942 128 52 64;
530109 176 64 127;
530109 176 64 0;
530192 176 64 0;
530192 176 64 127;
530276 176 88 86;
530276 176 88 14;
530276 144 52 39;
530651 128 62 64;
530656 176 88 14;
530656 176 88 0;
530656 144 60 41;
530817 128 52 64;
531005 176 88 0;
531005 176 88 101;
531005 144 47 31;
531083 128 47 64;
531442 176 88 101;
531442 176 88 27;
531442 144 52 27;
531791 128 52 64;
531833 128 60 64;
531838 176 88 27;
531838 176 88 68;
531838 144 59 38;
532005 128 40 64;
532213 176 88 68;
532213 176 88 18;
532213 144 47 30;
532296 128 47 64;
532531 128 59 64;
534005 176 88 18;
534005 176 88 116;
534005 144 64 27;
534015 176 88 116;
534015 176 88 77;
534015 144 60 32;
534026 176 88 77;
534026 176 88 105;
534026 144 33 30;
534031 176 88 105;
534031 176 88 71;
534031 144 57 22;
534322 128 64 64;
534322 128 57 64;
534380 176 88 71;
534380 176 88 44;
534380 144 45 35;
534656 128 60 64;
534687 176 88 44;
534687 176 88 19;
534687 144 57 48;
534958 176 88 19;
534958 176 88 101;
534958 128 57 64;
534958 144 57 2;
534973 176 88 101;
534973 176 88 119;
534973 144 60 65;
535255 176 88 119;
535255 176 88 18;
535255 144 64 68;
535270 128 45 64;
535385 128 57 64;
535520 176 88 18;
535520 176 88 111;
535520 144 45 37;
535791 128 45 64;
535796 128 64 64;
535812 176 88 111;
535812 176 88 64;
535812 144 57 41;
535854 128 60 64;
535854 128 33 64;
536072 176 88 64;
536072 176 88 65;
536072 144 45 48;
536354 176 88 65;
536354 176 88 82;
536354 144 64 76;
536359 176 88 82;
536359 176 88 1;
536359 144 61 66;
536364 176 88 1;
536364 176 88 125;
536364 128 45 64;
536364 144 33 59;
536385 176 64 127;
536385 176 64 0;
536447 176 64 0;
536447 176 64 127;
536645 176 88 125;
536645 176 88 123;
536645 144 45 49;
536687 128 57 64;
536911 176 88 123;
536911 176 88 40;
536911 144 57 51;
536968 128 61 64;
537125 128 64 64;
537182 176 88 40;
537182 176 88 47;
537182 144 61 63;
537468 176 88 47;
537468 176 88 61;
537468 144 64 91;
537536 128 45 64;
537557 128 33 64;
537750 176 88 61;
537750 176 88 0;
537750 144 45 31;
537760 128 57 64;
537880 128 45 64;
538005 128 64 64;
538026 176 88 0;
538026 176 88 80;
538026 144 57 57;
538130 128 61 64;
538317 176 88 80;
538317 176 88 120;
538317 144 45 25;
538489 128 45 64;
538635 176 88 120;
538635 176 88 44;
538635 144 62 73;
538640 176 88 44;
538640 176 88 40;
538640 144 38 51;
538651 176 64 127;
538651 176 64 0;
538713 176 64 0;
538713 176 64 127;
539187 176 88 40;
539187 176 88 51;
539187 144 50 55;
539239 128 57 64;
539442 176 88 51;
539442 176 88 101;
539442 144 57 54;
539750 176 88 101;
539750 176 88 90;
539750 144 65 88;
539755 176 88 90;

539755 176 88 50;
539755 128 62 64;
539755 144 62 27;
539822 128 50 64;
540005 176 88 50;
540005 176 88 22;
540005 144 50 31;
540036 128 57 64;
540109 128 50 64;
540229 128 65 64;
540307 176 88 22;
540307 176 88 100;
540307 144 57 39;
540390 128 62 64;
540536 128 38 64;
540562 176 88 100;
540562 176 88 107;
540562 144 50 22;
540838 128 50 64;
540875 176 88 107;
540875 176 88 80;
540875 144 69 46;
540890 176 88 80;
540890 176 88 122;
540890 144 38 36;
540973 128 57 64;
541500 176 88 122;
541500 176 88 104;
541500 144 50 24;
541786 128 69 64;
542130 128 50 64;
542140 176 88 104;
542140 176 88 63;
542140 144 69 38;
542177 176 88 63;
542177 176 88 76;
542177 144 62 23;
542187 176 88 76;
542187 176 88 74;
542187 144 65 26;
542218 128 38 64;
542817 128 62 64;
542911 176 88 74;
542911 176 88 73;
542911 144 50 27;
542921 128 65 64;
543020 128 69 64;
543437 128 50 64;
543447 176 88 73;
543447 176 88 78;
543447 144 69 25;
543468 176 88 78;
543468 176 88 11;
543468 144 65 22;
543572 128 65 64;
545026 176 88 11;
545026 176 88 108;
545026 144 40 42;
545036 176 88 108;
545036 176 88 47;
545036 144 68 37;
545041 128 69 64;
545406 176 88 47;
545406 176 88 23;
545406 144 52 43;
545713 176 88 23;
545713 176 88 64;
545713 144 62 37;
545729 176 88 64;
545729 176 88 46;
545729 144 64 29;
545833 128 68 64;
545963 176 88 46;
545963 176 88 35;
545963 144 68 48;
546255 176 88 35;
546255 176 88 105;
546255 144 71 72;
546312 128 52 64;
546369 128 40 64;
546453 128 64 64;
546458 128 62 64;
546510 176 88 105;
546510 176 88 44;
546510 144 52 45;
546536 176 88 44;
546536 176 88 0;
546536 144 40 21;
546562 176 88 0;
546562 176 88 114;
546562 144 62 49;
546567 176 88 114;
546567 176 88 1;
546567 144 64 45;
546609 128 68 64;
546822 176 88 1;
546822 176 88 88;
546822 144 68 71;
546869 128 71 64;
546895 128 52 64;
547062 128 40 64;
547104 176 88 88;
547104 176 88 107;
547104 144 71 69;
547213 128 64 64;
547218 128 62 64;
547390 176 88 107;
547390 176 88 53;
547390 144 62 57;
547390 176 88 53;
547390 176 88 93;
547390 144 40 44;
547390 144 64 50;
547406 128 68 64;
547651 128 71 64;
547661 176 88 93;
547661 176 88 113;
547661 144 68 40;
547973 176 88 113;
547973 176 88 116;
547973 144 71 51;
548031 128 62 64;
548031 128 64 64;
548239 128 71 64;
548296 128 68 64;
548312 176 88 116;
548312 176 88 36;
548312 144 62 27;
548354 176 88 36;
548354 176 88 21;
548354 144 64 17;
548635 176 88 21;
548635 176 88 29;
548635 144 74 48;
548682 176 88 29;
548682 176 88 73;
548682 144 52 30;
548781 128 62 64;
548786 128 64 64;
548947 128 52 64;
549015 176 88 73;
549015 176 88 55;
549015 144 62 24;
549031 176 88 55;
549031 176 88 39;
549031 144 64 20;
549171 128 74 64;
549369 176 88 39;
549369 176 88 67;
549369 144 72 37;
549583 128 64 64;
549604 128 40 64;
549614 128 62 64;
549760 176 88 67;
549760 176 88 112;
549760 144 52 23;
549864 128 52 64;
550255 128 72 64;
550317 176 88 112;
550317 176 88 5;
550317 144 64 36;
550427 176 88 5;
550427 176 88 120;
550427 144 72 53;
550437 176 88 120;
550437 176 88 7;
550437 144 67 30;
550442 176 88 7;
550442 176 88 122;
550442 144 36 33;
550447 176 64 127;
550447 176 64 0;
550500 176 64 0;
550500 176 64 127;
550625 128 64 64;
550760 176 88 122;
550760 176 88 60;
550760 144 48 44;
550843 128 48 64;
551031 128 72 64;
551036 176 88 60;
551036 176 88 108;
551036 144 64 64;
551057 128 67 64;
551338 176 88 108;
551338 176 88 67;
551338 144 76 79;
551656 176 88 67;
551656 176 88 28;
551656 144 48 41;
551677 128 36 64;
551677 144 36 13;
551838 128 64 64;
551958 176 88 28;
551958 176 88 52;
551963 144 64 49;
552166 128 76 64;
552322 176 88 52;
552322 176 88 107;
552322 144 72 62;
552333 128 36 64;
552348 128 48 64;
552473 128 64 64;
552791 176 88 107;
552791 176 88 58;
552791 144 48 28;
552906 128 48 64;
553036 176 88 58;
553036 176 88 22;
553036 144 43 55;
553057 176 88 22;
553057 176 88 0;
553057 144 71 57;
553104 176 64 127;
553104 176 64 0;
553166 128 72 64;
553270 176 64 0;
553270 176 64 127;
553359 176 88 0;
553359 176 88 52;
553359 144 50 42;
553427 128 50 64;
553640 176 88 52;
553640 176 88 6;
553640 144 55 48;
553947 176 88 6;
553947 176 88 9;
553947 144 62 54;
554005 128 71 64;
554020 128 62 64;
554281 176 88 9;
554281 176 88 25;
554281 144 67 66;
554401 128 67 64;
554463 128 55 64;
554572 176 88 25;
554572 176 88 74;
554572 144 69 95;
554854 176 88 74;
554854 176 88 78;
554854 144 71 83;
554895 128 69 64;
555057 128 43 64;
555130 176 88 78;
555130 176 88 25;
555130 144 67 81;
555135 176 88 25;
555135 176 88 105;
555135 144 55 36;
555177 128 71 64;
555223 128 55 64;
555442 176 88 105;
555442 176 88 65;
555442 144 72 88;
555458 176 64 127;
555458 176 64 0;
555458 176 88 65;
555458 176 88 57;
555458 144 48 71;
555494 128 67 64;
555572 176 64 0;
555572 176 64 127;
555718 176 88 57;
555718 176 88 18;
555718 144 55 49;
555786 128 55 64;
556000 176 88 18;
556000 176 88 28;
556000 144 60 64;
556015 176 88 28;
556015 176 88 117;
556015 144 67 79;
556057 128 72 64;
556312 176 88 117;
556312 176 88 104;
556312 144 76 85;
556322 176 88 104;
556322 176 88 61;
556322 144 72 82;
556359 128 60 64;
556614 176 88 61;
556614 176 88 68;
556614 144 60 35;
556630 128 67 64;
556802 128 60 64;
556875 128 76 64;
556901 176 88 68;
556901 176 88 126;
556901 144 67 51;
556963 128 72 64;
557203 176 88 126;
557203 176 88 15;
557203 144 76 83;
557218 176 88 15;
557218 176 88 119;
557218 144 72 68;
557250 128 48 64;
557380 128 67 64;
557432 128 76 64;
557484 176 88 119;
557484 176 88 20;
557484 144 74 80;
557484 176 88 20;
557484 176 88 126;
557484 144 67 64;
557505 176 88 126;
557505 176 88 119;
557505 144 55 29;
557515 128 72 64;
557583 128 55 64;
557760 128 67 64;
557807 176 88 119;
557807 176 88 126;
557807 144 71 94;
557812 176 88 126;
557812 176 88 25;
557812 144 43 70;
557817 176 64 127;
557817 176 64 0;
557864 128 74 64;
557927 176 64 0;
557927 176 64 127;
558098 176 88 25;
558098 176 88 54;
558098 144 50 47;
558156 128 50 64;
558406 176 88 54;
558406 176 88 48;
558406 144 55 53;
558718 176 88 48;
558718 176 88 90;
558718 144 62 49;
558770 128 71 64;
558807 128 62 64;
559000 176 88 90;
559000 176 88 58;
559000 144 67 50;
559062 128 67 64;
559078 176 88 58;
559078 176 88 110;
559078 128 55 64;
559078 144 71 81;

559348 176 88 110;
559348 176 88 8;
559348 144 69 82;
559369 176 88 8;
559369 176 88 115;
559369 144 55 43;
559442 128 55 64;
559442 128 71 64;
559734 176 88 115;
559734 176 88 3;
559734 144 67 74;
559765 176 88 3;
559765 176 88 52;
559765 144 50 42;
559838 128 69 64;
559854 128 50 64;
560005 128 43 64;
560166 176 88 52;
560166 176 88 4;
560166 144 50 45;
560229 128 50 64;
560723 176 88 4;
560723 176 88 0;
560723 144 65 53;
560755 176 88 0;
560755 176 88 84;
560755 128 67 64;
560755 144 41 43;
560760 176 64 127;
560760 176 64 0;
560854 176 64 0;
560854 176 64 127;
561036 176 88 84;
561036 176 88 127;
561036 144 48 47;
561119 128 48 64;
561317 176 88 127;
561317 176 88 89;
561317 144 53 59;
561578 176 88 89;
561578 176 88 92;
561578 144 60 61;
561640 128 65 64;
561708 128 60 64;
561833 176 88 92;
561833 176 88 68;
561833 144 60 46;
561838 176 88 68;
561838 176 88 74;
561838 144 65 61;
561869 128 53 64;
561895 176 88 74;
561895 176 88 111;
561895 144 69 76;
561906 128 65 64;
562166 176 88 111;
562166 176 88 76;
562166 144 67 84;
562187 176 88 76;
562187 176 88 124;
562187 144 53 53;
562281 128 69 64;
562291 128 53 64;
562447 128 41 64;
562515 176 88 124;
562515 176 88 79;
562515 144 48 47;
562781 128 48 64;
562838 128 60 64;
562859 176 88 79;
562859 176 88 16;
562859 144 65 51;
562963 128 67 64;
563015 176 88 16;
563015 176 88 68;
563015 144 64 50;
563067 128 65 64;
563166 176 88 68;
563166 176 88 66;
563166 144 38 54;
563166 176 88 66;
563166 176 88 65;
563166 144 62 83;
563182 128 64 64;
563197 176 64 127;
563197 176 64 0;
563302 176 64 0;
563302 176 64 127;
563390 176 88 65;
563390 176 88 1;
563390 144 45 60;
563447 128 45 64;
563718 176 88 1;
563718 176 88 72;
563718 144 50 79;
563989 176 88 72;
563989 176 88 105;
563989 144 57 78;
564015 128 62 64;
564276 176 88 105;
564276 176 88 65;
564276 144 62 83;
564380 128 50 64;
564416 176 64 127;
564416 176 64 0;
564515 176 64 0;
564515 176 64 127;
564541 176 88 65;
564541 176 88 33;
564541 144 64 79;
564546 176 88 33;
564546 176 88 52;
564546 144 50 68;
564843 176 88 52;
564843 176 88 14;
564843 144 65 94;
564885 128 62 64;
564885 128 50 64;
564916 128 64 64;
565067 128 57 64;
565109 128 38 64;
565130 176 88 14;
565130 176 88 108;
565130 144 57 88;
565135 176 88 108;
565135 176 88 63;
565135 144 50 57;
565250 128 65 64;
565406 128 57 64;
565416 128 50 64;
565463 176 88 63;
565463 176 88 124;
565463 144 64 98;
565463 176 88 124;
565463 176 88 4;
565463 144 40 81;
565723 176 88 4;
565723 176 88 110;
565723 144 47 50;
565750 128 64 64;
565791 128 47 64;
566005 176 88 110;
566005 176 88 113;
566005 144 52 57;
566072 128 52 64;
566296 176 88 113;
566296 176 88 127;
566296 144 71 80;
566307 176 88 127;
566307 176 88 77;
566307 144 62 60;
566307 144 68 73;
566307 176 88 77;
566307 176 88 79;
566307 144 64 63;
566359 128 64 64;
566359 128 62 64;
566395 128 71 64;
566421 128 68 64;
566578 176 88 79;
566578 176 88 72;
566578 144 52 57;
566869 176 88 72;
566869 176 88 37;
566869 144 74 70;
566869 144 83 86;
566880 176 88 37;
566880 176 88 59;
566880 144 76 84;
566880 144 80 74;
566901 128 52 64;
567031 128 76 64;
567041 128 74 64;
567166 128 83 64;
567187 128 80 64;
567213 176 88 59;
567213 176 88 113;
567213 144 52 87;
567546 176 88 113;
567546 176 88 89;
567546 144 86 57;
567557 176 88 89;
567557 176 88 28;
567557 144 88 80;
567656 128 52 64;
567697 128 88 64;
567708 128 86 64;
567802 128 40 64;
567895 176 88 28;
567895 176 88 20;
567895 144 95 96;
567906 176 88 20;
567906 176 88 5;
567906 144 40 52;
567911 176 88 5;
567911 176 88 13;
567911 144 86 72;
567911 144 88 66;
567911 144 92 75;
568255 176 88 13;
568255 176 88 104;
568255 144 47 68;
568416 128 47 64;
568557 128 88 64;
568572 128 86 64;
568588 176 88 104;
568588 176 88 70;
568588 144 52 86;
568645 128 95 64;
568890 176 88 70;
568890 176 88 88;
568890 144 86 45;
568911 128 92 64;
568932 176 88 88;
568932 176 88 16;
568932 144 88 26;
569005 128 88 64;
569020 128 52 64;
569041 128 86 64;
569255 176 88 16;
569255 176 88 120;
569255 144 86 28;
569265 176 88 120;
569265 176 88 21;
569265 144 95 78;
569281 176 88 21;
569281 176 88 40;
569281 144 88 22;
569348 128 86 64;
569354 128 88 64;
569593 176 88 40;
569593 176 88 112;
569593 144 92 67;
569619 176 88 112;
569619 176 88 8;
569619 144 52 50;
569708 128 52 64;
569723 128 95 64;
569828 176 88 8;
569828 176 88 98;
569828 144 88 71;
569843 128 92 64;
569885 128 88 64;
570015 176 88 98;
570015 176 88 33;
570015 144 83 71;
570119 128 83 64;
570177 176 88 33;
570177 176 88 35;
570177 144 80 60;
570312 128 80 64;
570359 176 88 35;
570359 176 88 78;
570359 144 76 57;
570432 128 76 64;
570640 176 88 78;
570640 176 88 33;
570640 144 52 18;
570708 128 52 64;
570760 176 88 33;
570760 176 88 71;
570760 144 71 80;
571072 176 88 71;
571072 176 88 115;
571072 128 71 64;
571072 144 69 51;
571083 176 88 115;
571083 176 88 92;
571083 144 52 30;
571130 128 69 64;
571166 128 52 64;
571177 176 88 92;
571177 176 88 98;
571177 144 71 41;
571229 176 88 98;
571229 176 88 59;
571229 144 69 47;
571244 128 71 64;
571296 128 69 64;
571296 176 88 59;
571296 176 88 101;
571296 144 71 75;
571369 128 71 64;
571401 176 88 101;
571401 176 88 20;
571401 144 68 65;
571562 128 68 64;
571677 176 88 20;
571677 176 88 127;
571677 144 64 68;
571760 128 64 64;
571911 176 88 127;
571911 176 88 51;
571911 144 62 84;
571973 128 62 64;
572109 128 40 64;
572171 176 88 51;
572171 176 88 36;
572171 144 62 85;
572307 128 62 64;
572406 176 88 36;
572406 176 88 84;
572406 144 64 86;
572447 176 88 84;
572447 176 88 75;
572447 144 52 34;
572500 128 64 64;
572526 128 52 64;
572656 176 88 75;
572656 176 88 103;
572656 144 68 83;
572708 128 68 64;
572906 176 88 103;
572906 176 88 115;
572906 144 69 96;
572921 176 88 115;
572921 176 88 46;
572921 144 52 35;
573000 128 69 64;
573005 128 52 64;
573166 176 88 46;
573166 176 88 55;
573166 144 71 95;
573239 128 71 64;
573421 176 64 127;
573421 176 64 0;
573442 176 88 55;
573442 176 88 31;
573442 144 52 44;
573453 176 88 31;
573453 176 88 82;
573453 144 72 87;
573541 176 64 0;
573541 176 64 127;
573744 128 52 64;
573765 176 88 82;
573765 176 88 120;
573765 144 74 77;
573776 128 72 64;
574088 176 88 120;
574088 176 88 60;
574088 144 76 68;

574104 128 74 64;
574104 176 88 60;
574104 176 88 123;
574104 144 52 35;
574213 128 52 64;
574416 128 76 64;
574484 176 88 123;
574484 176 88 110;
574484 144 74 44;
574885 176 88 110;
574885 176 88 60;
574885 144 52 33;
575229 128 52 64;
575270 128 74 64;
575651 176 88 60;
575651 176 88 62;
575651 144 64 34;
575755 176 88 62;
575755 176 88 10;
575755 144 69 30;
575796 176 88 10;
575796 176 88 61;
575796 144 45 45;
575817 176 88 61;
575817 176 88 112;
575817 144 72 50;
575843 128 69 64;
575875 128 64 64;
576151 176 88 112;
576151 176 88 43;
576151 144 52 41;
576229 128 52 64;
576229 128 45 64;
576437 176 88 43;
576437 176 88 21;
576437 144 57 39;
576526 128 57 64;
576718 176 88 21;
576718 176 88 115;
576718 144 60 36;
576744 128 72 64;
577020 176 88 115;
577020 176 88 36;
577020 144 64 39;
577302 176 88 36;
577302 176 88 104;
577302 144 72 34;
577307 176 88 104;
577307 176 88 43;
577307 144 76 26;
577359 128 60 64;
577375 128 64 64;
577635 128 76 64;
577682 128 72 64;
577682 176 88 43;
577682 176 88 65;
577682 144 69 48;
578000 176 88 65;
578000 176 88 36;
578000 144 64 39;
578031 128 69 64;
578041 176 88 36;
578041 176 88 110;
578041 144 72 31;
578088 128 64 64;
578364 128 72 64;
578395 176 88 110;
578395 176 88 49;
578395 144 69 28;
578744 128 69 64;
579671 176 64 127;
579671 176 64 0;
732447 176 88 49;
732447 176 88 31;
732447 144 66 47;
734744 128 66 64;
734953 176 88 31;
734953 176 88 80;
734953 144 66 39;
735005 176 88 80;
735005 176 88 42;
735005 144 70 46;
735031 128 66 64;
735046 176 88 42;
735046 176 88 58;
735046 144 73 47;
735078 128 70 64;
735380 176 88 58;
735380 176 88 78;
735380 144 70 39;
735442 128 73 64;
735515 176 88 78;
735515 176 88 89;
735515 144 66 81;
735588 128 70 64;
735833 176 88 89;
735833 176 88 3;
735833 144 70 59;
735973 128 66 64;
736005 176 88 3;
736005 176 88 101;
736005 144 73 58;
736197 128 70 64;
736317 176 88 101;
736317 176 88 25;
736317 144 75 83;
736364 128 73 64;
737734 128 75 64;
737828 176 88 25;
737828 176 88 32;
737828 144 75 36;
738026 176 88 32;
738026 176 88 19;
738026 144 78 64;
738062 128 75 64;
738395 176 88 19;
738395 176 88 103;
738395 144 75 20;
738562 176 88 103;
738562 176 88 4;
738562 144 80 84;
738666 128 75 64;
738677 128 78 64;
739078 176 88 4;
739078 176 88 63;
739078 144 82 79;
739098 176 88 63;
739098 176 88 127;
739098 144 75 7;
739171 128 80 64;
739177 128 75 64;
739343 176 88 127;
739343 176 88 10;
739343 144 75 42;
739437 128 75 64;
739609 176 88 10;
739609 176 88 61;
739609 144 80 89;
739718 128 82 64;
740088 176 88 61;
740088 176 88 125;
740088 144 78 70;
740166 128 80 64;
740406 176 88 125;
740406 176 88 50;
740406 144 75 72;
740427 128 78 64;
741729 128 75 64;
741916 176 88 50;
741916 176 88 58;
741916 144 75 46;
742119 176 88 58;
742119 176 88 112;
742119 144 87 68;
742260 128 75 64;
742916 128 87 64;
742958 176 88 112;
742958 176 88 30;
742958 144 75 44;
743161 176 88 30;
743161 176 88 125;
743161 144 87 85;
743244 128 75 64;
743510 128 87 64;
743531 176 88 125;
743531 176 88 37;
743531 144 75 32;
743692 176 88 37;
743692 176 88 93;
743692 144 87 96;
743776 128 75 64;
744041 176 88 93;
744041 176 88 97;
744041 144 75 44;
744052 128 87 64;
744192 176 88 97;
744192 176 88 14;
744192 144 87 93;
744281 128 75 64;
744463 128 87 64;
744526 176 88 14;
744526 176 88 4;
744526 144 85 89;
745291 128 85 64;
745744 176 88 4;
745744 176 88 11;
745744 144 80 90;
745958 128 80 64;
745963 176 88 11;
745963 176 88 66;
745963 144 82 51;
746041 128 82 64;
746072 176 88 66;
746072 176 88 111;
746072 144 80 28;
746130 176 88 111;
746130 176 88 49;
746130 144 82 71;
746156 128 80 64;
746213 176 88 49;
746213 176 88 94;
746213 144 80 75;
746291 128 82 64;
746328 176 88 94;
746328 176 88 45;
746328 144 78 68;
746348 128 80 64;
746406 128 78 64;
746572 176 88 45;
746572 176 88 26;
746572 144 75 79;
746802 176 88 26;
746802 176 88 113;
746802 144 73 86;
746869 128 75 64;
747078 128 73 64;
747093 176 88 113;
747093 176 88 7;
747093 144 70 51;
747161 128 70 64;
747265 176 88 7;
747265 176 88 60;
747265 144 68 75;
747572 176 88 60;
747572 176 88 115;
747572 144 66 57;
747578 128 68 64;
747651 128 66 64;
747765 176 88 115;
747765 176 88 0;
747765 144 63 70;
748041 128 63 64;
748083 176 88 0;
748083 176 88 42;
748083 144 61 46;
748229 128 61 64;
748265 176 88 42;
748265 176 88 68;
748265 144 58 70;
748567 128 58 64;
748598 176 88 68;
748598 176 88 9;
748598 144 61 67;
748885 176 88 9;
748885 176 88 83;
748885 144 63 72;
748947 128 61 64;
750114 176 88 83;
750114 176 88 2;
750114 144 51 56;
750145 128 63 64;
750343 128 51 64;
750619 176 88 2;
750619 176 88 39;
750619 144 51 90;
750848 176 88 39;
750848 176 88 56;
750848 128 51 64;
750848 144 51 32;
750848 144 63 89;
750968 128 51 64;
751671 128 63 64;
751703 176 88 56;
751703 176 88 122;
751703 144 51 32;
751927 176 88 122;
751927 176 88 50;
751927 144 63 84;
752015 128 51 64;
752286 176 88 50;
752286 176 88 18;
752286 128 63 64;
752286 144 51 40;
752427 176 88 18;
752427 176 88 110;
752427 144 63 80;
752489 128 51 64;
752651 128 63 64;
752734 176 88 110;
752734 176 88 114;
752734 144 63 58;
754171 128 63 64;
754322 176 88 114;
754322 176 88 95;
754322 144 73 73;
754338 176 88 95;
754338 176 88 86;
754338 144 63 61;
754489 128 63 64;
754489 128 73 64;
754786 176 88 86;
754786 176 88 39;
754786 144 73 74;
754791 176 88 39;
754791 176 88 37;
754791 144 63 66;
754963 128 73 64;
754973 128 63 64;
755302 176 88 37;
755302 176 88 56;
755302 144 63 76;
755302 144 73 75;
755468 128 63 64;
755536 128 73 64;
755786 176 88 56;
755786 176 88 4;
755786 144 73 61;
755802 176 88 4;
755802 176 88 65;
755802 144 63 47;
755916 128 63 64;
755994 128 73 64;
756036 176 88 65;
756036 176 88 76;
756036 144 63 71;
756046 176 88 76;
756046 176 88 46;
756046 144 70 77;
756265 128 63 64;
756385 128 70 64;
756411 176 88 46;
756411 176 88 68;
756411 144 63 70;
756416 176 88 68;
756416 176 88 22;
756416 144 68 69;
756614 128 63 64;
756723 128 68 64;
756776 176 88 22;
756776 176 88 125;
756776 144 66 83;
756994 128 66 64;
757114 176 88 125;
757114 176 88 25;
757114 144 63 86;
757911 128 63 64;
757916 176 88 25;
757916 176 88 67;
757916 144 61 45;
757994 128 61 64;
758125 176 88 67;
758125 176 88 115;

758125 144 70 85;
758161 176 88 115;
758161 176 88 98;
758161 144 61 24;
758244 128 61 64;
758463 128 70 64;
758473 144 61 37;
758557 128 61 64;
758968 176 88 98;
758968 176 88 90;
758968 144 61 69;
759177 176 88 90;
759177 176 88 74;
759177 144 70 83;
759265 128 61 64;
759546 128 70 64;
759557 176 88 74;
759557 176 88 61;
759557 144 61 27;
759651 128 61 64;
760067 176 88 61;
760067 176 88 18;
760067 144 61 78;
760250 176 88 18;
760250 176 88 3;
760250 144 70 83;
760270 128 61 64;
760364 128 70 64;
760572 176 88 3;
760572 176 88 123;
760572 144 73 90;
760781 128 73 64;
761005 176 88 123;
761005 176 88 83;
761005 144 82 89;
762052 128 82 64;
762156 176 88 83;
762156 176 88 91;
762156 144 73 32;
762260 128 73 64;
762348 176 88 91;
762348 176 88 105;
762348 144 82 84;
762546 128 82 64;
762645 176 88 105;
762645 176 88 90;
762645 144 82 53;
762911 128 82 64;
762984 176 88 90;
762984 176 88 108;
762984 144 82 81;
763234 128 82 64;
763317 176 88 108;
763317 176 88 29;
763317 144 82 87;
763598 128 82 64;
763671 176 88 29;
763671 176 88 109;
763671 144 82 90;
763916 128 82 64;
764020 176 88 109;
764020 176 88 28;
764020 144 82 84;
764182 128 82 64;
764265 176 88 28;
764265 176 88 6;
764265 144 82 48;
764296 176 88 6;
764296 176 88 103;
764296 144 85 69;
764328 128 82 64;
764348 176 88 103;
764348 176 88 32;
764348 144 87 66;
764390 128 85 64;
764645 176 88 32;
764645 176 88 6;
764645 144 82 89;
764682 128 87 64;
764968 176 88 6;
764968 176 88 55;
764968 144 90 88;
765020 128 82 64;
765218 128 90 64;
765307 176 88 55;
765307 176 88 66;
765307 144 92 99;
766130 176 88 66;
766130 176 88 100;
766130 144 90 98;
766218 128 92 64;
766916 176 88 100;
766916 176 88 44;
766916 144 87 88;
766979 128 90 64;
767203 176 88 44;
767203 176 88 37;
767203 144 94 91;
767354 128 87 64;
767755 176 88 37;
767755 176 88 74;
767755 144 87 83;
767812 128 94 64;
767963 176 88 74;
767963 176 88 76;
767963 144 92 94;
768026 128 87 64;
768302 176 88 76;
768302 176 88 41;
768302 144 90 70;
768322 128 92 64;
768479 176 88 41;
768479 176 88 0;
768479 144 92 94;
768505 128 90 64;
768791 176 88 0;
768791 176 88 63;
768791 144 90 79;
768828 128 92 64;
768963 176 88 63;
768963 176 88 76;
768963 144 92 94;
769020 128 90 64;
769270 176 88 76;
769270 176 88 73;
769270 144 90 105;
769338 128 92 64;
769729 128 90 64;
769760 176 88 73;
769760 176 88 120;
769760 144 87 88;
771015 176 88 120;
771015 176 88 48;
771015 144 85 91;
771067 128 87 64;
771182 176 88 48;
771182 176 88 118;
771182 144 87 69;
771197 128 85 64;
771265 128 87 64;
771348 176 88 118;
771348 176 88 120;
771348 144 87 78;
771447 176 88 120;
771447 176 88 58;
771447 144 85 88;
771536 128 87 64;
771588 176 88 58;
771588 176 88 55;
771588 144 82 82;
771630 128 85 64;
771854 128 82 64;
771906 176 88 55;
771906 176 88 112;
771906 144 80 88;
772255 176 88 112;
772255 176 88 66;
772255 144 78 92;
772276 128 80 64;
772489 176 88 66;
772489 176 88 12;
772489 144 75 96;
772526 128 78 64;
772822 176 88 12;
772822 176 88 37;
772822 144 78 74;
772880 128 75 64;
772984 176 88 37;
772984 176 88 70;
772984 144 80 74;
773229 128 78 64;
773276 128 80 64;
773281 176 88 70;
773281 176 88 62;
773281 144 75 95;
773291 176 88 62;
773291 176 88 7;
773291 144 82 92;
774140 128 75 64;
774177 128 82 64;
774312 176 88 7;
774312 176 88 106;
774312 144 80 82;
774317 176 88 106;
774317 176 88 99;
774317 144 85 75;
774317 176 88 99;
774317 176 88 81;
774317 144 75 74;
774369 128 80 64;
774369 128 85 64;
774369 128 75 64;
774833 176 88 81;
774833 176 88 111;
774833 144 85 71;
774838 176 88 111;
774838 176 88 20;
774838 144 75 65;
774838 176 88 20;
774838 176 88 88;
774838 144 80 61;
775203 128 75 64;
775218 128 85 64;
775286 128 80 64;
775359 176 88 88;
775359 176 88 74;
775359 144 73 76;
775364 176 88 74;
775364 176 88 78;
775364 144 82 83;
775375 176 88 78;
775375 176 88 65;
775375 144 78 71;
776359 128 73 64;
776401 128 82 64;
776437 128 78 64;
776609 176 88 65;
776609 176 88 63;
776609 144 73 70;
776609 144 82 79;
776614 176 88 63;
776614 176 88 79;
776614 144 78 76;
776869 128 73 64;
776937 176 88 79;
776937 176 88 60;
776937 144 80 80;
776942 176 88 60;
776942 176 88 46;
776942 144 73 63;
776958 128 82 64;
777000 128 78 64;
777239 128 73 64;
777322 176 88 46;
777322 176 88 97;
777322 144 78 87;
777427 128 80 64;
777598 176 88 97;
777598 176 88 113;
777598 128 78 64;
777598 144 75 97;
778468 128 75 64;
778625 176 88 113;
778625 176 88 39;
778625 144 73 86;
778635 176 88 39;
778635 176 88 67;
778635 144 82 65;
778677 128 73 64;
778682 128 82 64;
778979 176 88 67;
778979 176 88 69;
778979 144 73 91;
778979 176 88 69;
778979 176 88 32;
778979 144 82 90;
779244 128 73 64;
779265 128 82 64;
779401 176 88 32;
779401 176 88 82;
779401 144 73 95;
779401 176 88 82;
779401 176 88 41;
779401 144 80 94;
779937 176 88 41;
779937 176 88 25;
779937 144 78 77;
779953 128 73 64;
779979 128 80 64;
780098 176 88 25;
780098 176 88 65;
780098 144 80 77;
780161 128 78 64;
780411 176 88 65;
780411 176 88 4;
780411 144 78 76;
780458 128 80 64;
780593 176 88 4;
780593 176 88 7;
780593 144 80 87;
780630 128 78 64;
780932 176 88 7;
780932 176 88 103;
780932 144 78 47;
780994 128 80 64;
781088 176 88 103;
781088 176 88 26;
781088 144 75 79;
781104 128 78 64;
781166 128 75 64;
781406 176 88 26;
781406 176 88 36;
781406 144 73 93;
782947 176 88 36;
782947 176 88 90;
782947 144 70 41;
782989 128 73 64;
783052 128 70 64;
783104 176 88 90;
783104 176 88 123;
783104 144 73 90;
783447 176 88 123;
783447 176 88 84;
783447 144 70 44;
783473 128 73 64;
783630 176 88 84;
783630 176 88 28;
783630 144 68 87;
783661 128 70 64;
783958 176 88 28;
783958 176 88 13;
783958 144 70 55;
784010 128 68 64;
784119 176 88 13;
784119 176 88 88;
784119 144 68 71;
784161 128 70 64;
784421 128 68 64;
784453 144 66 49;
784609 176 88 88;
784609 176 88 46;
784609 144 68 79;
784630 128 66 64;
784906 176 88 46;
784906 176 88 77;
784906 144 66 45;
784911 128 68 64;
784973 128 66 64;
785031 176 88 77;
785031 176 88 40;
785031 144 68 39;
785359 176 88 40;
785359 176 88 106;
785359 144 63 73;
785385 128 68 64;
786244 128 63 64;
786458 176 88 106;
786458 176 88 80;
786458 144 63 82;
786666 176 88 80;
786666 176 88 126;
786666 144 61 83;

786781 128 63 64;
787052 128 61 64;
787510 176 88 126;
787510 176 88 60;
787510 144 61 73;
787718 144 58 69;
787739 128 61 64;
788005 128 58 64;
788041 176 88 60;
788041 176 88 16;
788041 144 56 64;
788197 176 88 16;
788197 176 88 50;
788197 144 54 83;
788244 128 56 64;
788468 128 54 64;
788494 176 88 50;
788494 176 88 3;
788494 144 51 67;
788713 176 88 3;
788713 176 88 54;
788713 144 49 83;
788739 128 51 64;
789020 176 88 54;
789020 176 88 6;
789020 128 49 64;
789020 144 46 49;
789177 176 88 6;
789177 176 88 3;
789177 144 44 67;
789192 128 46 64;
789500 176 88 3;
789500 176 88 87;
789500 128 44 64;
789500 144 42 81;
789703 176 88 87;
789703 176 88 121;
789703 144 39 84;
789765 128 42 64;
790906 128 39 64;
791197 176 88 121;
791197 176 88 58;
791197 144 37 81;
791390 176 88 58;
791390 176 88 30;
791390 144 39 57;
791411 128 37 64;
791536 176 88 30;
791536 176 88 91;
791536 144 37 41;
791546 128 39 64;
791614 128 37 64;
791614 176 88 91;
791614 176 88 20;
791614 144 39 65;
791734 176 88 20;
791734 176 88 6;
791734 144 37 82;
791802 128 39 64;
792932 128 37 64;
793276 176 88 6;
793276 176 88 100;
793276 144 25 102;
793572 128 25 64;
793755 176 88 100;
793755 176 88 32;
793755 144 27 110;
794125 128 27 64;
808921 144 39 66;
809135 128 39 64;
809437 176 88 32;
809437 176 88 98;
809437 144 39 56;
809692 128 39 64;
809973 176 88 98;
809973 176 88 112;
809973 144 37 62;
810203 128 37 64;
810500 176 88 112;
810500 176 88 59;
810500 144 37 61;
810802 128 37 64;
811057 176 88 59;
811057 176 88 74;
811057 144 35 57;
811328 128 35 64;
811604 176 88 74;
811604 176 88 116;
811604 144 35 57;
811828 128 35 64;
812125 176 88 116;
812125 176 88 101;
812125 144 34 44;
812645 128 34 64;
813093 176 88 101;
813093 176 88 13;
813093 144 39 12;
813171 128 39 64;
813572 176 88 13;
813572 176 88 85;
813572 144 37 14;
813682 128 37 64;
814703 176 88 85;
814703 176 88 125;
814703 144 35 39;
815088 128 35 64;
815317 176 88 125;
815317 176 88 115;
815317 144 34 36;
815973 128 34 64;
817786 176 88 115;
817786 176 88 111;
817786 144 39 41;
818026 128 39 64;
818322 176 88 111;
818322 176 88 81;
818322 144 39 48;
818546 128 39 64;
818822 176 88 81;
818822 176 88 97;
818822 144 37 45;
819046 128 37 64;
819338 176 88 97;
819338 176 88 73;
819338 144 37 43;
819598 128 37 64;
819875 176 88 73;
819875 176 88 23;
819875 144 35 50;
820156 128 35 64;
820395 176 88 23;
820395 176 88 0;
820395 144 35 43;
820588 128 35 64;
823588 176 88 0;
823588 176 88 21;
823588 144 39 39;
824729 128 39 64;
827125 176 88 21;
827125 176 88 115;
827125 144 39 48;
827375 128 39 64;
827682 176 88 115;
827682 176 88 60;
827682 144 39 79;
827932 128 39 64;
828213 176 88 60;
828213 176 88 17;
828213 144 37 73;
828484 128 37 64;
828760 176 88 17;
828760 176 88 122;
828760 144 37 63;
829010 128 37 64;
829276 176 88 122;
829276 176 88 86;
829276 144 35 69;
829541 128 35 64;
829828 176 88 86;
829828 176 88 64;
829828 144 35 81;
830088 128 35 64;
830359 176 88 64;
830359 176 88 48;
830359 144 34 73;
831104 128 34 64;
831453 176 88 48;
831453 176 88 108;
831453 144 39 75;
831723 128 39 64;
831989 176 88 108;
831989 176 88 31;
831989 144 39 75;
832234 128 39 64;
832510 176 88 31;
832510 176 88 2;
832510 144 37 72;
832760 128 37 64;
833036 176 88 2;
833036 176 88 4;
833036 144 37 65;
833250 128 37 64;
833578 176 88 4;
833578 176 88 86;
833578 144 35 69;
833755 128 35 64;
834666 176 88 86;
834666 176 88 102;
834666 144 39 66;
835734 176 88 102;
835734 176 88 73;
835734 144 37 65;
835750 128 39 64;
836854 176 88 73;
836854 176 88 121;
836854 144 35 60;
836890 128 37 64;
837656 176 88 121;
837656 176 88 29;
837656 144 34 55;
837703 128 35 64;
838989 176 88 29;
838989 176 88 101;
838989 144 39 52;
839046 128 34 64;
840083 176 88 101;
840083 176 88 5;
840083 144 37 60;
840125 128 39 64;
841213 128 37 64;
841270 176 88 5;
841270 176 88 68;
841270 144 36 46;
841458 128 36 64;
841557 176 88 68;
841557 176 88 32;
841557 144 35 65;
842411 128 35 64;
842703 176 88 32;
842703 176 88 88;
842703 144 34 61;
843645 176 88 88;
843645 176 88 28;
843645 144 75 67;
843822 176 88 28;
843822 176 88 126;
843822 144 39 68;
843828 176 88 126;
843828 176 88 77;
843828 144 82 73;
843864 128 34 64;
843968 128 75 64;
844968 176 88 77;
844968 176 88 58;
844968 144 37 81;
845015 128 39 64;
846104 176 88 58;
846104 176 88 112;
846104 144 35 67;
846135 128 37 64;
846432 176 88 112;
846432 176 88 124;
846432 144 80 44;
846546 128 82 64;
846614 176 88 124;
846614 176 88 58;
846614 144 82 91;
846697 128 80 64;
846947 176 88 58;
846947 176 88 62;
846947 144 80 68;
847026 128 82 64;
847114 176 88 62;
847114 176 88 93;
847114 144 82 77;
847125 176 88 93;
847125 176 88 41;
847125 144 34 76;
847161 128 35 64;
847177 128 80 64;
847432 176 88 41;
847432 176 88 95;
847432 144 80 64;
847520 128 82 64;
847619 176 88 95;
847619 176 88 121;
847619 144 78 97;
847671 128 80 64;
847916 128 78 64;
847979 176 88 121;
847979 176 88 69;
847979 144 78 89;
848182 176 88 69;
848182 176 88 20;
848182 144 39 65;
848223 128 34 64;
848411 176 88 20;
848411 176 88 118;
848411 144 75 76;
848437 128 78 64;
849281 176 88 118;
849281 176 88 35;
849281 144 37 80;
849333 128 39 64;
850406 176 88 35;
850406 176 88 44;
850406 144 35 77;
850437 128 37 64;
850953 176 88 44;
850953 176 88 5;
850953 144 73 70;
850979 128 75 64;
851187 176 88 5;
851187 176 88 127;
851187 144 75 58;
851218 128 73 64;
851322 176 88 127;
851322 176 88 49;
851322 144 73 48;
851333 128 75 64;
851411 176 88 49;
851411 176 88 62;
851411 144 75 81;
851416 128 73 64;
851427 176 88 62;
851427 176 88 71;
851427 144 34 69;
851437 128 35 64;
851723 176 88 71;
851723 176 88 6;
851723 144 73 88;
851796 128 75 64;
851911 176 88 6;
851911 176 88 74;
851911 144 70 94;
851953 128 73 64;
852250 128 70 64;
852265 176 88 74;
852265 176 88 28;
852265 144 68 87;
852468 176 88 28;
852468 176 88 10;
852468 144 39 70;
852479 176 88 10;
852479 176 88 50;
852479 144 66 83;
852541 128 34 64;
852546 128 68 64;
852848 176 88 50;
852848 176 88 102;
852848 144 63 89;
852885 128 66 64;
853244 176 88 102;
853244 176 88 29;
853244 144 70 76;
853427 128 63 64;
853437 176 88 29;
853437 176 88 59;
853442 144 37 61;
853479 128 39 64;
853781 176 88 59;
853781 176 88 66;

853781 144 63 78;
853848 128 70 64;
853968 176 88 66;
853968 176 88 125;
853968 144 73 83;
854078 128 63 64;
854296 176 88 125;
854296 176 88 101;
854296 144 63 45;
854328 128 73 64;
854468 176 88 101;
854468 176 88 112;
854468 144 35 67;
854479 176 88 112;
854479 176 88 122;
854479 128 37 64;
854479 144 75 91;
854625 128 63 64;
854838 176 88 122;
854838 176 88 67;
854838 144 63 37;
855005 128 75 64;
855026 176 88 67;
855026 176 88 106;
855026 144 73 82;
855140 128 63 64;
855354 176 88 106;
855354 176 88 38;
855354 144 63 32;
855463 128 35 64;
855526 176 88 38;
855526 176 88 81;
855526 144 34 62;
855531 176 88 81;
855531 176 88 50;
855531 144 70 73;
855625 128 73 64;
855630 128 63 64;
855864 176 88 50;
855864 176 88 77;
855864 144 63 45;
856041 128 63 64;
856052 176 88 77;
856052 176 88 32;
856052 144 68 73;
856166 128 70 64;
856354 176 88 32;
856354 176 88 60;
856354 144 66 87;
856406 128 68 64;
856453 128 34 64;
856765 176 88 60;
856765 176 88 54;
856765 144 34 10;
856963 176 88 54;
856963 176 88 56;
856963 144 39 45;
857031 128 34 64;
857500 128 39 64;
857583 176 88 56;
857583 176 88 1;
857583 144 38 60;
857651 128 38 64;
858041 176 88 1;
858041 176 88 86;
858041 144 37 79;
858369 128 37 64;
858552 176 88 86;
858552 176 88 119;
858552 144 37 61;
859078 176 88 119;
859078 176 88 113;
859078 144 35 76;
859093 128 37 64;
859333 128 35 64;
859401 176 88 113;
859401 176 88 11;
859401 144 35 75;
859552 128 66 64;
859598 176 88 11;
859598 176 88 60;
859598 144 63 69;
859921 176 88 60;
859921 176 88 98;
859921 144 61 88;
860010 128 63 64;
860145 176 88 98;
860145 176 88 89;
860145 128 35 64;
860145 144 34 71;
861260 128 34 64;
861380 176 88 89;
861380 176 88 34;
861380 144 39 78;
861791 128 39 64;
861875 128 61 64;
861911 176 88 34;
861911 176 88 20;
861911 144 39 65;
862427 176 88 20;
862427 176 88 59;
862427 144 37 60;
862427 144 82 94;
862463 128 39 64;
862833 128 37 64;
862932 176 88 59;
862932 176 88 119;
862932 144 37 61;
863473 176 88 119;
863473 176 88 48;
863473 144 35 61;
863494 128 37 64;
863796 176 88 48;
863796 176 88 81;
863796 144 85 78;
863875 128 82 64;
863973 176 88 81;
863973 176 88 111;
863973 144 37 69;
863994 128 35 64;
864244 176 88 111;
864244 176 88 4;
864244 144 87 76;
864276 128 37 64;
864307 176 88 4;
864307 176 88 53;
864307 144 35 51;
864338 128 85 64;
864515 128 35 64;
864541 176 88 53;
864541 176 88 21;
864541 144 34 71;
865614 176 88 21;
865614 176 88 56;
865614 144 39 68;
865656 128 34 64;
866015 128 39 64;
866057 128 87 64;
866135 176 88 56;
866135 176 88 45;
866135 144 39 67;
866484 176 88 45;
866484 176 88 81;
866484 144 82 41;
866651 176 88 81;
866651 176 88 0;
866651 144 94 95;
866661 176 88 0;
866661 176 88 35;
866661 144 37 80;
866713 128 39 64;
866786 128 82 64;
867015 176 88 35;
867015 176 88 48;
867015 144 82 49;
867145 128 94 64;
867197 176 88 48;
867197 176 88 102;
867197 144 92 89;
867291 128 82 64;
867505 176 88 102;
867505 176 88 55;
867505 144 90 82;
867625 128 92 64;
867697 128 37 64;
867713 176 88 55;
867713 176 88 68;
867713 144 35 75;
868046 176 88 68;
868046 176 88 120;
868046 144 87 88;
868119 128 90 64;
868572 176 88 120;
868572 176 88 55;
868572 144 90 82;
868630 128 87 64;
868744 176 88 55;
868744 176 88 74;
868744 144 87 83;
868744 176 88 74;
868744 176 88 19;
868744 144 34 75;
868786 128 35 64;
868859 128 90 64;
869078 176 88 19;
869078 176 88 84;
869078 144 85 57;
869088 128 87 64;
869223 128 85 64;
869265 176 88 84;
869265 176 88 116;
869265 144 82 78;
869572 176 88 116;
869572 176 88 84;
869572 144 80 87;
869651 128 82 64;
869781 176 88 84;
869781 176 88 102;
869781 144 39 72;
869822 128 34 64;
870052 176 88 102;
870052 176 88 92;
870052 144 78 65;
870067 128 80 64;
870255 128 78 64;
870276 176 88 92;
870276 176 88 115;
870276 144 75 72;
870302 176 88 115;
870302 176 88 24;
870302 144 37 67;
870348 128 39 64;
870635 176 88 24;
870635 176 88 71;
870635 144 73 80;
870697 128 75 64;
871119 176 88 71;
871119 176 88 28;
871119 144 70 88;
871151 128 73 64;
871322 176 88 28;
871322 176 88 118;
871322 144 39 54;
871328 176 88 118;
871328 176 88 37;
871328 144 73 78;
871359 128 37 64;
871390 128 70 64;
871619 128 73 64;
871625 176 88 37;
871625 176 88 95;
871625 144 70 46;
871828 128 39 64;
871848 176 88 95;
871848 176 88 79;
871848 144 35 54;
871848 144 68 64;
871880 128 70 64;
872161 176 88 79;
872161 176 88 46;
872161 144 66 63;
872239 128 68 64;
872661 176 88 46;
872661 176 88 105;
872661 144 63 54;
872708 128 66 64;
872859 176 88 105;
872859 176 88 26;
872859 144 66 89;
872864 176 88 26;
872864 176 88 75;
872864 144 34 56;
872901 128 35 64;
872932 128 63 64;
873213 176 88 75;
873213 176 88 26;
873213 144 63 61;
873234 128 66 64;
873380 176 88 26;
873380 176 88 93;
873380 144 66 85;
873453 128 63 64;
873703 176 88 93;
873703 176 88 74;
873703 144 63 75;
873729 128 66 64;
873911 176 88 74;
873911 176 88 3;
873911 144 39 74;
873953 128 34 64;
874338 128 39 64;
874447 176 88 3;
874447 176 88 63;
874447 144 39 82;
874890 128 39 64;
874942 176 88 63;
874942 176 88 107;
874942 144 37 83;
875317 128 37 64;
875453 176 88 107;
875453 176 88 13;
875453 144 37 76;
875869 128 37 64;
875958 176 88 13;
875958 176 88 74;
875958 144 35 86;
876333 128 35 64;
876473 176 88 74;
876473 176 88 65;
876473 144 35 60;
876791 128 63 64;
876984 176 88 65;
876984 176 88 7;
876984 144 34 81;
876989 128 35 64;
877406 128 34 64;
877510 176 88 7;
877510 176 88 5;
877510 144 34 57;
878015 128 34 64;
878057 176 88 5;
878057 176 88 23;
878057 144 39 86;
878078 176 88 23;
878078 176 88 4;
878078 144 80 84;
878328 128 39 64;
878401 176 88 4;
878401 176 88 41;
878401 144 82 70;
878427 128 80 64;
878494 176 88 41;
878494 176 88 81;
878494 128 82 64;
878494 144 80 62;
878505 176 88 81;
878505 176 88 70;
878505 144 39 60;
878562 176 88 70;
878562 176 88 116;
878562 144 82 78;
878578 128 80 64;
878822 176 88 116;
878822 176 88 54;
878822 144 80 97;
878838 128 39 64;
878921 128 82 64;
879015 176 88 54;
879015 176 88 66;
879015 144 37 75;
879041 176 88 66;
879041 176 88 99;
879041 144 78 77;
879093 128 80 64;
879343 176 88 99;
879343 176 88 108;
879343 144 80 85;
879354 128 37 64;
879406 128 78 64;
879505 176 88 108;
879505 176 88 57;
879505 144 37 84;

879520 176 88 57;
879520 176 88 74;
879520 144 78 83;
879567 128 80 64;
879833 128 37 64;
879854 176 88 74;
879854 176 88 8;
879854 144 75 74;
879901 128 78 64;
880041 176 88 8;
880041 176 88 39;
880041 144 35 68;
880244 128 35 64;
880541 176 88 39;
880541 176 88 64;
880541 144 35 81;
881046 128 35 64;
881067 176 88 64;
881067 176 88 61;
881067 144 34 89;
881369 176 88 61;
881369 176 88 34;
881369 144 73 82;
881463 128 75 64;
881536 176 88 34;
881536 176 88 14;
881536 144 70 91;
881598 128 73 64;
881864 176 88 14;
881864 176 88 101;
881864 144 73 89;
881916 128 70 64;
881994 128 34 64;
882062 176 88 101;
882062 176 88 31;
882062 144 39 75;
882093 176 88 31;
882093 176 88 79;
882093 144 75 89;
882171 128 73 64;
882359 128 39 64;
882479 128 75 64;
882541 176 88 79;
882541 176 88 114;
882541 144 75 94;
882546 176 88 114;
882546 176 88 9;
882546 144 39 80;
882791 128 39 64;
883031 176 88 9;
883031 176 88 110;
883031 144 37 80;
883343 128 37 64;
883348 176 88 110;
883348 176 88 6;
883348 144 73 95;
883447 128 75 64;
883515 176 88 6;
883515 176 88 56;
883515 144 35 76;
883546 176 88 56;
883546 176 88 68;
883546 144 70 90;
883614 128 73 64;
883786 128 35 64;
883838 176 88 68;
883838 176 88 50;
883838 144 73 83;
883895 128 70 64;
884026 176 88 50;
884026 176 88 111;
884026 144 35 36;
884078 176 88 111;
884078 176 88 81;
884078 144 34 84;
884083 176 88 81;
884083 176 88 8;
884083 144 75 74;
884104 128 73 64;
884109 128 35 64;
884421 128 34 64;
884468 128 75 64;
884520 176 88 8;
884520 176 88 10;
884520 144 75 99;
884817 176 88 10;
884817 176 88 5;
884817 144 34 57;
885177 176 88 5;
885177 176 88 26;
885177 144 73 89;
885437 128 75 64;
885697 176 88 26;
885697 176 88 126;
885697 144 39 68;
885708 176 88 126;
885708 176 88 1;
885708 144 70 94;
885750 128 73 64;
885776 128 34 64;
886197 128 39 64;
886265 176 88 1;
886265 176 88 20;
886265 144 39 65;
886291 176 88 20;
886291 176 88 117;
886291 144 68 74;
886333 128 70 64;
886645 128 39 64;
886666 176 88 117;
886666 176 88 10;
886666 144 39 70;
886666 144 63 82;
886734 128 68 64;
888286 128 39 64;
888343 128 63 64;
916166 176 88 10;
916166 176 88 100;
916166 144 75 49;
916473 128 75 64;
916479 176 88 100;
916479 176 88 14;
916479 144 87 45;
916692 128 87 64;
916770 176 88 14;
916770 176 88 66;
916770 144 75 38;
916880 128 75 64;
916911 176 88 66;
916911 176 88 76;
916911 144 87 62;
916984 128 87 64;
917005 176 88 76;
917005 176 88 24;
917005 144 75 66;
917083 128 75 64;
917109 176 88 24;
917109 176 88 66;
917109 144 87 79;
917192 128 87 64;
917197 176 88 66;
917197 176 88 107;
917197 144 75 70;
917291 176 88 107;
917291 176 88 120;
917291 144 87 78;
917296 128 75 64;
917375 176 88 120;
917375 176 88 100;
917375 144 75 83;
917395 128 87 64;
917713 176 88 100;
917713 176 88 97;
917713 144 87 87;
917796 128 75 64;
918031 176 88 97;
918031 176 88 91;
918031 144 75 93;
918067 128 87 64;
918250 176 88 91;
918250 176 88 16;
918250 144 87 96;
918328 128 75 64;
918557 176 88 16;
918557 176 88 99;
918557 144 75 79;
918583 128 87 64;
918750 176 88 99;
918750 176 88 71;
918750 144 87 98;
918848 128 75 64;
919067 176 88 71;
919067 176 88 44;
919067 144 75 80;
919109 128 87 64;
919265 176 88 44;
919265 176 88 125;
919265 144 87 85;
919328 128 75 64;
919427 128 87 64;
919828 176 88 125;
919828 176 88 120;
919828 144 87 88;
919828 144 99 96;
919932 128 99 64;
919937 128 87 64;
920161 176 88 120;
920161 176 88 44;
920161 144 87 97;
920166 176 88 44;
920166 176 88 82;
920166 144 99 100;
921130 128 87 64;
921145 128 99 64;
921250 176 88 82;
921250 176 88 42;
921250 144 87 55;
921458 176 88 42;
921458 176 88 59;
921458 144 99 94;
921593 128 87 64;
921661 128 99 64;
921786 176 88 59;
921786 176 88 126;
921786 144 85 40;
921979 176 88 126;
921979 176 88 85;
921979 144 97 92;
922088 128 85 64;
922192 128 97 64;
922328 176 88 85;
922328 176 88 49;
922328 144 82 71;
922520 176 88 49;
922520 176 88 37;
922520 144 94 91;
922614 176 88 37;
922614 176 88 58;
922614 128 82 64;
922614 144 92 16;
922692 128 92 64;
922765 128 94 64;
922843 176 88 58;
922843 176 88 84;
922843 144 80 51;
923057 176 88 84;
923057 176 88 127;
923057 144 92 84;
923135 128 80 64;
923312 128 92 64;
923385 176 88 127;
923385 176 88 55;
923385 144 90 88;
923390 176 88 55;
923390 176 88 65;
923390 144 78 71;
923432 176 88 65;
923432 176 88 5;
923432 144 91 51;
923432 176 88 5;
923432 176 88 7;
923432 144 80 31;
923505 128 80 64;
923578 128 91 64;
923625 128 90 64;
923687 128 78 64;
923875 176 88 7;
923875 176 88 3;
923875 144 78 78;
923875 144 90 86;
924260 128 90 64;
924286 128 78 64;
924380 176 88 3;
924380 176 88 80;
924380 144 90 86;
924380 176 88 80;
924380 176 88 20;
924380 144 78 87;
924567 128 78 64;
924578 128 90 64;
924656 176 88 20;
924656 176 88 32;
924656 144 75 106;
924656 144 87 99;
925005 128 75 64;
925005 128 87 64;
925052 176 88 32;
925052 176 88 88;
925052 144 75 96;
925052 176 88 88;
925052 176 88 67;
925052 144 87 95;
925119 176 88 67;
925119 176 88 68;
925119 144 85 24;
925208 128 85 64;
925270 128 75 64;
925286 128 87 64;
925375 176 88 68;
925375 176 88 66;
925375 144 75 87;
925375 144 87 79;
925520 128 87 64;
925520 128 75 64;
925656 176 88 66;
925656 176 88 124;
925656 144 73 103;
925661 176 88 124;
925661 176 88 69;
925661 144 85 92;
925770 128 73 64;
925791 128 85 64;
925953 176 88 69;
925953 176 88 19;
925953 144 70 98;
925953 144 82 92;
926088 128 70 64;
926109 128 82 64;
926234 176 88 19;
926234 176 88 103;
926234 144 80 96;
926239 176 88 103;
926239 176 88 80;
926239 144 68 90;
926281 176 88 80;
926281 176 88 107;
926281 144 70 31;
926354 128 70 64;
926369 128 68 64;
926369 128 80 64;
926583 176 88 107;
926583 176 88 57;
926583 144 66 94;
926588 176 88 57;
926588 176 88 44;
926588 144 78 97;
926979 128 66 64;
926984 128 78 64;
927062 176 88 44;
927062 176 88 43;
927062 144 68 92;
927067 176 88 43;
927067 176 88 92;
927067 144 80 93;
927182 128 68 64;
927197 128 80 64;
927505 176 88 92;
927505 176 88 123;
927505 144 70 96;
927505 144 82 93;
927588 128 70 64;
927640 128 82 64;
927796 176 88 123;
927796 176 88 127;
927796 144 73 100;
927802 176 88 127;
927802 176 88 69;
927802 144 85 92;
927906 128 73 64;
927942 128 85 64;
928072 176 88 69;
928072 176 88 41;

928072 144 87 91;
928078 176 88 41;
928078 176 88 36;
928078 144 75 87;
928130 128 87 64;
928145 128 75 64;
928536 176 88 36;
928536 176 88 116;
928536 144 63 98;
928541 176 88 116;
928541 176 88 14;
928541 144 75 86;
928625 128 63 64;
928630 128 75 64;
928833 144 66 96;
928833 144 78 93;
930578 128 78 64;
930598 128 66 64;
930796 176 88 14;
930796 176 88 110;
930796 144 63 80;
930812 176 88 110;
930812 176 88 33;
930812 144 75 68;
930848 128 63 64;
930864 128 75 64;
930984 176 88 33;
930984 176 88 69;
930984 144 66 91;
930989 176 88 69;
930989 176 88 14;
930989 144 78 93;
931067 128 66 64;
931109 128 78 64;
931286 176 88 14;
931286 176 88 45;
931286 144 63 67;
931296 176 88 45;
931296 176 88 101;
931296 144 75 60;
931333 128 63 64;
931359 128 75 64;
931463 176 88 101;
931463 176 88 41;
931463 144 78 91;
931463 176 88 41;
931463 176 88 39;
931463 144 66 74;
931515 128 66 64;
931515 128 78 64;
931781 176 88 39;
931781 176 88 106;
931781 144 63 73;
931828 176 88 106;
931828 176 88 116;
931828 144 75 28;
931833 128 63 64;
931911 128 75 64;
932000 176 88 116;
932000 176 88 69;
932000 144 66 91;
932005 176 88 69;
932005 176 88 41;
932005 144 78 91;
932151 128 66 64;
932197 128 78 64;
932338 176 88 41;
932338 176 88 92;
932338 144 80 93;
932338 176 88 92;
932338 176 88 65;
932338 144 68 85;
932651 128 80 64;
932651 128 68 64;
932817 176 88 65;
932817 176 88 67;
932817 144 66 97;
932817 144 78 95;
933718 128 78 64;
933723 128 66 64;
933864 176 88 67;
933864 176 88 44;
933864 144 63 77;
933864 144 75 75;
935312 128 75 64;
935328 128 63 64;
935484 176 88 44;
935484 176 88 79;
935484 144 75 62;
935494 176 88 79;
935494 176 88 101;
935494 144 63 45;
935557 128 75 64;
935572 128 63 64;
935687 176 88 101;
935687 176 88 54;
935687 144 73 79;
935692 176 88 54;
935692 176 88 44;
935692 144 61 72;
935755 128 61 64;
935822 128 73 64;
936010 176 88 44;
936010 176 88 21;
936010 144 58 76;
936015 176 88 21;
936015 176 88 28;
936015 144 70 68;
936067 128 58 64;
936088 128 70 64;
936208 176 88 28;
936208 176 88 80;
936208 144 73 88;
936213 176 88 80;
936213 176 88 28;
936213 144 61 75;
936296 128 61 64;
936354 128 73 64;
936526 176 88 28;
936526 176 88 44;
936526 144 58 77;
936546 176 88 44;
936546 176 88 40;
936546 144 70 44;
936578 128 58 64;
936619 128 70 64;
936718 176 88 40;
936718 176 88 39;
936718 144 61 79;
936729 176 88 39;
936729 176 88 16;
936729 144 72 73;
936807 128 72 64;
936833 128 61 64;
937088 176 88 16;
937088 176 88 0;
937088 144 63 88;
937093 176 88 0;
937093 176 88 79;
937093 144 75 89;
937937 128 75 64;
937963 128 63 64;
938119 176 88 79;
938119 176 88 81;
938119 144 63 63;
938130 176 88 81;
938130 176 88 42;
938130 144 75 56;
938171 128 63 64;
938192 128 75 64;
938312 176 88 42;
938312 176 88 85;
938312 144 80 98;
938333 176 88 85;
938333 176 88 6;
938333 144 70 39;
938338 176 88 6;
938338 176 88 44;
938338 144 68 61;
938395 128 70 64;
938484 128 68 64;
938484 128 80 64;
938656 176 88 44;
938656 176 88 21;
938656 144 63 76;
938671 176 88 21;
938671 176 88 16;
938671 144 75 64;
938703 128 63 64;
938739 128 75 64;
938859 176 88 16;
938859 176 88 90;
938859 144 82 95;
938869 176 88 90;
938869 176 88 36;
938869 144 70 71;
938921 128 70 64;
938958 128 82 64;
939192 176 88 36;
939192 176 88 105;
939192 144 63 66;
939208 176 88 105;
939208 176 88 4;
939208 144 75 55;
939239 128 63 64;
939260 128 75 64;
939406 176 88 4;
939406 176 88 34;
939406 144 73 82;
939411 176 88 34;
939411 176 88 61;
939411 144 84 68;
939453 176 88 61;
939453 176 88 78;
939453 128 73 64;
939453 144 82 15;
939463 128 84 64;
939510 128 82 64;
939739 176 88 78;
939739 176 88 76;
939739 144 63 71;
939765 176 88 76;
939765 176 88 71;
939765 144 75 43;
939786 128 63 64;
939828 128 75 64;
939994 176 88 71;
939994 176 88 110;
939994 144 75 103;
940015 176 88 110;
940015 176 88 30;
940015 144 85 44;
940020 176 88 30;
940020 176 88 94;
940020 144 87 36;
940041 176 88 94;
940041 176 88 41;
940041 144 86 37;
940098 128 85 64;
940171 128 75 64;
940171 128 86 64;
940171 128 87 64;
940328 176 88 41;
940328 176 88 12;
940328 144 75 96;
940328 176 88 12;
940328 176 88 14;
940328 144 87 93;
940578 128 75 64;
940593 128 87 64;
940713 176 88 14;
940713 176 88 9;
940713 144 75 102;
940713 144 87 98;
940994 128 75 64;
940994 128 87 64;
941083 176 88 9;
941083 176 88 16;
941083 144 87 96;
941088 144 75 93;
941276 128 75 64;
941281 128 87 64;
941416 176 88 16;
941416 176 88 9;
941416 144 75 108;
941416 176 88 9;
941416 176 88 76;
941416 144 87 101;
941484 176 88 76;
941484 176 88 48;
941484 144 85 17;
941562 128 85 64;
942213 128 87 64;
942333 128 75 64;
942515 176 88 48;
942515 176 88 54;
942515 144 87 82;
942864 176 88 54;
942864 176 88 52;
942864 144 75 42;
942869 128 87 64;
943010 128 75 64;
943036 176 88 52;
943036 176 88 4;
943036 144 85 89;
943380 176 88 4;
943380 176 88 50;
943380 144 73 46;
943427 128 85 64;
943890 128 73 64;
943963 176 88 50;
943963 176 88 60;
943963 144 73 87;
944182 176 88 60;
944182 176 88 62;
944182 144 85 81;
944234 128 73 64;
944484 176 88 62;
944484 176 88 105;
944484 144 73 51;
944515 128 85 64;
944572 128 73 64;
944666 176 88 105;
944666 176 88 117;
944666 144 87 91;
945000 128 87 64;
945010 176 88 117;
945010 176 88 87;
945010 144 75 40;
945098 128 75 64;
945187 176 88 87;
945187 176 88 116;
945187 144 87 94;
945421 128 87 64;
945505 176 88 116;
945505 176 88 120;
945505 144 87 78;
946031 128 87 64;
946625 176 88 120;
946625 176 88 36;
946625 144 99 57;
946796 176 88 36;
946796 176 88 76;
946796 144 87 62;
946843 128 99 64;
946963 128 87 64;
947708 176 88 76;
947708 176 88 98;
947708 144 87 55;
947880 176 88 98;
947880 176 88 28;
947880 144 75 57;
947947 128 87 64;
948010 128 75 64;
948781 176 88 28;
948781 176 88 42;
948781 144 75 56;
948963 176 88 42;
948963 176 88 55;
948963 144 63 58;
949026 128 75 64;
949083 128 63 64;
949348 176 88 55;
949348 176 88 12;
949348 144 63 79;
949557 128 63 64;
949765 176 88 12;
949765 176 88 4;
949765 144 63 82;
950760 128 63 64;
951015 176 88 4;
951015 176 88 62;
951015 144 63 76;
951015 144 75 77;
951135 128 63 64;
951145 128 75 64;
951510 176 88 62;
951510 176 88 111;
951510 144 61 72;
951510 144 73 77;
951635 128 61 64;
951645 128 73 64;

952041 176 88 111;
952041 176 88 119;
952041 144 58 77;
952041 176 88 119;
952041 176 88 96;
952041 144 70 80;
952182 128 58 64;
952187 128 70 64;
952406 176 88 96;
952406 176 88 103;
952406 144 68 83;
952411 176 88 103;
952411 176 88 25;
952411 144 56 83;
952520 128 56 64;
952552 128 68 64;
952880 176 88 25;
952880 176 88 125;
952880 144 54 83;
952880 176 88 125;
952880 176 88 1;
952880 144 66 76;
952958 128 54 64;
953005 128 66 64;
953083 176 88 1;
953083 176 88 101;
953083 144 68 87;
953104 176 88 101;
953104 176 88 63;
953104 144 56 49;
953104 144 58 39;
953161 128 58 64;
953161 128 56 64;
953213 128 68 64;
953390 176 88 63;
953390 176 88 126;
953390 144 54 67;
953416 176 88 126;
953416 176 88 115;
953416 144 66 41;
953442 128 54 64;
953479 128 66 64;
953567 176 88 115;
953567 176 88 121;
953567 144 68 84;
953572 176 88 121;
953572 176 88 62;
953572 144 56 81;
953687 128 56 64;
953697 128 68 64;
953916 176 88 62;
953916 176 88 36;
953916 144 54 93;
953916 144 66 86;
956140 128 54 64;
956354 128 66 64;
957203 176 88 36;
957203 176 88 3;
957203 144 78 72;
957348 176 88 3;
957348 176 88 55;
957348 144 90 82;
957375 128 78 64;
957656 128 90 64;
957661 176 88 55;
957661 176 88 42;
957661 144 78 49;
957807 176 88 42;
957807 176 88 54;
957807 144 90 85;
957901 128 78 64;
958067 128 90 64;
958145 176 88 54;
958145 176 88 4;
958145 144 90 83;
959796 128 90 64;
960723 176 88 4;
960723 176 88 12;
960723 144 78 63;
960854 128 78 64;
960875 176 88 12;
960875 176 88 32;
960875 144 90 69;
960973 176 88 32;
960973 176 88 25;
960973 144 78 65;
961010 128 90 64;
961098 176 88 25;
961098 176 88 110;
961098 144 90 51;
961104 128 78 64;
961156 128 90 64;
961182 176 88 110;
961182 176 88 98;
961182 144 78 66;
961265 128 78 64;
961291 176 88 98;
961291 176 88 100;
961291 144 90 74;
961354 128 90 64;
961385 176 88 100;
961385 176 88 51;
961385 144 78 69;
961468 128 78 64;
961479 176 88 51;
961479 176 88 3;
961479 144 90 86;
961562 176 88 3;
961562 176 88 112;
961562 144 78 68;
961572 128 90 64;
961671 176 88 112;
961671 176 88 116;
961671 144 90 78;
961677 128 78 64;
961750 128 90 64;
961765 176 88 116;
961765 176 88 42;
961765 144 78 55;
961854 128 78 64;
961875 176 88 42;
961875 176 88 25;
961875 144 90 74;
961927 128 90 64;
961947 176 88 25;
961947 176 88 27;
961947 144 78 53;
962067 128 78 64;
962072 176 88 27;
962072 176 88 105;
962072 144 90 84;
962156 176 88 105;
962156 176 88 64;
962156 144 78 50;
962161 128 90 64;
962286 176 88 64;
962286 176 88 4;
962286 144 90 55;
962317 128 78 64;
962385 176 88 4;
962385 176 88 99;
962385 128 90 64;
962385 144 90 37;
963229 128 90 64;
963364 176 88 99;
963364 176 88 62;
963364 144 82 36;
963536 128 82 64;
965807 176 88 62;
965807 176 88 86;
965807 144 82 40;
965984 176 88 86;
965984 176 88 33;
965984 144 94 74;
966052 128 82 64;
966322 128 94 64;
966359 176 88 33;
966359 176 88 46;
966359 144 82 36;
966437 128 82 64;
966583 176 88 46;
966583 176 88 85;
966583 144 97 92;
966984 176 88 85;
966984 176 88 79;
966984 144 85 63;
967062 128 97 64;
967312 128 85 64;
967473 176 88 79;
967473 176 88 11;
967473 144 97 75;
967921 128 97 64;
968000 176 88 11;
968000 176 88 62;
968000 144 85 81;
968187 176 88 62;
968187 176 88 99;
968187 144 97 95;
968250 128 85 64;
968520 176 88 99;
968520 176 88 84;
968520 144 85 57;
968552 128 97 64;
968776 128 85 64;
968791 176 88 84;
968791 176 88 35;
968791 144 97 79;
969031 128 97 64;
969062 176 88 35;
969062 176 88 84;
969062 144 85 57;
969130 128 85 64;
969187 176 88 84;
969187 176 88 60;
969187 144 97 63;
969239 128 97 64;
969276 176 88 60;
969276 176 88 28;
969276 144 85 67;
969333 128 85 64;
969375 176 88 28;
969375 176 88 86;
969375 144 97 78;
969432 128 97 64;
969463 176 88 86;
969463 176 88 24;
969463 144 85 66;
969557 176 88 24;
969557 176 88 77;
969557 128 85 64;
969557 144 97 89;
969656 128 97 64;
969687 176 88 77;
969687 176 88 29;
969687 144 82 87;
969786 128 82 64;
969802 176 88 29;
969802 176 88 49;
969802 144 94 71;
969885 128 94 64;
969895 176 88 49;
969895 176 88 84;
969895 144 82 67;
969968 128 82 64;
969989 176 88 84;
969989 176 88 6;
969989 144 94 73;
970104 128 94 64;
970114 176 88 6;
970114 176 88 86;
970114 144 82 30;
970114 176 88 86;
970114 176 88 4;
970114 144 80 84;
970171 128 80 64;
970182 128 82 64;
970229 176 88 4;
970229 176 88 19;
970229 144 92 44;
970260 176 88 19;
970260 176 88 111;
970260 144 80 53;
970307 128 92 64;
970390 176 88 111;
970390 176 88 92;
970390 144 92 55;
970421 128 80 64;
970500 176 88 92;
970500 176 88 99;
970500 144 80 35;
970510 176 88 99;
970510 176 88 74;
970510 144 78 83;
970515 128 92 64;
970572 128 78 64;
970572 128 80 64;
970625 176 88 74;
970625 176 88 58;
970625 144 90 72;
970671 176 88 58;
970671 176 88 24;
970671 144 78 42;
970703 128 90 64;
970817 128 78 64;
970890 176 88 24;
970890 176 88 88;
970890 144 75 96;
970984 176 88 88;
970984 176 88 85;
970984 144 87 56;
971067 128 75 64;
971354 176 88 85;
971354 176 88 39;
971354 144 75 12;
972083 128 87 64;
972114 128 75 64;
972244 176 88 39;
972244 176 88 87;
972244 144 75 65;
972244 176 88 87;
972244 176 88 4;
972244 144 87 76;
972364 128 75 64;
972390 128 87 64;
972723 176 88 4;
972723 176 88 26;
972723 144 73 89;
972734 176 88 26;
972734 176 88 62;
972734 144 85 81;
972807 128 73 64;
972854 128 85 64;
972932 176 88 62;
972932 176 88 17;
972932 144 87 90;
972932 176 88 17;
972932 176 88 113;
972932 144 75 59;
972984 128 87 64;
972994 128 75 64;
973223 176 88 113;
973223 176 88 109;
973223 144 73 78;
973250 176 88 109;
973250 176 88 56;
973250 144 85 59;
973286 128 73 64;
973317 128 85 64;
973473 176 88 56;
973473 176 88 105;
973473 144 90 84;
973489 176 88 105;
973489 176 88 103;
973489 144 78 47;
973500 176 88 103;
973500 176 88 7;
973500 144 80 31;
973557 128 78 64;
973567 128 80 64;
973609 128 90 64;
973791 176 88 7;
973791 176 88 107;
973791 144 92 95;
973791 176 88 107;
973791 176 88 61;
973791 144 80 89;
974020 128 80 64;
974031 128 92 64;
974234 176 88 61;
974234 176 88 106;
974234 144 82 87;
974244 176 88 106;
974244 176 88 61;
974244 144 94 81;
974463 128 82 64;
974520 128 94 64;
974760 176 88 61;
974760 176 88 54;
974760 144 82 85;
974921 128 82 64;
975015 176 88 54;
975015 176 88 2;

975015 144 82 73;
975270 128 82 64;
975338 176 88 2;
975338 176 88 93;
975338 144 82 77;
975515 128 82 64;
975583 176 88 93;
975583 176 88 58;
975583 144 82 72;
975807 128 82 64;
975869 176 88 58;
975869 176 88 29;
975869 144 82 87;
976401 176 88 29;
976401 176 88 89;
976401 144 94 62;
976619 128 82 64;
976953 128 94 64;
976968 176 88 89;
976968 176 88 106;
976968 144 82 56;
977161 176 88 106;
977161 176 88 114;
977161 144 94 83;
977250 128 82 64;
977437 128 94 64;
977479 176 88 114;
977479 176 88 127;
977479 144 82 35;
977692 176 88 127;
977692 176 88 31;
977692 144 94 61;
977744 128 82 64;
977911 128 94 64;
977958 176 88 31;
977958 176 88 103;
977958 144 82 47;
978244 128 82 64;
978244 176 88 103;
978244 176 88 98;
978244 144 94 47;
978348 128 94 64;
978380 176 88 98;
978380 176 88 9;
978380 144 82 45;
978453 128 82 64;
978510 176 88 9;
978510 176 88 72;
978510 144 94 45;
978578 176 88 72;
978578 176 88 74;
978578 128 94 64;
978578 144 82 54;
978677 128 82 64;
978703 176 88 74;
978703 176 88 100;
978703 144 94 53;
978765 128 94 64;
978786 176 88 100;
978786 176 88 72;
978786 144 82 52;
978911 176 88 72;
978911 176 88 8;
978911 144 94 76;
978932 128 82 64;
979036 176 88 8;
979036 176 88 32;
979036 128 94 64;
979036 144 82 50;
979161 176 88 32;
979161 176 88 109;
979161 144 94 64;
979182 128 82 64;
979260 128 94 64;
979312 176 88 109;
979312 176 88 46;
979312 144 80 52;
979343 176 88 46;
979343 176 88 8;
979343 144 82 31;
979411 128 82 64;
979437 176 88 8;
979437 176 88 6;
979437 144 92 51;
979447 128 80 64;
979531 176 88 6;
979531 176 88 95;
979531 144 80 60;
979541 128 92 64;
979656 128 80 64;
979677 176 88 95;
979677 176 88 19;
979677 144 92 44;
979739 128 92 64;
979791 176 88 19;
979791 176 88 17;
979791 144 80 15;
979796 176 88 17;
979796 176 88 101;
979796 144 78 29;
979859 128 80 64;
979864 128 78 64;
979906 176 88 101;
979906 176 88 37;
979906 144 90 23;
979932 176 88 37;
979932 176 88 58;
979932 144 78 58;
979979 128 90 64;
980062 176 88 58;
980062 176 88 106;
980062 128 78 64;
980062 144 90 56;
980151 176 88 106;
980151 176 88 73;
980151 144 78 44;
980166 128 90 64;
980265 176 88 73;
980265 176 88 94;
980265 144 90 57;
980276 128 78 64;
980354 128 90 64;
980375 176 88 94;
980375 176 88 90;
980375 144 75 86;
980505 128 75 64;
980515 176 88 90;
980515 176 88 78;
980515 144 87 49;
980583 128 87 64;
980583 176 88 78;
980583 176 88 20;
980583 144 75 62;
980713 128 75 64;
980744 176 88 20;
980744 176 88 54;
980744 144 87 29;
980812 176 88 54;
980812 176 88 37;
980812 144 73 78;
980817 128 87 64;
980932 128 73 64;
980947 176 88 37;
980947 176 88 113;
980947 144 85 59;
981031 176 88 113;
981031 176 88 92;
981031 144 73 55;
981041 128 85 64;
981156 128 73 64;
981161 176 88 92;
981161 176 88 87;
981161 144 85 65;
981223 128 85 64;
981270 176 88 87;
981270 176 88 74;
981270 144 70 83;
981385 128 70 64;
981421 176 88 74;
981421 176 88 110;
981421 144 82 62;
981505 176 88 110;
981505 176 88 33;
981505 144 70 70;
981536 128 82 64;
981630 176 88 33;
981630 176 88 100;
981630 144 82 69;
981692 128 70 64;
981755 176 88 100;
981755 176 88 30;
981755 144 70 38;
981770 128 82 64;
981864 128 70 64;
981895 176 88 30;
981895 176 88 93;
981895 144 80 40;
981927 176 88 93;
981927 176 88 117;
981927 144 68 79;
981937 176 88 117;
981937 176 88 76;
981937 144 70 37;
981958 128 80 64;
982000 128 70 64;
982052 128 68 64;
982057 176 88 76;
982057 176 88 23;
982057 144 80 42;
982156 176 88 23;
982156 176 88 95;
982156 144 68 45;
982213 128 80 64;
982244 128 68 64;
982328 176 88 95;
982328 176 88 91;
982328 144 68 32;
982328 176 88 91;
982328 176 88 74;
982328 144 66 76;
982395 128 68 64;
982489 176 88 74;
982489 176 88 120;
982489 144 78 78;
982500 128 66 64;
982614 176 88 120;
982614 176 88 115;
982614 144 66 57;
982640 128 78 64;
982765 176 88 115;
982765 176 88 117;
982765 144 78 58;
982786 128 66 64;
982859 176 88 117;
982859 176 88 28;
982859 144 66 53;
982880 128 78 64;
982947 128 66 64;
982994 144 75 57;
983031 176 88 28;
983031 176 88 78;
983031 144 63 62;
983119 128 75 64;
983213 176 88 78;
983213 176 88 94;
983213 144 75 53;
983223 128 63 64;
983322 176 88 94;
983322 176 88 29;
983322 144 63 45;
983348 128 75 64;
983473 176 88 29;
983473 176 88 113;
983473 144 75 59;
983515 128 63 64;
983578 176 88 113;
983578 176 88 120;
983578 144 63 68;
983598 128 75 64;
987296 128 63 64;
1029015 176 88 120;
1029015 176 88 12;
1029015 144 82 95;
1029020 176 88 12;
1029020 176 88 97;
1029020 144 78 87;
1029119 128 82 64;
1029130 128 78 64;
1029500 176 88 97;
1029500 176 88 32;
1029500 144 82 90;
1029567 176 88 32;
1029567 176 88 9;
1029567 144 77 23;
1029640 128 77 64;
1029640 128 82 64;
1029989 176 88 9;
1029989 176 88 25;
1029989 144 78 77;
1029994 176 88 25;
1029994 176 88 118;
1029994 144 82 63;
1030046 128 78 64;
1030052 128 82 64;
1030265 176 88 118;
1030265 176 88 58;
1030265 144 82 91;
1030270 176 88 58;
1030270 176 88 14;
1030270 144 78 93;
1031645 176 88 14;
1031645 176 88 89;
1031645 128 82 64;
1031645 144 75 61;
1031671 176 88 89;
1031671 176 88 3;
1031671 144 80 43;
1031687 128 78 64;
1031822 176 88 3;
1031822 176 88 86;
1031822 144 82 80;
1031916 128 80 64;
1031921 128 75 64;
1031953 176 88 86;
1031953 176 88 111;
1031953 144 78 27;
1032171 128 78 64;
1032208 176 88 111;
1032208 176 88 28;
1032208 144 80 61;
1032260 176 88 28;
1032260 176 88 91;
1032260 144 85 66;
1032328 128 82 64;
1032708 128 85 64;
1032755 128 80 64;
1032802 176 88 91;
1032802 176 88 84;
1032802 144 80 87;
1032802 176 88 84;
1032802 176 88 99;
1032802 144 85 75;
1033072 128 85 64;
1033104 128 80 64;
1033135 176 88 99;
1033135 176 88 20;
1033135 144 85 65;
1033140 176 88 20;
1033140 176 88 21;
1033140 144 80 71;
1033296 128 80 64;
1033302 128 85 64;
1033369 176 88 21;
1033369 176 88 113;
1033369 144 80 66;
1033380 176 88 113;
1033380 176 88 33;
1033380 144 85 68;
1033635 128 85 64;
1033708 128 80 64;
1033723 176 88 33;
1033723 176 88 81;
1033723 144 85 74;
1034052 176 88 81;
1034052 176 88 47;
1034052 144 82 75;
1034140 128 85 64;
1034348 176 88 47;
1034348 176 88 106;
1034348 144 80 82;
1034421 128 82 64;
1034708 176 88 106;
1034708 176 88 58;
1034708 144 78 75;
1034770 128 80 64;
1035135 128 78 64;
1035135 176 88 58;
1035135 176 88 45;
1035135 144 75 50;
1035223 128 75 64;
1035260 176 88 45;
1035260 176 88 126;

1035260 144 78 36;
1035328 128 78 64;
1035364 176 88 126;
1035364 176 88 45;
1035364 144 75 53;
1035416 176 88 45;
1035416 176 88 99;
1035416 144 78 51;
1035421 128 75 64;
1035494 176 88 99;
1035494 176 88 42;
1035494 144 75 70;
1035520 128 78 64;
1035645 128 75 64;
1035776 176 88 42;
1035776 176 88 46;
1035776 144 73 63;
1036244 176 88 46;
1036244 176 88 50;
1036244 144 75 72;
1036296 128 75 64;
1036307 128 73 64;
1036505 176 88 50;
1036505 176 88 102;
1036505 144 70 88;
1036510 176 88 102;
1036510 176 88 122;
1036510 144 75 91;
1036791 128 70 64;
1036822 128 75 64;
1036848 176 88 122;
1036848 176 88 41;
1036848 144 70 85;
1036864 176 88 41;
1036864 176 88 26;
1036864 144 75 79;
1037062 128 70 64;
1037166 128 75 64;
1037244 176 88 26;
1037244 176 88 15;
1037244 144 73 81;
1037244 176 88 15;
1037244 176 88 46;
1037244 144 78 78;
1037515 128 73 64;
1037552 128 78 64;
1037604 176 88 46;
1037604 176 88 14;
1037604 144 75 86;
1037604 176 88 14;
1037604 176 88 12;
1037604 144 80 78;
1037687 176 88 12;
1037687 176 88 50;
1037687 144 73 28;
1037848 128 73 64;
1037942 128 80 64;
1037947 128 75 64;
1038015 176 88 50;
1038015 176 88 8;
1038015 144 75 78;
1038140 176 88 8;
1038140 176 88 62;
1038140 144 80 23;
1038302 128 80 64;
1038338 128 75 64;
1038401 176 88 62;
1038401 176 88 113;
1038401 144 73 86;
1038406 176 88 113;
1038406 176 88 100;
1038406 144 78 84;
1038666 128 73 64;
1038666 128 78 64;
1038718 176 88 100;
1038718 176 88 113;
1038718 144 73 86;
1038729 176 88 113;
1038729 176 88 34;
1038729 144 78 81;
1039046 128 78 64;
1039046 128 73 64;
1039072 176 88 34;
1039072 176 88 103;
1039072 144 70 72;
1039078 176 88 103;
1039078 176 88 87;
1039078 144 75 65;
1040322 128 70 64;
1040354 128 75 64;
1040760 176 88 87;
1040760 176 88 125;
1040760 144 66 83;
1040765 176 88 125;
1040765 176 88 97;
1040765 144 78 87;
1041015 128 66 64;
1041015 128 78 64;
1041072 176 88 97;
1041072 176 88 17;
1041072 144 66 85;
1041083 176 88 17;
1041083 176 88 99;
1041083 144 75 79;
1041343 128 66 64;
1041395 128 75 64;
1041479 176 88 99;
1041479 176 88 39;
1041479 144 66 74;
1041479 176 88 39;
1041479 176 88 1;
1041479 144 73 76;
1041682 128 73 64;
1041718 128 66 64;
1041817 176 88 1;
1041817 176 88 122;
1041817 144 66 66;
1041822 176 88 122;
1041822 176 88 68;
1041822 144 70 90;
1042156 128 66 64;
1042213 176 88 68;
1042213 176 88 26;
1042213 128 70 64;
1042213 144 66 89;
1042218 176 88 26;
1042218 176 88 69;
1042218 144 73 91;
1042479 128 66 64;
1042541 128 73 64;
1042578 176 88 69;
1042578 176 88 39;
1042578 144 66 74;
1042583 176 88 39;
1042583 176 88 7;
1042583 144 75 82;
1042807 128 66 64;
1042828 128 75 64;
1042895 176 88 7;
1042895 176 88 41;
1042895 144 66 70;
1042895 144 78 91;
1044083 128 78 64;
1044114 128 66 64;
1044182 176 88 41;
1044182 176 88 32;
1044182 144 78 66;
1044187 176 88 32;
1044187 176 88 57;
1044187 144 66 66;
1044453 128 66 64;
1044479 128 78 64;
1044630 176 88 57;
1044630 176 88 74;
1044630 144 68 76;
1044635 176 88 74;
1044635 176 88 57;
1044635 144 80 86;
1044776 128 68 64;
1044828 128 80 64;
1044968 176 88 57;
1044968 176 88 29;
1044968 144 70 76;
1044973 176 88 29;
1044973 176 88 6;
1044973 144 82 89;
1045572 128 70 64;
1045843 176 88 6;
1045843 176 88 109;
1045843 144 73 78;
1045859 128 82 64;
1046057 176 88 109;
1046057 176 88 36;
1046057 144 85 87;
1046161 128 73 64;
1046401 176 88 36;
1046401 176 88 77;
1046401 144 82 28;
1046416 176 88 77;
1046416 176 88 105;
1046416 144 83 57;
1046463 128 85 64;
1046479 128 83 64;
1046500 128 82 64;
1046578 176 88 105;
1046578 176 88 49;
1046578 144 82 71;
1046859 176 88 49;
1046859 176 88 64;
1046859 144 80 57;
1046869 128 82 64;
1047286 176 88 64;
1047286 176 88 18;
1047286 144 78 62;
1047343 128 80 64;
1047651 128 78 64;
1047770 176 88 18;
1047770 176 88 15;
1047770 144 78 32;
1047859 128 78 64;
1047942 176 88 15;
1047942 176 88 12;
1047942 144 80 53;
1048114 176 88 12;
1048114 176 88 58;
1048114 144 78 70;
1048135 128 80 64;
1048234 176 88 58;
1048234 176 88 67;
1048234 144 80 45;
1048250 128 78 64;
1048312 128 80 64;
1048338 176 88 67;
1048338 176 88 88;
1048338 144 78 46;
1048401 128 78 64;
1048416 176 88 88;
1048416 176 88 46;
1048416 144 80 52;
1048552 176 88 46;
1048552 176 88 109;
1048552 144 78 39;
1048578 176 88 109;
1048578 176 88 63;
1048578 144 82 79;
1048614 128 80 64;
1048619 128 78 64;
1048880 176 88 63;
1048880 176 88 51;
1048880 144 78 69;
1048921 128 82 64;
1049098 176 88 51;
1049098 176 88 94;
1049098 144 87 90;
1049156 176 88 94;
1049156 176 88 86;
1049156 144 86 80;
1049234 128 87 64;
1049270 128 78 64;
1049281 128 86 64;
1049536 176 88 86;
1049536 176 88 100;
1049536 144 78 64;
1049536 144 87 84;
1049546 176 88 100;
1049546 176 88 35;
1049546 144 80 60;
1049598 128 80 64;
1049708 128 78 64;
1049739 128 87 64;
1049869 176 88 35;
1049869 176 88 71;
1049869 144 78 98;
1049880 176 88 71;
1049880 176 88 100;
1049880 144 87 84;
1050015 128 78 64;
1050041 128 87 64;
1050177 176 88 100;
1050177 176 88 4;
1050177 144 78 104;
1050182 176 88 4;
1050182 176 88 116;
1050182 144 87 94;
1050458 128 78 64;
1050484 128 87 64;
1050536 176 88 116;
1050536 176 88 69;
1050536 144 78 89;
1050536 176 88 69;
1050536 176 88 94;
1050536 144 87 90;
1050750 128 78 64;
1050786 128 87 64;
1050854 176 88 94;
1050854 176 88 3;
1050854 144 87 72;
1050859 176 88 3;
1050859 176 88 79;
1050859 144 78 76;
1051026 128 78 64;
1051088 128 87 64;
1051151 176 88 79;
1051151 176 88 32;
1051151 144 78 66;
1051156 176 88 32;
1051156 176 88 99;
1051156 144 85 79;
1051401 128 78 64;
1051505 176 88 99;
1051505 176 88 63;
1051505 144 90 79;
1051515 176 88 63;
1051515 176 88 16;
1051515 144 78 51;
1051609 128 85 64;
1052927 128 90 64;
1053015 128 78 64;
1053588 176 88 16;
1053588 176 88 25;
1053588 144 78 77;
1053604 176 88 25;
1053604 176 88 32;
1053604 144 82 69;
1053734 128 78 64;
1054020 176 88 32;
1054020 176 88 99;
1054020 144 78 77;
1054031 128 82 64;
1054171 128 78 64;
1054255 176 88 99;
1054255 176 88 87;
1054255 144 78 80;
1054281 176 88 87;
1054281 176 88 121;
1054281 144 85 84;
1054432 128 78 64;
1054593 176 88 121;
1054593 176 88 113;
1054593 144 78 54;
1054609 128 85 64;
1054718 128 78 64;
1054807 176 88 113;
1054807 176 88 99;
1054807 144 78 80;
1054807 144 87 99;
1054932 128 78 64;
1055119 128 87 64;
1055156 176 88 99;
1055156 176 88 33;
1055156 144 78 30;
1055250 128 78 64;
1055354 176 88 33;
1055354 176 88 12;
1055354 144 90 95;
1055375 176 88 12;
1055375 176 88 1;
1055375 144 78 83;
1055682 128 78 64;
1055703 128 90 64;
1055750 176 88 1;
1055750 176 88 87;

1055750 144 78 80;
1055750 176 88 87;
1055750 176 88 33;
1055755 144 90 93;
1056005 128 78 64;
1056010 128 90 64;
1056114 176 88 33;
1056114 176 88 72;
1056114 144 78 86;
1056125 176 88 72;
1056125 176 88 67;
1056125 144 90 65;
1056234 128 78 64;
1056265 128 90 64;
1056395 176 88 67;
1056395 176 88 77;
1056395 144 80 85;
1056395 144 92 91;
1056578 128 80 64;
1056588 128 92 64;
1056734 176 88 77;
1056734 176 88 116;
1056734 144 82 78;
1056739 176 88 116;
1056739 176 88 7;
1056739 144 94 90;
1056937 128 82 64;
1056947 128 94 64;
1057109 144 85 82;
1057114 176 88 7;
1057114 176 88 97;
1057114 144 97 87;
1058223 128 97 64;
1058260 128 85 64;
1059031 176 88 97;
1059031 176 88 62;
1059031 144 90 30;
1059114 128 90 64;
1059333 176 88 62;
1059333 176 88 48;
1059333 144 90 56;
1059557 176 88 48;
1059557 176 88 78;
1059557 144 92 56;
1059609 128 90 64;
1059838 128 92 64;
1059875 176 88 78;
1059875 176 88 46;
1059875 144 94 40;
1060062 176 88 46;
1060062 176 88 120;
1060062 144 92 68;
1060145 128 94 64;
1060348 128 92 64;
1060380 176 88 120;
1060380 176 88 122;
1060380 144 90 49;
1060484 128 90 64;
1061458 176 88 122;
1061458 176 88 110;
1061458 144 90 62;
1061630 176 88 110;
1061630 176 88 16;
1061630 144 92 72;
1061677 128 90 64;
1061947 128 92 64;
1061973 176 88 16;
1061973 176 88 72;
1061973 144 94 45;
1062151 176 88 72;
1062151 176 88 105;
1062151 144 92 66;
1062223 128 94 64;
1062468 176 88 105;
1062468 176 88 12;
1062468 128 92 64;
1062468 144 90 49;
1062869 128 90 64;
1063427 176 88 12;
1063427 176 88 35;
1063427 144 85 100;
1063427 176 88 35;
1063427 176 88 96;
1063427 144 80 101;
1065546 176 88 96;
1065546 176 88 120;
1065546 144 78 78;
1065557 128 80 64;
1065562 176 88 120;
1065562 176 88 93;
1065562 144 82 77;
1065583 128 85 64;
1067427 128 78 64;
1067505 128 82 64;
1067791 176 88 93;
1067791 176 88 50;
1067791 144 90 62;
1067973 176 88 50;
1067973 176 88 90;
1067973 144 92 51;
1068036 128 90 64;
1068276 176 88 90;
1068276 176 88 48;
1068276 144 94 45;
1068291 128 92 64;
1068432 176 88 48;
1068432 176 88 94;
1068432 144 92 60;
1068536 128 94 64;
1068750 176 88 94;
1068750 176 88 70;
1068750 144 90 76;
1068776 128 92 64;
1069661 128 90 64;
1069848 176 88 70;
1069848 176 88 20;
1069848 144 90 39;
1070020 176 88 20;
1070020 176 88 0;
1070020 144 92 70;
1070067 128 90 64;
1070364 176 88 0;
1070364 176 88 104;
1070364 144 94 44;
1070416 128 92 64;
1070567 176 88 104;
1070567 176 88 36;
1070567 144 97 76;
1070609 128 94 64;
1070864 176 88 36;
1070864 176 88 63;
1070864 144 99 79;
1070901 128 97 64;
1071255 128 99 64;
1071838 176 88 63;
1071838 176 88 94;
1071838 144 87 90;
1071843 176 88 94;
1071843 176 88 127;
1071843 144 80 102;
1071875 176 88 127;
1071875 176 88 104;
1071875 144 88 59;
1071947 128 88 64;
1072421 128 87 64;
1072453 128 80 64;
1072473 176 88 104;
1072473 176 88 127;
1072473 144 78 100;
1072479 176 88 127;
1072479 176 88 10;
1072479 144 85 99;
1073375 128 78 64;
1073734 128 85 64;
1073942 176 88 10;
1073942 176 88 81;
1073942 144 75 74;
1073947 176 88 81;
1073947 176 88 63;
1073947 144 82 79;
1074078 128 82 64;
1074083 128 75 64;
1074151 176 88 63;
1074151 176 88 116;
1074151 144 80 91;
1074161 176 88 116;
1074161 176 88 80;
1074161 144 73 88;
1074468 128 73 64;
1074484 128 80 64;
1074531 176 88 80;
1074531 176 88 54;
1074531 144 78 82;
1074536 176 88 54;
1074536 176 88 125;
1074536 144 73 49;
1074864 128 73 64;
1074911 128 78 64;
1074942 176 88 125;
1074942 176 88 50;
1074942 144 73 83;
1074958 176 88 50;
1074958 176 88 76;
1074958 144 78 101;
1075203 128 73 64;
1075239 176 88 76;
1075239 176 88 101;
1075239 144 73 89;
1075244 176 88 101;
1075244 176 88 98;
1075244 128 78 64;
1075244 144 78 55;
1075494 128 78 64;
1075520 128 73 64;
1075583 176 88 98;
1075583 176 88 86;
1075583 144 75 99;
1075598 176 88 86;
1075598 176 88 31;
1075598 144 73 45;
1075838 128 73 64;
1075921 176 88 31;
1075921 176 88 50;
1075921 144 73 83;
1075953 128 75 64;
1076244 176 88 50;
1076244 176 88 103;
1076244 144 75 69;
1076260 128 73 64;
1076312 128 75 64;
1076354 176 88 103;
1076354 176 88 18;
1076354 144 73 56;
1076473 176 88 18;
1076473 176 88 125;
1076473 144 75 36;
1076484 128 73 64;
1076541 128 75 64;
1076546 176 88 125;
1076546 176 88 49;
1076546 144 73 48;
1076604 176 88 49;
1076604 176 88 45;
1076604 144 75 50;
1076619 128 73 64;
1076666 128 75 64;
1076723 176 88 45;
1076723 176 88 72;
1076723 144 73 50;
1076770 176 88 72;
1076770 176 88 21;
1076770 144 75 54;
1076848 128 73 64;
1078666 128 75 64;
1078697 176 88 21;
1078697 176 88 100;
1078697 144 73 45;
1078880 176 88 100;
1078880 176 88 102;
1078880 128 73 64;
1078880 144 70 76;
1079234 176 88 102;
1079234 176 88 57;
1079234 144 68 60;
1079317 128 70 64;
1079432 176 88 57;
1079432 176 88 92;
1079432 144 66 77;
1079479 128 68 64;
1079750 176 88 92;
1079750 176 88 115;
1079750 144 68 52;
1079781 128 66 64;
1079942 176 88 115;
1079942 176 88 30;
1079942 144 70 69;
1080052 128 68 64;
1080239 128 70 64;
1080250 176 88 30;
1080250 176 88 64;
1080250 144 75 73;
1081291 176 88 64;
1081291 176 88 103;
1081291 144 70 72;
1081302 176 88 103;
1081302 176 88 0;
1081302 144 78 58;
1081333 128 75 64;
1082067 128 70 64;
1082260 176 88 0;
1082260 176 88 126;
1082260 144 73 79;
1082270 176 88 126;
1082270 176 88 98;
1082270 144 80 72;
1082369 128 78 64;
1083041 128 80 64;
1083041 128 73 64;
1083317 176 88 98;
1083317 176 88 4;
1083317 144 82 83;
1083328 176 88 4;
1083328 176 88 64;
1083328 144 75 73;
1083718 128 75 64;
1083765 128 82 64;
1083812 176 88 64;
1083812 176 88 62;
1083812 144 73 61;
1083838 176 88 62;
1083838 176 88 28;
1083838 144 80 61;
1084088 128 73 64;
1084109 128 80 64;
1084156 176 88 28;
1084156 176 88 74;
1084156 144 78 83;
1084489 128 78 64;
1084536 176 88 74;
1084536 176 88 102;
1084536 144 68 78;
1084546 176 88 102;
1084546 176 88 34;
1084546 144 78 81;
1085416 128 68 64;
1085515 176 88 34;
1085515 176 88 12;
1085515 144 66 72;
1085520 176 88 12;
1085520 176 88 20;
1085520 144 75 65;
1085583 128 78 64;
1087005 128 75 64;
1087020 128 66 64;
1087354 176 88 20;
1087354 176 88 92;
1087354 144 66 44;
1087489 128 66 64;
1087661 176 88 92;
1087661 176 88 57;
1087661 144 66 66;
1087807 176 88 57;
1087807 176 88 29;
1087807 128 66 64;
1087807 144 68 62;
1088046 128 68 64;
1088140 176 88 29;
1088140 176 88 32;
1088140 144 70 36;
1088244 128 70 64;
1088317 176 88 32;
1088317 176 88 74;
1088317 144 73 76;
1088666 128 73 64;
1088697 176 88 74;
1088697 176 88 93;
1088697 144 70 32;
1089807 176 88 93;
1089807 176 88 6;
1089807 144 68 45;
1089817 128 70 64;
1090177 128 68 64;

1090218 176 88 6;
1090218 176 88 53;
1090218 144 70 34;
1090307 128 70 64;
1090348 176 88 53;
1090348 176 88 62;
1090348 144 68 35;
1090416 176 88 62;
1090416 176 88 47;
1090416 144 70 51;
1090421 128 68 64;
1090515 176 88 47;
1090515 176 88 113;
1090515 144 68 27;
1090593 128 68 64;
1090692 176 88 113;
1090692 176 88 49;
1090692 144 68 45;
1090739 128 70 64;
1090890 176 88 49;
1090890 176 88 57;
1090890 144 66 66;
1090906 128 68 64;
1091208 128 66 64;
1091255 176 88 57;
1091255 176 88 91;
1091255 144 63 32;
1091421 176 88 91;
1091421 176 88 23;
1091421 144 61 47;
1091447 128 63 64;
1091739 128 61 64;
1091781 176 88 23;
1091781 176 88 0;
1091781 144 63 36;
1091885 128 63 64;
1091947 176 88 0;
1091947 176 88 111;
1091947 144 66 63;
1092286 176 88 111;
1092286 176 88 93;
1092286 144 68 42;
1092333 128 66 64;
1092453 176 88 93;
1092453 176 88 67;
1092453 144 70 55;
1092552 128 68 64;
1092786 128 70 64;
1092994 176 88 67;
1092994 176 88 115;
1092994 144 66 64;
1093000 176 88 115;
1093000 176 88 50;
1093000 144 73 64;
1093531 128 66 64;
1094088 176 88 50;
1094088 176 88 111;
1094088 144 66 63;
1094098 128 73 64;
1094104 176 88 111;
1094104 176 88 104;
1094104 144 70 60;
1094151 128 66 64;
1094166 128 70 64;
1094520 176 88 104;
1094520 176 88 41;
1094520 144 70 85;
1094531 176 88 41;
1094531 176 88 44;
1094531 144 66 58;
1094578 128 70 64;
1094583 128 66 64;
1094854 176 88 44;
1094854 176 88 7;
1094854 144 70 87;
1094859 176 88 7;
1094859 176 88 115;
1094859 144 66 64;
1094911 128 70 64;
1094911 128 66 64;
1095286 176 88 115;
1095286 176 88 89;
1095286 144 63 79;
1095286 144 68 82;
1095338 128 63 64;
1095343 128 68 64;
1095635 176 88 89;
1095635 176 88 49;
1095635 144 61 86;
1095640 176 88 49;
1095640 176 88 89;
1095640 144 66 81;
1095750 176 88 89;
1095750 176 88 84;
1095750 128 66 64;
1095750 144 66 11;
1095781 128 61 64;
1095822 128 66 64;
1096067 176 88 84;
1096067 176 88 50;
1096067 144 63 84;
1096078 176 88 50;
1096078 176 88 127;
1096078 144 58 74;
1096328 128 63 64;
1096333 128 58 64;
1096473 176 88 127;
1096473 176 88 62;
1096473 144 56 81;
1096479 176 88 62;
1096479 176 88 60;
1096479 144 61 73;
1096692 128 56 64;
1096723 128 61 64;
1096843 176 88 60;
1096843 176 88 1;
1096843 144 54 76;
1096848 176 88 1;
1096848 176 88 76;
1096848 144 58 71;
1097083 128 54 64;
1097140 128 58 64;
1097192 176 88 76;
1097192 176 88 90;
1097192 144 56 86;
1097197 176 88 90;
1097197 176 88 111;
1097197 144 61 72;
1097453 128 56 64;
1097500 128 61 64;
1097583 176 88 111;
1097583 176 88 12;
1097583 144 58 79;
1097588 176 88 12;
1097588 176 88 21;
1097588 144 63 76;
1097875 128 58 64;
1097921 128 63 64;
1097989 176 88 21;
1097989 176 88 26;
1097989 144 61 85;
1097989 144 66 89;
1099510 128 61 64;
1099572 128 66 64;
1099604 176 88 26;
1099604 176 88 18;
1099604 144 61 78;
1099604 176 88 18;
1099604 176 88 28;
1099604 144 70 88;
1099869 128 61 64;
1099885 128 70 64;
1099953 176 88 28;
1099953 176 88 98;
1099953 144 61 88;
1099958 176 88 98;
1099958 176 88 88;
1099958 144 70 91;
1100145 128 61 64;
1100161 128 70 64;
1100270 176 88 88;
1100270 176 88 98;
1100270 144 61 94;
1100276 176 88 98;
1100276 176 88 41;
1100276 144 70 85;
1100442 128 61 64;
1100458 128 70 64;
1100562 176 88 41;
1100562 176 88 101;
1100562 144 58 95;
1100562 144 68 87;
1100682 128 58 64;
1100708 128 68 64;
1100822 176 88 101;
1100822 176 88 59;
1100822 144 56 101;
1100828 176 88 59;
1100828 176 88 110;
1100828 144 66 93;
1100953 128 56 64;
1100968 128 66 64;
1101098 176 88 110;
1101098 176 88 79;
1101098 144 54 98;
1101104 176 88 79;
1101104 176 88 119;
1101104 144 63 77;
1101250 128 63 64;
1101260 128 54 64;
1101463 176 88 119;
1101463 176 88 96;
1101463 144 51 104;
1101463 176 88 96;
1101463 176 88 21;
1101463 144 61 91;
1102536 128 61 64;
1102557 128 51 64;
1102697 176 88 21;
1102697 176 88 103;
1102697 144 49 96;
1102713 176 88 103;
1102713 176 88 120;
1102713 144 58 68;
1102729 176 88 120;
1102729 176 88 119;
1102729 144 60 68;
1102890 128 60 64;
1102932 128 58 64;
1103041 128 49 64;
1103109 176 88 119;
1103109 176 88 92;
1103109 144 49 93;
1103140 176 88 92;
1103140 176 88 99;
1103140 144 56 68;
1103161 176 88 99;
1103161 176 88 125;
1103161 144 58 47;
1103328 128 56 64;
1103343 128 49 64;
1103343 128 58 64;
1103427 176 88 125;
1103427 176 88 112;
1103427 144 49 88;
1103432 176 88 112;
1103432 176 88 52;
1103432 144 58 68;
1104062 128 58 64;
1104140 128 49 64;
1104515 176 88 52;
1104515 176 88 43;
1104515 144 51 87;
1104520 144 44 87;
1104890 128 44 64;
1105010 176 88 43;
1105010 176 88 26;
1105010 144 44 71;
1105010 176 88 26;
1105010 176 88 89;
1105010 144 49 78;
1105088 128 51 64;
1105468 128 44 64;
1105515 128 49 64;
1105541 176 88 89;
1105541 176 88 43;
1105541 144 46 85;
1106640 176 88 43;
1106640 176 88 37;
1106640 144 44 47;
1106729 128 46 64;
1107666 176 88 37;
1107666 176 88 91;
1107666 144 42 45;
1107671 128 44 64;
1108526 128 42 64;
1108557 176 88 91;
1108557 176 88 78;
1108557 144 46 37;
1108562 176 88 78;
1108562 176 88 11;
1108562 144 39 33;
1110781 128 39 64;
1111671 128 46 64;
1206317 176 88 11;
1206317 176 88 17;
1206317 144 51 32;
1206994 176 88 17;
1206994 176 88 32;
1206994 144 54 27;
1207057 128 51 64;
1207796 176 88 32;
1207796 176 88 47;
1207796 144 55 37;
1207802 128 54 64;
1208098 128 55 64;
1208432 176 88 47;
1208432 176 88 52;
1208432 144 51 35;
1208640 128 51 64;
1208645 176 88 52;
1208645 176 88 28;
1208645 144 54 37;
1208901 176 88 28;
1208901 176 88 83;
1208901 144 56 39;
1208947 128 54 64;
1209223 128 56 64;
1209223 176 88 83;
1209223 176 88 88;
1209223 144 57 40;
1210067 176 88 88;
1210067 176 88 40;
1210067 144 58 38;
1210109 128 57 64;
1210536 176 88 40;
1210536 176 88 127;
1210536 144 61 38;
1210583 128 58 64;
1210984 176 88 127;
1210984 176 88 25;
1210984 128 61 64;
1210984 144 63 31;
1211208 176 88 25;
1211208 176 88 21;
1211208 144 61 40;
1211229 128 63 64;
1211432 176 88 21;
1211432 176 88 2;
1211432 144 58 39;
1211458 128 61 64;
1211609 128 58 64;
1211625 176 88 2;
1211625 176 88 105;
1211625 144 57 40;
1211864 128 57 64;
1211869 176 88 105;
1211869 176 88 126;
1211869 144 56 37;
1212171 176 88 126;
1212171 176 88 124;
1212171 144 54 33;
1212177 128 56 64;
1212510 128 54 64;
1212536 176 88 124;
1212536 176 88 105;
1212536 144 51 34;
1212921 128 51 64;
1217364 176 88 105;
1217364 176 88 85;
1217364 144 62 28;
1217510 128 62 64;
1218088 176 88 85;
1218088 176 88 104;
1218088 144 57 24;
1218098 176 88 104;
1218098 176 88 96;
1218098 144 62 19;
1218593 128 57 64;
1218593 128 62 64;
1244489 176 88 96;
1244489 176 88 92;

1244489 144 51 27;
1244723 128 51 64;
1244755 176 88 92;
1244755 176 88 96;
1244755 144 54 33;
1245015 128 54 64;
1245046 176 88 96;
1245046 176 88 47;
1245046 144 55 44;
1245291 128 55 64;
1245338 176 88 47;
1245338 176 88 35;
1245338 144 56 28;
1245421 128 56 64;
1245916 176 88 35;
1245916 176 88 64;
1245916 144 51 37;
1246088 128 51 64;
1246145 176 88 64;
1246145 176 88 50;
1246145 144 54 28;
1246348 176 88 50;
1246348 176 88 40;
1246348 144 56 33;
1246375 128 54 64;
1246593 128 56 64;
1246614 176 88 40;
1246614 176 88 61;
1246614 144 57 43;
1247593 128 57 64;
1248406 176 88 61;
1248406 176 88 96;
1248406 144 51 37;
1248598 128 51 64;
1248630 176 88 96;
1248630 176 88 39;
1248630 144 54 32;
1248843 176 88 39;
1248843 176 88 84;
1248843 128 54 64;
1248843 144 56 35;
1249046 128 56 64;
1249052 176 88 84;
1249052 176 88 120;
1249052 144 57 52;
1249286 176 88 120;
1249286 176 88 27;
1249286 144 58 46;
1249296 128 57 64;
1249562 176 88 27;
1249562 176 88 21;
1249562 144 61 40;
1249567 128 58 64;
1250442 128 61 64;
1250453 176 88 21;
1250453 176 88 57;
1250453 144 62 43;
1250822 176 88 57;
1250822 176 88 59;
1250822 144 63 33;
1250833 128 62 64;
1252041 128 63 64;
1252401 176 88 59;
1252401 176 88 41;
1252401 144 62 54;
1252406 176 88 41;
1252406 176 88 118;
1252406 144 57 50;
1252468 128 62 64;
1252473 128 57 64;
1252625 176 88 118;
1252625 176 88 77;
1252625 144 57 51;
1252625 144 62 60;
1252723 128 57 64;
1252723 128 62 64;
1252875 176 88 77;
1252875 176 88 105;
1252875 144 62 76;
1252875 176 88 105;
1252875 176 88 116;
1252875 144 57 66;
1252942 128 57 64;
1252958 128 62 64;
1414187 176 88 116;
1414187 176 88 122;
1414187 144 69 28;
1414848 128 69 64;
1417088 176 88 122;
1417088 176 88 116;
1417088 144 69 23;
1417776 128 69 64;
1426427 176 88 116;
1426427 176 88 8;
1426427 144 63 15;
1426859 128 63 64;
1426880 176 88 8;
1426880 176 88 24;
1426880 144 66 21;
1427244 128 66 64;
1427265 176 88 24;
1427265 176 88 104;
1427265 144 68 23;
1427682 128 68 64;
1427687 176 88 104;
1427687 176 88 41;
1427687 144 69 28;
1428161 128 69 64;
1428239 176 88 41;
1428239 176 88 98;
1428239 144 70 26;
1429421 128 70 64;
1429437 176 88 98;
1429437 176 88 46;
1429437 144 73 18;
1429937 128 73 64;
1430000 176 88 46;
1430000 176 88 17;
1430000 144 74 24;
1430458 128 74 64;
1431416 176 88 17;
1431416 176 88 63;
1431416 144 75 14;
1431505 128 75 64;
1433114 176 88 63;
1433114 176 88 122;
1433114 144 73 22;
1433286 128 73 64;
1433317 176 88 122;
1433317 176 88 84;
1433317 144 75 27;
1433583 176 88 84;
1433583 176 88 61;
1433583 144 73 29;
1433588 128 75 64;
1433901 128 73 64;
1433973 176 88 61;
1433973 176 88 121;
1433973 144 69 25;
1434171 128 69 64;
1434536 176 88 121;
1434536 176 88 78;
1434536 144 68 17;
1434848 128 68 64;
1434895 176 88 78;
1434895 176 88 48;
1434895 144 66 25;
1434979 128 66 64;
1434984 176 88 48;
1434984 176 88 90;
1434984 144 68 35;
1435083 176 88 90;
1435083 176 88 21;
1435083 144 66 27;
1435088 128 68 64;
1435161 128 66 64;
1435229 176 88 21;
1435229 176 88 40;
1435229 144 63 12;
1435286 128 63 64;
1447171 176 88 40;
1447171 176 88 45;
1447171 144 69 18;
1447479 128 69 64;
1448171 176 88 45;
1448171 176 88 10;
1448171 144 69 30;
1448208 176 88 10;
1448208 176 88 115;
1448208 144 63 24;
1448432 128 69 64;
1448453 128 63 64;
1449562 176 88 115;
1449562 176 88 116;
1449562 144 75 28;
1449562 176 88 116;
1449562 176 88 44;
1449562 144 69 32;
1450213 128 69 64;
1450296 128 75 64;
1450390 176 88 44;
1450390 176 88 72;
1450390 144 69 44;
1450588 176 88 72;
1450588 176 88 73;
1450588 128 69 64;
1450588 144 75 42;
1450781 176 88 73;
1450781 176 88 9;
1450781 128 75 64;
1450781 144 75 6;
1450880 128 75 64;
1450953 176 88 9;
1450953 176 88 85;
1450953 144 69 25;
1451072 176 88 85;
1451072 176 88 114;
1451072 128 69 64;
1451072 144 74 37;
1451145 128 74 64;
1451177 176 88 114;
1451177 176 88 111;
1451177 144 69 44;
1451265 128 69 64;
1451276 176 88 111;
1451276 176 88 113;
1451276 144 73 48;
1451343 128 73 64;
1451401 176 88 113;
1451401 176 88 82;
1451401 144 69 42;
1451625 176 88 82;
1451625 176 88 21;
1451625 144 70 50;
1451645 128 69 64;
1451687 128 70 64;
1451895 176 88 21;
1451895 176 88 72;
1451895 144 69 44;
1452020 128 69 64;
1452104 176 88 72;
1452104 176 88 53;
1452104 144 70 13;
1452156 176 88 53;
1452156 176 88 125;
1452156 144 69 47;
1452161 128 70 64;
1452229 128 69 64;
1452250 176 88 125;
1452250 176 88 17;
1452250 144 68 46;
1452333 128 68 64;
1452463 176 88 17;
1452463 176 88 60;
1452463 144 66 37;
1452671 128 66 64;
1452729 176 88 60;
1452729 176 88 68;
1452729 144 68 30;
1452812 128 68 64;
1452921 176 88 68;
1452921 176 88 82;
1452921 144 68 36;
1453015 128 68 64;
1453093 176 88 82;
1453093 176 88 65;
1453093 144 63 40;
1453270 128 63 64;
1453281 176 88 65;
1453281 176 88 39;
1453281 144 66 35;
1453411 128 66 64;
1453442 176 88 39;
1453442 176 88 69;
1453442 144 68 27;
1453531 128 68 64;
1453588 176 88 69;
1453588 176 88 109;
1453588 144 69 26;
1453692 128 69 64;
1453697 176 88 109;
1453697 176 88 42;
1453697 144 70 46;
1453776 128 70 64;
1453927 176 88 42;
1453927 176 88 8;
1453927 144 73 39;
1454000 128 73 64;
1454510 176 88 8;
1454510 176 88 85;
1454510 144 74 50;
1454567 128 74 64;
1454630 176 88 85;
1454630 176 88 47;
1454635 144 74 68;
1454697 128 74 64;
1458218 176 88 47;
1458218 176 88 9;
1458218 144 63 22;
1458229 176 88 9;
1458229 176 88 102;
1458229 144 74 33;
1458390 128 74 64;
1458416 128 63 64;
1458697 176 88 102;
1458697 176 88 27;
1458697 144 75 31;
1458859 128 75 64;
1477385 176 88 27;
1477385 176 88 96;
1477385 144 63 39;
1477697 128 63 64;
1477713 176 88 96;
1477713 176 88 0;
1477713 144 66 36;
1477875 176 88 0;
1477875 176 88 112;
1477875 144 68 44;
1477906 128 66 64;
1478197 128 68 64;
1478208 176 88 112;
1478208 176 88 23;
1478208 144 69 45;
1478682 128 69 64;
1478703 176 88 23;
1478703 176 88 46;
1478703 144 68 26;
1479234 176 88 46;
1479234 176 88 67;
1479234 144 66 43;
1479296 128 68 64;
1480411 128 66 64;
1480458 176 88 67;
1480458 176 88 35;
1480458 144 63 22;
1480625 176 88 35;
1480625 176 88 80;
1480625 144 66 39;
1480640 128 63 64;
1480984 128 66 64;
1480994 176 88 80;
1480994 176 88 116;
1480994 144 63 27;
1481171 176 88 116;
1481171 176 88 31;
1481171 144 66 45;
1481192 128 63 64;
1481458 128 66 64;
1481520 176 88 31;
1481520 176 88 46;
1481520 144 66 63;
1482010 176 88 46;
1482010 176 88 40;
1482010 144 63 39;
1482062 128 66 64;
1482406 128 63 64;
1483822 176 88 40;
1483822 176 88 45;
1483822 144 75 39;
1483859 176 88 45;
1483859 176 88 50;
1483859 144 78 44;

1483890 128 75 64;
1483895 176 88 50;
1483895 176 88 87;
1483895 144 80 50;
1483916 128 78 64;
1483947 128 80 64;
1483953 176 88 87;
1483953 176 88 46;
1483953 144 82 40;
1484015 128 82 64;
1484286 176 88 46;
1484286 176 88 108;
1484286 144 82 81;
1484302 176 88 108;
1484302 176 88 48;
1484302 144 75 40;
1484359 128 82 64;
1484364 128 75 64;
1484687 176 88 48;
1484687 176 88 103;
1484687 144 75 69;
1484692 176 88 103;
1484692 176 88 109;
1484692 144 82 70;
1484739 128 75 64;
1484739 128 82 64;
1485020 176 88 109;
1485020 176 88 118;
1485020 144 75 80;
1485020 176 88 118;
1485020 176 88 108;
1485020 144 82 81;
1485072 128 75 64;
1485088 128 82 64;
1485354 176 88 108;
1485354 176 88 118;
1485354 144 75 80;
1485359 176 88 118;
1485359 176 88 79;
1485359 144 81 81;
1485437 128 75 64;
1485468 128 81 64;
1485697 176 88 79;
1485697 176 88 81;
1485697 144 80 84;
1485713 176 88 81;
1485713 176 88 56;
1485713 144 75 59;
1485765 128 75 64;
1485776 128 80 64;
1486057 176 88 56;
1486057 176 88 64;
1486057 144 75 73;
1487890 128 75 64;
1488197 176 88 64;
1488197 176 88 106;
1488197 144 75 40;
1488317 128 75 64;
1488375 176 88 106;
1488375 176 88 38;
1488375 144 78 45;
1488515 128 78 64;
1488520 176 88 38;
1488520 176 88 69;
1488520 144 80 43;
1488640 128 80 64;
1488656 176 88 69;
1488656 176 88 91;
1488656 144 81 59;
1488729 128 81 64;
1488838 176 88 91;
1488838 176 88 48;
1488838 144 82 56;
1488932 128 82 64;
1489265 176 88 48;
1489265 176 88 103;
1489265 144 85 69;
1489619 128 85 64;
1489625 176 88 103;
1489625 176 88 99;
1489625 144 87 51;
1489776 176 88 99;
1489776 176 88 90;
1489776 144 85 86;
1489807 128 87 64;
1490098 128 85 64;
1490130 176 88 90;
1490130 176 88 48;
1490130 144 87 85;
1491135 128 87 64;
1491380 176 88 48;
1491380 176 88 109;
1491380 144 86 48;
1491468 128 86 64;
1491489 176 88 109;
1491489 176 88 66;
1491489 144 87 79;
1491562 128 87 64;
1492369 176 88 66;
1492369 176 88 104;
1492369 144 74 68;
1492515 176 88 104;
1492515 176 88 111;
1492515 144 75 87;
1492546 128 74 64;
1492671 128 75 64;
1493510 176 88 111;
1493510 176 88 117;
1493510 144 62 55;
1493656 176 88 117;
1493656 176 88 78;
1493656 144 63 56;
1493661 128 62 64;
1493942 128 63 64;
1493979 176 88 78;
1493979 176 88 0;
1493979 144 63 70;
1494145 128 63 64;
1494406 176 88 0;
1494406 176 88 120;
1494406 144 63 68;
1495437 128 63 64;
1495546 176 88 120;
1495546 176 88 105;
1495546 144 63 28;
1495723 128 63 64;
1495744 176 88 105;
1495744 176 88 0;
1495744 144 62 47;
1496000 128 62 64;
1496072 176 88 0;
1496072 176 88 98;
1496072 144 61 37;
1496604 128 61 64;
1496625 176 88 98;
1496625 176 88 108;
1496625 144 58 46;
1497151 128 58 64;
1497171 176 88 108;
1497171 176 88 80;
1497171 144 57 47;
1497364 176 88 80;
1497364 176 88 42;
1497364 144 56 70;
1497395 128 57 64;
1497729 176 88 42;
1497729 176 88 14;
1497729 144 54 41;
1497750 128 56 64;
1497906 176 88 14;
1497906 176 88 60;
1497906 144 51 81;
1497921 128 54 64;
1498255 176 88 60;
1498255 176 88 92;
1498255 144 54 55;
1498312 128 51 64;
1498421 176 88 92;
1498421 176 88 46;
1498421 144 51 63;
1498505 128 54 64;
1498755 128 51 64;
1498786 176 88 46;
1498786 176 88 18;
1498786 144 54 77;
1499010 176 88 18;
1499010 176 88 6;
1499010 144 51 82;
1499062 128 54 64;
1501197 128 51 64;
1501755 176 88 6;
1501755 176 88 19;
1501755 144 50 53;
1501916 176 88 19;
1501916 176 88 7;
1501916 144 51 41;
1501927 128 50 64;
1502041 176 88 7;
1502041 176 88 80;
1502041 144 54 39;
1502057 128 51 64;
1502104 128 54 64;
1502203 176 88 80;
1502203 176 88 44;
1502203 144 56 80;
1502614 176 88 44;
1502614 176 88 95;
1502614 144 54 73;
1502666 128 56 64;
1503046 128 54 64;
1503098 176 88 95;
1503098 176 88 100;
1503098 144 51 83;
1504125 128 51 64;
1504244 176 88 100;
1504244 176 88 63;
1504244 144 51 42;
1504364 128 51 64;
1504416 176 88 63;
1504416 176 88 38;
1504416 144 51 99;
1504416 144 63 88;
1504677 128 51 64;
1504750 176 88 38;
1504750 176 88 21;
1504750 144 51 70;
1504911 128 63 64;
1504979 176 88 21;
1504979 176 88 30;
1504979 144 61 82;
1505114 128 51 64;
1505307 176 88 30;
1505307 176 88 29;
1505307 144 51 49;
1505458 128 61 64;
1505463 128 51 64;
1505515 176 88 29;
1505515 176 88 100;
1505515 144 51 83;
1505526 176 88 100;
1505526 176 88 68;
1505526 144 58 70;
1505859 128 51 64;
1506041 176 88 68;
1506041 176 88 6;
1506041 144 51 31;
1506213 128 51 64;
1506291 128 58 64;
1506921 176 88 6;
1506921 176 88 96;
1506921 144 63 36;
1507000 128 63 64;
1507067 176 88 96;
1507067 176 88 119;
1507067 144 63 77;
1507078 176 88 119;
1507078 176 88 68;
1507078 144 75 85;
1507265 128 63 64;
1507291 128 75 64;
1507432 176 88 68;
1507432 176 88 42;
1507432 144 63 84;
1507432 144 75 70;
1508390 128 63 64;
1508437 128 75 64;
1508572 176 88 42;
1508572 176 88 65;
1508572 144 63 47;
1508645 128 63 64;
1508718 176 88 65;
1508718 176 88 26;
1508718 144 75 79;
1508723 176 88 26;
1508723 176 88 55;
1508723 144 63 58;
1508895 128 63 64;
1509062 176 88 55;
1509062 176 88 8;
1509062 144 63 47;
1509151 128 75 64;
1509182 128 63 64;
1509244 176 88 8;
1509244 176 88 63;
1509244 144 73 68;
1509255 176 88 63;
1509255 176 88 124;
1509255 144 63 57;
1509421 128 63 64;
1509598 176 88 124;
1509598 176 88 20;
1509598 144 63 39;
1509765 128 73 64;
1509796 176 88 20;
1509796 176 88 80;
1509796 128 63 64;
1509796 144 63 36;
1509807 176 88 80;
1509807 176 88 12;
1509807 144 70 63;
1510692 128 63 64;
1510755 128 70 64;
1510895 176 88 12;
1510895 176 88 9;
1510895 144 63 57;
1510895 144 70 53;
1511125 128 63 64;
1511135 128 70 64;
1511213 176 88 9;
1511213 176 88 111;
1511213 144 63 44;
1511223 176 88 111;
1511223 176 88 44;
1511223 144 69 66;
1511505 128 63 64;
1511598 128 69 64;
1511604 176 88 44;
1511604 176 88 108;
1511604 144 63 46;
1511625 176 88 108;
1511625 176 88 52;
1511625 144 68 57;
1511843 128 68 64;
1511895 128 63 64;
1511973 176 88 52;
1511973 176 88 15;
1511973 144 63 43;
1511989 176 88 15;
1511989 176 88 75;
1511989 144 66 53;
1512187 128 63 64;
1512286 176 88 75;
1512286 176 88 67;
1512286 144 63 53;
1512593 128 66 64;
1513114 128 63 64;
1513411 176 88 67;
1513411 176 88 60;
1513411 144 75 45;
1513572 176 88 60;
1513572 176 88 32;
1513572 144 87 66;
1513776 128 75 64;
1513927 176 88 32;
1513927 176 88 30;
1513927 144 75 40;
1514020 128 87 64;
1514145 176 88 30;
1514145 176 88 22;
1514145 144 86 76;
1514291 128 75 64;
1514453 128 86 64;
1514468 176 88 22;
1514468 176 88 115;
1514468 144 75 55;
1514645 176 88 115;
1514645 176 88 118;
1514645 144 85 80;
1514723 128 75 64;
1515026 176 88 118;
1515026 176 88 92;
1515026 144 75 28;

1515244 176 88 92;
1515244 176 88 25;
1515244 144 82 58;
1515265 128 75 64;
1515286 128 85 64;
1515572 128 82 64;
1515572 176 88 25;
1515572 176 88 86;
1515572 144 81 58;
1515807 176 88 86;
1515807 176 88 19;
1515807 144 80 75;
1515843 128 81 64;
1516161 176 88 19;
1516161 176 88 25;
1516161 144 78 65;
1516182 128 80 64;
1516604 176 88 25;
1516604 176 88 111;
1516604 144 75 71;
1516645 128 78 64;
1517505 128 75 64;
1517682 176 88 111;
1517682 176 88 17;
1517682 144 75 44;
1517682 144 80 56;
1517739 128 80 64;
1517744 128 75 64;
1517848 176 88 17;
1517848 176 88 0;
1517848 144 80 52;
1517859 176 88 0;
1517859 176 88 89;
1517859 144 75 61;
1517921 128 75 64;
1517973 128 80 64;
1518203 176 88 89;
1518203 176 88 36;
1518203 144 75 87;
1518208 176 88 36;
1518208 176 88 33;
1518208 144 80 88;
1519078 128 75 64;
1519255 128 80 64;
1519312 176 88 33;
1519312 176 88 62;
1519312 144 75 81;
1519572 176 88 62;
1519572 176 88 54;
1519572 144 80 58;
1519588 128 75 64;
1519630 128 80 64;
1519682 176 88 54;
1519682 176 88 34;
1519682 144 75 51;
1519755 128 75 64;
1519848 176 88 34;
1519848 176 88 122;
1519848 144 75 50;
1519911 176 88 122;
1519911 176 88 84;
1519911 144 80 51;
1519916 128 75 64;
1519963 128 80 64;
1520010 176 88 84;
1520010 176 88 76;
1520010 144 75 54;
1520062 176 88 76;
1520062 176 88 43;
1520062 144 80 23;
1520078 128 75 64;
1520145 128 80 64;
1520156 176 88 43;
1520156 176 88 37;
1520156 144 75 69;
1520416 176 88 37;
1520416 176 88 81;
1520416 144 80 84;
1520463 128 75 64;
1520630 176 88 81;
1520630 176 88 15;
1520630 144 78 73;
1520656 128 80 64;
1520911 176 88 15;
1520911 176 88 99;
1520911 144 75 75;
1520927 128 78 64;
1522041 176 88 99;
1522041 176 88 35;
1522041 144 73 50;
1522046 128 75 64;
1522213 176 88 35;
1522213 176 88 91;
1522213 144 75 66;
1522223 128 73 64;
1522343 128 75 64;
1522359 176 88 91;
1522359 176 88 67;
1522359 144 73 43;
1522421 128 73 64;
1522437 176 88 67;
1522437 176 88 127;
1522437 144 75 58;
1522531 128 75 64;
1522536 176 88 127;
1522536 176 88 88;
1522536 144 73 43;
1522614 176 88 88;
1522614 176 88 0;
1522614 144 75 36;
1522687 128 75 64;
1522750 176 88 0;
1522750 176 88 68;
1522750 144 70 74;
1522765 128 73 64;
1523062 176 88 68;
1523062 176 88 111;
1523062 144 69 76;
1523078 128 70 64;
1523416 128 69 64;
1523442 176 88 111;
1523442 176 88 44;
1523442 144 68 61;
1523598 128 68 64;
1523739 176 88 44;
1523739 176 88 89;
1523739 144 66 81;
1524109 128 66 64;
1524135 176 88 89;
1524135 176 88 60;
1524135 144 63 69;
1524442 128 63 64;
1524489 176 88 60;
1524489 176 88 21;
1524489 144 66 73;
1524864 176 88 21;
1524864 176 88 8;
1524864 144 63 71;
1524895 128 66 64;
1525302 176 88 8;
1525302 176 88 37;
1525302 144 75 69;
1526625 128 75 64;
1526869 128 63 64;
1526958 176 88 37;
1526958 176 88 20;
1526958 144 63 56;
1527098 176 88 20;
1527098 176 88 74;
1527098 144 73 76;
1527140 128 63 64;
1527458 176 88 74;
1527458 176 88 40;
1527458 144 63 38;
1527463 128 73 64;
1527651 176 88 40;
1527651 176 88 46;
1527651 144 70 77;
1527739 128 63 64;
1528015 176 88 46;
1528015 176 88 5;
1528015 144 63 38;
1528057 128 70 64;
1528109 128 63 64;
1528197 176 88 5;
1528197 176 88 81;
1528197 144 75 82;
1528203 176 88 81;
1528203 176 88 21;
1528203 144 63 76;
1528312 128 63 64;
1528369 128 75 64;
1528640 176 88 21;
1528640 176 88 121;
1528640 144 75 84;
1528645 176 88 121;
1528645 176 88 48;
1528645 144 87 85;
1528692 128 75 64;
1528703 128 87 64;
1529067 176 88 48;
1529067 176 88 91;
1529067 144 75 93;
1529072 176 88 91;
1529072 176 88 1;
1529072 144 87 83;
1529833 128 87 64;
1529875 128 75 64;
1530114 176 88 1;
1530114 176 88 43;
1530114 144 75 60;
1530114 176 88 43;
1530114 176 88 118;
1530114 144 87 69;
1530166 128 75 64;
1530171 128 87 64;
1530296 176 88 118;
1530296 176 88 19;
1530296 144 78 64;
1530296 144 90 84;
1530552 128 78 64;
1530562 128 90 64;
1530692 176 88 19;
1530692 176 88 25;
1530692 144 92 89;
1530697 176 88 25;
1530697 176 88 84;
1530697 144 80 87;
1530927 128 80 64;
1530953 128 92 64;
1531208 176 88 84;
1531208 176 88 81;
1531208 144 93 87;
1531213 176 88 81;
1531213 176 88 24;
1531213 144 81 70;
1531468 128 81 64;
1531484 128 93 64;
1531729 176 88 24;
1531729 176 88 66;
1531729 144 94 92;
1531729 176 88 66;
1531729 176 88 83;
1531729 144 82 89;
1532223 128 94 64;
1532234 128 82 64;
1532359 176 88 83;
1532359 176 88 35;
1532359 144 94 83;
1532364 176 88 35;
1532364 176 88 116;
1532364 144 82 71;
1532437 128 82 64;
1532505 128 94 64;
1532588 176 88 116;
1532588 176 88 21;
1532588 144 81 79;
1532588 144 93 87;
1532656 128 81 64;
1532703 128 93 64;
1532854 176 88 21;
1532854 176 88 16;
1532854 144 94 85;
1532859 176 88 16;
1532859 176 88 58;
1532859 144 82 72;
1532932 128 82 64;
1532963 128 94 64;
1533088 176 88 58;
1533088 176 88 97;
1533088 144 93 53;
1533093 176 88 97;
1533093 176 88 109;
1533093 144 91 58;
1533104 176 88 109;
1533104 176 88 34;
1533104 144 79 29;
1533104 144 81 39;
1533151 128 91 64;
1533156 128 93 64;
1533166 128 81 64;
1533171 128 79 64;
1533375 176 88 34;
1533375 176 88 66;
1533375 144 94 84;
1533380 176 88 66;
1533380 176 88 24;
1533380 144 82 68;
1533458 128 82 64;
1533500 128 94 64;
1533656 176 88 24;
1533656 176 88 64;
1533656 144 93 89;
1533666 176 88 64;
1533666 176 88 79;
1533666 144 81 78;
1533921 128 81 64;
1533947 128 93 64;
1534031 176 88 79;
1534031 176 88 64;
1534031 144 93 89;
1534046 176 88 64;
1534046 176 88 25;
1534046 144 81 84;
1534250 128 81 64;
1534250 128 93 64;
1534364 176 88 25;
1534364 176 88 80;
1534364 144 93 65;
1534369 176 88 80;
1534369 176 88 1;
1534369 144 81 59;
1534505 128 81 64;
1534520 128 93 64;
1534682 176 88 1;
1534682 176 88 32;
1534682 144 93 93;
1534687 176 88 32;
1534687 176 88 28;
1534687 144 81 75;
1534901 128 81 64;
1534911 128 93 64;
1535010 176 88 28;
1535010 176 88 109;
1535010 144 93 77;
1535031 176 88 109;
1535031 176 88 87;
1535031 144 81 67;
1535171 128 81 64;
1535203 128 93 64;
1535359 176 88 87;
1535359 176 88 23;
1535359 144 93 80;
1535369 176 88 23;
1535369 176 88 35;
1535369 144 81 67;
1535520 128 81 64;
1535520 128 93 64;
1535677 176 88 35;
1535677 176 88 56;
1535677 144 81 71;
1535687 176 88 56;
1535687 176 88 65;
1535687 144 93 66;
1535802 128 81 64;
1536276 128 93 64;
1536286 176 88 65;
1536286 176 88 34;
1536286 144 92 49;
1536390 128 92 64;
1536395 176 88 34;
1536395 176 88 1;
1536395 144 93 54;
1536494 128 93 64;
1536510 176 88 1;
1536510 176 88 106;
1536510 144 92 49;
1536562 128 92 64;
1536614 176 88 106;
1536614 176 88 8;
1536614 144 93 26;
1536651 176 88 8;

1536651 176 88 94;
1536651 144 92 60;
1536687 128 93 64;
1536750 176 88 94;
1536750 176 88 82;
1536750 144 93 80;
1536765 128 92 64;
1536812 128 93 64;
1536833 176 88 82;
1536833 176 88 0;
1536833 144 92 70;
1536885 176 88 0;
1536885 176 88 4;
1536885 144 93 82;
1536916 128 92 64;
1537020 176 88 4;
1537020 176 88 12;
1537020 144 92 79;
1537078 128 93 64;
1537125 176 88 12;
1537125 176 88 89;
1537125 144 93 86;
1537286 128 93 64;
1537343 176 88 89;
1537343 176 88 111;
1537343 144 90 101;
1537390 128 92 64;
1537510 128 90 64;
1537635 176 88 111;
1537635 176 88 17;
1537635 144 87 90;
1537786 128 87 64;
1537890 176 88 17;
1537890 176 88 116;
1537890 144 87 94;
1538687 128 87 64;
1538848 176 88 116;
1538848 176 88 28;
1538848 144 87 44;
1539015 176 88 28;
1539015 176 88 37;
1539015 144 85 69;
1539125 128 87 64;
1539343 128 85 64;
1539359 176 88 37;
1539359 176 88 33;
1539359 144 82 45;
1539505 176 88 33;
1539505 176 88 115;
1539505 144 85 72;
1539557 128 82 64;
1539828 128 85 64;
1539854 176 88 115;
1539854 176 88 97;
1539854 144 87 87;
1541000 128 87 64;
1541026 176 88 97;
1541026 176 88 36;
1541026 144 81 44;
1541192 176 88 36;
1541192 176 88 4;
1541192 144 87 76;
1541223 128 81 64;
1541520 176 88 4;
1541520 176 88 97;
1541520 144 80 31;
1541692 176 88 97;
1541692 176 88 79;
1541692 144 85 63;
1541750 128 87 64;
1541755 128 80 64;
1542031 176 88 79;
1542031 176 88 33;
1542031 144 78 30;
1542057 128 85 64;
1542244 176 88 33;
1542244 176 88 100;
1542244 144 82 74;
1542291 128 78 64;
1542572 176 88 100;
1542572 176 88 44;
1542572 144 80 46;
1542619 128 82 64;
1542755 144 81 69;
1542817 128 80 64;
1543104 176 88 44;
1543104 176 88 31;
1543104 144 80 44;
1543114 128 81 64;
1543260 128 80 64;
1543281 176 88 31;
1543281 176 88 107;
1543281 144 78 59;
1543593 128 78 64;
1543625 176 88 107;
1543625 176 88 34;
1543625 144 75 51;
1544619 128 75 64;
1544770 176 88 34;
1544770 176 88 101;
1544770 144 75 90;
1544942 176 88 101;
1544942 176 88 114;
1544942 144 74 98;
1544994 128 75 64;
1545260 176 88 114;
1545260 176 88 4;
1545260 144 75 89;
1545307 128 74 64;
1545812 128 75 64;
1545828 176 88 4;
1545828 176 88 78;
1545828 144 74 76;
1546010 176 88 78;
1546010 176 88 95;
1546010 144 73 73;
1546041 128 74 64;
1546145 128 73 64;
1546385 176 88 95;
1546385 176 88 110;
1546385 144 74 50;
1546593 176 88 110;
1546593 176 88 121;
1546593 144 75 84;
1546614 128 74 64;
1547484 128 75 64;
1547484 176 88 121;
1547484 176 88 16;
1547484 144 74 56;
1547682 176 88 16;
1547682 176 88 103;
1547682 144 86 93;
1547765 128 74 64;
1548067 176 88 103;
1548067 176 88 2;
1548067 144 74 57;
1548072 128 86 64;
1548140 128 74 64;
1548296 176 88 2;
1548296 176 88 118;
1548296 144 87 69;
1548583 176 88 118;
1548583 176 88 119;
1548583 144 75 51;
1548604 128 87 64;
1548723 128 75 64;
1548791 176 88 119;
1548791 176 88 81;
1548791 144 86 85;
1549041 128 86 64;
1549088 176 88 81;
1549088 176 88 75;
1549088 144 74 39;
1549161 128 74 64;
1549328 176 88 75;
1549328 176 88 94;
1549328 144 87 90;
1549625 176 88 94;
1549625 176 88 52;
1549625 144 75 44;
1549645 128 87 64;
1549703 128 75 64;
1549848 176 88 52;
1549848 176 88 13;
1549848 144 86 96;
1550125 128 86 64;
1550151 176 88 13;
1550151 176 88 110;
1550151 144 74 51;
1550234 128 74 64;
1550395 176 88 110;
1550395 176 88 48;
1550395 144 85 91;
1550666 128 85 64;
1550687 176 88 48;
1550687 176 88 44;
1550687 144 73 58;
1550994 128 73 64;
1551000 176 88 44;
1551000 176 88 8;
1551000 144 85 74;
1551140 128 85 64;
1551302 176 88 8;
1551302 176 88 104;
1551302 144 73 46;
1551447 176 88 104;
1551447 176 88 87;
1551447 144 85 65;
1551458 128 73 64;
1551515 128 85 64;
1551541 176 88 87;
1551541 176 88 47;
1551541 144 73 44;
1551619 128 73 64;
1551645 176 88 47;
1551645 176 88 28;
1551645 144 85 57;
1551708 128 85 64;
1551718 176 88 28;
1551718 176 88 88;
1551718 144 73 43;
1551796 128 73 64;
1551838 176 88 88;
1551838 176 88 94;
1551838 144 85 53;
1551895 128 85 64;
1551911 176 88 94;
1551911 176 88 109;
1551911 144 73 36;
1552000 128 73 64;
1552026 176 88 109;
1552026 176 88 91;
1552026 144 85 66;
1552093 128 85 64;
1552135 176 88 91;
1552135 176 88 99;
1552135 144 73 35;
1552213 128 73 64;
1552223 176 88 99;
1552223 176 88 111;
1552223 144 85 71;
1552348 176 88 111;
1552348 176 88 75;
1552348 144 73 53;
1552364 128 85 64;
1552442 128 73 64;
1552458 176 88 75;
1552458 176 88 95;
1552458 144 85 67;
1552562 176 88 95;
1552562 176 88 72;
1552562 144 73 50;
1552572 128 85 64;
1552651 128 73 64;
1552666 176 88 72;
1552666 176 88 79;
1552666 144 85 62;
1552791 128 85 64;
1552802 176 88 79;
1552802 176 88 59;
1552802 144 73 67;
1552859 128 73 64;
1552906 176 88 59;
1552906 176 88 81;
1552906 144 85 28;
1552984 128 85 64;
1553052 176 88 81;
1553052 176 88 106;
1553052 144 82 87;
1553057 176 88 106;
1553057 176 88 50;
1553057 144 70 73;
1553208 128 70 64;
1553239 128 82 64;
1553380 176 88 50;
1553380 176 88 28;
1553380 144 70 88;
1553385 176 88 28;
1553385 176 88 54;
1553385 144 82 85;
1553437 128 70 64;
1553437 128 82 64;
1553677 176 88 54;
1553677 176 88 58;
1553677 144 70 82;
1553677 176 88 58;
1553677 176 88 109;
1553677 144 82 90;
1553723 128 82 64;
1553729 128 70 64;
1553942 176 88 109;
1553942 176 88 80;
1553942 144 82 86;
1553942 176 88 80;
1553942 176 88 102;
1553942 144 70 76;
1554020 128 70 64;
1554046 128 82 64;
1554218 176 88 102;
1554218 176 88 25;
1554218 144 81 84;
1554229 176 88 25;
1554229 176 88 49;
1554229 144 69 68;
1554296 128 69 64;
1554317 128 81 64;
1554500 176 88 49;
1554500 176 88 21;
1554500 144 68 68;
1554500 144 80 77;
1554546 128 80 64;
1554557 128 68 64;
1554791 176 88 21;
1554791 176 88 18;
1554791 144 66 77;
1554796 176 88 18;
1554796 176 88 54;
1554796 144 78 82;
1554843 128 78 64;
1554843 128 66 64;
1555098 176 88 54;
1555098 176 88 36;
1555098 144 75 87;
1555109 176 88 36;
1555109 176 88 127;
1555109 144 63 74;
1556489 128 75 64;
1556651 176 88 127;
1556651 176 88 33;
1556651 144 75 12;
1556755 128 75 64;
1557015 128 63 64;
1557463 176 88 33;
1557463 176 88 37;
1557463 144 63 43;
1557572 128 63 64;
1557593 176 88 37;
1557593 176 88 27;
1557593 144 64 63;
1557661 128 64 64;
1557718 176 88 27;
1557718 176 88 93;
1557718 144 65 47;
1557869 176 88 93;
1557869 176 88 120;
1557869 144 66 61;
1557875 128 65 64;
1558036 176 88 120;
1558036 176 88 38;
1558036 128 66 64;
1558036 144 67 86;
1558177 128 67 64;
1558192 176 88 38;
1558192 176 88 20;
1558192 144 68 63;
1558359 128 68 64;
1558375 176 88 20;
1558375 176 88 96;
1558375 144 69 66;
1558505 128 69 64;
1558541 176 88 96;
1558541 176 88 30;

1558541 144 70 69;
1558890 128 70 64;
1558901 176 88 30;
1558901 176 88 101;
1558901 144 69 53;
1559177 176 88 101;
1559177 176 88 0;
1559177 144 68 49;
1559187 128 69 64;
1559484 176 88 0;
1559484 176 88 18;
1559484 144 66 48;
1559531 128 68 64;
1559796 176 88 18;
1559796 176 88 35;
1559796 144 63 80;
1559822 128 66 64;
1561510 128 63 64;
1562203 176 88 35;
1562203 176 88 50;
1562203 144 75 43;
1562286 128 75 64;
1562307 176 88 50;
1562307 176 88 46;
1562307 144 76 69;
1562427 128 76 64;
1562437 176 88 46;
1562437 176 88 59;
1562437 144 77 47;
1562541 176 88 59;
1562541 176 88 118;
1562541 144 78 69;
1562546 128 77 64;
1562697 128 78 64;
1562901 176 88 118;
1562901 176 88 78;
1562901 144 79 76;
1562979 128 79 64;
1563203 176 88 78;
1563203 176 88 60;
1563203 144 80 80;
1563338 128 80 64;
1563489 176 88 60;
1563489 176 88 88;
1563489 144 81 93;
1563640 128 81 64;
1563755 144 80 90;
1563921 128 80 64;
1563994 176 88 88;
1563994 176 88 33;
1563994 144 81 93;
1564302 176 88 33;
1564302 176 88 106;
1564302 144 80 82;
1564333 128 81 64;
1564437 176 88 106;
1564437 176 88 120;
1564437 128 80 64;
1564437 144 81 53;
1564552 176 88 120;
1564552 176 88 119;
1564552 144 80 48;
1564562 128 81 64;
1564614 128 80 64;
1564630 176 88 119;
1564630 176 88 91;
1564630 144 81 53;
1564723 176 88 91;
1564723 176 88 35;
1564723 128 81 64;
1564723 144 80 60;
1564781 128 80 64;
1564796 176 88 35;
1564796 176 88 8;
1564796 144 81 51;
1564895 176 88 8;
1564895 176 88 17;
1564895 144 80 56;
1564932 128 81 64;
1564979 128 80 64;
1564984 176 88 17;
1564984 176 88 16;
1564984 144 81 73;
1565234 176 88 16;
1565234 176 88 63;
1565234 144 82 79;
1565281 128 81 64;
1565484 176 88 63;
1565484 176 88 121;
1565484 144 81 88;
1565567 128 82 64;
1565760 176 88 121;
1565760 176 88 84;
1565760 144 80 87;
1565828 128 81 64;
1566630 176 88 84;
1566630 176 88 46;
1566630 144 78 78;
1566635 128 80 64;
1566817 176 88 46;
1566817 176 88 98;
1566817 144 80 72;
1566854 128 78 64;
1566979 176 88 98;
1566979 176 88 4;
1566979 144 82 83;
1567015 128 80 64;
1567145 128 82 64;
1567166 176 88 4;
1567166 176 88 117;
1567166 144 83 90;
1567348 176 88 117;
1567348 176 88 105;
1567348 144 84 89;
1567385 128 83 64;
1567531 176 88 105;
1567531 176 88 79;
1567531 144 85 89;
1567557 128 84 64;
1567734 176 88 79;
1567734 176 88 72;
1567734 144 87 86;
1567770 128 85 64;
1567916 176 88 72;
1567916 176 88 44;
1567916 144 85 80;
1568000 128 87 64;
1568114 176 88 44;
1568114 176 88 99;
1568114 144 87 77;
1568166 128 85 64;
1568270 128 87 64;
1568312 176 88 99;
1568312 176 88 116;
1568312 144 87 94;
1569078 128 87 64;
1569208 176 88 116;
1569208 176 88 85;
1569208 144 87 64;
1569380 176 88 85;
1569380 176 88 45;
1569380 144 86 93;
1569401 128 87 64;
1569687 176 88 45;
1569687 176 88 81;
1569687 144 85 82;
1569718 128 86 64;
1570182 128 85 64;
1570296 176 88 81;
1570296 176 88 16;
1570296 144 85 64;
1570463 176 88 16;
1570463 176 88 127;
1570463 144 86 86;
1570468 128 85 64;
1570755 128 86 64;
1570786 176 88 127;
1570786 176 88 125;
1570786 144 87 70;
1571203 128 87 64;
1571453 176 88 125;
1571453 176 88 27;
1571453 144 87 56;
1571609 176 88 27;
1571609 176 88 57;
1571609 144 86 70;
1571614 128 87 64;
1571854 128 86 64;
1571895 176 88 57;
1571895 176 88 31;
1571895 144 85 61;
1572088 128 85 64;
1572171 176 88 31;
1572171 176 88 68;
1572171 144 85 85;
1572406 128 85 64;
1572489 176 88 68;
1572489 176 88 99;
1572489 144 85 75;
1572854 144 75 75;
1573864 128 85 64;
1574026 128 75 64;
1574296 176 88 99;
1574296 176 88 101;
1574296 144 75 60;
1574479 128 75 64;
1574604 176 88 101;
1574604 176 88 83;
1574604 144 76 64;
1574822 128 76 64;
1575125 176 88 83;
1575125 176 88 111;
1575125 144 75 71;
1575270 128 75 64;
1575333 176 88 111;
1575333 176 88 59;
1575333 144 76 84;
1575473 128 76 64;
1575651 176 88 59;
1575651 176 88 107;
1575651 144 77 72;
1575796 128 77 64;
1575890 176 88 107;
1575890 176 88 66;
1575890 144 78 92;
1576171 128 78 64;
1576234 176 88 66;
1576234 176 88 120;
1576234 144 80 94;
1576677 176 88 120;
1576677 176 88 87;
1576677 128 80 64;
1576677 144 78 80;
1577031 128 78 64;
1577067 176 88 87;
1577067 176 88 126;
1577067 144 80 46;
1577208 128 80 64;
1577270 176 88 126;
1577270 176 88 35;
1577270 144 80 60;
1577328 128 80 64;
1577364 176 88 35;
1577364 176 88 42;
1577364 144 78 49;
1577411 176 88 42;
1577411 176 88 85;
1577411 144 80 48;
1577421 128 78 64;
1577500 176 88 85;
1577500 176 88 4;
1577500 144 78 76;
1577557 128 80 64;
1577671 176 88 4;
1577671 176 88 62;
1577671 144 75 95;
1577692 128 78 64;
1577953 128 75 64;
1577963 176 88 62;
1577963 176 88 89;
1577963 144 73 81;
1578708 128 73 64;
1578979 176 88 89;
1578979 176 88 71;
1578979 144 73 80;
1578984 176 88 71;
1578984 176 88 21;
1578984 144 63 64;
1579041 128 63 64;
1579046 128 73 64;
1579151 176 88 21;
1579151 176 88 87;
1579151 144 63 79;
1579156 176 88 87;
1579156 176 88 34;
1579156 144 73 82;
1579270 128 63 64;
1579317 128 73 64;
1579515 176 88 34;
1579515 176 88 89;
1579515 144 66 81;
1579526 176 88 89;
1579526 176 88 25;
1579526 144 75 83;
1579833 128 66 64;
1579848 128 75 64;
1580000 176 88 25;
1580000 176 88 26;
1580000 144 66 89;
1580000 144 75 90;
1580229 128 66 64;
1580260 128 75 64;
1580312 176 88 26;
1580312 176 88 123;
1580312 144 66 90;
1580312 176 88 123;
1580312 176 88 38;
1580312 144 75 94;
1580546 128 75 64;
1580552 128 66 64;
1580630 176 88 38;
1580630 176 88 64;
1580630 144 75 73;
1580630 176 88 64;
1580630 176 88 106;
1580630 144 66 82;
1580880 128 66 64;
1580895 128 75 64;
1580963 176 88 106;
1580963 176 88 7;
1580963 144 75 82;
1580963 176 88 7;
1580963 176 88 106;
1580963 144 66 82;
1581213 128 66 64;
1581213 128 75 64;
1581291 176 88 106;
1581291 176 88 81;
1581291 144 75 78;
1581291 176 88 81;
1581291 176 88 93;
1581291 144 66 85;
1582135 128 75 64;
1582140 128 66 64;
1582223 176 88 93;
1582223 176 88 53;
1582223 144 66 49;
1582229 176 88 53;
1582229 176 88 58;
1582229 144 75 48;
1582354 128 66 64;
1582395 128 75 64;
1582421 176 88 58;
1582421 176 88 74;
1582421 144 66 76;
1582447 176 88 74;
1582447 176 88 77;
1582447 144 73 72;
1582755 128 66 64;
1582796 128 73 64;
1582822 176 88 77;
1582822 176 88 122;
1582822 144 66 66;
1582838 176 88 122;
1582838 176 88 12;
1582838 144 73 72;
1583125 128 66 64;
1583171 128 73 64;
1583255 176 88 12;
1583255 176 88 109;
1583255 144 73 54;
1583265 176 88 109;
1583265 176 88 52;
1583265 144 66 65;
1583510 128 66 64;
1583562 176 88 52;
1583562 176 88 54;
1583562 128 73 64;
1583562 144 66 74;
1583562 144 73 41;
1583921 176 88 54;
1583921 176 88 18;

1583921 144 70 58;
1583942 128 66 64;
1584010 128 73 64;
1584192 128 70 64;
1584234 176 88 18;
1584234 176 88 73;
1584234 144 67 30;
1584239 176 88 73;
1584239 176 88 123;
1584239 144 69 26;
1584317 128 67 64;
1584390 128 69 64;
1584463 176 88 123;
1584463 176 88 44;
1584463 144 69 66;
1584677 176 88 44;
1584677 176 88 34;
1584677 128 69 64;
1584677 144 70 61;
1584791 128 70 64;
1584807 176 88 34;
1584807 176 88 107;
1584807 144 69 58;
1584875 128 69 64;
1584901 144 70 56;
1584968 128 70 64;
1584989 176 88 107;
1584989 176 88 59;
1584989 144 69 61;
1585062 128 69 64;
1585072 176 88 59;
1585072 176 88 88;
1585072 144 68 72;
1585203 176 88 88;
1585203 176 88 15;
1585203 144 66 81;
1585265 128 68 64;
1585463 176 88 15;
1585463 176 88 44;
1585463 144 63 77;
1585468 128 66 64;
1585718 128 63 64;
1585765 176 88 44;
1585765 176 88 103;
1585765 144 63 92;
1586666 176 88 103;
1586666 176 88 55;
1586666 144 51 53;
1586677 128 63 64;
1586880 176 88 55;
1586880 176 88 105;
1586880 144 63 66;
1586979 128 51 64;
1587234 176 88 105;
1587234 176 88 85;
1587234 144 51 42;
1587281 128 63 64;
1588052 128 51 64;
1588317 176 88 85;
1588317 176 88 69;
1588317 144 51 67;
1588328 176 88 69;
1588328 176 88 6;
1588328 144 63 52;
1588369 128 51 64;
1588390 128 63 64;
1588484 176 88 6;
1588484 176 88 52;
1588484 144 64 49;
1588500 176 88 52;
1588500 176 88 16;
1588500 144 52 37;
1588557 128 52 64;
1588604 128 64 64;
1588854 176 88 16;
1588854 176 88 110;
1588854 144 54 50;
1588854 176 88 110;
1588854 176 88 51;
1588854 144 66 62;
1588906 128 54 64;
1588911 128 66 64;
1589031 176 88 51;
1589031 176 88 48;
1589031 144 56 91;
1589036 176 88 48;
1589036 176 88 65;
1589036 144 68 85;
1589125 128 56 64;
1589140 128 68 64;
1589369 176 88 65;
1589369 176 88 74;
1589369 144 54 71;
1589369 144 66 71;
1589427 128 54 64;
1589489 128 66 64;
1589812 176 88 74;
1589812 176 88 34;
1589812 144 52 36;
1589817 176 88 34;
1589817 176 88 70;
1589817 144 64 31;
1589869 128 52 64;
1589890 128 64 64;
1590130 176 88 70;
1590130 176 88 83;
1590130 144 63 72;
1590140 176 88 83;
1590140 176 88 76;
1590140 144 51 68;
1591395 128 63 64;
1591401 128 51 64;
1591505 176 88 76;
1591505 176 88 90;
1591505 144 63 51;
1591515 176 88 90;
1591515 176 88 63;
1591515 144 51 43;
1591708 128 51 64;
1591734 128 63 64;
1591979 176 88 63;
1591979 176 88 52;
1591979 144 62 48;
1591984 176 88 52;
1591984 176 88 58;
1591984 144 50 43;
1592057 128 50 64;
1592083 128 62 64;
1592203 176 88 58;
1592203 176 88 44;
1592203 144 61 72;
1592213 176 88 44;
1592213 176 88 30;
1592213 144 49 72;
1592473 128 49 64;
1592494 128 61 64;
1592593 176 88 30;
1592593 176 88 34;
1592593 144 62 51;
1592593 176 88 34;
1592593 176 88 108;
1592593 144 50 65;
1592640 176 88 108;
1592640 176 88 22;
1592640 144 60 32;
1592713 128 60 64;
1592755 128 50 64;
1592765 128 62 64;
1593062 176 88 22;
1593062 176 88 73;
1593062 144 51 78;
1593062 144 63 85;
1593161 128 51 64;
1593208 128 63 64;
1593333 176 88 73;
1593333 176 88 53;
1593333 144 50 65;
1593343 176 88 53;
1593343 176 88 6;
1593343 144 62 48;
1593401 128 50 64;
1593442 128 62 64;
1593635 176 88 6;
1593635 176 88 72;
1593635 144 61 77;
1593640 176 88 72;
1593640 176 88 118;
1593640 144 49 76;
1593697 128 49 64;
1593781 128 61 64;
1593890 144 50 80;
1593895 176 88 118;
1593895 176 88 81;
1593895 144 62 55;
1593906 176 88 81;
1593906 176 88 83;
1593906 144 60 64;
1593963 128 60 64;
1593994 128 50 64;
1594010 128 62 64;
1594276 176 88 83;
1594276 176 88 0;
1594276 144 51 65;
1594276 144 63 70;
1597921 128 51 64;
1598593 128 63 64;
1599067 176 88 0;
1599067 176 88 101;
1599067 144 63 31;
1599130 176 88 101;
1599130 176 88 94;
1599130 144 66 34;
1599182 176 88 94;
1599182 176 88 77;
1599182 144 70 36;
1599244 128 66 64;
1599281 128 63 64;
1599302 128 70 64;
1627125 176 88 77;
1627125 176 88 66;
1627125 144 70 28;
1627135 176 88 66;
1627135 176 88 77;
1627135 144 66 37;
1627140 176 88 77;
1627140 176 88 55;
1627140 144 63 15;
1627270 128 63 64;
1627302 128 66 64;
1627312 128 70 64;
1627739 176 88 55;
1627739 176 88 15;
1627739 144 73 33;
1627744 176 88 15;
1627744 176 88 46;
1627744 144 63 28;
1627750 176 88 46;
1627750 176 88 118;
1627750 144 68 34;
1627937 128 63 64;
1627963 128 73 64;
1627973 128 68 64;
1628255 176 88 118;
1628255 176 88 114;
1628255 144 70 45;
1628265 176 88 114;
1628265 176 88 13;
1628265 144 63 29;
1628427 128 70 64;
1628437 128 63 64;
1628859 176 88 13;
1628859 176 88 115;
1628859 144 70 39;
1628895 176 88 115;
1628895 176 88 74;
1628895 144 63 21;
1628901 176 88 74;
1628901 176 88 112;
1628901 144 66 24;
1629000 128 66 64;
1629000 128 70 64;
1629005 128 63 64;
1629734 176 88 112;
1629734 176 88 4;
1629734 144 68 39;
1629765 176 88 4;
1629765 176 88 38;
1629765 144 61 29;
1629802 176 88 38;
1629802 176 88 85;
1629802 144 63 24;
1629895 128 63 64;
1629911 128 61 64;
1629927 128 68 64;
1810291 176 88 85;
1810291 176 88 121;
1810291 144 66 30;
1810557 176 88 121;
1810557 176 88 22;
1810557 144 68 30;
1810713 128 66 64;
1810843 128 68 64;
1811901 176 88 22;
1811901 176 88 24;
1811901 144 63 12;
1812093 176 88 24;
1812093 176 88 77;
1812093 144 68 11;
1812098 176 88 77;
1812098 176 88 116;
1812098 144 66 6;
1812098 144 70 14;
1812286 176 88 116;
1812286 176 88 0;
1812286 144 61 1;
1812427 128 66 64;
1812432 128 70 64;
1812447 128 68 64;
1812484 128 63 64;
1812510 128 61 64;
1816838 176 88 0;
1816838 176 88 120;
1816838 144 65 49;
1816989 128 65 64;
1817416 176 88 120;
1817416 176 88 126;
1817416 144 72 43;
1818010 128 72 64;
1827151 176 88 126;
1827151 176 88 78;
1827151 144 63 30;
1827385 128 63 64;
1827411 176 88 78;
1827411 176 88 86;
1827411 144 65 45;
1827671 176 88 86;
1827671 176 88 8;
1827671 144 66 45;
1827687 128 65 64;
1827979 176 88 8;
1827979 176 88 13;
1827979 144 68 42;
1827994 128 66 64;
1828312 128 68 64;
1828317 176 88 13;
1828317 176 88 24;
1828317 144 70 45;
1828963 176 88 24;
1828963 176 88 22;
1828963 144 72 41;
1829000 128 70 64;
1830723 176 88 22;
1830723 176 88 24;
1830723 128 72 64;
1830723 144 73 35;
1831458 176 88 24;
1831458 176 88 27;
1831458 128 73 64;
1831458 144 75 31;
1832505 176 88 27;
1832505 176 88 29;
1832505 144 73 33;
1832536 128 75 64;
1832755 128 73 64;
1832786 176 88 29;
1832786 176 88 92;
1832786 144 72 34;
1832953 128 72 64;
1833026 176 88 92;
1833026 176 88 90;
1833026 144 70 41;
1833260 176 88 90;
1833260 176 88 0;
1833260 144 68 37;
1833270 128 70 64;
1833578 176 88 0;
1833578 176 88 5;
1833578 144 66 31;
1833604 128 68 64;
1833760 128 66 64;
1833796 176 88 5;
1833796 176 88 58;

1833796 144 65 31;
1833937 128 65 64;
1834031 176 88 58;
1834031 176 88 46;
1834031 144 63 28;
1834307 128 63 64;
1834807 176 88 46;
1834807 176 88 69;
1834807 144 63 28;
1834984 128 63 64;
1835015 176 88 69;
1835015 176 88 111;
1835015 144 65 40;
1835213 128 65 64;
1835276 176 88 111;
1835276 176 88 86;
1835276 144 66 30;
1835578 128 66 64;
1835604 176 88 86;
1835604 176 88 24;
1835604 144 68 26;
1835854 128 68 64;
1835885 176 88 24;
1835885 176 88 106;
1835885 144 70 23;
1836171 128 70 64;
1836218 176 88 106;
1836218 176 88 61;
1836218 144 72 35;
1838479 128 72 64;
1838765 176 88 61;
1838765 176 88 66;
1838765 144 72 32;
1838807 176 88 66;
1838807 176 88 114;
1838807 144 73 35;
1838848 128 72 64;
1838901 176 88 114;
1838901 176 88 123;
1838901 144 75 33;
1838906 128 73 64;
1839359 128 75 64;
1870333 176 64 0;
1870333 176 64 127;
1890734 176 88 123;
1890734 176 88 63;
1890734 144 27 33;
1890869 176 64 127;
1890869 176 64 0;
1891119 128 27 64;
1894421 176 88 63;
1894421 176 88 19;
1894421 144 27 34;
1894421 176 88 19;
1894421 176 88 97;
1894421 144 39 33;
1895020 128 39 64;
1895083 128 27 64;
1895703 176 88 97;
1895703 176 88 80;
1895703 144 25 39;
1895718 176 88 80;
1895718 176 88 13;
1895718 144 37 34;
1896333 128 37 64;
1896359 128 25 64;
1900645 176 64 0;
1900645 176 64 127;
1901270 176 64 127;
1901270 176 64 0;
2000750 144 70 37;
2000760 176 88 13;
2000760 176 88 65;
2000760 144 63 25;
2000781 176 88 65;
2000781 176 88 117;
2000781 144 66 32;
2001083 128 63 64;
2001109 128 66 64;
2001125 128 70 64;
2002161 176 88 117;
2002161 176 88 96;
2002161 144 65 27;
2002171 176 88 96;
2002171 176 88 103;
2002171 144 68 34;
2002171 144 72 39;
2002583 128 65 64;
2002598 128 72 64;
2002604 128 68 64;
2003026 176 88 103;
2003026 176 88 0;
2003026 144 66 32;
2003026 144 73 36;
2003052 176 88 0;
2003052 176 88 108;
2003052 144 70 37;
2003614 128 70 64;
2003630 128 66 64;
2003645 128 73 64;
2004010 176 88 108;
2004010 176 88 29;
2004010 144 68 28;
2004015 176 88 29;
2004015 176 88 58;
2004015 144 75 41;
2004036 176 88 58;
2004036 176 88 40;
2004036 144 72 37;
2004697 128 72 64;
2004703 128 75 64;
2004703 128 68 64;
2005072 176 88 40;
2005072 176 88 78;
2005072 144 77 43;
2005078 176 88 78;
2005078 176 88 4;
2005078 144 70 32;
2005093 176 88 4;
2005093 176 88 114;
2005093 144 73 35;
2006692 128 73 64;
2006692 128 77 64;
2006697 128 70 64;
2007500 176 88 114;
2007500 176 88 126;
2007500 144 75 37;
2007510 176 88 126;
2007510 176 88 113;
2007510 144 78 37;
2007531 176 88 113;
2007531 176 88 99;
2007531 144 72 27;
2008671 128 75 64;
2008708 128 72 64;
2008786 128 78 64;
2009520 176 88 99;
2009520 176 88 49;
2009520 144 73 29;
2009526 176 88 49;
2009526 176 88 45;
2009526 144 80 34;
2009536 144 77 37;
2010536 128 77 64;
2010614 128 73 64;
2010890 128 80 64;
2093578 176 64 0;
2093578 176 64 127;
2099593 176 64 127;
2099593 176 64 0;
2102666 176 64 0;
2102666 176 64 127;
2103781 176 64 127;
2103781 176 64 0;
2104067 176 64 0;
2104067 176 64 127;
2104692 176 64 127;
2104692 176 64 0;
2105161 176 64 0;
2105161 176 64 127;
2120869 176 64 127;
2120869 176 64 0;
2121276 176 64 0;
2121276 176 64 127;
2121312 176 88 45;
2121312 176 88 62;
2121312 144 27 36;
2121328 176 88 62;
2121328 176 88 68;
2121328 144 39 33;
2121427 128 39 64;
2121468 128 27 64;
2124093 176 88 68;
2124093 176 88 99;
2124093 144 80 35;
2124244 128 80 64;
2124718 176 88 99;
2124718 176 88 115;
2124718 144 80 36;
2124859 128 80 64;
2126380 176 88 115;
2126380 176 88 29;
2126380 144 80 55;
2128895 128 80 64;
2128953 176 88 29;
2128953 176 88 54;
2128953 144 78 32;
2133145 128 78 64;
2133177 176 88 54;
2133177 176 88 68;
2133177 144 27 31;
2133187 176 88 68;
2133187 176 88 43;
2133187 144 39 34;
2133291 176 64 127;
2133291 176 64 0;
2133906 176 64 0;
2133906 176 64 127;
2134500 128 39 64;
2134656 128 27 64;
2136401 176 88 43;
2136401 176 88 34;
2136401 144 92 49;
2137661 176 88 34;
2137661 176 88 98;
2137661 144 94 47;
2137723 128 92 64;
2139380 128 94 64;
2140578 176 88 98;
2140578 176 88 6;
2140578 144 94 29;
2140677 128 94 64;
2141583 176 88 6;
2141583 176 88 69;
2141583 144 94 27;
2141682 128 94 64;
2142677 176 88 69;
2142677 176 88 108;
2142677 144 94 18;
2142770 128 94 64;
2144364 176 88 108;
2144364 176 88 100;
2144364 144 94 14;
2145015 128 94 64;
2145015 176 88 100;
2145015 176 88 72;
2145015 144 97 29;
2148447 128 97 64;
2148473 176 88 72;
2148473 176 88 71;
2148473 144 99 31;
2148494 176 88 71;
2148494 176 88 104;
2148494 144 27 18;
2148526 176 88 104;
2148526 176 88 15;
2148526 144 39 21;
2148531 176 64 127;
2148531 176 64 0;
2148895 176 64 0;
2148895 176 64 127;
2149880 128 99 64;
2150901 128 27 64;
2150901 128 39 64;
2151838 176 88 15;
2151838 176 88 7;
2151838 144 87 20;
2151843 176 88 7;
2151843 176 88 112;
2151843 144 99 17;
2151953 128 87 64;
2151963 128 99 64;
2152723 176 88 112;
2152723 176 88 47;
2152723 144 85 28;
2152744 176 88 47;
2152744 176 88 45;
2152744 144 97 22;
2152822 128 85 64;
2152843 128 97 64;
2154796 176 88 45;
2154796 176 88 58;
2154796 144 82 9;
2154916 128 82 64;
2156010 176 88 58;
2156010 176 88 54;
2156010 144 80 11;
2156088 128 80 64;
2156921 176 88 54;
2156921 176 88 56;
2156921 144 90 19;
2156932 176 88 56;
2156932 176 88 76;
2156932 144 78 15;
2157026 128 90 64;
2157036 128 78 64;
2158781 176 88 76;
2158781 176 88 60;
2158781 144 75 12;
2158817 176 88 60;
2158817 176 88 9;
2158817 144 87 6;
2159692 128 75 64;
2159713 128 87 64;
2161630 176 88 9;
2161630 176 88 107;
2161630 144 90 16;
2161651 176 88 107;
2161651 176 88 15;
2161651 144 78 17;
2163187 128 90 64;
2163276 128 78 64;
2166348 176 88 15;
2166348 176 88 5;
2166348 144 27 21;
2166354 176 88 5;
2166354 176 88 79;
2166354 144 87 17;
2166354 176 88 79;
2166354 176 88 90;
2166354 144 75 19;
2166369 176 64 127;
2166369 176 64 0;
2166401 176 88 90;
2166401 176 88 106;
2166401 144 39 21;
2166869 176 64 0;
2166869 176 64 127;
2168072 128 87 64;
2168130 128 75 64;
2170625 128 39 64;
2170651 128 27 64;
2170697 176 88 106;
2170697 176 88 26;
2170697 144 73 20;
2172223 128 73 64;
2172291 176 88 26;
2172291 176 88 1;
2172291 144 75 22;
2173375 128 75 64;
2175864 176 88 1;
2175864 176 88 108;
2175864 144 78 14;
2178796 176 88 108;
2178796 176 88 0;
2178796 144 80 1;
2178838 128 78 64;
2180041 176 88 0;
2180041 176 88 13;
2180041 144 82 34;
2180041 176 88 13;
2180041 176 88 106;
2180041 144 70 13;
2180046 128 80 64;
2182171 128 70 64;
2185567 176 88 106;
2185567 176 88 87;
2185567 144 27 23;
2185583 176 88 87;
2185583 176 88 33;
2185583 144 39 18;
2185635 128 82 64;

2185640 176 64 127;
2185640 176 64 0;
2186385 176 64 0;
2186385 176 64 127;
2188552 128 27 64;
2188656 128 39 64;
2188869 176 88 33;
2188869 176 88 34;
2188869 144 99 14;
2193322 176 88 34;
2193322 176 88 111;
2193322 144 102 19;
2193364 128 99 64;
2194739 176 88 111;
2194739 176 88 24;
2194739 128 102 64;
2194739 144 104 28;
2195937 128 104 64;
2195937 176 88 24;
2195937 176 88 41;
2195937 144 106 28;
2196208 128 106 64;
2198614 144 96 25;
2198656 176 88 41;
2198656 176 88 105;
2198656 144 89 15;
2198760 128 96 64;
2198765 128 89 64;
2199406 176 88 105;
2199406 176 88 16;
2199406 144 96 27;
2199526 128 96 64;
2200177 176 88 16;
2200177 176 88 18;
2200177 144 89 25;
2200177 176 88 18;
2200177 176 88 91;
2200177 144 96 27;
2200302 128 96 64;
2200317 128 89 64;
2202093 176 88 91;
2202093 176 88 107;
2202093 144 89 16;
2202098 176 88 107;
2202098 176 88 34;
2202098 144 96 21;
2202239 128 89 64;
2202239 128 96 64;
2203671 176 88 34;
2203671 176 88 65;
2203671 144 27 21;
2203697 176 88 65;
2203697 176 88 61;
2203697 144 39 22;
2203713 176 64 127;
2203713 176 64 0;
2204218 176 64 0;
2204218 176 64 127;
2205760 128 39 64;
2205932 128 27 64;
2206083 176 88 61;
2206083 176 88 82;
2206083 144 87 22;
2209390 176 88 82;
2209390 176 88 44;
2209390 144 85 15;
2209411 128 87 64;
2213484 176 88 44;
2213484 176 88 70;
2213484 144 84 15;
2213526 128 85 64;
2216343 128 84 64;
2216411 176 88 70;
2216411 176 88 0;
2216411 144 82 16;
2219067 128 82 64;
2219083 176 88 0;
2219083 176 88 115;
2219083 144 84 15;
2221578 176 88 115;
2221578 176 88 126;
2221578 144 77 13;
2221734 128 84 64;
2225828 176 88 126;
2225828 176 88 70;
2225828 144 39 22;
2225864 176 88 70;
2225864 176 88 104;
2225864 144 27 9;
2226083 176 64 127;
2226083 176 64 0;
2226588 176 64 0;
2226588 176 64 127;
2228604 128 77 64;
2228687 176 88 104;
2228687 176 88 36;
2228687 144 75 16;
2230432 128 39 64;
2230958 128 75 64;
2231072 176 88 36;
2231072 176 88 75;
2231072 144 72 23;
2231218 176 88 75;
2231218 176 88 13;
2231218 144 77 4;
2231229 128 27 64;
2231328 128 72 64;
2231380 128 77 64;
2231765 176 88 13;
2231765 176 88 4;
2231765 144 77 24;
2231770 176 88 4;
2231770 176 88 0;
2231770 144 72 21;
2231927 128 72 64;
2231953 128 77 64;
2232520 176 88 0;
2232520 176 88 24;
2232520 144 77 21;
2232531 176 88 24;
2232531 176 88 51;
2232531 144 72 17;
2232666 128 72 64;
2232677 128 77 64;
2233270 176 88 51;
2233270 176 88 4;
2233270 144 77 27;
2233276 176 88 4;
2233276 176 88 11;
2233276 144 84 34;
2233364 128 84 64;
2233375 128 77 64;
2234291 176 88 11;
2234291 176 88 35;
2234291 144 84 37;
2234307 176 88 35;
2234307 176 88 8;
2234307 144 77 26;
2234416 128 77 64;
2234421 128 84 64;
2236661 176 88 8;
2236661 176 88 5;
2236661 144 77 28;
2236666 176 88 5;
2236666 176 88 102;
2236666 144 72 22;
2236786 128 72 64;
2236791 128 77 64;
2237776 176 88 102;
2237776 176 88 60;
2237776 144 72 23;
2237776 144 77 31;
2237869 128 77 64;
2237885 128 72 64;
2238338 176 88 60;
2238338 176 88 92;
2238338 144 72 34;
2238348 176 88 92;
2238348 176 88 43;
2238348 144 77 39;
2238411 128 72 64;
2238427 128 77 64;
2238994 176 88 43;
2238994 176 88 65;
2238994 144 84 35;
2238994 176 88 65;
2238994 176 88 98;
2238994 144 77 31;
2239072 128 84 64;
2239078 128 77 64;
2240187 176 64 127;
2240187 176 64 0;
2240203 176 88 98;
2240203 176 88 96;
2240203 144 27 26;
2240213 176 88 96;
2240213 176 88 33;
2240213 144 84 29;
2240218 176 88 33;
2240218 176 88 7;
2240218 144 39 26;
2240229 176 88 7;
2240229 176 88 115;
2240229 144 77 23;
2240583 176 64 0;
2240583 176 64 127;
2240869 128 77 64;
2240932 128 84 64;
2241515 176 88 115;
2241515 176 88 16;
2241515 144 89 46;
2241531 176 88 16;
2241531 176 88 80;
2241531 144 84 29;
2241630 128 84 64;
2241635 128 89 64;
2242062 176 88 80;
2242062 176 88 53;
2242062 144 89 38;
2242078 176 88 53;
2242078 176 88 116;
2242078 144 84 27;
2242156 128 89 64;
2242166 128 84 64;
2242276 128 27 64;
2242296 128 39 64;
2242619 176 88 116;
2242619 176 88 110;
2242619 144 89 33;
2242640 176 88 110;
2242640 176 88 44;
2242640 144 96 21;
2242713 128 89 64;
2242718 128 96 64;
2243458 176 88 44;
2243458 176 88 94;
2243458 144 96 24;
2243468 176 88 94;
2243468 176 88 26;
2243468 144 89 21;
2244484 128 89 64;
2244505 128 96 64;
2244520 176 88 26;
2244520 176 88 70;
2244520 144 94 12;
2244817 128 94 64;
2246270 176 88 70;
2246270 176 88 16;
2246270 144 96 24;
2246286 176 88 16;
2246286 176 88 44;
2246286 144 89 29;
2246921 128 96 64;
2246942 128 89 64;
2246994 176 88 44;
2246994 176 88 102;
2246994 144 94 16;
2247145 128 94 64;
2247963 176 88 102;
2247963 176 88 73;
2247963 144 77 18;
2248026 176 88 73;
2248026 176 88 96;
2248026 144 84 14;
2249390 128 77 64;
2249447 128 84 64;
2249484 176 88 96;
2249484 176 88 11;
2249484 144 82 11;
2249744 128 82 64;
2251562 176 88 11;
2251562 176 88 0;
2251562 144 65 1;
2251609 144 72 1;
2251687 128 72 64;
2251729 128 65 64;
2253317 176 88 0;
2253317 176 88 74;
2253317 144 70 15;
2253416 176 88 74;
2253416 176 88 47;
2253416 144 68 35;
2253432 128 70 64;
2253572 128 68 64;
2253578 176 88 47;
2253578 176 88 0;
2253578 144 66 36;
2253656 128 66 64;
2253750 176 88 0;
2253750 176 88 81;
2253750 144 65 32;
2254255 128 65 64;
2254265 176 64 127;
2254265 176 64 0;
2254270 176 88 81;
2254270 176 88 93;
2254270 144 63 38;
2254286 176 88 93;
2254286 176 88 109;
2254286 144 27 32;
2254296 176 88 109;
2254296 176 88 25;
2254296 144 39 30;
2254802 176 64 0;
2254802 176 64 127;
2256416 128 39 64;
2256645 128 27 64;
2257104 128 63 64;
2257140 176 88 25;
2257140 176 88 59;
2257140 144 61 17;
2257760 128 61 64;
2257796 176 88 59;
2257796 176 88 63;
2257796 144 60 27;
2258604 128 60 64;
2258812 176 88 63;
2258812 176 88 123;
2258812 144 65 3;
2260359 128 65 64;
2260401 176 88 123;
2260401 176 88 119;
2260401 144 66 23;
2260468 176 64 127;
2260468 176 64 0;
2260489 176 88 119;
2260489 176 88 26;
2260489 144 61 20;
2260578 176 64 0;
2260578 176 64 127;
2260973 128 61 64;
2261000 128 66 64;
2261369 176 88 26;
2261369 176 88 27;
2261369 144 66 32;
2261385 176 88 27;
2261385 176 88 87;
2261385 144 61 19;
2261489 128 66 64;
2261494 128 61 64;
2262197 176 88 87;
2262197 176 88 110;
2262197 144 66 27;
2262244 176 88 110;
2262244 176 88 120;
2262244 144 61 13;
2262312 128 66 64;
2262328 128 61 64;
2263114 176 88 120;
2263114 176 88 15;
2263114 144 65 30;
2263177 176 88 15;
2263177 176 88 13;
2263177 144 60 22;
2263182 176 64 127;
2263182 176 64 0;
2263302 176 64 0;
2263302 176 64 127;
2263593 128 60 64;
2264156 128 65 64;
2264796 176 88 13;
2264796 176 88 14;

2264796 144 66 41;
2264817 176 88 14;
2264817 176 88 50;
2264817 144 61 29;
2265026 128 61 64;
2265067 128 66 64;
2265359 176 88 50;
2265359 176 88 62;
2265359 144 66 51;
2265385 176 88 62;
2265385 176 88 127;
2265385 144 61 35;
2265458 128 61 64;
2265463 128 66 64;
2265750 176 88 127;
2265750 176 88 52;
2265750 144 61 47;
2265755 176 88 52;
2265755 176 88 51;
2265755 144 66 62;
2265812 128 61 64;
2265843 128 66 64;
2266156 176 88 51;
2266156 176 88 47;
2266156 144 65 71;
2266166 176 88 47;
2266166 176 88 94;
2266166 144 60 53;
2266312 128 60 64;
2266322 128 65 64;
2266734 176 88 94;
2266734 176 88 25;
2266734 144 78 65;
2266739 176 88 25;
2266739 176 88 4;
2266739 144 73 61;
2266880 128 73 64;
2266906 128 78 64;
2267078 176 88 4;
2267078 176 88 120;
2267078 144 78 78;
2267083 176 88 120;
2267083 176 88 16;
2267083 144 73 60;
2267171 128 73 64;
2267208 128 78 64;
2267364 176 88 16;
2267364 176 88 59;
2267364 144 73 67;
2267380 176 88 59;
2267380 176 88 87;
2267380 144 78 80;
2267453 128 73 64;
2267531 128 78 64;
2267677 176 88 87;
2267677 176 88 82;
2267677 144 72 75;
2267687 176 88 82;
2267687 176 88 91;
2267687 144 77 49;
2267708 176 88 91;
2267708 176 88 43;
2267708 144 79 46;
2267760 128 79 64;
2267828 128 72 64;
2267838 128 77 64;
2268385 176 88 43;
2268385 176 88 93;
2268385 144 90 77;
2268401 176 88 93;
2268401 176 88 21;
2268401 144 85 54;
2268802 128 85 64;
2268854 128 90 64;
2269098 176 88 21;
2269098 176 88 66;
2269098 144 90 51;
2269109 176 88 66;
2269109 176 88 79;
2269109 144 85 34;
2269270 128 85 64;
2269270 128 90 64;
2269937 176 88 79;
2269937 176 88 122;
2269937 144 89 40;
2269953 176 88 122;
2269953 176 88 52;
2269953 144 84 35;
2270223 128 84 64;
2271510 128 89 64;
2271562 176 88 52;
2271562 176 88 53;
2271562 144 87 31;
2271572 176 88 53;
2271572 176 88 32;
2271572 144 82 21;
2271697 128 82 64;
2271947 176 88 32;
2271947 176 88 70;
2271947 144 80 37;
2271968 176 88 70;
2271968 176 88 63;
2271968 144 85 32;
2272000 128 87 64;
2272328 128 80 64;
2272437 176 88 63;
2272437 176 88 109;
2272437 144 78 39;
2272447 176 88 109;
2272447 176 88 20;
2272447 144 82 39;
2272468 128 85 64;
2273161 128 78 64;
2273380 128 82 64;
2275171 176 88 20;
2275171 176 88 36;
2275171 144 27 27;
2275171 144 75 29;
2275182 176 88 36;
2275182 176 88 30;
2275182 144 39 26;
2276296 128 75 64;
2276437 128 39 64;
2276625 128 27 64;
2276661 176 88 30;
2276661 176 88 17;
2276661 144 75 27;
2276677 176 88 17;
2276677 176 88 99;
2276677 144 87 17;
2276750 128 75 64;
2276781 128 87 64;
2277057 176 88 99;
2277057 176 88 110;
2277057 144 78 36;
2277067 176 88 110;
2277067 176 88 15;
2277067 144 90 33;
2277119 128 78 64;
2277145 128 90 64;
2277427 176 88 15;
2277427 176 88 33;
2277427 144 80 40;
2277437 176 88 33;
2277437 176 88 80;
2277437 144 92 36;
2277500 128 80 64;
2277526 128 92 64;
2277937 176 88 80;
2277937 176 88 94;
2277937 144 94 22;
2277953 176 88 94;
2277953 176 88 21;
2277953 144 82 16;
2278020 128 82 64;
2278031 128 94 64;
2278416 176 88 21;
2278416 176 88 80;
2278416 144 92 36;
2278421 176 88 80;
2278421 176 88 97;
2278421 144 80 31;
2278520 128 80 64;
2278536 128 92 64;
2279437 176 88 97;
2279437 176 88 6;
2279437 144 78 29;
2279437 144 90 27;
2279567 128 78 64;
2279578 128 90 64;
2282546 176 88 6;
2282546 176 88 84;
2282546 144 78 20;
2282546 144 90 23;
2282677 128 78 64;
2282682 128 90 64;
2285057 176 88 84;
2285057 176 88 33;
2285057 144 75 19;
2285078 176 88 33;
2285078 176 88 59;
2285078 144 39 15;
2285078 144 87 16;
2285088 176 88 59;
2285088 176 88 38;
2285088 144 27 16;
2285317 128 75 64;
2285359 128 87 64;
2286145 176 88 38;
2286145 176 88 117;
2286145 144 89 32;
2286156 176 88 117;
2286156 176 88 40;
2286156 144 77 27;
2286265 128 89 64;
2286265 128 77 64;
2286281 128 39 64;
2286401 128 27 64;
2287057 176 88 40;
2287057 176 88 55;
2287057 144 96 55;
2287130 128 96 64;
2287531 176 88 55;
2287531 176 88 99;
2287531 144 97 44;
2287546 176 88 99;
2287546 176 88 17;
2287546 144 85 27;
2287604 128 97 64;
2287630 128 85 64;
2287937 176 88 17;
2287937 176 88 122;
2287937 144 84 39;
2287958 176 88 122;
2287958 176 88 2;
2287958 144 96 31;
2288010 128 84 64;
2288046 128 96 64;
2288281 176 88 2;
2288281 176 88 124;
2288281 144 97 41;
2288296 176 88 124;
2288296 176 88 13;
2288296 144 85 24;
2288348 128 97 64;
2288375 128 85 64;
2288578 176 88 13;
2288578 176 88 50;
2288578 144 99 63;
2288583 176 88 50;
2288583 176 88 45;
2288583 144 87 42;
2288635 128 99 64;
2288651 128 87 64;
2288848 176 88 45;
2288848 176 88 24;
2288848 144 97 40;
2288869 176 88 24;
2288869 176 88 43;
2288869 144 85 21;
2288921 128 97 64;
2288942 128 85 64;
2289239 176 88 43;
2289239 176 88 79;
2289239 144 84 43;
2289239 144 96 43;
2289338 128 84 64;
2289343 128 96 64;
2289697 176 88 79;
2289697 176 88 66;
2289697 144 85 38;
2289703 176 88 66;
2289703 176 88 93;
2289703 144 97 37;
2289796 128 85 64;
2289807 128 97 64;
2291838 176 88 93;
2291838 176 88 5;
2291838 144 84 24;
2291848 176 88 5;
2291848 176 88 101;
2291848 144 72 24;
2291979 128 72 64;
2291989 128 84 64;
2294354 176 88 101;
2294354 176 88 78;
2294354 144 85 16;
2294489 128 85 64;
2295203 176 88 78;
2295203 176 88 59;
2295203 144 73 25;
2295239 176 88 59;
2295239 176 88 12;
2295239 144 85 17;
2295359 128 73 64;
2295369 128 85 64;
2296322 176 88 12;
2296322 176 88 42;
2296322 144 73 22;
2296359 176 88 42;
2296359 176 88 71;
2296359 144 85 14;
2296442 128 73 64;
2296473 128 85 64;
2297515 176 88 71;
2297515 176 88 113;
2297515 144 73 22;
2297546 176 88 113;
2297546 176 88 103;
2297546 144 27 16;
2297557 176 88 103;
2297557 176 88 106;
2297557 144 85 17;
2297557 176 88 106;
2297557 176 88 90;
2297557 144 39 17;
2297791 128 73 64;
2297812 128 85 64;
2298838 176 88 90;
2298838 176 88 123;
2298838 144 75 23;
2298838 176 88 123;
2298838 176 88 109;
2298838 144 87 22;
2298994 128 75 64;
2299000 128 87 64;
2299421 128 39 64;
2299895 128 27 64;
2301656 176 88 109;
2301656 176 88 99;
2301656 144 87 28;
2301671 176 88 99;
2301671 176 88 50;
2301671 144 75 27;
2301796 128 75 64;
2301802 128 87 64;
2304958 176 88 50;
2304958 176 88 85;
2304958 144 82 21;
2306239 176 88 85;
2306239 176 88 96;
2306239 144 77 26;
2306255 128 82 64;
2307682 128 77 64;
2307729 176 88 96;
2307729 176 88 16;
2307729 144 80 20;
2309687 176 88 16;
2309687 176 88 50;
2309687 144 75 24;
2309807 128 80 64;
2309932 176 88 50;
2309932 176 88 0;
2309932 144 39 1;
2309979 144 27 1;
2311093 128 75 64;
2311119 176 88 0;
2311119 176 88 10;
2311119 144 82 23;
2312473 128 82 64;
2312494 176 88 10;
2312494 176 88 86;

2312494 144 78 21;
2313359 176 88 86;
2313359 176 88 15;
2313359 144 80 34;
2313390 128 78 64;
2314062 128 39 64;
2314088 128 27 64;
2314781 176 88 15;
2314781 176 88 108;
2314781 144 75 34;
2314817 176 88 108;
2314817 176 88 10;
2314817 144 27 17;
2314822 176 88 10;
2314822 176 88 81;
2314822 144 39 20;
2314921 128 80 64;
2317192 176 88 81;
2317192 176 88 120;
2317192 144 82 22;
2317213 128 75 64;
2318244 176 88 120;
2318244 176 88 35;
2318244 144 78 26;
2318427 128 82 64;
2319015 176 88 35;
2319015 176 88 83;
2319015 144 80 39;
2319114 128 78 64;
2320203 176 88 83;
2320203 176 88 107;
2320203 144 77 29;
2320250 128 80 64;
2321708 128 77 64;
2321723 176 88 107;
2321723 176 88 41;
2321723 144 75 23;
2322401 128 39 64;
2322437 128 27 64;
2323265 176 88 41;
2323265 176 88 73;
2323265 144 27 20;
2323265 144 73 34;
2323286 176 88 73;
2323286 176 88 123;
2323286 144 39 23;
2323296 128 75 64;
2324093 176 88 123;
2324093 176 88 62;
2324093 144 75 36;
2324130 128 73 64;
2325010 176 88 62;
2325010 176 88 108;
2325010 144 72 30;
2325036 128 75 64;
2326119 128 72 64;
2326177 176 88 108;
2326177 176 88 77;
2326177 144 73 15;
2327156 128 39 64;
2327244 128 27 64;
2328093 176 88 77;
2328093 176 88 115;
2328093 144 27 22;
2328104 176 88 115;
2328104 176 88 83;
2328104 144 39 24;
2328109 176 88 83;
2328109 176 88 20;
2328109 128 73 64;
2328109 144 70 31;
2328135 176 88 20;
2328135 176 88 39;
2328135 144 75 35;
2328276 128 70 64;
2328286 128 75 64;
2328557 176 88 39;
2328557 176 88 91;
2328557 144 68 30;
2328567 176 88 91;
2328567 176 88 25;
2328567 144 73 39;
2328630 128 73 64;
2328645 128 68 64;
2328911 176 88 25;
2328911 176 88 43;
2328911 144 66 39;
2328932 176 88 43;
2328932 176 88 10;
2328932 144 70 35;
2328984 128 66 64;
2329000 128 70 64;
2329312 176 88 10;
2329312 176 88 64;
2329312 144 63 36;
2329322 176 88 64;
2329322 176 88 66;
2329322 144 68 36;
2329385 128 63 64;
2329390 128 68 64;
2329760 176 88 66;
2329760 176 88 18;
2329760 144 70 43;
2329786 176 88 18;
2329786 176 88 8;
2329786 144 66 19;
2329843 128 70 64;
2329859 128 66 64;
2330208 176 88 8;
2330208 176 88 42;
2330208 144 68 41;
2330223 176 88 42;
2330223 176 88 115;
2330223 144 73 41;
2330302 128 73 64;
2330312 128 68 64;
2330395 128 39 64;
2330473 128 27 64;
2330833 176 88 115;
2330833 176 88 76;
2330833 144 70 30;
2330843 176 88 76;
2330843 176 88 43;
2330843 144 39 25;
2330843 144 75 37;
2330848 176 88 43;
2330848 176 88 31;
2330848 144 27 21;
2331114 128 70 64;
2331130 128 75 64;
2332427 176 88 31;
2332427 176 88 24;
2332427 144 72 33;
2332437 176 88 24;
2332437 176 88 50;
2332437 144 75 32;
2332536 128 75 64;
2332562 128 72 64;
2332791 176 88 50;
2332791 176 88 42;
2332791 144 75 56;
2332822 176 88 42;
2332822 176 88 65;
2332822 144 72 39;
2332901 128 72 64;
2332937 128 75 64;
2333109 176 88 65;
2333109 176 88 42;
2333109 144 77 65;
2333114 176 88 42;
2333114 176 88 86;
2333114 144 72 58;
2333286 128 72 64;
2333302 128 77 64;
2333416 176 88 86;
2333416 176 88 15;
2333416 144 77 51;
2333427 176 88 15;
2333427 176 88 64;
2333427 144 72 56;
2333588 128 72 64;
2333588 128 77 64;
2333713 176 88 64;
2333713 176 88 111;
2333713 144 75 71;
2333723 176 88 111;
2333723 176 88 39;
2333723 144 72 50;
2333786 128 72 64;
2333786 128 75 64;
2334041 176 88 39;
2334041 176 88 89;
2334041 144 75 61;
2334052 176 88 89;
2334052 176 88 58;
2334052 144 72 51;
2334114 128 72 64;
2334130 128 75 64;
2334447 176 88 58;
2334447 176 88 97;
2334447 144 77 46;
2334453 176 88 97;
2334453 176 88 82;
2334453 144 72 39;
2334531 128 72 64;
2334546 128 77 64;
2334947 176 88 82;
2334947 176 88 78;
2334947 144 77 34;
2334994 176 88 78;
2334994 176 88 71;
2334994 144 72 21;
2335010 128 39 64;
2335083 128 27 64;
2335151 128 72 64;
2335166 128 77 64;
2336005 176 88 71;
2336005 176 88 81;
2336005 144 27 29;
2336036 176 88 81;
2336036 176 88 26;
2336036 144 39 18;
2336036 144 73 30;
2336052 176 88 26;
2336052 176 88 78;
2336052 144 68 25;
2336328 128 73 64;
2336385 128 68 64;
2336458 176 88 78;
2336458 176 88 96;
2336458 144 66 29;
2336473 144 70 24;
2336541 128 66 64;
2336796 128 70 64;
2336817 176 88 96;
2336817 176 88 17;
2336817 144 63 36;
2336854 176 88 17;
2336854 176 88 61;
2336854 144 68 34;
2336921 128 63 64;
2336994 128 68 64;
2337125 176 88 61;
2337125 176 88 109;
2337125 144 66 37;
2337130 176 88 109;
2337130 176 88 95;
2337130 144 70 46;
2337208 128 66 64;
2337260 128 70 64;
2337515 176 88 95;
2337515 176 88 123;
2337515 144 73 45;
2337531 176 88 123;
2337531 176 88 62;
2337531 144 68 37;
2337614 128 68 64;
2337666 128 73 64;
2337885 176 88 62;
2337885 176 88 93;
2337885 144 75 42;
2337895 176 88 93;
2337895 176 88 53;
2337895 144 70 34;
2337947 128 75 64;
2337963 128 70 64;
2338151 128 39 64;
2338171 128 27 64;
2338640 176 88 53;
2338640 176 88 57;
2338640 144 78 30;
2338651 176 88 57;
2338651 176 88 97;
2338651 144 39 16;
2338656 176 88 97;
2338656 176 88 48;
2338656 144 27 11;
2338656 176 88 48;
2338656 176 88 39;
2338656 144 73 28;
2338802 128 73 64;
2338822 128 78 64;
2338854 128 39 64;
2338880 128 27 64;
2339203 176 88 39;
2339203 176 88 94;
2339203 144 77 44;
2339213 176 88 94;
2339213 176 88 33;
2339213 144 72 34;
2339223 176 88 33;
2339223 176 88 106;
2339223 144 27 22;
2339244 176 88 106;
2339244 176 88 84;
2339244 144 39 17;
2339328 128 77 64;
2339343 128 72 64;
2339364 128 39 64;
2339369 128 27 64;
2339598 176 88 84;
2339598 176 88 116;
2339598 144 73 39;
2339609 176 88 116;
2339609 176 88 28;
2339609 144 78 44;
2339619 176 88 28;
2339619 176 88 32;
2339619 144 27 36;
2339630 176 88 32;
2339630 176 88 95;
2339630 144 39 28;
2339760 128 78 64;
2339770 128 73 64;
2339776 128 39 64;
2339802 128 27 64;
2339932 176 88 95;
2339932 176 88 63;
2339932 144 72 53;
2339947 176 88 63;
2339947 176 88 119;
2339947 144 27 31;
2339947 144 77 50;
2339973 176 88 119;
2339973 176 88 34;
2339973 144 39 28;
2340015 128 77 64;
2340052 128 27 64;
2340052 128 72 64;
2340062 128 39 64;
2340270 176 88 34;
2340270 176 88 58;
2340270 144 73 47;
2340281 176 88 58;
2340281 176 88 28;
2340281 144 78 44;
2340286 176 88 28;
2340286 176 88 94;
2340286 144 27 40;
2340286 144 39 33;
2340369 128 78 64;
2340390 128 73 64;
2340406 128 39 64;
2340427 128 27 64;
2340697 176 88 94;
2340697 176 88 97;
2340697 144 72 48;
2340697 144 77 46;
2340713 176 88 97;
2340713 176 88 58;
2340713 144 27 29;
2340739 176 88 58;
2340739 176 88 108;
2340739 144 39 22;
2340776 128 77 64;
2340776 128 72 64;
2340807 128 27 64;
2340833 128 39 64;
2341255 176 88 108;
2341255 176 88 106;
2341255 144 27 29;
2341255 144 78 38;

2341270 176 88 106;
2341270 176 88 3;
2341270 144 73 29;
2341286 176 88 3;
2341286 176 88 8;
2341286 144 39 23;
2341348 128 73 64;
2341406 128 78 64;
2341791 176 88 8;
2341791 176 88 94;
2341791 144 78 36;
2341859 128 78 64;
2342317 176 88 94;
2342317 176 88 110;
2342317 144 90 36;
2342406 128 90 64;
2342994 176 88 110;
2342994 176 88 33;
2342994 144 82 37;
2343135 128 82 64;
2343807 176 88 33;
2343807 176 88 100;
2343807 144 94 19;
2344036 128 94 64;
2346380 176 88 100;
2346380 176 88 31;
2346380 144 92 25;
2351541 176 88 31;
2351541 176 88 40;
2351541 144 90 17;
2351625 128 92 64;
2354083 128 27 64;
2354161 128 39 64;
2357098 128 90 64;
2357546 176 64 127;
2357546 176 64 0;
2517718 176 88 40;
2517718 176 88 91;
2517718 144 27 19;
2517755 176 88 91;
2517755 176 88 125;
2517755 144 39 21;
2517760 176 88 125;
2517760 176 88 47;
2517760 144 75 30;
2517776 176 88 47;
2517776 176 88 57;
2517776 144 87 26;
2518151 128 75 64;
2518156 128 87 64;
2521333 128 39 64;
2521354 128 27 64;
2626890 176 64 0;
2626890 176 64 127;
2627052 176 88 57;
2627052 176 88 44;
2627052 144 65 63;
2627052 144 69 66;
2627052 144 72 60;
2627057 176 88 44;
2627057 176 88 36;
2627057 144 71 59;
2627057 176 88 36;
2627057 176 88 93;
2627057 144 68 42;
2627057 144 70 47;
2627062 176 88 93;
2627062 176 88 105;
2627062 144 64 78;
2627062 176 88 105;
2627062 176 88 114;
2627062 144 50 57;
2627062 144 52 58;
2627062 144 67 69;
2627067 176 88 114;
2627067 176 88 14;
2627067 144 53 48;
2627067 176 88 14;
2627067 176 88 120;
2627067 144 74 77;
2627072 176 88 120;
2627072 176 88 29;
2627072 144 57 49;
2627072 176 88 29;
2627072 176 88 47;
2627072 144 55 44;
2627078 176 88 47;
2627078 176 88 60;
2627078 144 59 54;
2627083 176 88 60;
2627083 176 88 68;
2627083 144 51 35;
2627125 128 67 64;
2627125 128 71 64;
2627130 128 55 64;
2627130 128 53 64;
2627130 128 69 64;
2627140 128 52 64;
2627140 128 59 64;
2627156 128 57 64;
2627156 128 72 64;
2627156 128 74 64;
2627156 128 50 64;
2627156 128 68 64;
2627161 128 51 64;
2627182 128 64 64;
2627203 128 65 64;
2627213 128 70 64;
2627692 176 88 68;
2627692 176 88 92;
2627692 144 39 71;
2627692 144 74 95;
2627697 176 88 92;
2627697 176 88 43;
2627697 144 45 85;
2627697 144 77 98;
2627697 176 88 43;
2627697 176 88 34;
2627697 144 40 78;
2627697 144 76 99;
2627708 176 88 34;
2627708 176 88 95;
2627708 144 36 70;
2627708 144 38 80;
2627708 144 41 87;
2627708 144 43 84;
2627708 144 47 86;
2627708 144 79 89;
2627708 144 80 60;
2627708 144 81 59;
2627708 176 88 95;
2627708 176 88 43;
2627708 144 42 43;
2627718 176 88 43;
2627718 176 88 59;
2627718 144 82 46;
2627718 144 83 81;
2627718 144 84 81;
2627718 176 88 59;
2627718 176 88 45;
2627723 144 85 50;
2627723 176 88 45;
2627723 176 88 83;
2627723 144 72 52;
2627755 176 88 83;
2627755 176 88 7;
2627755 128 76 64;
2627755 144 76 38;
2627822 176 88 7;
2627822 176 88 80;
2627822 128 85 64;
2627822 144 85 14;
2627828 128 79 64;
2627843 176 88 80;
2627843 176 88 110;
2627843 128 82 64;
2627843 144 82 8;
2627864 128 41 64;
2627864 128 42 64;
2627864 128 77 64;
2627864 128 81 64;
2627864 128 43 64;
2627869 128 83 64;
2627875 128 40 64;
2627875 128 45 64;
2627880 128 38 64;
2627880 128 76 64;
2627885 128 80 64;
2627885 128 72 64;
2627895 128 36 64;
2627895 128 47 64;
2627901 128 84 64;
2627911 128 39 64;
2627911 128 74 64;
2627911 128 82 64;
2627921 128 85 64;
2628489 176 88 110;
2628489 176 88 61;
2628489 144 24 85;
2628489 144 26 74;
2628489 144 27 51;
2628489 144 28 62;
2628489 144 29 39;
2628489 144 31 48;
2628489 144 32 64;
2628489 144 33 73;
2628489 144 35 92;
2628489 144 36 82;
2628489 144 84 69;
2628489 144 86 101;
2628489 144 89 99;
2628489 144 90 92;
2628489 144 91 90;
2628489 144 93 73;
2628489 144 94 77;
2628489 144 95 94;
2628489 144 96 85;
2628489 144 98 99;
2628494 176 88 61;
2628494 176 88 54;
2628494 144 88 37;
2628546 128 84 64;
2628687 128 88 64;
2628692 128 93 64;
2628697 128 33 64;
2628697 128 91 64;
2628697 128 29 64;
2628697 128 89 64;
2628703 128 31 64;
2628723 128 28 64;
2628723 128 35 64;
2628750 128 32 64;
2628755 128 98 64;
2628781 128 24 64;
2628781 128 36 64;
2628791 128 95 64;
2628802 128 26 64;
2628807 128 27 64;
2628807 128 96 64;
2628807 128 86 64;
2628817 128 94 64;
2628854 128 90 64;
2629427 176 64 127;
2629427 176 64 0;
2629463 176 88 54;
2629463 176 88 94;
2629463 144 50 80;
2629463 144 58 60;
2629463 144 59 93;
2629463 144 60 89;
2629463 144 62 95;
2629463 144 63 65;
2629463 144 65 79;
2629463 144 69 87;
2629463 144 71 82;
2629468 176 88 94;
2629468 176 88 58;
2629468 144 67 46;
2629479 176 88 58;
2629479 176 88 95;
2629479 144 66 53;
2629479 144 68 45;
2629500 176 88 95;
2629500 176 88 92;
2629500 144 52 26;
2629500 144 55 43;
2629515 128 58 64;
2629526 176 88 92;
2629526 176 88 4;
2629526 128 59 64;
2629526 128 67 64;
2629526 144 59 35;
2629536 176 88 4;
2629536 176 88 1;
2629536 144 53 20;
2629567 128 52 64;
2629572 176 64 0;
2629572 176 64 127;
2629614 128 55 64;
2629619 128 53 64;
2629625 128 65 64;
2629630 128 69 64;
2629755 128 62 64;
2629760 128 59 64;
2629760 128 71 64;
2629765 128 60 64;
2629770 128 68 64;
2629786 128 66 64;
2629791 128 50 64;
2629807 128 63 64;
2630234 176 64 127;
2630234 176 64 0;
2630291 176 88 1;
2630291 176 88 60;
2630291 144 52 99;
2630291 144 61 84;
2630317 176 88 60;
2630317 176 88 126;
2630317 144 38 69;
2630317 144 56 52;
2630322 176 88 126;
2630322 176 88 22;
2630322 144 42 58;
2630328 176 88 22;
2630328 176 88 38;
2630328 144 48 76;
2630328 144 55 52;
2630333 176 88 38;
2630333 176 88 92;
2630333 144 54 21;
2630343 176 88 92;
2630343 176 88 57;
2630343 144 58 38;
2630359 176 88 57;
2630359 176 88 108;
2630359 144 57 27;
2630369 176 88 108;
2630369 176 88 51;
2630369 144 53 47;
2630375 176 88 51;
2630375 176 88 102;
2630375 144 45 38;
2630427 176 64 0;
2630427 176 64 127;
2630458 128 57 64;
2630500 128 55 64;
2630552 128 54 64;
2630609 128 53 64;
2630619 128 45 64;
2630645 128 48 64;
2630656 128 58 64;
2630666 128 61 64;
2630677 128 38 64;
2630682 128 52 64;
2630682 128 42 64;
2630708 128 56 64;
2631239 176 88 102;
2631239 176 88 20;
2631239 144 30 84;
2631244 176 88 20;
2631244 176 88 66;
2631244 144 43 95;
2631244 144 51 74;
2631255 176 88 66;
2631255 176 88 76;
2631255 144 44 68;
2631260 176 88 76;
2631260 176 88 108;
2631260 144 46 57;
2631260 176 88 108;
2631260 176 88 81;
2631260 144 34 62;
2631265 176 64 127;
2631265 176 64 0;
2631281 176 88 81;
2631281 176 88 58;
2631281 144 32 47;
2631281 144 40 54;
2631281 144 47 41;
2631296 176 88 58;
2631296 176 88 85;
2631296 144 45 33;

2631307 176 88 85;
2631307 176 88 92;
2631307 144 38 26;
2631307 144 48 32;
2631307 144 50 22;
2631312 176 88 92;
2631312 176 88 60;
2631312 144 36 30;
2631359 128 45 64;
2631369 128 50 64;
2631380 128 47 64;
2631380 128 38 64;
2631385 128 48 64;
2631442 128 36 64;
2631442 176 64 0;
2631442 176 64 127;
2631723 128 32 64;
2631760 128 44 64;
2631776 128 34 64;
2631796 128 43 64;
2631807 128 40 64;
2631828 128 30 64;
2631875 128 51 64;
2631911 128 46 64;
2633473 176 64 127;
2633473 176 64 0;
2633526 176 88 60;
2633526 176 88 102;
2633526 144 93 100;
2633541 176 88 102;
2633541 176 88 15;
2633541 144 73 74;
2633541 144 78 73;
2633541 144 79 99;
2633541 144 88 101;
2633541 144 96 98;
2633546 176 88 15;
2633546 176 88 32;
2633546 144 86 83;
2633546 144 89 89;
2633546 144 90 46;
2633546 144 91 80;
2633557 176 88 32;
2633557 176 88 8;
2633557 144 80 39;
2633557 144 95 60;
2633562 176 88 8;
2633562 176 88 83;
2633562 144 94 33;
2633588 128 89 64;
2633598 128 86 64;
2633604 128 88 64;
2633604 128 91 64;
2633604 128 90 64;
2633614 128 80 64;
2633625 128 95 64;
2633630 176 64 0;
2633630 176 64 127;
2633645 128 94 64;
2633692 128 93 64;
2633703 128 79 64;
2633729 128 96 64;
2633729 128 78 64;
2633734 128 73 64;
2633963 176 64 127;
2633963 176 64 0;
2634036 176 88 83;
2634036 176 88 3;
2634036 144 96 101;
2634041 176 88 3;
2634041 176 88 95;
2634041 144 73 73;
2634046 176 88 95;
2634046 176 88 30;
2634046 144 80 72;
2634046 144 86 82;
2634046 144 90 73;
2634057 176 88 30;
2634057 176 88 24;
2634057 144 81 70;
2634057 144 94 54;
2634062 176 88 24;
2634062 176 88 48;
2634062 144 78 51;
2634062 144 88 57;
2634062 144 93 57;
2634078 176 88 48;
2634078 176 88 101;
2634078 144 79 38;
2634109 128 88 64;
2634125 128 86 64;
2634135 128 79 64;
2634140 128 93 64;
2634151 128 78 64;
2634151 128 81 64;
2634182 128 94 64;
2634192 128 96 64;
2634192 128 80 64;
2634192 128 73 64;
2634223 128 90 64;
2634359 176 88 101;
2634359 176 88 126;
2634359 144 73 79;
2634364 176 88 126;
2634364 176 88 79;
2634364 144 96 99;
2634364 176 88 79;
2634364 176 88 123;
2634364 144 80 73;
2634369 176 88 123;
2634369 176 88 27;
2634369 144 81 87;
2634375 176 88 27;
2634375 176 88 22;
2634375 144 86 90;
2634375 176 88 22;
2634375 176 88 76;
2634375 144 92 71;
2634395 176 88 76;
2634395 176 88 107;
2634395 144 78 45;
2634401 176 88 107;
2634401 176 88 74;
2634401 144 94 37;
2634411 176 88 74;
2634411 176 88 57;
2634411 144 89 44;
2634463 128 78 64;
2634479 128 89 64;
2634479 128 94 64;
2634489 128 81 64;
2634489 128 96 64;
2634494 128 86 64;
2634510 128 80 64;
2634520 128 92 64;
2634520 128 73 64;
2634630 176 88 57;
2634630 176 88 111;
2634630 144 73 63;
2634635 176 88 111;
2634635 176 88 112;
2634635 144 96 103;
2634651 176 88 112;
2634651 176 88 17;
2634651 144 80 46;
2634651 144 81 85;
2634651 144 86 89;
2634651 144 90 77;
2634651 144 92 80;
2634666 176 88 17;
2634666 176 88 35;
2634666 144 78 46;
2634671 176 88 35;
2634671 176 88 83;
2634671 144 94 41;
2634703 128 86 64;
2634718 128 90 64;
2634734 128 78 64;
2634744 128 94 64;
2634770 128 80 64;
2634781 128 96 64;
2634781 128 81 64;
2634786 128 73 64;
2634796 128 92 64;
2634953 176 88 83;
2634953 176 88 118;
2634953 144 96 102;
2634958 176 88 118;
2634958 176 88 50;
2634958 144 92 84;
2634958 176 88 50;
2634958 176 88 81;
2634958 144 73 71;
2634958 144 86 90;
2634963 176 88 81;
2634963 176 88 17;
2634963 144 81 88;
2634963 144 90 77;
2634973 176 88 17;
2634973 176 88 21;
2634973 144 80 62;
2634973 176 88 21;
2634973 176 88 18;
2634973 144 78 62;
2634989 176 88 18;
2634989 176 88 74;
2634989 144 94 37;
2635078 128 94 64;
2635098 128 81 64;
2635104 128 80 64;
2635119 128 90 64;
2635119 128 86 64;
2635125 128 96 64;
2635135 128 73 64;
2635140 128 92 64;
2635140 128 78 64;
2635317 176 88 74;
2635317 176 88 23;
2635317 144 96 108;
2635328 176 88 23;
2635328 176 88 96;
2635328 144 73 88;
2635328 144 81 103;
2635328 144 86 97;
2635328 144 90 86;
2635328 144 92 84;
2635338 176 88 96;
2635338 176 88 122;
2635338 144 80 67;
2635348 176 88 122;
2635348 176 88 47;
2635348 144 94 43;
2635364 176 88 47;
2635364 176 88 0;
2635364 144 88 39;
2635395 176 88 0;
2635395 176 88 96;
2635395 144 77 28;
2635401 128 80 64;
2635432 128 94 64;
2635442 128 88 64;
2635484 128 77 64;
2635489 128 81 64;
2635500 128 96 64;
2635510 128 86 64;
2635510 128 90 64;
2635515 128 92 64;
2635526 128 73 64;
2635697 176 88 96;
2635697 176 88 7;
2635697 144 73 83;
2635697 144 96 111;
2635703 176 88 7;
2635703 176 88 75;
2635703 144 86 101;
2635703 176 88 75;
2635703 176 88 39;
2635703 144 81 103;
2635708 176 88 39;
2635708 176 88 78;
2635708 144 90 83;
2635708 144 92 86;
2635718 176 88 78;
2635718 176 88 127;
2635718 144 75 58;
2635729 176 88 127;
2635729 176 88 112;
2635729 144 80 61;
2635729 144 94 50;
2635864 128 94 64;
2635885 128 81 64;
2635885 128 75 64;
2635885 128 80 64;
2635911 128 86 64;
2635911 128 96 64;
2635916 128 92 64;
2635921 128 90 64;
2635921 128 73 64;
2636088 176 64 0;
2636088 176 64 127;
2636166 176 88 112;
2636166 176 88 7;
2636166 144 96 111;
2636177 176 88 7;
2636177 176 88 6;
2636177 144 73 64;
2636177 144 81 99;
2636177 144 86 100;
2636177 144 90 89;
2636187 176 88 6;
2636187 176 88 28;
2636187 144 80 61;
2636187 144 94 65;
2636192 176 88 28;
2636192 176 88 18;
2636192 144 93 62;
2636203 176 88 18;
2636203 176 88 86;
2636203 144 72 69;
2636203 144 88 65;
2636203 176 88 86;
2636203 176 88 106;
2636203 144 75 45;
2636255 128 88 64;
2636541 128 72 64;
2637098 128 75 64;
2637098 128 80 64;
2637125 128 73 64;
2637145 128 81 64;
2637171 128 93 64;
2637265 128 86 64;
2637312 128 94 64;
2637328 128 90 64;
2637406 128 96 64;
2637859 176 88 106;
2637859 176 88 96;
2637859 144 101 102;
2637859 144 103 42;
2637890 176 88 96;
2637890 176 88 84;
2637890 144 28 43;
2637890 144 29 89;
2637890 144 100 102;
2637911 144 98 102;
2637916 176 88 84;
2637916 176 88 107;
2637916 144 31 80;
2637921 128 103 64;
2637942 176 88 107;
2637942 176 88 3;
2637942 144 96 101;
2637958 176 88 3;
2637958 176 88 116;
2637958 128 28 64;
2637958 128 101 64;
2637958 144 33 84;
2637968 128 100 64;
2637973 176 88 116;
2637973 176 88 53;
2637973 144 95 101;
2637984 176 88 53;
2637984 176 88 1;
2637984 144 35 94;
2638005 176 88 1;
2638005 176 88 42;
2638005 144 93 100;
2638010 128 98 64;
2638010 128 31 64;
2638015 176 88 42;
2638015 176 88 119;
2638015 128 29 64;
2638015 144 36 84;
2638020 128 33 64;
2638031 176 88 119;
2638031 176 88 82;
2638031 144 91 106;
2638036 128 35 64;
2638046 176 88 82;
2638046 176 88 64;
2638046 128 96 64;
2638046 144 38 82;
2638057 176 88 64;

2638057 176 88 38;
2638057 128 36 64;
2638057 144 89 103;
2638062 128 95 64;
2638072 176 88 38;
2638072 176 88 93;
2638072 144 40 78;
2638078 128 91 64;
2638083 176 88 93;
2638083 176 88 6;
2638083 144 88 102;
2638093 128 38 64;
2638098 176 88 6;
2638098 176 88 99;
2638098 128 89 64;
2638098 144 41 58;
2638104 176 88 99;
2638104 176 88 127;
2638104 144 86 91;
2638114 176 88 127;
2638114 176 88 43;
2638114 128 93 64;
2638114 128 40 64;
2638114 144 43 82;
2638125 176 88 43;
2638125 176 88 29;
2638125 128 88 64;
2638125 144 84 91;
2638135 176 88 29;
2638135 176 88 78;
2638135 144 45 83;
2638145 128 86 64;
2638145 176 88 78;
2638145 176 88 8;
2638145 128 41 64;
2638145 144 83 89;
2638156 128 43 64;
2638161 176 88 8;
2638161 176 88 91;
2638161 144 47 72;
2638166 176 88 91;
2638166 176 88 6;
2638166 128 84 64;
2638166 144 81 82;
2638182 128 45 64;
2638187 176 88 6;
2638187 176 88 2;
2638187 128 83 64;
2638187 144 79 95;
2638192 176 88 2;
2638192 176 88 9;
2638192 144 48 70;
2638208 176 88 9;
2638208 176 88 110;
2638208 128 47 64;
2638208 128 81 64;
2638208 144 77 99;
2638229 176 88 110;
2638229 176 88 121;
2638229 128 79 64;
2638229 144 50 59;
2638234 176 88 121;
2638234 176 88 66;
2638234 144 76 98;
2638244 128 48 64;
2638250 128 77 64;
2638260 176 88 66;
2638260 176 88 82;
2638260 144 74 96;
2638265 176 88 82;
2638265 176 88 99;
2638265 144 52 55;
2638276 128 76 64;
2638281 128 50 64;
2638286 176 88 99;
2638286 176 88 95;
2638286 144 72 96;
2638302 128 74 64;
2638317 176 88 95;
2638317 176 88 61;
2638317 128 52 64;
2638317 144 71 91;
2638328 128 72 64;
2638338 176 88 61;
2638338 176 88 120;
2638338 144 53 22;
2638354 176 88 120;
2638354 176 88 64;
2638354 144 69 58;
2638359 128 71 64;
2638401 128 69 64;
2638401 128 53 64;
2638682 176 64 127;
2638682 176 64 0;
2638692 176 88 64;
2638692 176 88 96;
2638692 144 51 104;
2638692 144 70 109;
2638703 176 88 96;
2638703 176 88 108;
2638703 144 60 113;
2638703 144 62 114;
2638708 176 88 108;
2638708 176 88 7;
2638708 144 61 81;
2638718 176 88 7;
2638718 176 88 98;
2638718 144 64 100;
2638718 144 67 80;
2638723 176 88 98;
2638723 176 88 18;
2638723 144 58 74;
2638723 144 68 59;
2638729 176 88 18;
2638729 176 88 46;
2638729 144 53 69;
2638734 176 88 46;
2638734 176 88 38;
2638734 144 54 34;
2638744 176 88 38;
2638744 176 88 5;
2638744 144 55 64;
2638744 144 66 33;
2638744 144 71 91;
2638755 176 88 5;
2638755 176 88 99;
2638755 144 69 49;
2638776 176 88 99;
2638776 176 88 47;
2638776 128 64 64;
2638776 144 64 53;
2638802 128 54 64;
2639015 128 53 64;
2639020 128 69 64;
2639114 176 64 0;
2639114 176 64 127;
2639151 128 68 64;
2639151 128 66 64;
2639223 128 62 64;
2639244 128 67 64;
2639281 128 55 64;
2639317 128 61 64;
2639317 128 64 64;
2639343 128 70 64;
2639343 128 71 64;
2639343 128 60 64;
2639359 128 58 64;
2639432 128 51 64;
2641151 176 88 47;
2641151 176 88 38;
2641151 144 60 107;
2641171 176 88 38;
2641171 176 88 98;
2641171 144 48 110;
2641187 176 88 98;
2641187 176 88 8;
2641187 144 65 40;
2641223 176 88 8;
2641223 176 88 15;
2641223 144 43 28;
2641255 128 65 64;
2641291 128 43 64;
2641312 128 60 64;
2641317 128 48 64;
2641442 176 64 127;
2641442 176 64 0;
2641453 176 88 15;
2641453 176 88 54;
2641453 144 49 97;
2641463 176 88 54;
2641463 176 88 24;
2641463 144 61 85;
2641489 176 88 24;
2641489 176 88 105;
2641489 144 60 37;
2641546 128 60 64;
2641562 128 49 64;
2641588 128 61 64;
2641656 176 88 105;
2641656 176 88 120;
2641656 144 62 107;
2641656 176 88 120;
2641656 176 88 102;
2641656 144 50 97;
2641770 128 50 64;
2641776 128 62 64;
2641838 176 88 102;
2641838 176 88 38;
2641838 144 51 99;
2641838 144 63 92;
2641890 176 88 38;
2641890 176 88 115;
2641890 144 49 16;
2641947 128 51 64;
2641963 128 49 64;
2641989 176 88 115;
2641989 176 88 49;
2641989 144 64 108;
2641994 176 88 49;
2641994 176 88 13;
2641994 128 63 64;
2641994 144 52 94;
2642104 128 52 64;
2642109 128 64 64;
2642145 176 88 13;
2642145 176 88 28;
2642145 144 53 82;
2642156 176 88 28;
2642156 176 88 3;
2642156 144 65 80;
2642156 144 66 44;
2642182 176 88 3;
2642182 176 88 109;
2642182 144 51 32;
2642218 128 65 64;
2642234 128 53 64;
2642250 176 88 109;
2642250 176 88 96;
2642250 144 65 102;
2642260 176 88 96;
2642260 176 88 92;
2642260 144 53 86;
2642265 128 51 64;
2642265 128 66 64;
2642281 176 64 0;
2642281 176 64 127;
2642364 176 88 92;
2642364 176 88 85;
2642364 144 68 62;
2642364 176 88 85;
2642364 176 88 57;
2642364 144 54 94;
2642395 128 65 64;
2642401 176 88 57;
2642401 176 88 127;
2642401 128 53 64;
2642401 144 67 48;
2642458 128 67 64;
2642479 128 68 64;
2642479 176 88 127;
2642479 176 88 111;
2642479 128 54 64;
2642479 144 67 88;
2642489 176 88 111;
2642489 176 88 3;
2642489 144 55 101;
2642562 176 88 3;
2642562 176 88 115;
2642562 144 70 85;
2642578 176 88 115;
2642578 176 88 56;
2642578 144 54 75;
2642609 128 67 64;
2642630 128 55 64;
2642671 128 70 64;
2642687 176 88 56;
2642687 176 88 59;
2642687 144 69 96;
2642708 176 88 59;
2642708 176 88 94;
2642708 144 55 70;
2642713 128 54 64;
2642781 176 88 94;
2642781 176 88 106;
2642781 144 54 82;
2642807 176 88 106;
2642807 176 88 44;
2642807 144 70 26;
2642828 176 88 44;
2642828 176 88 7;
2642828 128 69 64;
2642828 144 69 25;
2642838 128 55 64;
2642890 128 70 64;
2642895 128 69 64;
2642901 176 88 7;
2642901 176 88 98;
2642901 144 65 83;
2642906 128 54 64;
2642911 176 88 98;
2642911 176 88 114;
2642911 144 67 82;
2642916 176 88 114;
2642916 176 88 66;
2642916 144 53 98;
2642963 128 67 64;
2642984 176 88 66;
2642984 176 88 32;
2642984 144 68 73;
2642994 128 65 64;
2643020 176 88 32;
2643020 176 88 117;
2643020 144 51 56;
2643036 176 88 117;
2643036 176 88 73;
2643036 144 52 48;
2643057 128 53 64;
2643088 128 51 64;
2643093 128 52 64;
2643104 128 68 64;
2643125 176 88 73;
2643125 176 88 96;
2643125 144 53 73;
2643130 176 88 96;
2643130 176 88 42;
2643130 144 65 50;
2643135 144 64 43;
2643177 176 88 42;
2643177 176 88 72;
2643177 144 66 50;
2643177 144 68 51;
2643197 128 65 64;
2643203 128 64 64;
2643213 176 88 72;
2643213 176 88 104;
2643213 144 49 65;
2643234 128 68 64;
2643239 176 88 104;
2643239 176 88 32;
2643239 128 53 64;
2643239 144 50 56;
2643286 128 50 64;
2643291 128 66 64;
2643302 176 88 32;
2643302 176 88 70;
2643302 144 64 25;
2643312 176 88 70;
2643312 176 88 76;
2643312 144 62 65;
2643322 128 49 64;
2643338 176 88 76;
2643338 176 88 29;
2643338 144 52 71;
2643375 128 64 64;
2643390 176 88 29;
2643390 176 88 32;
2643390 144 66 36;
2643421 176 88 32;
2643421 176 88 120;
2643421 144 43 41;
2643421 144 46 65;
2643442 176 88 120;

2643442 176 88 45;
2643442 128 62 64;
2643442 144 48 67;
2643453 128 52 64;
2643489 128 48 64;
2643489 128 43 64;
2643510 128 66 64;
2643515 128 46 64;
2643536 176 88 45;
2643536 176 88 94;
2643536 144 60 77;
2643557 176 88 94;
2643557 176 88 24;
2643557 144 48 51;
2643562 176 88 24;
2643562 176 88 5;
2643562 144 50 46;
2643598 176 88 5;
2643598 176 88 34;
2643598 144 40 78;
2643598 176 88 34;
2643598 176 88 9;
2643598 144 42 81;
2643614 176 88 9;
2643614 176 88 19;
2643614 128 48 64;
2643614 128 50 64;
2643614 144 44 79;
2643640 176 88 19;
2643640 176 88 80;
2643640 144 63 43;
2643656 128 60 64;
2643661 176 88 80;
2643661 176 88 100;
2643661 128 44 64;
2643661 144 62 9;
2643677 128 40 64;
2643703 128 63 64;
2643723 128 62 64;
2643739 176 88 100;
2643739 176 88 116;
2643739 128 42 64;
2643739 144 45 84;
2643755 176 88 116;
2643755 176 88 127;
2643755 144 57 68;
2643755 176 88 127;
2643755 176 88 103;
2643755 144 59 69;
2643770 176 88 103;
2643770 176 88 37;
2643770 144 43 49;
2643822 176 88 37;
2643822 176 88 108;
2643822 144 42 57;
2643822 144 63 18;
2643828 176 88 108;
2643828 176 88 35;
2643828 128 43 64;
2643828 144 58 80;
2643833 128 45 64;
2643838 176 88 35;
2643838 176 88 22;
2643838 144 37 46;
2643843 128 59 64;
2643848 176 88 22;
2643848 176 88 117;
2643848 128 57 64;
2643848 144 36 35;
2643875 128 42 64;
2643890 128 63 64;
2643906 128 58 64;
2643911 176 88 117;
2643911 176 88 16;
2643911 144 56 64;
2643921 128 36 64;
2643921 128 37 64;
2643947 176 88 16;
2643947 176 88 47;
2643947 144 41 63;
2643958 176 88 47;
2643958 176 88 118;
2643958 144 43 54;
2643963 128 56 64;
2643973 176 88 118;
2643973 176 88 94;
2643973 144 55 70;
2644000 176 64 127;
2644000 176 64 0;
2644015 128 43 64;
2644020 176 88 94;
2644020 176 88 4;
2644020 144 37 65;
2644026 176 88 4;
2644026 176 88 46;
2644026 144 56 71;
2644031 176 88 46;
2644031 176 88 101;
2644031 144 33 53;
2644057 176 88 101;
2644057 176 88 69;
2644057 128 41 64;
2644057 144 36 32;
2644104 128 33 64;
2644125 128 36 64;
2644130 128 56 64;
2644151 176 88 69;
2644151 176 88 109;
2644151 144 38 99;
2644161 128 37 64;
2644166 128 55 64;
2644203 176 88 109;
2644203 176 88 127;
2644203 144 50 72;
2644203 144 52 45;
2644265 176 88 127;
2644265 176 88 22;
2644265 144 34 25;
2644276 176 88 22;
2644276 176 88 33;
2644276 144 29 43;
2644276 144 51 93;
2644281 176 88 33;
2644281 176 88 19;
2644281 144 33 58;
2644286 128 38 64;
2644312 176 88 19;
2644312 176 88 76;
2644312 128 50 64;
2644312 144 31 23;
2644348 128 33 64;
2644359 128 29 64;
2644359 176 88 76;
2644359 176 88 1;
2644359 144 36 92;
2644364 128 34 64;
2644375 128 31 64;
2644385 128 52 64;
2644395 176 64 0;
2644395 176 64 127;
2644401 176 88 1;
2644401 176 88 50;
2644401 144 49 27;
2644411 128 51 64;
2644442 176 88 50;
2644442 176 88 19;
2644442 144 47 73;
2644473 176 88 19;
2644473 176 88 1;
2644473 144 32 75;
2644484 176 88 1;
2644484 176 88 2;
2644484 144 28 45;
2644484 144 29 48;
2644500 176 88 2;
2644500 176 88 76;
2644500 128 36 64;
2644500 144 31 43;
2644505 176 88 76;
2644505 176 88 34;
2644505 144 46 77;
2644546 128 29 64;
2644552 128 47 64;
2644562 128 28 64;
2644567 128 31 64;
2644583 128 49 64;
2644588 176 88 34;
2644588 176 88 96;
2644588 144 44 86;
2644598 176 88 96;
2644598 176 88 97;
2644598 144 35 90;
2644614 128 32 64;
2644619 128 46 64;
2644682 128 44 64;
2644692 176 88 97;
2644692 176 88 85;
2644692 144 41 54;
2644692 144 43 54;
2644697 176 88 85;
2644697 176 88 8;
2644697 144 26 83;
2644713 176 88 8;
2644713 176 88 46;
2644713 144 30 77;
2644739 176 88 46;
2644739 176 88 11;
2644739 128 35 64;
2644739 144 42 84;
2644750 128 43 64;
2644765 128 41 64;
2644796 176 88 11;
2644796 176 88 85;
2644796 144 32 65;
2644822 176 88 85;
2644822 176 88 87;
2644822 144 39 80;
2644838 128 30 64;
2644843 176 88 87;
2644843 176 88 30;
2644843 144 33 81;
2644848 128 26 64;
2644848 176 64 127;
2644848 176 64 0;
2644859 128 42 64;
2644875 128 32 64;
2644921 176 88 30;
2644921 176 88 117;
2644921 128 39 64;
2644921 144 38 71;
2644937 176 88 117;
2644937 176 88 46;
2644937 144 24 95;
2644968 128 33 64;
2644979 176 88 46;
2644979 176 88 17;
2644979 144 27 55;
2644994 176 88 17;
2644994 176 88 18;
2644994 144 37 52;
2645057 128 38 64;
2645072 128 24 64;
2645088 176 88 18;
2645088 176 88 104;
2645088 144 36 41;
2645114 176 88 104;
2645114 176 88 73;
2645114 144 31 54;
2645125 128 27 64;
2645151 176 88 73;
2645151 176 88 34;
2645151 144 23 79;
2645166 176 88 34;
2645166 176 88 74;
2645166 144 35 86;
2645171 128 37 64;
2645187 176 88 74;
2645187 176 88 4;
2645187 128 36 64;
2645187 128 31 64;
2645187 144 36 57;
2645307 176 64 0;
2645307 176 64 127;
2645833 128 23 64;
2645848 128 35 64;
2645864 128 36 64;
2647145 176 88 4;
2647145 176 88 85;
2647145 144 96 112;
2647145 144 102 96;
2647145 144 105 110;
2647161 176 88 85;
2647161 176 88 42;
2647161 144 86 105;
2647161 144 100 79;
2647166 176 88 42;
2647166 176 88 34;
2647166 144 87 81;
2647171 176 88 34;
2647171 176 88 74;
2647171 144 103 61;
2647177 176 88 74;
2647177 176 88 123;
2647177 144 85 48;
2647177 144 98 53;
2647182 176 88 123;
2647182 176 88 76;
2647182 144 101 37;
2647197 176 88 76;
2647197 176 88 36;
2647197 144 81 44;
2647203 128 96 64;
2647208 128 100 64;
2647213 176 88 36;
2647213 176 88 86;
2647213 144 84 30;
2647223 128 98 64;
2647244 128 101 64;
2647250 128 103 64;
2647250 128 85 64;
2647265 128 81 64;
2647265 128 86 64;
2647281 128 87 64;
2647291 128 105 64;
2647296 128 84 64;
2647322 128 102 64;
2647520 176 88 86;
2647520 176 88 56;
2647520 144 90 107;
2647520 176 88 56;
2647520 176 88 38;
2647520 144 100 109;
2647531 176 88 38;
2647531 176 88 79;
2647531 144 81 81;
2647531 144 94 80;
2647536 176 88 79;
2647536 176 88 66;
2647536 144 77 75;
2647541 176 88 66;
2647541 176 88 114;
2647541 144 80 45;
2647557 176 88 114;
2647557 176 88 33;
2647557 144 74 50;
2647557 144 76 50;
2647557 144 79 63;
2647557 144 91 59;
2647557 144 93 51;
2647557 144 96 52;
2647567 176 88 33;
2647567 176 88 30;
2647567 144 78 25;
2647588 128 77 64;
2647593 128 76 64;
2647598 128 91 64;
2647598 128 79 64;
2647604 128 93 64;
2647604 128 80 64;
2647609 128 81 64;
2647614 128 74 64;
2647625 128 96 64;
2647635 128 100 64;
2647640 128 78 64;
2647651 128 90 64;
2647671 128 94 64;
2647791 176 88 30;
2647791 176 88 71;
2647791 144 68 87;
2647791 144 73 95;
2647791 144 83 110;
2647791 144 88 101;
2647791 144 91 100;
2647791 144 93 85;
2647807 176 88 71;
2647807 176 88 90;
2647807 144 71 83;
2647807 144 72 81;
2647807 144 76 46;
2647807 144 86 85;
2647807 144 89 83;
2647828 176 88 90;

2647828 176 88 67;
2647828 144 69 30;
2647828 144 74 48;
2647848 128 72 64;
2647848 128 71 64;
2647854 128 89 64;
2647875 128 83 64;
2647875 128 74 64;
2647880 128 76 64;
2647895 128 73 64;
2647895 128 93 64;
2647906 128 69 64;
2647906 128 88 64;
2647911 128 91 64;
2647921 128 86 64;
2647932 128 68 64;
2647973 176 64 127;
2647973 176 64 0;
2648031 176 88 67;
2648031 176 88 21;
2648031 144 71 101;
2648036 176 88 21;
2648036 176 88 50;
2648036 144 87 111;
2648036 176 64 0;
2648036 176 64 127;
2648046 176 88 50;
2648046 176 88 67;
2648046 144 62 87;
2648046 144 67 94;
2648046 144 69 99;
2648046 144 79 114;
2648052 176 88 67;
2648052 176 88 29;
2648052 144 81 100;
2648052 176 88 29;
2648052 176 88 8;
2648052 144 65 70;
2648057 176 88 8;
2648057 176 88 111;
2648057 144 77 85;
2648057 144 85 71;
2648067 176 88 111;
2648067 176 88 77;
2648067 144 83 57;
2648067 144 84 56;
2648083 128 79 64;
2648088 128 67 64;
2648088 128 69 64;
2648093 128 81 64;
2648098 128 65 64;
2648104 128 62 64;
2648109 128 71 64;
2648114 128 84 64;
2648125 128 83 64;
2648130 128 77 64;
2648145 128 85 64;
2648171 128 87 64;
2648208 176 64 127;
2648208 176 64 0;
2648270 176 88 77;
2648270 176 88 90;
2648270 144 78 107;
2648281 176 88 90;
2648281 176 88 117;
2648281 144 57 91;
2648281 144 63 79;
2648281 144 65 102;
2648281 144 72 110;
2648281 144 75 98;
2648286 176 88 117;
2648286 176 88 45;
2648286 144 61 85;
2648286 144 83 85;
2648291 176 88 45;
2648291 176 88 27;
2648291 144 81 87;
2648312 176 64 0;
2648312 176 64 127;
2648317 128 72 64;
2648322 176 88 27;
2648322 176 88 49;
2648322 144 59 26;
2648338 128 81 64;
2648385 176 64 127;
2648385 176 64 0;
2648385 128 59 64;
2648385 128 83 64;
2648390 128 57 64;
2648395 128 65 64;
2648395 128 61 64;
2648401 128 75 64;
2648411 128 78 64;
2648416 128 63 64;
2648536 176 88 49;
2648536 176 88 116;
2648536 144 65 104;
2648536 144 67 100;
2648536 144 73 86;
2648541 176 88 116;
2648541 176 88 37;
2648541 144 71 89;
2648557 176 88 37;
2648557 176 88 31;
2648557 144 47 88;
2648557 144 50 98;
2648557 144 64 85;
2648557 144 66 72;
2648557 144 69 76;
2648557 144 72 65;
2648562 176 88 31;
2648562 176 88 30;
2648562 144 54 59;
2648567 176 88 30;
2648567 176 88 66;
2648567 144 57 74;
2648572 176 88 66;
2648572 176 88 75;
2648572 144 74 73;
2648588 176 88 75;
2648588 176 88 9;
2648588 144 53 34;
2648593 176 88 9;
2648593 176 88 23;
2648593 128 50 64;
2648593 128 71 64;
2648593 144 52 45;
2648609 176 88 23;
2648609 176 88 72;
2648609 128 72 64;
2648609 128 73 64;
2648609 144 73 19;
2648630 128 67 64;
2648635 128 65 64;
2648635 128 57 64;
2648640 128 53 64;
2648656 176 64 0;
2648656 176 64 127;
2648656 128 52 64;
2648661 128 74 64;
2648666 128 73 64;
2648677 128 54 64;
2648682 128 69 64;
2648692 128 47 64;
2648713 128 66 64;
2648718 128 64 64;
2648750 176 64 127;
2648750 176 64 0;
2648843 176 88 72;
2648843 176 88 0;
2648843 144 63 91;
2648854 176 88 0;
2648854 176 88 20;
2648854 144 53 90;
2648854 144 55 95;
2648854 144 56 86;
2648854 144 58 83;
2648864 176 88 20;
2648864 176 88 21;
2648864 144 62 71;
2648864 176 64 0;
2648864 176 64 127;
2648864 176 88 21;
2648864 176 88 35;
2648864 144 40 97;
2648869 176 88 35;
2648869 176 88 48;
2648869 144 43 97;
2648880 176 88 48;
2648880 176 88 36;
2648880 144 44 76;
2648880 144 50 89;
2648880 144 57 47;
2648890 176 88 36;
2648890 176 88 20;
2648890 144 48 58;
2648890 144 60 39;
2648895 176 88 20;
2648895 176 88 79;
2648895 144 46 33;
2648901 176 88 79;
2648901 176 88 54;
2648901 144 59 31;
2648911 176 88 54;
2648911 176 88 42;
2648911 144 47 46;
2648916 128 43 64;
2648932 128 48 64;
2648942 128 57 64;
2648942 128 60 64;
2648953 128 59 64;
2648958 128 62 64;
2648973 128 47 64;
2648984 128 46 64;
2649015 128 55 64;
2649020 128 44 64;
2649026 128 53 64;
2649031 128 50 64;
2649031 176 64 127;
2649031 176 64 0;
2649046 128 58 64;
2649052 128 40 64;
2649052 128 56 64;
2649067 128 63 64;
2649291 176 88 42;
2649291 176 88 109;
2649291 144 26 102;
2649296 176 88 109;
2649296 176 88 19;
2649296 144 37 99;
2649312 176 88 19;
2649312 176 88 34;
2649312 144 79 110;
2649312 144 81 111;
2649312 144 86 97;
2649312 144 87 81;
2649312 144 89 105;
2649317 176 88 34;
2649317 176 88 111;
2649317 144 28 85;
2649317 144 29 64;
2649317 144 32 72;
2649322 176 88 111;
2649322 176 88 105;
2649322 144 83 85;
2649328 176 88 105;
2649328 176 88 76;
2649328 144 84 67;
2649328 176 88 76;
2649328 176 88 107;
2649328 144 31 53;
2649458 128 31 64;
2649479 128 84 64;
2649484 128 81 64;
2649489 128 29 64;
2649505 128 32 64;
2649510 128 28 64;
2649510 128 83 64;
2649515 128 26 64;
2649520 128 86 64;
2649520 128 89 64;
2649526 128 37 64;
2649526 128 87 64;
2649526 128 79 64;
2649765 176 88 107;
2649765 176 88 116;
2649765 144 49 91;
2649770 176 88 116;
2649770 176 88 43;
2649770 144 59 100;
2649776 176 88 43;
2649776 176 88 97;
2649776 144 60 107;
2649781 176 88 97;
2649781 176 88 39;
2649781 144 62 103;
2649781 144 67 95;
2649781 144 70 83;
2649791 176 88 39;
2649791 176 88 20;
2649791 144 54 72;
2649791 144 56 62;
2649791 144 57 98;
2649791 144 64 102;
2649791 144 65 87;
2649796 176 88 20;
2649796 176 88 115;
2649796 144 66 64;
2649802 176 64 0;
2649802 176 64 127;
2649802 176 88 115;
2649802 176 88 72;
2649802 144 52 57;
2649833 176 88 72;
2649833 176 88 54;
2649833 144 63 25;
2649833 176 88 54;
2649833 176 88 45;
2649833 144 53 32;
2649848 176 88 45;
2649848 176 88 16;
2649848 128 64 64;
2649848 144 64 33;
2649890 128 52 64;
2649890 128 53 64;
2649906 128 63 64;
2649932 128 57 64;
2649947 128 67 64;
2649963 128 64 64;
2649984 128 56 64;
2649994 128 59 64;
2649994 128 65 64;
2650000 128 62 64;
2650000 128 54 64;
2650010 128 66 64;
2650010 128 60 64;
2650036 128 49 64;
2650041 128 70 64;
2650151 176 64 127;
2650151 176 64 0;
2650322 176 88 16;
2650322 176 88 37;
2650322 144 28 101;
2650333 176 88 37;
2650333 176 88 32;
2650333 144 95 108;
2650348 176 88 32;
2650348 176 88 26;
2650348 144 30 77;
2650348 144 84 108;
2650348 144 86 107;
2650348 144 91 101;
2650348 144 92 89;
2650359 176 88 26;
2650359 176 88 120;
2650359 144 29 69;
2650359 144 32 59;
2650359 144 36 70;
2650359 144 38 66;
2650359 144 88 83;
2650359 144 89 82;
2650364 176 88 120;
2650364 176 88 0;
2650364 144 35 67;
2650369 176 88 0;
2650369 176 88 51;
2650369 144 31 37;
2650369 144 83 74;
2650375 176 88 51;
2650375 176 88 64;
2650375 144 93 58;
2650380 176 88 64;
2650380 176 88 45;
2650380 144 33 37;
2650416 128 83 64;
2650437 176 64 0;
2650437 176 64 127;
2650531 128 31 64;
2650531 128 35 64;
2650531 128 89 64;
2650536 128 33 64;
2650546 128 36 64;

2650567 128 86 64;
2650572 128 93 64;
2650583 128 29 64;
2650593 128 32 64;
2650593 128 38 64;
2650598 128 30 64;
2650604 128 95 64;
2650614 128 84 64;
2650614 128 91 64;
2650614 128 28 64;
2650614 128 92 64;
2650619 128 88 64;
2651015 176 64 127;
2651015 176 64 0;
2651015 176 88 45;
2651015 176 88 81;
2651015 144 51 98;
2651015 144 59 112;
2651015 144 60 110;
2651015 144 62 105;
2651015 144 67 110;
2651026 176 88 81;
2651026 176 88 19;
2651026 144 64 106;
2651026 144 65 109;
2651026 144 69 88;
2651031 176 88 19;
2651031 176 88 101;
2651031 144 53 90;
2651031 176 88 101;
2651031 176 88 74;
2651031 144 54 71;
2651036 176 88 74;
2651036 176 88 111;
2651036 144 56 71;
2651036 144 58 63;
2651041 176 88 111;
2651041 176 88 75;
2651041 144 66 53;
2651057 176 88 75;
2651057 176 88 59;
2651057 144 63 41;
2651072 176 88 59;
2651072 176 88 111;
2651072 144 57 36;
2651072 176 88 111;
2651072 176 88 105;
2651072 144 55 36;
2651145 128 55 64;
2651145 128 57 64;
2651218 128 63 64;
2651239 176 64 0;
2651239 176 64 127;
2651260 128 53 64;
2651354 128 65 64;
2651411 128 64 64;
2651427 128 67 64;
2651473 128 54 64;
2651484 128 56 64;
2651489 128 66 64;
2651505 128 58 64;
2651505 128 69 64;
2651520 128 59 64;
2651536 128 62 64;
2651588 128 51 64;
2651593 128 60 64;
2653046 176 88 105;
2653046 176 88 96;
2653046 144 31 43;
2653067 176 88 96;
2653067 176 88 117;
2653067 144 32 29;
2653083 176 88 117;
2653083 176 88 99;
2653083 144 30 30;
2653114 128 31 64;
2653145 128 32 64;
2653166 128 30 64;
2653223 176 88 99;
2653223 176 88 86;
2653223 144 45 105;
2653229 176 88 86;
2653229 176 88 108;
2653229 144 46 57;
2653239 176 88 108;
2653239 176 88 118;
2653239 144 47 81;
2653250 176 88 118;
2653250 176 88 0;
2653250 144 48 80;
2653250 144 49 44;
2653250 144 50 58;
2653250 144 51 51;
2653250 144 52 74;
2653281 128 47 64;
2653286 128 48 64;
2653291 128 52 64;
2653296 128 45 64;
2653296 128 50 64;
2653302 128 51 64;
2653302 128 49 64;
2653307 128 46 64;
2653416 176 88 0;
2653416 176 88 122;
2653416 144 44 105;
2653427 176 88 122;
2653427 176 88 14;
2653427 144 43 104;
2653427 144 46 80;
2653479 176 88 14;
2653479 176 88 86;
2653479 144 45 34;
2653489 128 46 64;
2653520 128 44 64;
2653526 176 88 86;
2653526 176 88 115;
2653526 144 60 105;
2653526 144 63 98;
2653531 176 88 115;
2653531 176 88 83;
2653531 128 43 64;
2653531 144 65 98;
2653546 176 88 83;
2653546 176 88 4;
2653546 144 61 64;
2653546 144 64 84;
2653552 176 88 4;
2653552 176 88 25;
2653552 128 45 64;
2653552 144 62 56;
2653567 128 60 64;
2653588 128 64 64;
2653593 128 61 64;
2653593 128 65 64;
2653598 128 62 64;
2653598 176 88 25;
2653598 176 88 116;
2653598 144 59 23;
2653630 128 63 64;
2653640 176 88 116;
2653640 176 88 118;
2653640 144 51 113;
2653671 176 88 118;
2653671 176 88 67;
2653671 128 59 64;
2653671 144 49 68;
2653671 144 52 72;
2653671 144 53 78;
2653671 176 88 67;
2653671 176 88 123;
2653671 144 50 55;
2653677 176 88 123;
2653677 176 88 91;
2653677 144 48 56;
2653708 128 52 64;
2653713 128 50 64;
2653718 128 53 64;
2653723 128 48 64;
2653723 128 49 64;
2653765 176 88 91;
2653765 176 88 23;
2653765 144 73 102;
2653765 176 88 23;
2653765 176 88 42;
2653765 144 69 105;
2653770 176 88 42;
2653770 176 88 48;
2653770 144 70 89;
2653776 176 88 48;
2653776 176 88 114;
2653776 128 51 64;
2653776 144 74 98;
2653781 176 88 114;
2653781 176 88 127;
2653781 144 67 91;
2653781 144 71 80;
2653786 176 88 127;
2653786 176 88 116;
2653786 144 72 79;
2653812 128 69 64;
2653822 128 67 64;
2653822 128 74 64;
2653828 128 71 64;
2653828 128 72 64;
2653838 128 70 64;
2653869 128 73 64;
2653885 176 88 116;
2653885 176 88 17;
2653885 144 61 102;
2653885 144 63 100;
2653911 176 88 17;
2653911 176 88 50;
2653911 144 60 63;
2653916 176 88 50;
2653916 176 88 72;
2653916 144 59 41;
2653916 144 62 58;
2653927 176 88 72;
2653927 176 88 38;
2653927 144 64 34;
2653937 176 88 38;
2653937 176 88 57;
2653937 144 57 38;
2653953 128 60 64;
2653958 128 62 64;
2653963 128 59 64;
2653979 128 63 64;
2653994 176 88 57;
2653994 176 88 112;
2653994 128 61 64;
2653994 144 82 103;
2653994 176 88 112;
2653994 176 88 31;
2653994 128 64 64;
2653994 144 79 105;
2653994 144 80 85;
2653994 144 83 104;
2653994 144 84 109;
2654005 176 88 31;
2654005 176 88 113;
2654005 144 77 81;
2654010 176 88 113;
2654010 176 88 108;
2654010 144 81 64;
2654015 128 57 64;
2654026 128 79 64;
2654031 128 83 64;
2654036 128 80 64;
2654036 128 84 64;
2654046 128 77 64;
2654052 128 81 64;
2654067 128 82 64;
2654104 176 88 108;
2654104 176 88 66;
2654104 144 70 100;
2654114 176 88 66;
2654114 176 88 37;
2654114 144 71 100;
2654114 144 73 95;
2654114 176 88 37;
2654114 176 88 26;
2654114 144 69 91;
2654125 176 88 26;
2654125 176 88 6;
2654125 144 72 82;
2654125 176 88 6;
2654125 176 88 24;
2654125 144 67 64;
2654130 176 88 24;
2654130 176 88 121;
2654130 144 68 46;
2654156 128 71 64;
2654156 128 69 64;
2654171 128 72 64;
2654171 128 67 64;
2654187 128 68 64;
2654203 176 88 121;
2654203 176 88 127;
2654203 128 73 64;
2654203 144 94 113;
2654208 176 88 127;
2654208 176 88 33;
2654208 128 70 64;
2654208 144 89 118;
2654208 144 95 114;
2654218 176 88 33;
2654218 176 88 82;
2654218 144 91 106;
2654218 144 92 98;
2654218 144 93 112;
2654229 176 88 82;
2654229 176 88 106;
2654229 144 96 72;
2654250 128 91 64;
2654250 128 93 64;
2654265 128 89 64;
2654265 176 64 127;
2654265 176 64 0;
2654276 128 96 64;
2654281 128 92 64;
2654291 128 95 64;
2654317 128 94 64;
2654338 176 88 106;
2654338 176 88 78;
2654338 144 80 103;
2654338 176 88 78;
2654338 176 88 39;
2654338 144 82 109;
2654348 176 88 39;
2654348 176 88 35;
2654348 144 79 102;
2654359 176 88 35;
2654359 176 88 88;
2654359 144 77 78;
2654359 144 81 87;
2654364 176 88 88;
2654364 176 88 18;
2654364 144 78 62;
2654375 176 88 18;
2654375 176 88 108;
2654375 144 76 48;
2654375 144 83 57;
2654406 128 81 64;
2654406 128 77 64;
2654411 128 83 64;
2654416 128 76 64;
2654437 128 78 64;
2654447 128 79 64;
2654468 176 64 0;
2654468 176 64 127;
2654473 176 88 108;
2654473 176 88 107;
2654473 144 102 107;
2654479 176 88 107;
2654479 176 88 110;
2654479 144 98 118;
2654489 176 88 110;
2654489 176 88 4;
2654489 144 96 113;
2654489 144 100 107;
2654489 144 101 112;
2654489 144 103 106;
2654489 144 104 84;
2654505 128 82 64;
2654510 128 80 64;
2654526 128 100 64;
2654588 128 101 64;
2654588 128 103 64;
2654598 128 104 64;
2654619 176 88 4;
2654619 176 88 40;
2654619 128 96 64;
2654619 128 98 64;
2654619 144 78 108;
2654619 144 80 98;
2654640 176 88 40;
2654640 176 88 32;
2654640 128 102 64;
2654640 144 82 50;
2654645 176 88 32;
2654645 176 88 16;
2654645 144 79 51;

2654645 176 88 16;
2654645 176 88 90;
2654645 144 77 50;
2654656 176 88 90;
2654656 176 88 83;
2654656 144 81 40;
2654692 128 77 64;
2654697 128 79 64;
2654697 128 82 64;
2654703 128 80 64;
2654713 128 78 64;
2654713 128 81 64;
2654760 176 88 83;
2654760 176 88 59;
2654760 144 88 123;
2654760 144 92 111;
2654760 144 93 109;
2654760 176 88 59;
2654760 176 88 126;
2654760 144 89 110;
2654765 176 88 126;
2654765 176 88 108;
2654765 144 90 81;
2654765 144 91 112;
2654770 176 88 108;
2654770 176 88 8;
2654770 144 94 76;
2654802 128 89 64;
2654802 128 91 64;
2654817 128 90 64;
2654828 128 94 64;
2654838 128 93 64;
2654848 128 88 64;
2654875 128 92 64;
2654880 176 88 8;
2654880 176 88 11;
2654880 144 68 105;
2654885 176 88 11;
2654885 176 88 71;
2654885 144 70 97;
2654890 176 88 71;
2654890 176 88 50;
2654890 144 66 83;
2654901 176 88 50;
2654901 176 88 14;
2654901 144 67 76;
2654901 144 69 66;
2654911 176 88 14;
2654911 176 88 66;
2654911 144 65 58;
2654921 176 88 66;
2654921 176 88 5;
2654921 144 64 43;
2654927 176 88 5;
2654927 176 88 62;
2654927 144 71 34;
2654942 128 67 64;
2654947 128 69 64;
2654947 128 70 64;
2654958 128 65 64;
2654958 128 66 64;
2654958 176 64 127;
2654958 176 64 0;
2654973 128 64 64;
2654973 128 68 64;
2654984 128 71 64;
2655026 176 88 62;
2655026 176 88 15;
2655026 144 76 122;
2655026 144 77 115;
2655026 144 78 114;
2655026 144 79 118;
2655026 144 80 106;
2655026 144 81 114;
2655062 128 77 64;
2655072 128 79 64;
2655109 128 81 64;
2655119 128 76 64;
2655151 176 88 15;
2655151 176 88 89;
2655151 176 88 89;
2655151 176 88 21;
2655151 128 78 64;
2655151 144 54 108;
2655151 144 56 111;
2655161 128 80 64;
2655166 176 88 21;
2655166 176 88 116;
2655166 144 52 93;
2655166 144 53 102;
2655166 144 55 92;
2655166 144 57 99;
2655203 128 55 64;
2655203 128 57 64;
2655208 128 52 64;
2655208 128 53 64;
2655218 176 88 116;
2655218 176 88 35;
2655218 144 59 28;
2655281 128 56 64;
2655286 128 54 64;
2655286 128 59 64;
2655286 176 64 0;
2655286 176 64 127;
2655302 176 88 35;
2655302 176 88 57;
2655302 144 66 94;
2655312 176 88 57;
2655312 176 88 87;
2655312 144 64 109;
2655312 144 65 103;
2655312 144 67 109;
2655312 144 69 104;
2655317 176 88 87;
2655317 176 88 117;
2655317 144 68 74;
2655317 176 88 117;
2655317 176 88 72;
2655317 144 62 91;
2655328 176 88 72;
2655328 176 88 65;
2655328 144 63 47;
2655359 128 65 64;
2655359 128 62 64;
2655364 128 67 64;
2655364 128 69 64;
2655369 128 68 64;
2655390 128 63 64;
2655395 128 64 64;
2655401 176 64 127;
2655401 176 64 0;
2655432 128 66 64;
2655442 176 88 65;
2655442 176 88 103;
2655442 144 42 105;
2655447 176 88 103;
2655447 176 88 54;
2655447 144 40 102;
2655458 176 88 54;
2655458 176 88 62;
2655458 144 41 103;
2655458 144 45 97;
2655458 144 43 96;
2655468 176 88 62;
2655468 176 88 81;
2655468 144 39 48;
2655500 128 43 64;
2655500 128 45 64;
2655541 128 39 64;
2655578 128 41 64;
2655588 128 40 64;
2655593 176 88 81;
2655593 176 88 82;
2655593 144 51 109;
2655598 176 88 82;
2655598 176 88 56;
2655598 144 55 114;
2655598 176 88 56;
2655598 176 88 47;
2655598 144 52 105;
2655598 176 88 47;
2655598 176 88 124;
2655598 144 53 104;
2655604 176 88 124;
2655604 176 88 75;
2655604 128 42 64;
2655604 144 50 94;
2655609 176 88 75;
2655609 176 88 18;
2655609 144 54 77;
2655614 176 88 18;
2655614 176 88 5;
2655614 144 57 91;
2655635 176 88 5;
2655635 176 88 83;
2655635 144 49 35;
2655640 128 53 64;
2655645 128 50 64;
2655656 128 57 64;
2655661 128 54 64;
2655682 128 52 64;
2655713 128 55 64;
2655729 128 49 64;
2655755 128 51 64;
2655770 176 88 83;
2655770 176 88 30;
2655770 144 30 111;
2655776 176 88 30;
2655776 176 88 85;
2655776 144 29 107;
2655786 176 88 85;
2655786 176 88 53;
2655786 144 28 100;
2655786 144 31 108;
2655791 176 88 53;
2655791 176 88 109;
2655791 144 32 82;
2655791 176 88 109;
2655791 176 88 41;
2655791 144 33 80;
2655796 176 88 41;
2655796 176 88 113;
2655796 144 35 76;
2655807 176 88 113;
2655807 176 88 8;
2655807 144 34 44;
2655843 176 88 8;
2655843 176 88 108;
2655843 128 35 64;
2655843 144 27 19;
2655848 128 33 64;
2655869 176 88 108;
2655869 176 88 1;
2655869 128 28 64;
2655869 128 34 64;
2655869 144 28 32;
2655921 128 31 64;
2655947 128 32 64;
2655979 176 88 1;
2655979 176 88 51;
2655979 144 37 100;
2655994 176 88 51;
2655994 176 88 72;
2655994 128 27 64;
2655994 128 29 64;
2655994 144 38 104;
2655994 144 39 90;
2655994 144 40 101;
2655994 144 41 100;
2655994 144 42 84;
2655994 144 43 101;
2656000 128 28 64;
2656000 176 88 72;
2656000 176 88 30;
2656000 144 36 80;
2656010 128 30 64;
2656223 176 88 30;
2656223 176 88 96;
2656223 144 25 105;
2656229 176 88 96;
2656229 176 88 7;
2656229 144 26 107;
2656229 144 27 95;
2656239 176 88 7;
2656239 176 88 40;
2656239 128 43 64;
2656239 128 41 64;
2656239 144 24 107;
2656239 144 28 113;
2656239 144 29 97;
2656239 144 31 98;
2656255 176 64 0;
2656255 176 64 127;
2656276 128 40 64;
2656281 128 38 64;
2656286 128 42 64;
2656286 128 31 64;
2656291 128 36 64;
2656328 176 88 40;
2656328 176 88 77;
2656328 128 29 64;
2656328 144 29 27;
2656333 176 88 77;
2656333 176 88 7;
2656333 128 39 64;
2656333 128 37 64;
2656333 144 31 39;
2656411 128 31 64;
2656421 128 29 64;
2656432 128 28 64;
2656463 128 27 64;
2656473 128 24 64;
2656479 128 26 64;
2656505 128 25 64;
2657062 176 88 7;
2657062 176 88 76;
2657062 144 95 117;
2657067 176 64 127;
2657067 176 64 0;
2657067 176 88 76;
2657067 176 88 108;
2657067 144 93 116;
2657067 144 98 107;
2657067 144 99 81;
2657067 176 88 108;
2657067 176 88 96;
2657067 144 96 106;
2657078 176 88 96;
2657078 176 88 68;
2657078 144 100 87;
2657078 144 102 67;
2657083 176 88 68;
2657083 176 88 36;
2657083 144 25 93;
2657088 176 88 36;
2657088 176 88 12;
2657088 144 26 99;
2657088 144 28 108;
2657088 144 31 106;
2657093 176 88 12;
2657093 176 88 80;
2657093 144 27 67;
2657093 144 29 102;
2657104 176 88 80;
2657104 176 88 116;
2657104 144 101 54;
2657135 128 29 64;
2657135 176 88 116;
2657135 176 88 123;
2657135 144 24 26;
2657140 128 31 64;
2657151 128 27 64;
2657177 128 101 64;
2657203 128 24 64;
2657208 128 28 64;
2657208 128 96 64;
2657223 128 100 64;
2657229 128 26 64;
2657234 128 95 64;
2657234 128 93 64;
2657239 128 98 64;
2657250 128 25 64;
2657260 128 102 64;
2657270 128 99 64;
2659005 176 64 0;
2659005 176 64 127;
2659182 176 88 123;
2659182 176 88 99;
2659182 144 60 66;
2659208 176 88 99;
2659208 176 88 122;
2659208 144 65 34;
2659213 176 88 122;
2659213 176 88 92;
2659213 144 56 29;
2659218 176 88 92;
2659218 176 88 60;
2659218 144 54 26;
2659223 176 88 60;
2659223 176 88 20;
2659223 144 63 25;
2659255 128 60 64;

2659286 128 65 64;
2659296 128 56 64;
2659307 128 54 64;
2659307 128 63 64;
2659703 176 88 20;
2659703 176 88 36;
2659703 144 60 53;
2659708 176 88 36;
2659708 176 88 98;
2659708 144 66 39;
2659729 176 88 98;
2659729 176 88 37;
2659729 144 54 29;
2659734 176 88 37;
2659734 176 88 116;
2659734 144 56 28;
2659765 128 60 64;
2659776 128 66 64;
2659807 128 54 64;
2659812 128 56 64;
2660208 176 88 116;
2660208 176 88 99;
2660208 144 60 66;
2660213 176 88 99;
2660213 176 88 115;
2660213 144 66 47;
2660234 176 88 115;
2660234 176 88 127;
2660234 144 63 31;
2660276 128 60 64;
2660286 128 66 64;
2660307 128 63 64;
2660812 176 88 127;
2660812 176 88 88;
2660812 144 66 43;
2660833 176 88 88;
2660833 176 88 44;
2660833 144 60 31;
2660848 176 88 44;
2660848 176 88 102;
2660848 144 56 31;
2660848 144 63 23;
2660848 144 68 20;
2660885 128 66 64;
2660911 128 60 64;
2660921 128 68 64;
2660921 128 56 64;
2660932 128 63 64;
2661473 176 88 102;
2661473 176 88 96;
2661473 144 60 47;
2661500 176 88 96;
2661500 176 88 17;
2661500 144 63 31;
2661500 144 66 28;
2661541 176 88 17;
2661541 176 88 77;
2661541 144 54 10;
2661619 128 54 64;
2662255 128 63 64;
2662380 128 66 64;
2662510 128 60 64;
2664223 176 64 127;
2664223 176 64 0;
2664265 176 88 77;
2664265 176 88 109;
2664265 144 51 46;
2664270 176 88 109;
2664270 176 88 126;
2664270 144 75 52;
2664276 176 88 126;
2664276 176 88 125;
2664276 144 73 49;
2664276 176 88 125;
2664276 176 88 6;
2664276 144 49 26;
2664322 128 51 64;
2664328 128 75 64;
2664328 128 73 64;
2664343 128 49 64;
2664656 176 88 6;
2664656 176 88 2;
2664656 144 51 56;
2664661 176 88 2;
2664661 176 88 61;
2664661 144 49 53;
2664671 176 88 61;
2664671 176 88 108;
2664671 144 73 62;
2664671 144 75 66;
2664713 128 51 64;
2664718 128 49 64;
2664718 128 75 64;
2664723 128 73 64;
2664953 176 88 108;
2664953 176 88 12;
2664953 144 49 70;
2664958 176 88 12;
2664958 176 88 39;
2664958 144 73 74;
2664963 176 88 39;
2664963 176 88 37;
2664963 144 75 69;
2664984 176 88 37;
2664984 176 88 81;
2664984 144 51 29;
2665005 128 49 64;
2665015 128 73 64;
2665015 128 75 64;
2665072 128 51 64;
2665213 176 88 81;
2665213 176 88 94;
2665213 144 49 75;
2665229 176 88 94;
2665229 176 88 68;
2665229 144 51 53;
2665229 144 73 69;
2665229 144 75 72;
2665276 128 49 64;
2665281 128 51 64;
2665286 128 73 64;
2665286 128 75 64;
2665541 176 88 68;
2665541 176 88 111;
2665541 144 48 85;
2665552 144 76 80;
2665557 176 88 111;
2665557 176 88 70;
2665557 144 73 70;
2665578 176 88 70;
2665578 176 88 124;
2665578 144 51 38;
2665625 128 73 64;
2665645 128 51 64;
2665661 128 48 64;
2665682 128 76 64;
2665828 176 88 124;
2665828 176 88 69;
2665828 144 51 67;
2665833 176 88 69;
2665833 176 88 74;
2665833 144 73 76;
2665833 176 88 74;
2665833 176 88 45;
2665833 144 76 85;
2665843 176 88 45;
2665843 176 88 57;
2665843 144 48 71;
2665890 128 73 64;
2665895 128 48 64;
2665911 128 76 64;
2665953 128 51 64;
2666083 176 88 57;
2666083 176 88 101;
2666083 144 76 90;
2666088 176 88 101;
2666088 176 88 89;
2666088 144 73 81;
2666088 176 88 89;
2666088 176 88 90;
2666088 144 48 80;
2666104 176 88 90;
2666104 176 88 93;
2666104 144 51 71;
2666187 128 51 64;
2666192 128 73 64;
2666223 128 48 64;
2666239 128 76 64;
2666385 144 49 82;
2666385 144 51 79;
2666385 176 88 93;
2666385 176 88 69;
2666385 144 73 91;
2666395 176 88 69;
2666395 176 88 100;
2666395 144 75 83;
2666520 128 51 64;
2666536 128 75 64;
2666557 128 49 64;
2666562 128 73 64;
2666723 176 88 100;
2666723 176 88 12;
2666723 144 75 96;
2666723 176 88 12;
2666723 176 88 108;
2666723 144 49 85;
2666729 176 88 108;
2666729 176 88 17;
2666729 144 73 85;
2666734 176 88 17;
2666734 176 88 107;
2666734 144 51 76;
2666843 128 51 64;
2666859 128 75 64;
2666875 128 73 64;
2666880 128 49 64;
2667010 176 88 107;
2667010 176 88 59;
2667010 144 75 101;
2667020 176 88 59;
2667020 176 88 31;
2667020 144 49 82;
2667026 176 88 31;
2667026 176 88 14;
2667026 144 51 89;
2667026 144 73 93;
2667177 128 51 64;
2667197 128 49 64;
2667197 128 73 64;
2667218 128 75 64;
2667296 176 88 14;
2667296 176 88 34;
2667296 144 73 82;
2667302 176 88 34;
2667302 176 88 9;
2667302 144 48 91;
2667317 176 88 9;
2667317 176 88 15;
2667317 144 51 79;
2667317 144 76 71;
2667479 128 51 64;
2667489 128 48 64;
2667510 128 73 64;
2667510 128 76 64;
2667593 176 88 15;
2667593 176 88 77;
2667593 144 73 72;
2667593 176 88 77;
2667593 176 88 73;
2667593 144 48 65;
2667598 176 88 73;
2667598 176 88 56;
2667598 144 76 72;
2667604 176 88 56;
2667604 176 88 26;
2667604 144 51 71;
2667765 128 48 64;
2667786 128 51 64;
2667796 128 73 64;
2667796 128 76 64;
2667921 176 88 26;
2667921 176 88 109;
2667921 144 73 78;
2667932 176 88 109;
2667932 176 88 108;
2667932 144 46 77;
2667932 176 88 108;
2667932 176 88 76;
2667932 144 51 68;
2667942 176 88 76;
2667942 176 88 87;
2667942 144 78 80;
2668166 128 51 64;
2668166 128 46 64;
2668187 128 73 64;
2668203 128 78 64;
2668307 176 88 87;
2668307 176 88 59;
2668307 144 46 66;
2668312 176 88 59;
2668312 176 88 95;
2668312 144 78 60;
2668312 176 88 95;
2668312 176 88 76;
2668312 144 51 68;
2668317 176 88 76;
2668317 176 88 56;
2668317 144 73 75;
2668578 128 46 64;
2668583 128 51 64;
2668604 128 73 64;
2668609 128 78 64;
2668765 176 88 56;
2668765 176 88 58;
2668765 144 73 47;
2668765 176 88 58;
2668765 176 88 36;
2668765 144 78 35;
2668776 176 88 36;
2668776 176 88 124;
2668776 144 46 44;
2668776 144 51 44;
2668812 176 64 0;
2668812 176 64 127;
2669010 128 51 64;
2669015 128 46 64;
2669015 128 73 64;
2669020 128 78 64;
2669171 176 88 124;
2669171 176 88 126;
2669171 144 73 79;
2669171 176 88 126;
2669171 176 88 25;
2669171 144 78 77;
2669177 176 88 25;
2669177 176 88 20;
2669177 144 46 72;
2669182 176 88 20;
2669182 176 88 33;
2669182 144 51 72;
2669333 128 51 64;
2669343 128 46 64;
2669364 128 73 64;
2669375 128 78 64;
2669505 176 64 127;
2669505 176 64 0;
2669567 176 88 33;
2669567 176 88 87;
2669567 144 46 72;
2669588 176 88 87;
2669588 176 88 55;
2669588 144 44 45;
2669604 176 88 55;
2669604 176 88 41;
2669604 144 80 51;
2669614 176 88 41;
2669614 176 88 2;
2669614 144 73 30;
2669614 176 88 2;
2669614 176 88 36;
2669614 144 78 38;
2669692 128 78 64;
2669708 128 73 64;
2669713 128 44 64;
2669750 128 46 64;
2669760 128 80 64;
2669958 176 88 36;
2669958 176 88 111;
2669958 144 75 87;
2669968 176 88 111;
2669968 176 88 87;
2669968 144 42 81;
2669968 176 88 87;
2669968 176 88 123;
2669968 144 49 73;
2669979 176 88 123;
2669979 176 88 118;
2669979 144 82 63;
2670005 176 88 118;
2670005 176 88 15;
2670005 144 85 45;
2670130 128 82 64;

2670177 128 49 64;
2670182 128 75 64;
2670187 128 42 64;
2670208 128 85 64;
2670463 176 88 15;
2670463 176 88 27;
2670463 144 78 61;
2670463 176 88 27;
2670463 176 88 72;
2670463 144 46 69;
2670468 176 88 72;
2670468 176 88 17;
2670468 144 90 77;
2670468 176 88 17;
2670468 176 88 106;
2670468 144 39 57;
2670468 144 80 82;
2670505 176 88 106;
2670505 176 88 103;
2670505 144 37 43;
2670505 144 87 33;
2670604 128 87 64;
2670656 128 80 64;
2670666 128 39 64;
2670682 128 46 64;
2670692 128 78 64;
2670692 128 37 64;
2670708 128 90 64;
2670921 176 88 103;
2670921 176 88 90;
2670921 144 82 95;
2670932 176 88 90;
2670932 176 88 46;
2670932 144 94 94;
2670947 176 88 46;
2670947 176 88 61;
2670947 144 42 77;
2670947 176 88 61;
2670947 176 88 15;
2670947 144 30 46;
2670958 176 88 15;
2670958 176 88 30;
2670958 144 32 71;
2671041 176 88 30;
2671041 176 88 100;
2671041 144 34 14;
2671109 128 34 64;
2671213 128 32 64;
2671546 176 64 0;
2671546 176 64 127;
2671947 128 42 64;
2672005 128 30 64;
2672031 128 82 64;
2672036 128 94 64;
2672651 144 99 69;
2672651 176 88 100;
2672651 176 88 107;
2672651 144 27 43;
2672666 176 88 107;
2672666 176 88 3;
2672666 144 30 83;
2672682 176 88 3;
2672682 176 88 124;
2672682 144 97 69;
2672703 176 88 124;
2672703 176 88 13;
2672703 144 32 52;
2672723 128 27 64;
2672739 176 88 13;
2672739 176 88 9;
2672739 144 94 79;
2672760 128 99 64;
2672760 176 88 9;
2672760 176 88 41;
2672760 144 34 76;
2672791 176 88 41;
2672791 176 88 51;
2672791 128 30 64;
2672791 144 92 74;
2672802 128 97 64;
2672807 176 88 51;
2672807 176 88 62;
2672807 144 37 78;
2672817 128 32 64;
2672833 176 88 62;
2672833 176 88 28;
2672833 144 90 84;
2672848 176 88 28;
2672848 176 88 92;
2672848 144 39 71;
2672854 128 34 64;
2672864 128 94 64;
2672880 176 88 92;
2672880 176 88 37;
2672880 144 87 74;
2672890 176 88 37;
2672890 176 88 87;
2672890 144 42 81;
2672895 128 92 64;
2672901 128 37 64;
2672901 128 39 64;
2672911 176 88 87;
2672911 176 88 101;
2672911 144 85 90;
2672921 176 88 101;
2672921 176 88 73;
2672921 144 44 78;
2672932 128 90 64;
2672937 128 42 64;
2672942 128 87 64;
2672947 176 88 73;
2672947 176 88 80;
2672947 144 82 86;
2672947 176 88 80;
2672947 176 88 13;
2672947 144 46 87;
2672973 128 44 64;
2672973 128 85 64;
2672984 176 88 13;
2672984 176 88 84;
2672984 144 80 81;
2672989 176 88 84;
2672989 176 88 36;
2672989 144 49 79;
2673000 128 46 64;
2673010 176 88 36;
2673010 176 88 44;
2673010 144 78 88;
2673020 128 82 64;
2673026 176 88 44;
2673026 176 88 107;
2673026 128 80 64;
2673026 144 51 76;
2673036 176 88 107;
2673036 176 88 69;
2673036 144 75 92;
2673046 176 64 127;
2673046 176 64 0;
2673062 128 78 64;
2673067 176 88 69;
2673067 176 88 18;
2673067 128 49 64;
2673067 144 54 77;
2673072 176 88 18;
2673072 176 88 80;
2673072 144 73 88;
2673109 176 88 80;
2673109 176 88 58;
2673109 144 56 48;
2673114 176 88 58;
2673114 176 88 82;
2673114 128 51 64;
2673114 144 70 87;
2673140 128 75 64;
2673151 176 88 82;
2673151 176 88 60;
2673151 144 58 52;
2673151 144 68 80;
2673161 128 73 64;
2673177 128 54 64;
2673203 176 88 60;
2673203 176 88 50;
2673203 144 66 83;
2673229 128 70 64;
2673776 128 58 64;
2673786 128 66 64;
2673796 128 56 64;
2673807 128 68 64;
2673947 176 64 0;
2673947 176 64 127;
2674177 176 88 50;
2674177 176 88 72;
2674177 144 54 50;
2674177 144 66 67;
2674177 144 68 51;
2674177 144 70 61;
2674192 176 88 72;
2674192 176 88 46;
2674192 144 56 37;
2674203 176 88 46;
2674203 176 88 108;
2674203 144 49 29;
2674203 144 64 33;
2674213 176 88 108;
2674213 176 88 25;
2674213 144 63 29;
2674234 128 70 64;
2674239 128 54 64;
2674239 128 66 64;
2674239 128 68 64;
2674260 128 56 64;
2674276 128 64 64;
2674281 128 49 64;
2674296 128 63 64;
2675739 176 88 25;
2675739 176 88 18;
2675739 144 48 44;
2675739 176 88 18;
2675739 176 88 46;
2675739 144 67 33;
2675750 176 88 46;
2675750 176 88 111;
2675750 144 60 40;
2675755 176 88 111;
2675755 176 88 56;
2675755 144 62 38;
2675760 176 88 56;
2675760 176 88 32;
2675760 144 50 33;
2675760 176 88 32;
2675760 176 88 31;
2675760 144 64 36;
2675770 176 88 31;
2675770 176 88 78;
2675770 144 52 25;
2675781 144 65 25;
2675812 176 88 78;
2675812 176 88 38;
2675812 144 55 24;
2675848 128 64 64;
2675854 128 65 64;
2675854 128 62 64;
2675875 128 60 64;
2675880 128 50 64;
2675885 128 52 64;
2675885 128 67 64;
2675901 128 55 64;
2675911 128 48 64;
2676182 176 88 38;
2676182 176 88 101;
2676182 144 48 55;
2676182 144 67 64;
2676197 176 88 101;
2676197 176 88 5;
2676197 144 50 50;
2676203 176 88 5;
2676203 176 88 73;
2676203 144 62 54;
2676203 176 88 73;
2676203 176 88 74;
2676203 144 60 47;
2676208 176 88 74;
2676208 176 88 2;
2676208 144 52 45;
2676234 176 88 2;
2676234 176 88 83;
2676234 144 64 36;
2676250 176 88 83;
2676250 176 88 69;
2676250 144 55 30;
2676276 176 88 69;
2676276 176 88 21;
2676276 144 53 19;
2676328 128 64 64;
2676338 128 53 64;
2676343 128 55 64;
2676369 128 52 64;
2676375 128 50 64;
2676380 128 48 64;
2676385 128 67 64;
2676390 128 62 64;
2676390 128 60 64;
2676598 176 88 21;
2676598 176 88 2;
2676598 144 67 91;
2676609 176 88 2;
2676609 176 88 31;
2676609 144 60 91;
2676614 176 88 31;
2676614 176 88 7;
2676614 144 62 96;
2676619 176 88 7;
2676619 176 88 30;
2676619 144 48 80;
2676625 176 88 30;
2676625 176 88 93;
2676625 144 64 67;
2676630 176 88 93;
2676630 176 88 111;
2676630 144 52 64;
2676635 176 88 111;
2676635 176 88 109;
2676635 144 50 71;
2676635 144 51 46;
2676640 176 88 109;
2676640 176 88 16;
2676640 144 53 62;
2676640 176 88 16;
2676640 176 88 56;
2676640 144 65 53;
2676666 176 88 56;
2676666 176 88 20;
2676666 144 55 37;
2676697 128 65 64;
2676697 128 64 64;
2676708 128 53 64;
2676723 128 55 64;
2676744 128 52 64;
2676755 128 50 64;
2676755 128 62 64;
2676755 128 60 64;
2676770 128 48 64;
2676776 128 67 64;
2676796 128 51 64;
2676979 176 88 20;
2676979 176 88 47;
2676979 144 69 83;
2676984 176 88 47;
2676984 176 88 94;
2676984 144 62 85;
2676994 176 88 94;
2676994 176 88 119;
2676994 144 64 86;
2676994 144 66 57;
2676994 176 88 119;
2676994 176 88 99;
2676994 144 47 63;
2676994 144 48 82;
2676994 144 50 90;
2676994 144 52 94;
2676994 144 65 81;
2677010 176 88 99;
2677010 176 88 30;
2677010 144 67 60;
2677062 128 67 64;
2677125 128 64 64;
2677130 128 65 64;
2677135 128 52 64;
2677145 128 50 64;
2677151 128 47 64;
2677161 128 62 64;
2677161 176 64 127;
2677161 176 64 0;
2677161 128 48 64;
2677171 128 66 64;
2677171 128 69 64;
2677296 176 64 0;
2677296 176 64 127;
2677317 176 88 30;
2677317 176 88 19;
2677317 144 46 97;
2677317 176 88 19;

2677317 176 88 6;
2677317 144 66 88;
2677322 176 88 6;
2677322 176 88 14;
2677322 144 44 89;
2677328 176 88 14;
2677328 176 88 19;
2677328 144 67 103;
2677328 144 69 96;
2677328 144 74 88;
2677333 176 88 19;
2677333 176 88 70;
2677333 144 71 68;
2677348 176 88 70;
2677348 176 88 52;
2677348 144 41 79;
2677348 144 45 62;
2677348 144 47 53;
2677348 144 48 71;
2677348 144 72 88;
2677354 176 88 52;
2677354 176 88 91;
2677354 144 43 34;
2677385 128 72 64;
2677390 128 47 64;
2677390 128 45 64;
2677411 128 43 64;
2677421 128 71 64;
2677421 176 64 127;
2677421 176 64 0;
2677421 128 48 64;
2677442 128 67 64;
2677447 128 41 64;
2677479 128 46 64;
2677479 128 69 64;
2677494 128 44 64;
2677500 128 74 64;
2677510 128 66 64;
2677588 176 88 91;
2677588 176 88 61;
2677588 144 67 100;
2677598 176 88 61;
2677598 176 88 121;
2677598 144 72 88;
2677598 176 88 121;
2677598 176 88 43;
2677598 144 71 85;
2677604 176 64 0;
2677604 176 64 127;
2677604 176 88 43;
2677604 176 88 59;
2677604 144 69 89;
2677614 176 88 59;
2677614 176 88 77;
2677614 144 40 87;
2677614 144 43 91;
2677614 144 45 97;
2677614 144 47 94;
2677614 144 48 100;
2677651 128 47 64;
2677661 128 48 64;
2677661 128 43 64;
2677687 128 45 64;
2677692 128 72 64;
2677703 128 71 64;
2677703 128 69 64;
2677723 128 40 64;
2677723 128 67 64;
2677776 176 64 127;
2677776 176 64 0;
2677859 176 64 0;
2677859 176 64 127;
2677916 176 88 77;
2677916 176 88 10;
2677916 144 42 78;
2677942 176 88 10;
2677942 176 88 125;
2677942 144 35 56;
2677942 144 36 63;
2677942 144 38 61;
2677942 144 39 32;
2677942 144 40 80;
2677942 144 41 57;
2677942 144 71 91;
2677942 144 73 91;
2677942 144 74 99;
2677942 144 76 94;
2677942 144 77 85;
2677942 144 78 70;
2677942 144 79 81;
2677942 144 81 91;
2677973 176 88 125;
2677973 176 88 121;
2677973 144 43 27;
2677984 128 41 64;
2678000 128 39 64;
2678026 176 64 127;
2678026 176 64 0;
2678026 128 38 64;
2678031 128 74 64;
2678046 128 43 64;
2678052 128 77 64;
2678052 128 35 64;
2678057 128 79 64;
2678062 128 36 64;
2678062 128 40 64;
2678062 128 71 64;
2678067 128 76 64;
2678088 128 78 64;
2678088 128 81 64;
2678093 128 42 64;
2678104 128 73 64;
2678229 176 88 121;
2678229 176 88 118;
2678229 144 41 104;
2678255 176 88 118;
2678255 176 88 51;
2678255 144 31 86;
2678255 144 33 84;
2678255 144 34 84;
2678255 144 36 90;
2678255 144 38 94;
2678255 144 72 95;
2678255 144 74 94;
2678255 144 77 92;
2678255 144 79 100;
2678255 144 80 89;
2678255 144 82 70;
2678255 144 83 74;
2678255 144 84 96;
2678260 176 88 51;
2678260 176 88 122;
2678260 144 35 63;
2678312 176 64 0;
2678312 176 64 127;
2678317 128 35 64;
2678354 128 79 64;
2678354 128 36 64;
2678380 176 64 127;
2678380 176 64 0;
2678395 128 33 64;
2678395 128 38 64;
2678401 128 77 64;
2678406 128 83 64;
2678411 128 82 64;
2678411 128 72 64;
2678421 128 34 64;
2678421 128 41 64;
2678421 128 74 64;
2678421 128 80 64;
2678427 128 84 64;
2678432 128 31 64;
2678536 176 64 0;
2678536 176 64 127;
2678593 176 88 122;
2678593 176 88 125;
2678593 144 26 87;
2678619 176 88 125;
2678619 176 88 105;
2678619 144 27 40;
2678619 144 28 93;
2678619 144 29 83;
2678619 144 31 71;
2678619 144 32 71;
2678619 144 36 93;
2678619 144 83 114;
2678619 144 84 115;
2678619 144 86 105;
2678619 144 88 106;
2678619 144 89 106;
2678619 144 93 108;
2678619 176 88 105;
2678619 176 88 27;
2678619 144 91 93;
2678630 176 88 27;
2678630 176 88 20;
2678630 144 33 50;
2678635 176 88 20;
2678635 176 88 33;
2678635 144 90 45;
2678651 176 88 33;
2678651 176 88 30;
2678651 144 35 26;
2678677 128 27 64;
2678682 128 33 64;
2678723 128 35 64;
2678744 128 31 64;
2678765 128 88 64;
2678770 128 84 64;
2678776 128 90 64;
2678781 128 91 64;
2678781 176 64 127;
2678781 176 64 0;
2678791 128 28 64;
2678791 128 29 64;
2678802 128 32 64;
2678802 128 89 64;
2678802 128 86 64;
2678807 128 83 64;
2678812 128 36 64;
2678817 128 26 64;
2678828 128 93 64;
2679005 176 88 30;
2679005 176 88 26;
2679005 144 88 107;
2679005 144 94 91;
2679010 176 88 26;
2679010 176 88 66;
2679010 144 98 80;
2679041 176 88 66;
2679041 176 88 10;
2679041 144 23 68;
2679041 144 24 88;
2679041 144 25 68;
2679041 144 26 84;
2679041 144 27 37;
2679041 144 28 56;
2679041 144 29 76;
2679041 144 33 55;
2679041 144 35 44;
2679041 144 86 59;
2679041 144 87 32;
2679041 144 89 98;
2679041 144 91 80;
2679041 144 93 72;
2679041 144 95 81;
2679041 144 96 95;
2679046 176 88 10;
2679046 176 88 36;
2679046 144 31 67;
2679088 176 88 36;
2679088 176 88 80;
2679088 144 34 13;
2679109 176 64 0;
2679109 176 64 127;
2679197 128 93 64;
2679208 128 31 64;
2679218 128 89 64;
2679223 128 95 64;
2679229 128 28 64;
2679229 128 33 64;
2679234 128 26 64;
2679239 128 34 64;
2679239 128 88 64;
2679239 128 27 64;
2679255 128 35 64;
2679255 128 29 64;
2679260 128 91 64;
2679265 128 86 64;
2679270 128 25 64;
2679270 128 23 64;
2679276 176 64 127;
2679276 176 64 0;
2679286 128 24 64;
2679286 128 87 64;
2679286 128 96 64;
2679286 128 98 64;
2679291 128 94 64;
2679458 176 88 80;
2679458 176 88 120;
2679458 144 91 109;
2679473 176 88 120;
2679473 176 88 78;
2679473 144 29 97;
2679473 144 32 85;
2679473 144 33 82;
2679473 144 81 103;
2679473 144 83 102;
2679473 144 85 83;
2679473 144 88 94;
2679479 176 88 78;
2679479 176 88 10;
2679479 144 35 74;
2679479 144 86 91;
2679494 176 88 10;
2679494 176 88 79;
2679494 144 31 76;
2679494 144 36 81;
2679494 144 38 80;
2679494 144 40 59;
2679494 144 41 66;
2679494 144 84 77;
2679494 144 89 87;
2679494 144 90 24;
2679505 176 64 0;
2679505 176 64 127;
2679562 128 90 64;
2679567 176 88 79;
2679567 176 88 98;
2679567 128 84 64;
2679567 144 84 19;
2679630 128 31 64;
2679630 128 84 64;
2679630 128 38 64;
2679640 128 86 64;
2679645 128 89 64;
2679651 128 83 64;
2679656 128 33 64;
2679661 176 64 127;
2679661 176 64 0;
2679661 128 35 64;
2679671 128 36 64;
2679671 128 32 64;
2679671 128 40 64;
2679677 128 41 64;
2679682 128 29 64;
2679682 128 88 64;
2679687 128 81 64;
2679703 128 91 64;
2679708 128 85 64;
2679885 176 88 98;
2679885 176 88 3;
2679885 144 39 98;
2679890 176 88 3;
2679890 176 88 94;
2679890 144 74 105;
2679895 176 88 94;
2679895 176 88 10;
2679895 144 83 95;
2679921 176 88 10;
2679921 176 88 123;
2679921 144 41 74;
2679921 144 43 72;
2679921 144 46 60;
2679921 144 47 67;
2679921 144 48 56;
2679921 144 50 70;
2679921 144 72 95;
2679921 144 76 86;
2679921 144 77 80;
2679921 144 79 80;
2679921 144 80 69;
2679921 144 81 79;
2679932 176 88 123;
2679932 176 88 73;
2679932 144 45 55;
2679932 176 88 73;
2679932 176 88 101;
2679932 144 42 46;
2680072 128 77 64;
2680072 128 45 64;
2680093 128 47 64;

2680093 128 81 64;
2680098 128 48 64;
2680109 128 41 64;
2680125 128 74 64;
2680130 128 43 64;
2680135 128 42 64;
2680145 128 50 64;
2680161 128 80 64;
2680161 128 76 64;
2680161 128 79 64;
2680171 128 72 64;
2680171 128 46 64;
2680177 128 83 64;
2680187 128 39 64;
2680427 176 88 101;
2680427 176 88 106;
2680432 144 73 96;
2680437 176 88 106;
2680437 176 88 72;
2680437 144 50 89;
2680447 176 88 72;
2680447 176 88 89;
2680447 144 57 94;
2680447 144 66 81;
2680458 176 88 89;
2680458 176 88 2;
2680458 144 48 66;
2680458 144 52 89;
2680458 144 62 76;
2680458 144 64 81;
2680458 144 69 89;
2680458 144 71 79;
2680468 176 88 2;
2680468 176 88 32;
2680468 144 47 72;
2680468 144 53 76;
2680468 144 67 57;
2680468 144 70 55;
2680468 144 51 45;
2680479 176 88 32;
2680479 176 88 70;
2680479 144 65 38;
2680484 176 88 70;
2680484 176 88 117;
2680484 144 55 55;
2680510 128 53 64;
2680515 128 67 64;
2680531 128 65 64;
2680682 128 71 64;
2680718 128 55 64;
2680723 176 64 0;
2680723 176 64 127;
2680723 128 52 64;
2680729 128 64 64;
2680739 128 62 64;
2680744 128 50 64;
2680744 128 51 64;
2680755 128 69 64;
2680760 128 70 64;
2680776 128 47 64;
2680786 128 48 64;
2680796 128 66 64;
2680802 128 73 64;
2680802 128 57 64;
2681229 176 88 117;
2681229 176 88 46;
2681229 144 51 73;
2681234 176 88 46;
2681234 176 88 100;
2681234 144 59 95;
2681239 176 88 100;
2681239 176 88 23;
2681239 144 68 70;
2681250 176 64 127;
2681250 176 64 0;
2681250 176 88 23;
2681250 176 88 69;
2681250 144 61 66;
2681260 176 88 69;
2681260 176 88 25;
2681260 144 60 67;
2681260 144 63 59;
2681260 176 88 25;
2681260 176 88 84;
2681260 144 52 52;
2681265 176 88 84;
2681265 176 88 92;
2681265 144 65 50;
2681265 176 88 92;
2681265 176 88 106;
2681265 144 67 45;
2681281 176 88 106;
2681281 176 88 71;
2681281 144 57 51;
2681281 144 62 39;
2681286 176 88 71;
2681286 176 88 41;
2681286 144 53 45;
2681286 144 64 38;
2681291 176 88 41;
2681291 176 88 42;
2681291 144 50 48;
2681296 176 88 42;
2681296 176 88 71;
2681296 144 55 39;
2681369 128 53 64;
2681401 128 55 64;
2681593 176 64 0;
2681593 176 64 127;
2681619 128 52 64;
2681625 128 64 64;
2681661 128 62 64;
2681687 128 50 64;
2681697 128 57 64;
2681911 128 60 64;
2681921 128 63 64;
2681968 128 67 64;
2681973 128 65 64;
2681994 128 61 64;
2682046 128 59 64;
2682078 128 51 64;
2682156 128 68 64;
2682635 176 64 127;
2682635 176 64 0;
2753255 176 88 71;
2753255 176 88 78;
2753255 144 39 37;
2754364 128 39 64;
2754734 176 88 78;
2754734 176 88 58;
2754734 144 63 43;
2754880 176 88 58;
2754880 176 88 24;
2754880 144 65 66;
2754911 128 63 64;
2755244 128 65 64;
2755250 176 88 24;
2755250 176 88 8;
2755250 144 66 40;
2755411 176 88 8;
2755411 176 88 55;
2755411 144 68 54;
2755479 128 66 64;
2755770 176 88 55;
2755770 176 88 80;
2755770 144 66 38;
2755786 128 68 64;
2755890 128 66 64;
2756031 176 88 80;
2756031 176 88 7;
2756031 144 66 42;
2756171 176 88 7;
2756171 176 88 112;
2756171 144 65 56;
2756177 128 66 64;
2756244 128 65 64;
2756265 176 88 112;
2756265 176 88 4;
2756265 144 66 61;
2756359 128 66 64;
2756359 176 88 4;
2756359 176 88 96;
2756359 144 65 60;
2756406 176 88 96;
2756406 176 88 80;
2756406 144 66 88;
2756421 128 65 64;
2756494 176 88 80;
2756494 176 88 111;
2756494 144 65 74;
2756536 128 66 64;
2756635 128 65 64;
2756651 176 88 111;
2756651 176 88 106;
2756651 144 63 49;
2756901 176 88 106;
2756901 176 88 97;
2756901 144 65 65;
2756953 128 63 64;
2757119 176 88 97;
2757119 176 88 92;
2757119 144 66 44;
2757171 128 65 64;
2757317 176 88 92;
2757317 176 88 46;
2757317 144 68 74;
2757385 128 66 64;
2757557 128 68 64;
2757598 176 88 46;
2757598 176 88 61;
2757598 144 70 86;
2757890 128 70 64;
2757911 176 88 61;
2757911 176 88 37;
2757911 144 73 78;
2757958 128 73 64;
2757989 176 88 37;
2757989 176 88 83;
2757989 144 72 52;
2758302 176 88 83;
2758302 176 88 62;
2758302 144 73 51;
2758312 128 72 64;
2758588 128 73 64;
2758604 176 88 62;
2758604 176 88 86;
2758604 144 72 36;
2758682 128 72 64;
2758703 176 88 86;
2758703 176 88 111;
2758703 144 73 42;
2758770 176 88 111;
2758770 176 88 120;
2758770 128 73 64;
2758770 144 72 53;
2758848 176 88 120;
2758848 176 88 115;
2758848 144 73 64;
2758875 128 72 64;
2758979 128 73 64;
2759000 176 88 115;
2759000 176 88 25;
2759000 144 70 67;
2759250 128 70 64;
2759286 176 88 25;
2759286 176 88 89;
2759286 144 68 43;
2759598 128 68 64;
2760458 176 88 89;
2760458 176 88 55;
2760458 144 63 50;
2760661 176 88 55;
2760661 176 88 120;
2760661 128 63 64;
2760661 144 63 5;
2760729 128 63 64;
2760739 176 88 120;
2760739 176 88 10;
2760739 144 65 48;
2761005 128 65 64;
2761286 176 88 10;
2761286 176 88 48;
2761286 144 63 51;
2761437 128 63 64;
2761598 176 88 48;
2761598 176 88 117;
2761598 144 63 53;
2761651 128 63 64;
2761838 176 88 117;
2761838 176 88 114;
2761838 144 63 58;
2761963 128 63 64;
2762088 176 88 114;
2762088 176 88 60;
2762088 144 66 22;
2762197 128 66 64;
2762276 176 88 60;
2762276 176 88 59;
2762276 144 66 67;
2762401 128 66 64;
2762473 176 88 59;
2762473 176 88 12;
2762473 144 66 72;
2762552 128 66 64;
2762666 176 88 12;
2762666 176 88 84;
2762666 144 68 63;
2762755 128 68 64;
2762817 176 88 84;
2762817 176 88 37;
2762817 144 69 64;
2762906 128 69 64;
2762984 176 88 37;
2762984 176 88 107;
2762984 144 70 56;
2763041 128 70 64;
2763203 176 88 107;
2763203 176 88 12;
2763203 144 73 72;
2763255 128 73 64;
2764026 176 88 12;
2764026 176 88 59;
2764026 144 75 55;
2764307 128 75 64;
2764317 176 88 59;
2764317 176 88 43;
2764317 144 73 39;
2764385 128 73 64;
2764442 176 88 43;
2764442 176 88 39;
2764442 144 75 31;
2764494 176 88 39;
2764494 176 88 80;
2764494 144 73 39;
2764526 128 75 64;
2764562 128 73 64;
2764578 176 88 80;
2764578 176 88 100;
2764578 144 75 83;
2764880 176 88 100;
2764880 176 88 91;
2764880 144 74 72;
2764885 128 75 64;
2765156 176 88 91;
2765156 176 88 126;
2765156 144 73 67;
2765161 128 74 64;
2765427 176 88 126;
2765427 176 88 18;
2765427 144 70 58;
2765442 128 73 64;
2765619 128 70 64;
2765645 176 88 18;
2765645 176 88 84;
2765645 144 68 50;
2765723 128 68 64;
2765854 176 88 84;
2765854 176 88 8;
2765854 144 69 73;
2765932 128 69 64;
2766036 176 88 8;
2766036 176 88 86;
2766036 144 68 68;
2766098 128 68 64;
2766203 176 88 86;
2766203 176 88 22;
2766203 144 66 51;
2766296 128 66 64;
2766359 176 88 22;
2766359 176 88 97;
2766359 144 63 65;
2766692 128 63 64;
2766828 176 88 97;
2766828 176 88 119;
2766828 144 63 15;
2766947 128 63 64;
2766947 176 88 119;
2766947 176 88 102;
2766947 144 39 72;
2766979 176 88 102;
2766979 176 88 86;
2766979 144 63 61;

2768994 176 88 86;
2768994 176 88 111;
2768994 144 37 69;
2769020 128 39 64;
2769161 128 63 64;
2770197 128 37 64;
2770234 176 88 111;
2770234 176 88 12;
2770234 144 35 46;
2770317 128 35 64;
2793911 176 88 12;
2793911 176 88 123;
2793911 144 75 34;
2795359 128 75 64;
2796994 176 88 123;
2796994 176 88 37;
2796994 144 75 32;
2798755 128 75 64;
2798906 176 88 37;
2798906 176 88 84;
2798906 144 75 35;
2799473 128 75 64;
2799562 176 88 84;
2799562 176 88 34;
2799562 144 75 52;
2801932 176 88 34;
2801932 176 88 103;
2801932 144 87 21;
2802000 128 75 64;
2805812 128 87 64;
2806078 176 88 103;
2806078 176 88 121;
2806078 144 85 21;
2806750 176 88 121;
2806750 176 88 15;
2806750 144 84 45;
2806796 128 85 64;
2807692 128 84 64;
2807703 176 88 15;
2807703 176 88 11;
2807703 144 85 30;
2808958 128 85 64;
2808963 176 88 11;
2808963 176 88 77;
2808963 144 82 28;
2811395 128 82 64;
2811447 176 88 77;
2811447 176 88 113;
2811447 144 81 27;
2812828 176 88 113;
2812828 176 88 99;
2812828 144 80 35;
2812833 128 81 64;
2813270 176 88 99;
2813270 176 88 109;
2813270 144 81 48;
2813291 128 80 64;
2813770 176 88 109;
2813770 176 88 70;
2813770 144 78 41;
2813786 128 81 64;
2815932 128 78 64;
2815968 176 88 70;
2815968 176 88 0;
2815968 144 75 36;
2817083 176 88 0;
2817083 176 88 24;
2817083 128 75 64;
2817083 144 73 34;
2817328 176 88 24;
2817328 176 88 38;
2817328 144 75 41;
2817369 128 73 64;
2818802 176 88 38;
2818802 176 88 58;
2818802 144 63 28;
2818880 128 75 64;
2820192 128 63 64;
2820213 176 88 58;
2820213 176 88 19;
2820213 144 62 26;
2821848 128 62 64;
2821880 176 88 19;
2821880 176 88 102;
2821880 144 61 25;
2822390 176 88 102;
2822390 176 88 105;
2822390 144 63 40;
2822406 128 61 64;
2822755 128 63 64;
2822755 176 88 105;
2822755 176 88 7;
2822755 144 66 42;
2823286 176 88 7;
2823286 176 88 81;
2823286 144 68 29;
2823291 128 66 64;
2824015 128 68 64;
2824041 176 88 81;
2824041 176 88 14;
2824041 144 66 38;
2824614 176 88 14;
2824614 176 88 32;
2824614 144 75 38;
2825145 128 75 64;
2825182 128 66 64;
2825312 176 88 32;
2825312 176 88 93;
2825312 144 66 36;
2825312 144 75 42;
2825708 128 66 64;
2825729 128 75 64;
2825833 176 88 93;
2825833 176 88 3;
2825833 144 68 44;
2825875 176 88 3;
2825875 176 88 115;
2825875 144 78 49;
2826140 128 78 64;
2826151 128 68 64;
2826239 176 88 115;
2826239 176 88 20;
2826239 144 79 61;
2826250 176 88 20;
2826250 176 88 125;
2826250 144 69 47;
2826453 128 69 64;
2826453 128 79 64;
2826635 176 88 125;
2826635 176 88 91;
2826635 144 70 57;
2826640 176 88 91;
2826640 176 88 6;
2826640 144 82 60;
2826901 128 70 64;
2826921 128 82 64;
2827041 176 88 6;
2827041 176 88 122;
2827041 144 73 66;
2827052 176 88 122;
2827052 176 88 107;
2827052 144 85 70;
2827385 128 85 64;
2827390 128 73 64;
2827458 176 88 107;
2827458 176 88 0;
2827458 144 72 21;
2827463 176 88 0;
2827463 176 88 113;
2827463 144 84 38;
2827494 176 88 113;
2827494 176 88 126;
2827494 144 83 30;
2827708 128 83 64;
2827713 128 84 64;
2827880 128 72 64;
2827947 176 88 126;
2827947 176 88 34;
2827947 144 71 70;
2828484 176 88 34;
2828484 176 88 94;
2828484 128 71 64;
2828484 144 71 17;
2829869 128 71 64;
2830104 176 88 94;
2830104 176 88 68;
2830104 144 73 69;
2830109 176 88 68;
2830109 176 88 101;
2830109 144 85 60;
2830369 128 85 64;
2830380 128 73 64;
2830411 176 88 101;
2830411 176 88 104;
2830411 144 72 34;
2830421 176 88 104;
2830421 176 88 65;
2830421 144 84 31;
2830479 176 88 65;
2830479 176 88 75;
2830479 144 83 20;
2830557 128 83 64;
2830583 128 84 64;
2830729 176 88 75;
2830729 176 88 47;
2830729 144 71 75;
2830770 128 72 64;
2831244 176 88 47;
2831244 176 88 6;
2831244 144 72 79;
2831302 128 71 64;
2831625 176 88 6;
2831625 176 88 116;
2831625 144 71 39;
2831651 128 72 64;
2832046 176 88 116;
2832046 176 88 16;
2832046 144 84 44;
2832062 176 88 16;
2832062 176 88 71;
2832062 144 83 54;
2832291 128 83 64;
2832296 128 84 64;
2832390 128 71 64;
2832484 176 88 71;
2832484 176 88 30;
2832484 144 84 71;
2832489 176 88 30;
2832489 176 88 100;
2832489 144 71 46;
2832494 176 88 100;
2832494 176 88 15;
2832494 144 72 50;
2832817 128 84 64;
2832843 128 71 64;
2832843 128 72 64;
2832968 176 88 15;
2832968 176 88 38;
2832968 144 84 50;
2833005 176 88 38;
2833005 176 88 118;
2833005 144 71 43;
2833005 176 88 118;
2833005 176 88 92;
2833005 144 72 34;
2833270 128 84 64;
2833291 128 71 64;
2833296 128 72 64;
2833468 176 88 92;
2833468 176 88 20;
2833468 144 83 72;
2833479 176 88 20;
2833479 176 88 9;
2833479 144 71 44;
2833484 176 88 9;
2833484 176 88 126;
2833484 144 72 43;
2834380 128 72 64;
2834390 128 71 64;
2834442 128 83 64;
2834479 176 88 126;
2834479 176 88 9;
2834479 144 81 60;
2834510 176 88 9;
2834510 176 88 50;
2834510 144 71 31;
2834520 176 88 50;
2834520 176 88 108;
2834520 144 72 32;
2834645 128 72 64;
2834734 176 88 108;
2834734 176 88 45;
2834734 144 80 66;
2834776 128 71 64;
2834932 128 81 64;
2834989 176 88 45;
2834989 176 88 36;
2834989 144 78 48;
2835057 128 80 64;
2835255 176 88 36;
2835255 176 88 53;
2835255 144 80 67;
2835281 128 78 64;
2835703 128 80 64;
2835703 176 88 53;
2835703 176 88 6;
2835703 144 78 28;
2835979 128 78 64;
2835989 176 88 6;
2835989 176 88 72;
2835989 144 76 41;
2836151 176 88 72;
2836151 176 88 119;
2836151 144 75 51;
2836161 128 76 64;
2836255 128 75 64;
2836276 176 88 119;
2836276 176 88 15;
2836276 144 74 62;
2836359 128 74 64;
2836375 176 88 15;
2836375 176 88 115;
2836375 144 73 57;
2836479 176 88 115;
2836479 176 88 55;
2836479 144 72 62;
2836526 128 73 64;
2836572 176 88 55;
2836572 176 88 117;
2836572 144 69 50;
2836593 128 72 64;
2836953 176 88 117;
2836953 176 88 50;
2836953 144 67 65;
2837005 128 69 64;
2837250 176 88 50;
2837250 176 88 62;
2837250 144 66 51;
2837333 128 67 64;
2837630 176 88 62;
2837630 176 88 68;
2837630 144 65 51;
2837697 128 66 64;
2838057 128 65 64;
2838098 176 88 68;
2838098 176 88 12;
2838098 144 64 29;
2838713 176 88 12;
2838713 176 88 5;
2838713 144 65 39;
2838734 128 64 64;
2839072 128 65 64;
2839109 176 88 5;
2839109 176 88 66;
2839109 144 66 31;
2839338 128 66 64;
2839348 176 88 66;
2839348 176 88 22;
2839348 144 67 49;
2839536 176 88 22;
2839536 176 88 96;
2839536 144 69 66;
2839557 128 67 64;
2839713 176 88 96;
2839713 176 88 68;
2839713 144 70 74;
2839755 128 69 64;
2839869 128 70 64;
2839901 176 88 68;
2839901 176 88 17;
2839901 144 72 47;
2839911 176 88 17;
2839911 176 88 64;
2839911 144 71 41;
2839989 128 71 64;
2840140 176 88 64;
2840140 176 88 35;
2840140 144 74 66;
2840171 128 72 64;
2840411 176 88 35;
2840411 176 88 39;
2840411 144 75 47;

2840447 128 74 64;
2840682 176 88 39;
2840682 176 88 2;
2840682 144 78 38;
2840708 128 75 64;
2840994 176 88 2;
2840994 176 88 24;
2840994 144 79 66;
2841005 176 88 24;
2841005 176 88 35;
2841005 144 47 79;
2841026 128 78 64;
2841369 128 79 64;
2841369 176 88 35;
2841369 176 88 19;
2841369 144 78 64;
2841645 176 88 19;
2841645 176 88 38;
2841645 144 76 61;
2841671 128 78 64;
2841875 176 88 38;
2841875 176 88 107;
2841880 144 75 70;
2841937 128 76 64;
2842093 176 88 107;
2842093 176 88 106;
2842093 144 74 78;
2842104 128 75 64;
2842276 176 88 106;
2842276 176 88 74;
2842276 144 73 71;
2842307 128 74 64;
2842484 176 88 74;
2842484 176 88 98;
2842484 144 72 80;
2842546 128 73 64;
2842708 176 88 98;
2842708 176 88 60;
2842708 144 71 84;
2842713 128 72 64;
2842718 176 88 60;
2842718 176 88 76;
2842718 144 45 90;
2842760 128 47 64;
2842953 176 88 76;
2842953 176 88 29;
2842953 144 70 76;
2843015 128 71 64;
2843156 176 88 29;
2843156 176 88 99;
2843156 144 69 90;
2843171 128 70 64;
2843395 176 88 99;
2843395 176 88 92;
2843395 144 47 91;
2843401 176 88 92;
2843401 176 88 102;
2843401 144 68 78;
2843473 128 69 64;
2843479 128 45 64;
2843619 176 88 102;
2843619 176 88 93;
2843619 144 67 86;
2843635 128 68 64;
2843786 176 88 93;
2843786 176 88 34;
2843791 144 66 82;
2843791 176 88 34;
2843791 176 88 117;
2843791 144 45 91;
2843843 128 67 64;
2843843 128 47 64;
2843973 176 88 117;
2843973 176 88 70;
2843973 144 65 85;
2844005 176 88 70;
2844005 176 88 91;
2844005 144 47 79;
2844046 128 66 64;
2844072 128 45 64;
2844166 176 88 91;
2844166 176 88 84;
2844166 144 64 86;
2844177 176 88 84;
2844177 176 88 15;
2844177 144 45 80;
2844187 128 65 64;
2844229 128 47 64;
2844338 176 88 15;
2844338 176 88 12;
2844338 144 63 79;
2844354 176 88 12;
2844354 176 88 126;
2844354 144 47 70;
2844385 128 64 64;
2844406 128 45 64;
2844510 176 88 126;
2844510 176 88 116;
2844510 144 45 66;
2844515 128 63 64;
2844515 176 88 116;
2844515 176 88 123;
2844515 144 62 75;
2844552 128 47 64;
2844640 176 88 123;
2844640 176 88 9;
2844640 144 61 67;
2844671 176 88 9;
2844671 176 88 42;
2844671 128 62 64;
2844671 144 47 57;
2844718 128 45 64;
2844776 128 61 64;
2844781 176 88 42;
2844781 176 88 54;
2844781 144 60 80;
2844812 176 88 54;
2844812 176 88 85;
2844812 144 45 65;
2844848 128 47 64;
2844890 128 60 64;
2844895 176 88 85;
2844895 176 88 50;
2844895 144 61 42;
2844973 176 88 50;
2844973 176 88 2;
2844973 144 47 65;
2845020 128 45 64;
2845031 128 61 64;
2845057 176 88 2;
2845057 176 88 105;
2845057 144 59 37;
2845083 176 88 105;
2845083 176 88 40;
2845083 144 60 24;
2845109 176 88 40;
2845109 176 88 49;
2845109 144 61 62;
2845119 176 88 49;
2845119 176 88 127;
2845119 128 59 64;
2845119 144 45 68;
2845145 128 47 64;
2845156 128 60 64;
2845223 176 88 127;
2845223 176 88 9;
2845223 144 59 65;
2845244 128 61 64;
2845260 176 88 9;
2845260 176 88 2;
2845260 144 47 55;
2845302 128 45 64;
2845338 176 88 2;
2845338 176 88 67;
2845338 144 61 45;
2845364 176 88 67;
2845364 176 88 125;
2845364 144 45 87;
2845380 128 59 64;
2845390 128 47 64;
2845453 176 88 125;
2845453 176 88 80;
2845453 144 59 67;
2845473 128 61 64;
2845520 176 88 80;
2845520 176 88 15;
2845520 144 47 62;
2845536 128 59 64;
2845536 176 88 15;
2845536 176 88 75;
2845536 144 61 67;
2845557 128 45 64;
2845645 176 88 75;
2845645 176 88 105;
2845645 144 45 78;
2845656 176 88 105;
2845656 176 88 94;
2845656 144 60 77;
2845661 128 47 64;
2845661 128 61 64;
2845744 128 60 64;
2845750 176 88 94;
2845750 176 88 80;
2845750 144 61 54;
2845791 176 88 80;
2845791 176 88 36;
2845791 144 47 72;
2845828 128 45 64;
2845828 128 61 64;
2845869 144 62 77;
2845916 176 88 36;
2845916 176 88 121;
2845916 144 45 77;
2845937 128 47 64;
2845942 128 62 64;
2845968 176 88 121;
2845968 176 88 55;
2845968 144 63 58;
2846041 128 63 64;
2846052 176 88 55;
2846052 176 88 2;
2846052 144 47 57;
2846078 176 88 2;
2846078 176 88 107;
2846078 144 64 69;
2846088 128 45 64;
2846171 176 88 107;
2846171 176 88 88;
2846171 144 45 79;
2846187 128 64 64;
2846187 128 47 64;
2846197 176 88 88;
2846197 176 88 23;
2846197 144 65 76;
2846307 176 88 23;
2846307 176 88 2;
2846307 144 47 65;
2846328 128 45 64;
2846354 176 88 2;
2846354 176 88 119;
2846354 144 66 65;
2846380 128 65 64;
2846437 176 88 119;
2846437 176 88 10;
2846437 144 45 77;
2846447 128 47 64;
2846479 128 66 64;
2846489 176 88 10;
2846489 176 88 78;
2846489 144 67 73;
2846500 176 88 78;
2846500 176 88 31;
2846500 144 69 55;
2846567 128 69 64;
2846588 176 88 31;
2846588 176 88 35;
2846588 144 47 79;
2846593 128 67 64;
2846604 176 88 35;
2846604 176 88 46;
2846604 144 68 79;
2846614 128 45 64;
2846734 176 88 46;
2846734 176 88 19;
2846734 128 47 64;
2846734 128 68 64;
2846734 144 45 79;
2846734 144 69 88;
2846838 176 88 19;
2846838 176 88 50;
2846838 144 70 73;
2846854 128 69 64;
2846880 176 88 50;
2846880 176 88 70;
2846880 144 47 70;
2846890 128 45 64;
2846921 128 70 64;
2846942 176 88 70;
2846942 176 88 4;
2846942 144 71 84;
2847010 128 47 64;
2847015 176 88 4;
2847015 176 88 127;
2847015 144 45 80;
2847057 176 88 127;
2847057 176 88 110;
2847057 128 71 64;
2847057 144 72 71;
2847177 176 88 110;
2847177 176 88 64;
2847177 144 47 77;
2847177 176 88 64;
2847177 176 88 37;
2847177 144 73 78;
2847182 128 72 64;
2847182 128 45 64;
2847302 128 47 64;
2847302 128 73 64;
2847312 176 88 37;
2847312 176 88 15;
2847312 144 45 77;
2847312 144 74 67;
2847421 176 88 15;
2847421 176 88 118;
2847421 144 75 80;
2847453 128 74 64;
2847463 176 88 118;
2847463 176 88 109;
2847463 144 47 71;
2847479 128 45 64;
2847552 128 75 64;
2847567 176 88 109;
2847567 176 88 56;
2847567 144 74 65;
2847630 176 88 56;
2847630 176 88 38;
2847630 144 45 82;
2847640 128 47 64;
2847817 176 88 38;
2847817 176 88 45;
2847817 144 75 76;
2847822 176 88 45;
2847822 176 88 88;
2847822 144 47 69;
2847848 128 74 64;
2847864 128 45 64;
2848135 176 88 88;
2848135 176 88 36;
2848135 144 45 59;
2848151 128 75 64;
2848151 128 47 64;
2848182 176 88 36;
2848182 176 88 127;
2848182 144 81 45;
2848260 128 81 64;
2848432 176 88 127;
2848432 176 88 32;
2848432 144 82 46;
2848536 128 82 64;
2848729 176 88 32;
2848729 176 88 118;
2848729 144 82 63;
2848786 128 82 64;
2849026 176 88 118;
2849026 176 88 65;
2849026 144 82 60;
2849098 128 82 64;
2849348 176 88 65;
2849348 176 88 41;
2849348 144 82 53;
2849416 128 82 64;
2849416 176 88 41;
2849416 176 88 1;
2849416 144 43 52;
2849442 128 45 64;
2849770 176 88 1;
2849770 176 88 6;
2849770 144 82 60;
2849833 128 82 64;
2850239 176 88 6;
2850239 176 88 86;
2850239 144 82 46;

2850302 128 82 64;
2850723 176 88 86;
2850723 176 88 66;
2850723 144 82 40;
2850796 128 82 64;
2851197 176 88 66;
2851197 176 88 19;
2851197 144 82 41;
2851229 176 88 19;
2851229 176 88 104;
2851229 144 41 39;
2851244 128 43 64;
2851458 176 88 104;
2851458 176 88 85;
2851458 128 82 64;
2851458 144 82 17;
2851687 128 82 64;
2851838 176 88 85;
2851838 176 88 33;
2851838 144 94 44;
2851848 176 88 33;
2851848 176 88 45;
2851848 144 82 44;
2852000 128 82 64;
2852005 128 94 64;
2852234 176 88 45;
2852234 176 88 9;
2852234 144 94 56;
2852250 144 82 45;
2852296 128 94 64;
2852322 128 82 64;
2852515 176 88 9;
2852515 176 88 28;
2852515 144 80 61;
2852520 176 88 28;
2852520 176 88 66;
2852520 144 92 57;
2852572 128 80 64;
2852583 128 92 64;
2852760 176 88 66;
2852760 176 88 45;
2852760 144 78 68;
2852765 176 88 45;
2852765 176 88 6;
2852765 144 90 60;
2852812 128 78 64;
2852822 128 90 64;
2852979 176 88 6;
2852979 176 88 26;
2852979 144 89 89;
2852989 176 88 26;
2852989 176 88 122;
2852989 144 77 38;
2853052 128 89 64;
2853052 128 77 64;
2853255 176 88 122;
2853255 176 88 74;
2853255 144 87 83;
2853260 176 88 74;
2853260 176 88 121;
2853260 144 75 84;
2853322 128 75 64;
2853369 128 87 64;
2853567 176 88 121;
2853567 176 88 119;
2853567 144 74 89;
2853572 176 88 119;
2853572 176 88 30;
2853572 144 86 68;
2853630 128 74 64;
2853671 128 86 64;
2853890 176 88 30;
2853890 176 88 51;
2853890 144 73 100;
2853890 176 88 51;
2853890 176 88 26;
2853890 144 85 90;
2853963 128 73 64;
2854000 128 85 64;
2854187 176 88 26;
2854187 176 88 36;
2854187 144 72 86;
2854187 144 84 90;
2854244 128 72 64;
2854281 128 84 64;
2854500 176 88 36;
2854500 176 88 57;
2854500 144 43 61;
2854520 176 88 57;
2854520 176 88 95;
2854520 144 70 98;
2854526 176 88 95;
2854526 176 88 83;
2854526 144 82 89;
2854531 128 41 64;
2854625 128 70 64;
2854645 128 82 64;
2854848 176 88 83;
2854848 176 88 49;
2854848 144 45 78;
2854864 176 88 49;
2854864 176 88 57;
2854864 144 80 86;
2854869 176 88 57;
2854869 176 88 64;
2854869 144 68 89;
2854916 128 43 64;
2854953 128 68 64;
2854989 128 80 64;
2855187 176 88 64;
2855187 176 88 120;
2855187 144 47 77;
2855197 176 88 120;
2855197 176 88 44;
2855197 144 78 88;
2855203 176 88 44;
2855203 176 88 39;
2855203 144 66 86;
2855239 128 45 64;
2855281 128 66 64;
2855322 128 78 64;
2855541 176 88 39;
2855541 176 88 20;
2855541 144 75 65;
2855557 176 88 20;
2855557 176 88 30;
2855557 144 48 68;
2855588 128 47 64;
2855604 128 75 64;
2855885 176 88 30;
2855885 176 88 85;
2855885 144 64 93;
2855890 176 88 85;
2855890 176 88 43;
2855890 144 76 90;
2855994 128 64 64;
2856010 128 76 64;
2856260 176 88 43;
2856260 176 88 9;
2856260 144 64 92;
2856260 144 76 88;
2856291 176 88 9;
2856291 176 88 42;
2856291 144 74 57;
2856338 128 74 64;
2856359 128 64 64;
2856369 128 76 64;
2856635 176 88 42;
2856635 176 88 76;
2856635 144 63 94;
2856640 176 88 76;
2856640 176 88 44;
2856640 144 75 80;
2856645 176 88 44;
2856645 176 88 76;
2856645 144 50 61;
2856697 128 48 64;
2856744 128 63 64;
2856755 128 75 64;
2857062 176 88 76;
2857062 176 88 70;
2857062 144 61 93;
2857067 176 88 70;
2857067 176 88 80;
2857067 144 73 88;
2857182 128 61 64;
2857197 128 73 64;
2857500 176 88 80;
2857500 176 88 54;
2857500 144 62 88;
2857500 144 74 93;
2857531 176 88 54;
2857531 176 88 33;
2857531 144 51 54;
2857609 128 50 64;
2857609 128 74 64;
2857614 128 62 64;
2857901 176 88 33;
2857901 176 88 31;
2857901 144 60 91;
2857911 176 88 31;
2857911 176 88 25;
2857911 144 72 78;
2857921 176 88 25;
2857921 176 88 90;
2857921 144 52 65;
2857958 176 88 90;
2857958 176 88 15;
2857958 144 71 34;
2857963 128 60 64;
2857963 128 51 64;
2858005 128 72 64;
2858031 128 71 64;
2858281 176 88 15;
2858281 176 88 124;
2858281 144 61 89;
2858286 176 88 124;
2858286 176 88 71;
2858286 144 73 80;
2858291 176 88 71;
2858291 176 88 31;
2858291 144 53 70;
2858333 128 52 64;
2858348 128 73 64;
2858359 128 61 64;
2858458 128 53 64;
2858593 176 88 31;
2858593 176 88 18;
2858593 144 54 77;
2858640 176 88 18;
2858640 176 88 3;
2858640 144 72 71;
2858651 176 88 3;
2858651 176 88 58;
2858651 144 60 60;
2858651 144 71 80;
2858661 176 88 58;
2858661 176 88 31;
2858661 144 59 44;
2858708 128 59 64;
2858713 128 71 64;
2858739 128 60 64;
2858755 128 72 64;
2858958 128 54 64;
2858979 176 88 31;
2858979 176 88 79;
2858979 144 55 82;
2858984 176 88 79;
2858984 176 88 72;
2858984 144 59 71;
2858989 176 88 72;
2858989 176 88 93;
2858989 144 71 67;
2858994 176 88 93;
2858994 176 88 5;
2858994 144 69 79;
2859005 176 88 5;
2859005 176 88 39;
2859005 144 57 55;
2859057 128 57 64;
2859104 128 59 64;
2859109 128 71 64;
2859114 128 69 64;
2859192 128 55 64;
2859322 176 88 39;
2859322 176 88 25;
2859322 144 56 83;
2859328 176 88 25;
2859328 176 88 86;
2859328 176 88 86;
2859328 176 88 19;
2859328 144 60 92;
2859328 144 72 90;
2859463 128 60 64;
2859494 128 72 64;
2859593 128 56 64;
2859687 176 88 19;
2859687 176 88 122;
2859687 144 61 92;
2859687 176 88 122;
2859687 176 88 26;
2859687 144 73 89;
2859692 176 88 26;
2859692 176 88 71;
2859692 144 57 87;
2859692 144 58 26;
2859817 128 58 64;
2859833 128 57 64;
2859901 128 61 64;
2859932 128 73 64;
2860015 176 88 71;
2860015 176 88 80;
2860015 144 59 87;
2860026 176 88 80;
2860026 176 88 41;
2860026 144 62 89;
2860026 176 88 41;
2860026 176 88 57;
2860026 144 74 74;
2860062 176 88 57;
2860062 176 88 81;
2860062 144 72 46;
2860109 128 72 64;
2860151 128 62 64;
2860182 128 74 64;
2860250 128 59 64;
2860333 176 88 81;
2860333 176 88 93;
2860333 144 57 67;
2860333 176 88 93;
2860333 176 88 26;
2860333 144 75 90;
2860338 176 88 26;
2860338 176 88 0;
2860338 144 63 88;
2860338 176 88 0;
2860338 176 88 122;
2860338 144 59 75;
2860531 128 59 64;
2860541 128 63 64;
2860557 128 75 64;
2860572 128 57 64;
2860630 176 88 122;
2860630 176 88 26;
2860630 144 56 79;
2860671 176 88 26;
2860671 176 88 72;
2860671 144 76 83;
2860677 176 88 72;
2860677 176 88 52;
2860677 144 64 88;
2860838 128 64 64;
2860869 128 76 64;
2860927 128 56 64;
2860932 176 88 52;
2860932 176 88 36;
2860932 144 55 77;
2860953 176 88 36;
2860953 176 88 117;
2860953 144 65 100;
2860953 176 88 117;
2860953 176 88 50;
2860953 144 77 99;
2861098 128 65 64;
2861098 128 77 64;
2861203 176 88 50;
2861203 176 88 113;
2861203 144 54 86;
2861239 176 88 113;
2861239 176 88 94;
2861239 128 55 64;
2861239 144 66 86;
2861239 144 78 90;
2861291 176 88 94;
2861291 176 88 5;
2861291 144 68 25;
2861364 128 68 64;
2861406 128 78 64;
2861411 128 66 64;
2861468 128 54 64;
2861479 176 88 5;
2861479 176 88 50;

2861479 144 53 76;
2861489 176 88 50;
2861489 176 88 1;
2861489 144 67 88;
2861494 176 88 1;
2861494 176 88 55;
2861494 144 79 74;
2861546 176 88 55;
2861546 176 88 75;
2861546 144 77 24;
2861619 128 77 64;
2861640 128 67 64;
2861656 128 79 64;
2861739 176 88 75;
2861739 176 88 84;
2861739 144 52 85;
2861760 128 53 64;
2861776 176 88 84;
2861776 176 88 43;
2861776 144 68 92;
2861781 176 88 43;
2861781 176 88 4;
2861781 144 80 84;
2861937 128 80 64;
2861942 128 68 64;
2862046 128 52 64;
2862052 176 88 4;
2862052 176 88 19;
2862052 144 51 79;
2862072 176 88 19;
2862072 176 88 26;
2862072 144 69 91;
2862182 128 69 64;
2862427 176 88 26;
2862427 176 88 47;
2862427 144 70 96;
2862432 176 88 47;
2862432 176 88 53;
2862432 144 49 67;
2862437 176 88 53;
2862437 176 88 33;
2862437 144 82 81;
2862473 128 51 64;
2862494 176 88 33;
2862494 176 88 94;
2862494 144 83 30;
2862583 128 83 64;
2862760 128 82 64;
2862765 128 70 64;
2863541 176 88 94;
2863541 176 88 21;
2863541 144 75 36;
2863541 144 87 35;
2863546 128 49 64;
2863562 176 88 21;
2863562 176 88 56;
2863562 144 51 32;
2865000 128 87 64;
2865046 128 75 64;
2865239 176 88 56;
2865239 176 88 23;
2865239 144 75 30;
2865250 176 88 23;
2865250 176 88 78;
2865250 144 87 31;
2865411 128 75 64;
2865427 128 87 64;
2865869 176 88 78;
2865869 176 88 47;
2865869 144 87 18;
2865875 176 88 47;
2865875 176 88 94;
2865875 144 75 20;
2866255 128 75 64;
2866302 128 87 64;
2866625 176 88 94;
2866625 176 88 57;
2866625 144 90 33;
2866760 128 90 64;
2867281 176 88 57;
2867281 176 88 121;
2867281 144 90 21;
2867296 176 88 121;
2867296 176 88 118;
2867296 144 78 20;
2868109 128 90 64;
2868255 128 78 64;
2868822 176 88 118;
2868822 176 88 80;
2868822 144 75 22;
2868843 176 88 80;
2868843 176 88 94;
2868843 144 87 13;
2870109 128 51 64;
2870223 176 88 94;
2870223 176 88 101;
2870223 144 51 49;
2870567 128 51 64;
2870619 176 88 101;
2870619 176 88 95;
2870619 144 49 64;
2870958 128 49 64;
2871000 176 88 95;
2871000 176 88 111;
2871000 144 48 85;
2871296 128 48 64;
2871338 176 88 111;
2871338 176 88 51;
2871338 144 47 94;
2871614 128 47 64;
2871645 176 88 51;
2871645 176 88 4;
2871645 144 46 90;
2871890 128 46 64;
2871906 176 88 4;
2871906 176 88 73;
2871906 144 44 78;
2872166 128 44 64;
2872218 176 88 73;
2872218 176 88 113;
2872218 144 42 76;
2872500 128 42 64;
2872598 176 88 113;
2872598 176 88 60;
2872598 144 39 79;
2872656 128 75 64;
2872671 128 87 64;
2873276 176 88 60;
2873276 176 88 4;
2873276 144 82 55;
2873286 176 88 4;
2873286 176 88 105;
2873286 144 70 47;
2873713 128 82 64;
2873723 128 70 64;
2873817 176 88 105;
2873817 176 88 103;
2873817 144 70 51;
2873817 144 82 47;
2874151 128 82 64;
2874171 128 70 64;
2874312 176 88 103;
2874312 176 88 12;
2874312 144 80 70;
2874312 176 88 12;
2874312 176 88 21;
2874312 144 68 68;
2874598 128 80 64;
2874609 128 68 64;
2874666 176 88 21;
2874666 176 88 104;
2874666 144 68 78;
2874666 144 80 65;
2874906 128 68 64;
2874916 128 80 64;
2875015 176 88 104;
2875015 176 88 89;
2875015 144 78 93;
2875026 176 88 89;
2875026 176 88 126;
2875026 144 66 79;
2875062 176 88 126;
2875062 176 88 22;
2875062 144 68 30;
2875145 128 68 64;
2875203 128 66 64;
2875223 128 78 64;
2875338 176 88 22;
2875338 176 88 15;
2875338 144 66 81;
2875343 176 88 15;
2875343 176 88 87;
2875343 144 78 80;
2875604 128 66 64;
2875614 128 78 64;
2875744 176 88 87;
2875744 176 88 59;
2875744 144 77 64;
2875760 176 88 59;
2875760 176 88 44;
2875760 144 65 45;
2876015 128 65 64;
2876015 128 77 64;
2876244 176 88 44;
2876244 176 88 50;
2876244 144 77 45;
2876270 176 88 50;
2876270 176 88 97;
2876270 144 65 28;
2877786 128 39 64;
2877890 176 88 97;
2877890 176 88 111;
2877890 144 39 38;
2878156 128 39 64;
2878223 176 88 111;
2878223 176 88 21;
2878223 144 39 71;
2878489 128 39 64;
2878536 176 88 21;
2878536 176 88 45;
2878536 144 41 67;
2878770 128 41 64;
2878817 176 88 45;
2878817 176 88 36;
2878817 144 42 76;
2879135 176 88 36;
2879135 176 88 85;
2879135 144 44 92;
2879187 128 42 64;
2879526 176 88 85;
2879526 176 88 27;
2879526 128 44 64;
2879526 144 45 63;
2879916 176 88 27;
2879916 176 88 11;
2879916 144 44 50;
2879979 128 45 64;
2880479 176 88 11;
2880479 176 88 119;
2880479 144 39 34;
2880484 128 44 64;
2880562 128 77 64;
2880666 128 65 64;
2880994 176 88 119;
2880994 176 88 24;
2880994 144 75 66;
2881000 176 88 24;
2881000 176 88 98;
2881000 144 63 52;
2881250 128 75 64;
2881281 128 63 64;
2881380 176 88 98;
2881380 176 88 60;
2881380 144 63 69;
2881390 176 88 60;
2881390 176 88 81;
2881390 144 75 74;
2881583 128 63 64;
2881598 128 75 64;
2881703 176 88 81;
2881703 176 88 63;
2881703 144 73 68;
2881708 176 88 63;
2881708 176 88 18;
2881708 144 61 78;
2881911 128 61 64;
2881932 128 73 64;
2882026 176 88 18;
2882026 176 88 76;
2882026 144 63 85;
2882031 176 88 76;
2882031 176 88 118;
2882031 144 75 80;
2882177 128 63 64;
2882203 128 75 64;
2882291 176 88 118;
2882291 176 88 98;
2882291 144 61 91;
2882296 176 88 98;
2882296 176 88 125;
2882296 144 73 83;
2882411 128 61 64;
2882458 128 73 64;
2882546 176 88 125;
2882546 176 88 58;
2882546 144 75 88;
2882552 176 88 58;
2882552 176 88 4;
2882552 144 63 82;
2882687 128 63 64;
2882692 128 75 64;
2882786 176 88 4;
2882786 176 88 60;
2882786 144 61 91;
2882786 144 73 87;
2882911 128 61 64;
2882947 128 73 64;
2883104 176 88 60;
2883104 176 88 126;
2883104 144 63 48;
2883104 144 75 64;
2883385 128 39 64;
2883458 176 88 126;
2883458 176 88 90;
2883458 144 39 57;
2883708 128 63 64;
2883776 176 88 90;
2883776 176 88 12;
2883776 144 63 79;
2883953 128 63 64;
2884062 176 88 12;
2884062 176 88 96;
2884062 144 65 68;
2884093 128 75 64;
2884307 128 39 64;
2884307 128 65 64;
2884354 176 88 96;
2884354 176 88 71;
2884354 144 66 84;
2884359 176 88 71;
2884359 176 88 126;
2884359 144 39 68;
2884671 176 88 126;
2884671 176 88 46;
2884671 144 68 74;
2884682 128 39 64;
2884802 128 66 64;
2884979 176 88 46;
2884979 176 88 103;
2884979 144 69 83;
2884984 176 88 103;
2884984 176 88 90;
2884984 144 39 57;
2884994 128 68 64;
2885317 176 88 90;
2885317 176 88 76;
2885317 144 70 47;
2885322 128 69 64;
2885625 128 39 64;
2885682 128 70 64;
2885692 176 88 76;
2885692 176 88 83;
2885692 144 72 40;
2885703 176 88 83;
2885703 176 88 90;
2885703 144 39 57;
2886088 176 88 90;
2886088 176 88 64;
2886088 144 73 38;
2886119 128 72 64;
2886322 128 39 64;
2886489 176 88 64;
2886489 176 88 98;
2886489 144 74 40;
2886505 176 88 98;
2886505 176 88 27;
2886505 128 73 64;
2886505 144 39 34;
2886973 128 74 64;
2887000 176 88 27;
2887000 176 88 47;
2887000 144 75 28;

2887260 128 39 64;
2887614 176 88 47;
2887614 176 88 15;
2887614 144 39 32;
2887661 176 88 15;
2887661 176 88 81;
2887661 144 76 35;
2887671 128 75 64;
2888968 128 76 64;
2888989 176 88 81;
2888989 176 88 12;
2888989 144 88 37;
2890614 128 88 64;
2891432 176 88 12;
2891432 176 88 51;
2891432 144 87 21;
2891468 176 88 51;
2891468 176 88 107;
2891468 144 99 16;
2895500 128 99 64;
2895505 128 87 64;
2895776 128 39 64;
3062135 176 88 107;
3062135 176 88 40;
3062135 144 27 26;
3062177 176 88 40;
3062177 176 88 24;
3062177 144 39 21;
3063192 128 39 64;
3063192 128 27 64;

5838 176 88 0;
5838 176 88 59;
5838 144 28 46;
6145 128 28 64;
6854 176 88 59;
6854 176 88 103;
6854 144 35 48;
7223 128 35 64;
72328 176 88 103;
72328 176 88 98;
72328 144 63 25;
72343 176 88 98;
72343 176 88 100;
72343 144 60 28;
72364 176 88 100;
72364 176 88 61;
72364 144 67 31;
74947 128 63 64;
76588 176 88 61;
76588 176 88 86;
76588 128 60 64;
76588 144 65 38;
76609 128 67 64;
78177 128 65 64;
79500 176 88 86;
79500 176 88 40;
79500 144 67 27;
79520 176 88 40;
79520 176 88 68;
79520 144 60 22;
79526 176 88 68;
79526 176 88 72;
79526 144 63 23;
80484 128 63 64;
80984 128 60 64;
81015 176 88 72;
81015 176 88 48;
81015 144 65 31;
81098 128 67 64;
81796 128 65 64;
82000 176 88 48;
82000 176 88 13;
82000 144 67 27;
82026 176 88 13;
82026 176 88 34;
82026 144 60 27;
82098 176 88 34;
82098 176 88 119;
82098 144 63 12;
82317 128 63 64;
82739 128 60 64;
82796 128 67 64;
82828 176 88 119;
82828 176 88 11;
82828 144 68 24;
84562 128 68 64;
84708 176 88 11;

84708 176 88 35;
84708 144 63 27;
84713 176 88 35;
84713 176 88 48;
84713 144 67 26;
84713 176 88 48;
84713 176 88 100;
84713 144 60 16;
86718 128 60 64;
86807 128 63 64;
88197 128 67 64;
88234 176 88 100;
88234 176 88 121;
88234 144 70 26;
88885 128 70 64;
88927 176 88 121;
88927 176 88 17;
88927 144 68 21;
90208 128 68 64;
90390 176 88 17;
90390 176 88 83;
90390 144 63 13;
90484 176 88 83;
90484 176 88 49;
90484 144 67 21;
90500 176 88 49;
90500 176 88 97;
90500 144 72 18;
91921 128 67 64;
91932 128 63 64;
92322 176 88 97;
92322 176 88 16;
92322 144 65 27;
92375 128 72 64;
93682 128 65 64;
93703 176 88 16;
93703 176 88 7;
93703 144 68 14;
94114 128 68 64;
94130 176 88 7;
94130 176 88 122;
94130 144 67 20;
95510 128 67 64;
95567 176 88 122;
95567 176 88 80;
95567 144 65 26;
95625 176 88 80;
95625 176 88 1;
95625 144 72 22;
96718 128 65 64;
96739 128 72 64;
96817 144 70 9;
99223 128 70 64;
99343 176 88 1;
99343 176 88 100;
99343 144 68 15;
100052 144 67 22;

100057 128 68 64;
100343 128 67 64;
100390 176 88 100;
100390 176 88 108;
100390 144 65 22;
100713 128 65 64;
100781 176 88 108;
100781 176 88 13;
100781 144 67 24;
101619 128 67 64;
101651 176 88 13;
101651 176 88 67;
101651 144 68 26;
102578 176 88 67;
102578 176 88 0;
102578 144 65 23;
102593 176 88 0;
102593 176 88 41;
102598 144 72 30;
102703 128 68 64;
103312 128 65 64;
103380 176 88 41;
103380 176 88 75;
103380 144 68 22;
103395 128 72 64;
105692 128 68 64;
105880 176 88 75;
105880 176 88 114;
105880 144 63 14;
105932 176 88 114;
105932 176 88 105;
105932 144 67 22;
105947 176 88 105;
105947 176 88 96;
105947 144 72 22;
106687 128 67 64;
106692 128 72 64;
106812 176 88 96;
106812 176 88 78;
106812 128 63 64;
106812 144 70 14;
107755 128 70 64;
110395 176 88 78;
110395 176 88 19;
110395 144 65 11;
113536 176 88 19;
113536 176 88 123;
113536 144 63 9;
113598 128 65 64;
117036 128 63 64;
117427 176 88 123;
117427 176 88 19;
117427 144 67 12;
117453 176 88 19;
117453 176 88 76;
117453 144 60 15;
118489 128 67 64;

118520 176 88 76;
118520 176 88 122;
118520 144 65 25;
118546 128 60 64;
119489 176 88 122;
119489 176 88 127;
119489 144 63 23;
119515 128 65 64;
120234 176 88 127;
120234 176 88 24;
120234 144 67 32;
120250 176 88 24;
120250 176 88 73;
120250 128 63 64;
120250 144 60 19;
121083 176 88 73;
121083 176 88 62;
121083 144 63 20;
121109 128 60 64;
121119 128 67 64;
121723 176 88 62;
121723 176 88 40;
121723 144 62 25;
122119 128 62 64;
122328 176 88 40;
122328 176 88 61;
122328 144 67 31;
122338 176 88 61;
122338 176 88 107;
122338 144 60 21;
122369 128 63 64;
122958 176 88 107;
122958 176 88 91;
122958 128 67 64;
122958 144 65 33;
122989 128 60 64;
123473 176 88 91;
123473 176 88 29;
123473 144 63 30;
123500 128 65 64;
123963 176 88 29;
123963 176 88 72;
123963 128 63 64;
123963 144 67 40;
123989 176 88 72;
123989 176 88 104;
123989 144 60 24;
124458 128 60 64;
124463 176 88 104;
124463 176 88 6;
124463 144 63 35;
124484 128 67 64;
124921 176 88 6;
124921 176 88 118;
124921 128 63 64;
124921 144 62 30;
125390 128 62 64;

125447 176 88 118;
125447 176 88 102;
125447 144 67 33;
125489 176 88 102;
125489 176 88 62;
125489 144 60 21;
126927 128 67 64;
126947 176 88 62;
126947 176 88 38;
126947 144 65 38;
126994 128 60 64;
127854 128 65 64;
127947 176 88 38;
127947 176 88 21;
127953 144 67 31;
127958 176 88 21;
127958 176 88 107;
127958 144 60 21;
129916 128 60 64;
129947 176 88 107;
129947 176 88 99;
129947 144 63 17;
129973 128 67 64;
130390 128 63 64;
130432 176 88 99;
130432 176 88 91;
130432 144 67 30;
130494 176 88 91;
130494 176 88 48;
130494 144 60 21;
132062 128 60 64;
132125 176 88 48;
132125 176 88 1;
132125 144 62 18;
132255 128 67 64;
135390 128 62 64;
135531 176 88 1;
135531 176 88 3;
135531 144 67 17;
135557 176 88 3;
135557 176 88 55;
135557 144 60 10;
136385 128 67 64;
136453 176 88 55;
136453 176 88 46;
136453 144 65 22;
136494 128 60 64;
137067 128 65 64;
137125 176 88 46;
137125 176 88 43;
137125 144 67 28;
137177 176 88 43;
137177 176 88 76;
137177 144 60 15;
141718 128 60 64;
141802 128 67 64;
439515 176 88 76;
439515 176 88 84;
439515 144 69 22;
440864 176 88 84;
440864 176 88 86;
440864 144 81 15;
441864 128 69 64;
442375 176 88 86;
442375 176 88 120;
442375 144 69 15;
443046 128 81 64;
443869 176 88 120;
443869 176 88 126;
443869 144 81 4;
444421 128 69 64;
445130 176 88 126;
445130 176 88 14;
445130 144 68 15;
445343 128 81 64;
446322 176 88 14;
446322 176 88 92;
446322 144 80 16;
446593 128 68 64;
447526 176 88 92;
447526 176 88 77;
447526 144 69 24;
447531 128 80 64;
448645 176 88 77;
448645 176 88 74;
448645 144 81 20;
448833 128 69 64;
449838 176 88 74;
449838 176 88 89;
449838 144 70 18;
449994 128 81 64;
450989 176 88 89;
450989 176 88 102;
450989 144 82 22;
451229 128 70 64;
452192 128 82 64;
452265 176 88 102;
452265 176 88 0;
452265 144 69 1;
453489 176 88 0;
453489 176 88 53;
453489 144 81 9;
454901 128 69 64;
455697 176 88 53;
455697 176 88 26;
455697 144 69 3;
455817 128 81 64;
456979 176 88 26;
456979 176 88 0;
456979 144 81 1;
457708 128 69 64;
458286 176 88 0;
458286 176 88 52;
458286 144 80 23;
458406 128 81 64;
459427 176 88 52;
459427 176 88 56;
459427 144 68 13;
459619 128 80 64;
460781 176 88 56;
460781 176 88 17;
460781 144 81 7;
460833 128 68 64;
462000 176 88 17;
462000 176 88 73;
462000 144 69 2;
462557 128 81 64;
463062 128 69 64;
494901 176 88 73;
494901 176 88 90;
494901 144 69 29;
496333 176 88 90;
496333 176 88 107;
496333 144 81 23;
496536 128 69 64;
498328 128 81 64;
498333 176 88 107;
498333 176 88 67;
498333 144 69 9;
499557 176 88 67;
499557 176 88 84;
499557 144 81 24;
499723 128 69 64;
500781 144 68 17;
500817 128 81 64;
502151 176 88 84;
502151 176 88 62;
502151 144 80 30;
502244 128 68 64;
503380 176 88 62;
503380 176 88 48;
503380 144 69 31;
503432 128 80 64;
504552 176 88 48;
504552 176 88 74;
504552 144 81 35;
504645 128 69 64;
505828 176 88 74;
505828 176 88 17;
505828 144 70 23;
505895 128 81 64;
507041 176 88 17;
507041 176 88 115;
507041 144 82 25;
507250 128 70 64;
508296 128 82 64;
508333 176 88 115;
508333 176 88 73;
508333 144 69 11;
509593 176 88 73;
509593 176 88 79;
509593 144 81 31;
509927 128 69 64;
510406 128 81 64;
510822 176 88 79;
510822 176 88 74;
510822 144 81 35;
511989 176 88 74;
511989 176 88 15;
511989 144 69 23;
512281 128 81 64;
513286 176 88 15;
513286 176 88 75;
513286 144 82 23;
513442 128 69 64;
514572 176 88 75;
514572 176 88 23;
514572 144 70 24;
514744 128 82 64;
515895 144 81 24;
516166 128 70 64;
517114 128 81 64;
517140 176 88 23;
517140 176 88 12;
517140 144 68 17;
518348 176 88 12;
518348 176 88 65;
518348 144 80 13;
518494 128 68 64;
519677 128 80 64;
519713 176 88 65;
519713 176 88 56;
519713 144 69 19;
520973 176 88 56;
520973 176 88 85;
520973 144 81 25;
521281 128 69 64;
522140 128 81 64;
522182 176 88 85;
522182 176 88 11;
522182 144 71 25;
524348 128 71 64;
524692 176 88 11;
524692 176 88 0;
524692 144 71 15;
525927 176 88 0;
525927 176 88 67;
525927 144 83 18;
526177 128 71 64;
527260 176 88 67;
527260 176 88 110;
527260 144 70 13;
527359 128 83 64;
528598 176 88 110;
528598 176 88 76;
528598 144 82 21;
528848 128 70 64;
529812 176 88 76;
529812 176 88 14;
529812 144 68 18;
529838 128 82 64;
531093 176 88 14;
531093 176 88 90;
531093 144 80 23;
531411 128 68 64;
532380 128 80 64;
532453 176 88 90;
532453 176 88 58;
532453 144 67 16;
533625 176 88 58;
533625 176 88 87;
533625 144 79 26;
533869 128 67 64;
535031 176 88 87;
535031 176 88 10;
535031 144 68 16;
535057 128 79 64;
536401 176 88 10;
536401 176 88 106;
536401 144 80 10;
536671 128 68 64;
537421 128 80 64;
537567 176 88 106;
537567 176 88 5;
537567 144 71 26;
538854 176 88 5;
538854 176 88 43;
538854 144 83 29;
539177 128 71 64;
540114 128 83 64;
540166 176 88 43;
540166 176 88 47;
540166 144 74 30;
541463 176 88 47;
541463 176 88 77;
541463 144 86 26;
541765 128 74 64;
542822 176 88 77;
542822 176 88 78;
542822 144 75 16;
543057 128 86 64;
544072 176 88 78;
544072 176 88 27;
544072 144 87 19;
544697 128 75 64;
545364 176 88 27;
545364 176 88 68;
545364 144 76 24;
545406 128 87 64;
546197 128 76 64;
546885 176 88 68;
546885 176 88 0;
546885 144 88 1;
547494 128 88 64;
548026 176 88 0;
548026 176 88 125;
548026 144 76 19;
549401 176 88 125;
549401 176 88 17;
549401 144 88 15;
550026 128 76 64;
550281 128 88 64;
550843 176 88 17;
550843 176 88 49;
550843 144 88 31;
552317 176 88 49;
552317 176 88 13;
552317 144 76 23;
552848 128 88 64;
553562 176 88 13;
553562 176 88 117;
553562 144 86 22;
553770 128 76 64;
555166 176 88 117;
555166 176 88 0;
555166 128 86 64;
555166 144 74 1;
556843 176 88 0;
556843 176 88 105;
556843 144 85 15;
556864 128 74 64;
558375 176 88 105;
558375 176 88 75;
558375 144 73 7;
559885 128 73 64;
560083 176 88 75;
560083 176 88 122;
560083 144 74 20;
560822 128 85 64;
561546 176 88 122;
561546 176 88 21;
561546 144 86 23;
562526 128 74 64;
564536 176 88 21;
564536 176 88 36;
564536 144 74 7;
564541 128 86 64;
566088 176 88 36;
566088 176 88 38;
566088 144 86 19;
566515 128 74 64;
567494 128 86 64;
567598 176 88 38;
567598 176 88 39;
567598 144 76 21;
569182 176 88 39;
569182 176 88 27;
569182 144 88 13;
569531 128 76 64;
570572 176 88 27;
570572 176 88 63;
570572 144 75 22;

570625 128 88 64;
572119 176 88 63;
572119 176 88 122;
572119 144 87 28;
572869 128 75 64;
573463 128 87 64;
573473 176 88 122;
573473 176 88 79;
573473 144 74 27;
574968 176 88 79;
574968 176 88 93;
574968 144 86 25;
575104 128 74 64;
576296 128 86 64;
576364 176 88 93;
576364 176 88 34;
576364 144 84 30;
577776 176 88 34;
577776 176 88 28;
577776 144 72 21;
577880 128 84 64;
579119 176 88 28;
579119 176 88 13;
579119 144 82 30;
579312 128 72 64;
580572 176 88 13;
580572 176 88 116;
580572 144 70 21;
580718 128 82 64;
581890 176 88 116;
581890 176 88 49;
581890 144 81 25;
582046 128 70 64;
583343 176 88 49;
583343 176 88 62;
583343 144 69 12;
584364 128 69 64;
586296 176 88 62;
586296 176 88 33;
586296 144 69 19;
586520 128 81 64;
587265 176 88 33;
587265 176 88 48;
587265 144 81 20;
589604 128 69 64;
590697 128 81 64;
646583 176 88 48;
646583 176 88 53;
646583 144 71 10;
646989 128 71 64;
647442 176 88 53;
647442 176 88 112;
647442 144 71 10;
648468 128 71 64;
655343 176 88 112;
655343 176 88 2;
655343 144 71 3;
655375 176 88 2;
655375 176 88 0;
655375 144 74 1;
656744 176 88 0;
656744 176 88 47;
656744 144 73 23;
656781 128 74 64;
656942 128 71 64;
657734 128 73 64;
658901 176 88 47;
658901 176 88 69;
658901 144 72 7;
660572 128 72 64;
665281 176 88 69;
665281 176 88 81;
665281 144 71 20;
665296 176 88 81;
665296 176 88 1;
665296 144 74 17;
666921 176 88 1;
666921 176 88 13;
666927 144 73 6;
666942 128 74 64;
667713 128 73 64;
667828 176 88 13;
667828 176 88 1;
667828 144 72 20;
667875 128 71 64;
668526 128 72 64;
724593 176 88 1;
724593 176 88 6;
724593 144 76 18;
724906 128 76 64;
725312 176 88 6;
725312 176 88 66;
725312 144 74 19;
725510 128 74 64;
728020 176 88 66;
728020 176 88 81;
728020 144 73 19;
728067 176 88 81;
728067 176 88 115;
728067 144 71 13;
729140 128 73 64;
729145 176 88 115;
729145 176 88 73;
729145 144 74 20;
729354 128 71 64;
729541 128 74 64;
976968 176 88 73;
976968 176 88 68;
976968 144 74 28;
977442 176 88 68;
977442 176 88 20;
977442 144 73 23;
977500 128 74 64;
977812 128 73 64;
977854 176 88 20;
977854 176 88 36;
977854 144 72 35;
978177 128 72 64;
978213 176 88 36;
978213 176 88 66;
978213 144 71 28;
978614 128 71 64;
978635 176 88 66;
978635 176 88 6;
978635 144 70 29;
985000 128 70 64;
987380 176 88 6;
987380 176 88 28;
987380 144 70 13;
988546 176 88 28;
988546 176 88 75;
988546 128 70 64;
988546 144 71 22;
991166 128 71 64;
991187 176 88 75;
991187 176 88 79;
991187 144 74 19;
999432 128 74 64;
999510 176 88 79;
999510 176 88 12;
999510 144 72 20;
1000578 128 72 64;
1000625 176 88 12;
1000625 176 88 82;
1000625 144 74 16;
1001302 128 74 64;
1001338 176 88 82;
1001338 176 88 84;
1001338 144 73 16;
1002036 128 73 64;
1002187 176 88 84;
1002187 176 88 74;
1002187 144 74 10;
1010494 176 88 74;
1010494 176 88 10;
1010494 144 69 13;
1010692 128 74 64;
1011442 128 69 64;
1011505 176 88 10;
1011505 176 88 80;
1011505 144 70 9;
1012432 128 70 64;
1012484 176 88 80;
1012484 176 88 65;
1012484 144 69 18;
1014385 128 69 64;
1014390 176 88 65;
1014390 176 88 90;
1014390 144 68 15;
1015380 176 88 90;
1015380 176 88 13;
1015380 144 69 14;
1015479 128 68 64;
1016989 128 69 64;
1017229 176 88 13;
1017229 176 88 0;
1017229 144 70 1;
1017770 176 88 0;
1017770 176 88 73;
1017770 144 69 20;
1017786 128 70 64;
1018104 128 69 64;
1018291 176 88 73;
1018291 176 88 63;
1018291 144 68 6;
1018760 128 68 64;
1018802 176 88 63;
1018802 176 88 73;
1018802 144 69 14;
1019187 176 88 73;
1019187 176 88 127;
1019187 144 70 22;
1019229 128 69 64;
1019520 176 88 127;
1019520 176 88 31;
1019520 144 69 26;
1019536 128 70 64;
1019812 128 69 64;
1019864 176 88 31;
1019864 176 88 19;
1019864 144 68 16;
1020145 176 88 19;
1020145 176 88 101;
1020145 144 69 27;
1020151 128 68 64;
1023697 128 69 64;
1036697 176 88 101;
1036697 176 88 71;
1036697 144 67 20;
1040593 176 88 71;
1040593 176 88 92;
1040593 144 73 24;
1042744 128 73 64;
1042791 176 88 92;
1042791 176 88 93;
1042791 144 70 17;
1042947 128 67 64;
1044687 176 88 93;
1044687 176 88 88;
1044687 144 69 21;
1044833 128 70 64;
1057630 176 88 88;
1057630 176 88 7;
1057630 144 72 16;
1057817 128 69 64;
1059765 176 88 7;
1059765 176 88 62;
1059765 144 69 20;
1059822 128 72 64;
1064312 176 88 62;
1064312 176 88 52;
1064312 144 70 23;
1064364 128 69 64;
1064713 176 88 52;
1064713 176 88 41;
1064713 144 69 28;
1064750 128 70 64;
1065166 176 88 41;
1065166 176 88 115;
1065166 128 69 64;
1065166 144 67 28;
1070838 128 67 64;
1070848 176 88 115;
1070848 176 88 93;
1070848 144 73 37;
1073645 176 88 93;
1073645 176 88 114;
1073645 144 70 35;
1073661 128 73 64;
1074046 176 88 114;
1074046 176 88 103;
1074046 144 69 43;
1074062 128 70 64;
1079380 176 88 103;
1079380 176 88 74;
1079380 144 73 30;
1079385 176 88 74;
1079385 176 88 79;
1079385 144 67 34;
1079442 128 69 64;
1080197 176 88 79;
1080197 176 88 20;
1080197 128 67 64;
1080197 144 68 34;
1080234 128 73 64;
1084333 128 68 64;
1084348 176 88 20;
1084348 176 88 103;
1084348 144 67 25;
1084364 176 88 103;
1084364 176 88 82;
1084364 144 73 15;
1085812 128 67 64;
1086098 176 88 82;
1086098 176 88 49;
1086098 144 71 23;
1086114 128 73 64;
1089911 176 88 49;
1089911 176 88 18;
1089911 144 74 19;
1093875 128 71 64;
1093890 176 88 18;
1093890 176 88 95;
1093890 144 73 22;
1093989 128 74 64;
1095875 128 73 64;
1095901 144 72 19;
1108213 176 88 95;
1108213 176 88 75;
1108213 144 67 27;
1108234 128 72 64;
1108567 128 67 64;
1108598 176 88 75;
1108598 176 88 24;
1108598 144 68 26;
1109005 176 88 24;
1109005 176 88 79;
1109005 144 69 17;
1109046 128 68 64;
1111187 176 88 79;
1111187 176 88 92;
1111187 144 65 37;
1113239 176 88 92;
1113239 176 88 56;
1113239 144 64 36;
1113328 128 65 64;
1113369 128 69 64;
1116755 128 64 64;
1116765 176 88 56;
1116765 176 88 64;
1116765 144 69 35;
1116973 176 88 64;
1116973 176 88 82;
1116973 144 67 48;
1116984 128 69 64;
1117234 176 88 82;
1117234 176 88 54;
1117234 144 66 35;
1117239 128 67 64;
1117473 176 88 54;
1117473 176 88 52;
1117473 144 64 39;
1117479 128 66 64;
1122406 128 64 64;
1122598 176 88 52;
1122598 176 88 11;
1122598 144 64 25;
1123093 176 88 11;
1123093 176 88 32;
1123093 128 64 64;
1123093 144 64 18;
1123416 128 64 64;
1123619 176 88 32;
1123619 176 88 88;
1123619 144 64 20;
1127052 128 64 64;
1127114 176 88 88;
1127114 176 88 106;
1127114 144 69 24;
1128661 128 69 64;
1128677 176 88 106;
1128677 176 88 32;

1128677 144 67 25;
1132994 128 67 64;
1133036 176 88 32;
1133036 176 88 20;
1133036 144 69 30;
1133177 176 88 20;
1133177 176 88 5;
1133177 144 67 45;
1133203 128 69 64;
1133406 176 88 5;
1133406 176 88 14;
1133406 144 66 41;
1133416 128 67 64;
1137609 176 88 14;
1137609 176 88 26;
1137609 144 64 22;
1137671 128 66 64;
1144994 128 64 64;
1145526 176 88 26;
1145526 176 88 55;
1145526 144 67 40;
1145750 176 88 55;
1145750 176 88 46;
1145750 128 67 64;
1145750 144 69 42;
1147973 128 69 64;
1148010 176 88 46;
1148010 176 88 89;
1148010 144 71 25;
1150473 128 71 64;
1150505 176 88 89;
1150505 176 88 44;
1150505 144 76 31;
1152078 176 88 44;
1152078 176 88 55;
1152078 144 74 33;
1152213 128 76 64;
1155973 128 74 64;
1156026 176 88 55;
1156026 176 88 26;
1156026 144 76 21;
1159484 176 88 26;
1159484 176 88 79;
1159484 144 74 27;
1159598 128 76 64;
1161817 176 88 79;
1161817 176 88 80;
1161817 144 76 21;
1161953 128 74 64;
1165489 176 88 80;
1165489 176 88 108;
1165489 144 81 26;
1166328 128 81 64;
1167343 176 88 108;
1167343 176 88 18;
1167343 144 74 31;
1168203 128 76 64;
1168411 144 76 33;
1168536 128 74 64;
1171848 176 88 18;
1171848 176 88 90;
1171848 144 81 31;
1171880 128 76 64;
1172234 128 81 64;
1172286 176 88 90;
1172286 176 88 66;
1172286 144 79 25;
1174666 128 79 64;
1174723 176 88 66;
1174723 176 88 67;
1174723 144 81 29;
1174911 128 81 64;
1174916 176 88 67;
1174916 176 88 33;
1174916 144 79 26;
1175145 128 79 64;
1175161 176 88 33;
1175161 176 88 125;
1175161 144 78 29;
1176807 176 88 125;
1176807 176 88 43;
1176807 144 76 29;
1176854 128 78 64;
1178437 176 88 43;
1178437 176 88 42;
1178437 144 73 31;
1178505 128 76 64;
1180651 128 73 64;
1180666 176 88 42;
1180666 176 88 51;
1180666 144 76 28;
1182796 176 88 51;
1182796 176 88 59;
1182796 144 73 25;
1182880 176 88 59;
1182880 176 88 49;
1182880 144 69 19;
1182911 128 76 64;
1184791 128 69 64;
1185098 176 88 49;
1185098 176 88 110;
1185098 144 76 30;
1185182 128 73 64;
1187317 176 88 110;
1187317 176 88 30;
1187317 128 76 64;
1187317 144 71 18;
1187338 176 88 30;
1187338 176 88 40;
1187338 144 74 22;
1189395 128 71 64;
1189479 128 74 64;
1189526 176 88 40;
1189526 176 88 121;
1189526 144 76 19;
1191583 128 76 64;
1191614 176 88 121;
1191614 176 88 60;
1191614 144 71 31;
1191645 176 88 60;
1191645 176 88 35;
1191645 144 67 32;
1192463 128 67 64;
1193583 128 71 64;
1193802 176 88 35;
1193802 176 88 47;
1193802 144 67 23;
1193812 176 88 47;
1193812 176 88 33;
1193812 144 71 21;
1194895 176 88 33;
1194895 176 88 25;
1194895 144 70 18;
1194984 128 71 64;
1195994 128 67 64;
1196083 176 88 25;
1196083 176 88 19;
1196083 144 76 28;
1196140 128 70 64;
1199796 128 76 64;
1199828 176 88 19;
1199828 176 88 61;
1199828 144 81 25;
1199979 128 81 64;
1200036 176 88 61;
1200036 176 88 104;
1200036 144 79 33;
1200197 128 79 64;
1200270 176 88 104;
1200270 176 88 126;
1200270 144 81 36;
1201885 128 81 64;
1201916 176 88 126;
1201916 176 88 19;
1201916 144 78 29;
1204744 176 88 19;
1204744 176 88 16;
1204744 144 79 32;
1204750 128 78 64;
1206260 128 79 64;
1206291 176 88 16;
1206291 176 88 127;
1206291 144 78 19;
1206578 176 88 127;
1206578 176 88 45;
1206578 144 76 23;
1206593 128 78 64;
1212364 176 88 45;
1212364 176 88 107;
1212364 144 64 19;
1212437 128 76 64;
1214718 176 88 107;
1214718 176 88 53;
1214718 144 69 16;
1214760 128 64 64;
1216895 176 88 53;
1216895 176 88 56;
1216895 144 71 19;
1216968 128 69 64;
1223447 176 88 56;
1223447 176 88 32;
1223447 144 67 23;
1223791 128 71 64;
1227192 176 88 32;
1227192 176 88 4;
1227192 144 76 45;
1227296 128 67 64;
1228286 176 88 4;
1228286 176 88 21;
1228286 144 79 55;
1228348 128 76 64;
1230468 176 88 21;
1230468 176 88 119;
1230468 144 76 68;
1230578 128 79 64;
1234708 176 88 119;
1234708 176 88 87;
1234708 144 79 72;
1234843 128 76 64;
1237333 176 88 87;
1237333 176 88 103;
1237333 144 76 69;
1238973 128 76 64;
1239447 144 76 69;
1240364 128 76 64;
1241208 176 88 103;
1241208 176 88 100;
1241208 144 76 33;
1241369 128 76 64;
1241692 176 88 100;
1241692 176 88 67;
1241692 144 76 79;
1243854 128 76 64;
1246411 128 79 64;
1246427 176 88 67;
1246427 176 88 108;
1246427 144 81 51;
1246661 176 88 108;
1246661 176 88 56;
1246661 144 79 48;
1246697 128 81 64;
1246901 176 88 56;
1246901 176 88 83;
1246901 144 81 54;
1246937 128 79 64;
1248484 176 88 83;
1248484 176 88 41;
1248484 144 76 81;
1248588 128 81 64;
1252718 176 88 41;
1252718 176 88 89;
1252718 144 74 47;
1252812 128 76 64;
1254760 128 74 64;
1255416 176 88 89;
1255416 176 88 65;
1255416 144 76 18;
1256026 128 76 64;
1257604 176 88 65;
1257604 176 88 31;
1257604 144 76 30;
1257817 128 76 64;
1259859 176 88 31;
1259859 176 88 74;
1259859 144 76 37;
1259963 128 76 64;
1260270 176 88 74;
1260270 176 88 16;
1260270 144 81 73;
1260432 128 81 64;
1262317 176 88 16;
1262317 176 88 97;
1262317 144 79 43;
1262536 128 79 64;
1262572 176 88 97;
1262572 176 88 36;
1262572 144 81 44;
1262828 128 81 64;
1262848 176 88 36;
1262848 176 88 29;
1262848 144 79 44;
1266692 128 79 64;
1266984 176 88 29;
1266984 176 88 100;
1266984 144 76 51;
1268447 176 88 100;
1268447 176 88 0;
1268447 128 76 64;
1268447 144 76 1;
1268750 128 76 64;
1268973 176 88 0;
1268973 176 88 110;
1268973 144 74 65;
1269473 128 74 64;
1271015 176 88 110;
1271015 176 88 9;
1271015 144 72 60;
1271463 128 72 64;
1273078 176 88 9;
1273078 176 88 68;
1273078 144 69 59;
1273953 128 69 64;
1275067 176 88 68;
1275067 176 88 66;
1275067 144 67 76;
1276552 176 88 66;
1276552 176 88 43;
1276552 128 67 64;
1276552 144 69 48;
1276750 176 88 43;
1276750 176 88 121;
1276750 144 67 71;
1276781 128 69 64;
1277015 176 88 121;
1277015 176 88 47;
1277015 144 69 83;
1277114 128 67 64;
1278479 176 88 47;
1278479 176 88 119;
1278479 144 66 65;
1278552 128 69 64;
1279171 128 66 64;
1280895 176 88 119;
1280895 176 88 6;
1280895 144 66 88;
1281671 128 66 64;
1282927 176 88 6;
1282927 176 88 34;
1282927 144 66 82;
1283609 128 66 64;
1283807 176 88 34;
1283807 176 88 21;
1283807 144 66 73;
1284208 176 88 21;
1284208 176 88 107;
1284208 144 62 80;
1284223 128 66 64;
1284645 128 62 64;
1286088 176 88 107;
1286088 176 88 78;
1286088 144 64 32;
1286703 128 64 64;
1287546 176 88 78;
1287546 176 88 21;
1287546 144 64 40;
1287973 176 88 21;
1287973 176 88 39;
1287973 144 67 40;
1288317 128 64 64;
1288348 128 67 64;
1289458 176 88 39;
1289458 176 88 99;
1289458 144 67 52;
1289468 176 88 99;
1289468 176 88 41;
1289468 144 64 38;
1290010 128 64 64;
1290208 128 67 64;
1291296 176 88 41;
1291296 176 88 103;
1291296 144 66 40;
1291328 176 88 103;

1291328 176 88 34;
1291328 144 69 41;
1291734 128 66 64;
1291984 128 69 64;
1293208 176 88 34;
1293208 176 88 104;
1293208 144 67 48;
1293218 176 88 104;
1293218 176 88 39;
1293218 144 64 31;
1293317 128 64 64;
1293489 128 67 64;
1293630 176 88 39;
1293630 176 88 126;
1293630 144 66 37;
1293635 176 88 126;
1293635 176 88 117;
1293635 144 62 55;
1293843 128 66 64;
1293859 128 62 64;
1297859 176 88 117;
1297859 176 88 29;
1297859 144 71 29;
1298281 128 71 64;
1299708 176 88 29;
1299708 176 88 121;
1299708 144 71 24;
1300687 128 71 64;
1300703 176 88 121;
1300703 176 88 28;
1300703 144 69 20;
1302739 128 69 64;
1302760 176 88 28;
1302760 176 88 101;
1302760 144 71 22;
1304406 128 71 64;
1304625 176 88 101;
1304625 176 88 8;
1304625 144 83 25;
1306567 128 83 64;
1306572 176 88 8;
1306572 176 88 2;
1306572 144 81 33;
1308989 128 81 64;
1309072 176 88 2;
1309072 176 88 83;
1309072 144 79 21;
1309229 128 79 64;
1309281 176 88 83;
1309281 176 88 118;
1309281 144 81 30;
1309510 128 81 64;
1309546 176 88 118;
1309546 176 88 79;
1309546 144 79 31;
1310536 128 79 64;
1310588 176 88 79;
1310588 176 88 121;
1310588 144 78 21;
1311625 128 78 64;
1313578 176 88 121;
1313578 176 88 114;
1313578 144 76 19;
1313864 128 76 64;
1315526 176 88 114;
1315526 176 88 124;
1315526 144 76 33;
1315661 128 76 64;
1316020 176 88 124;
1316020 176 88 10;
1316020 144 74 33;
1316234 128 74 64;
1320369 176 88 10;
1320369 176 88 107;
1320369 144 74 26;
1320635 128 74 64;
1321812 176 88 107;
1321812 176 88 55;
1321812 144 72 32;
1322015 128 72 64;
1322786 176 88 55;
1322786 176 88 100;
1322786 144 71 46;
1323062 128 71 64;
1323703 176 88 100;
1323703 176 88 18;
1323703 144 69 46;
1324041 128 69 64;
1324557 176 88 18;
1324557 176 88 102;
1324557 144 67 63;
1324911 128 67 64;
1328062 176 88 102;
1328062 176 88 99;
1328062 144 76 66;
1328083 176 88 99;
1328083 176 88 6;
1328083 144 64 42;
1328114 176 88 6;
1328114 176 88 59;
1328114 144 65 32;
1328177 128 65 64;
1328630 128 64 64;
1328687 128 76 64;
1329958 176 88 59;
1329958 176 88 81;
1329958 144 76 74;
1329968 176 88 81;
1329968 176 88 47;
1329968 144 64 53;
1329973 176 88 47;
1329973 176 88 24;
1329973 144 65 57;
1330343 128 65 64;
1330348 128 64 64;
1330453 128 76 64;
1331802 176 88 24;
1331802 176 88 46;
1331802 144 67 72;
1331802 144 69 66;
1332281 128 69 64;
1332286 128 67 64;
1332708 176 88 46;
1332708 176 88 115;
1332708 144 66 64;
1332739 176 88 115;
1332739 176 88 55;
1332739 144 64 64;
1333109 128 64 64;
1333114 128 66 64;
1335526 176 88 55;
1335526 176 88 94;
1335526 144 64 82;
1335536 176 88 94;
1335536 176 88 56;
1335536 144 67 74;
1336114 128 64 64;
1336161 128 67 64;
1337442 176 88 56;
1337442 176 88 104;
1337442 144 65 71;
1337453 176 88 104;
1337453 176 88 121;
1337453 144 64 74;
1339963 128 64 64;
1339984 128 65 64;
1341083 176 88 121;
1341083 176 88 4;
1341083 144 65 29;
1341104 176 88 4;
1341104 176 88 43;
1341104 144 64 27;
1342015 176 88 43;
1342015 176 88 0;
1342015 128 64 64;
1342015 144 64 1;
1342401 128 65 64;
1342411 128 64 64;
1342911 176 88 0;
1342911 176 88 35;
1342911 144 65 25;
1342911 176 88 35;
1342911 176 88 120;
1342911 144 64 25;
1344755 128 64 64;
1344953 128 65 64;
1346791 176 88 120;
1346791 176 88 27;
1346791 144 65 19;
1346791 176 88 27;
1346791 176 88 118;
1346791 144 64 18;
1349031 128 65 64;
1350729 176 88 118;
1350729 176 88 26;
1350729 144 62 26;
1350828 128 64 64;
1352718 128 62 64;
1352734 176 88 26;
1352734 176 88 104;
1352734 144 60 18;
1354864 144 59 22;
1354984 128 60 64;
1358500 128 59 64;
1546578 176 88 104;
1546578 176 88 49;
1546578 144 62 2;
1549062 176 88 49;
1549062 176 88 58;
1549062 144 70 20;
1549828 128 62 64;
1552640 176 88 58;
1552640 176 88 2;
1552640 144 67 19;
1552666 128 70 64;
1555229 176 88 2;
1555229 176 88 21;
1555229 144 69 19;
1555369 128 67 64;
1558244 176 88 21;
1558244 176 88 48;
1558244 144 62 8;
1558427 128 69 64;
1560026 176 88 48;
1560026 176 88 45;
1560026 144 72 19;
1560130 128 62 64;
1561765 128 72 64;
1561765 176 88 45;
1561765 176 88 106;
1561765 144 70 23;
1563776 128 70 64;
1563812 176 88 106;
1563812 176 88 123;
1563812 144 72 23;
1567781 128 72 64;
1567843 176 88 123;
1567843 176 88 9;
1567843 144 69 5;
1574968 128 69 64;
1576901 176 88 9;
1576901 176 88 71;
1576901 144 67 14;
1578750 176 88 71;
1578750 176 88 23;
1578750 144 75 23;
1580354 128 75 64;
1580588 176 88 23;
1580588 176 88 33;
1580588 144 74 26;
1581854 128 67 64;
1582807 128 74 64;
1582875 176 88 33;
1582875 176 88 30;
1582875 144 72 13;
1589552 128 72 64;
1589677 176 88 30;
1589677 176 88 75;
1589677 144 67 20;
1591385 176 88 75;
1591385 176 88 7;
1591385 144 63 23;
1591526 128 67 64;
1598541 176 88 7;
1598541 176 88 0;
1598541 144 67 1;
1598640 128 63 64;
1599098 128 67 64;
1599260 144 62 1;
1601145 176 88 0;
1601145 176 88 91;
1601145 144 67 14;
1601625 128 62 64;
1607427 128 67 64;
1608843 176 88 91;
1608843 176 88 96;
1608843 144 65 24;
1609229 128 65 64;
1609244 176 88 96;
1609244 176 88 81;
1609244 144 63 23;
1616958 128 63 64;
1617020 176 88 81;
1617020 176 88 21;
1617020 144 62 20;
1620046 176 88 21;
1620046 176 88 13;
1620046 144 65 21;
1620208 128 62 64;
1620854 128 65 64;
1620880 176 88 13;
1620880 176 88 21;
1620880 144 63 17;
1621244 128 63 64;
1621307 176 88 21;
1621307 176 88 86;
1621307 144 62 23;
1624744 128 62 64;
1630968 176 88 86;
1630968 176 88 73;
1630968 144 63 10;
1631000 176 88 73;
1631000 176 88 93;
1631000 144 70 17;
1632125 128 70 64;
1632177 176 88 93;
1632177 176 88 118;
1632177 144 67 17;
1634307 176 88 118;
1634307 176 88 21;
1634307 144 68 13;
1634364 128 67 64;
1636281 128 63 64;
1636567 128 68 64;
1636677 176 88 21;
1636677 176 88 0;
1636677 144 67 1;
1637104 128 67 64;
1637151 176 88 0;
1637151 176 88 110;
1637151 144 65 25;
1642645 128 65 64;
1643500 176 88 110;
1643500 176 88 111;
1643500 144 63 13;
1643500 176 88 111;
1643500 176 88 38;
1643500 144 67 22;
1644723 128 67 64;
1644734 128 63 64;
1644807 176 88 38;
1644807 176 88 52;
1644807 144 65 27;
1645171 128 65 64;
1645213 176 88 52;
1645213 176 88 26;
1645213 144 63 26;
1646322 176 88 26;
1646322 176 88 70;
1646322 144 70 12;
1651578 128 63 64;
1651697 128 70 64;
1652072 176 88 70;
1652072 176 88 55;
1652072 144 65 30;
1652291 128 65 64;
1652322 176 88 55;
1652322 176 88 23;
1652322 144 63 28;
1652567 128 63 64;
1652619 176 88 23;
1652619 176 88 8;
1652619 144 62 24;
1654453 128 62 64;
1654484 176 88 8;
1654484 176 88 12;
1654484 144 60 28;
1660031 128 60 64;
1660833 176 88 12;
1660833 176 88 0;
1660833 144 67 1;
1663781 128 67 64;

1664000 176 88 0;
1664000 176 88 87;
1664000 144 68 19;
1665177 128 68 64;
1665244 176 88 87;
1665244 176 88 115;
1665244 144 67 22;
1666401 128 67 64;
1666437 176 88 115;
1666437 176 88 118;
1666437 144 68 22;
1666536 128 68 64;
1666557 176 88 118;
1666557 176 88 30;
1666557 144 70 29;
1666723 176 88 30;
1666723 176 88 57;
1666723 128 70 64;
1666723 144 68 25;
1667703 176 88 57;
1667703 176 88 20;
1667703 144 67 18;
1667734 128 68 64;
1674395 176 88 20;
1674395 176 88 124;
1674395 144 70 23;
1674416 128 67 64;
1674791 128 70 64;
1674802 176 88 124;
1674802 176 88 86;
1674802 144 75 24;
1675880 176 88 86;
1675880 176 88 104;
1675880 144 74 25;
1675885 128 75 64;
1678588 176 88 104;
1678588 176 88 77;
1678588 144 72 18;
1678713 128 74 64;
1680265 176 88 77;
1680265 176 88 14;
1680265 144 70 24;
1680447 128 72 64;
1681385 128 70 64;
1681406 176 88 14;
1681406 176 88 32;
1681406 144 68 19;
1681927 128 68 64;
1681947 176 88 32;
1681947 176 88 37;
1681947 144 67 25;
1684067 128 67 64;
1684161 176 88 37;
1684161 176 88 36;
1684161 144 65 15;
1685338 128 65 64;
1685390 176 88 36;
1685390 176 88 94;
1685390 144 63 12;
1687661 128 63 64;
1687666 176 88 94;
1687666 176 88 25;
1687666 144 65 15;
1694817 128 65 64;
1699614 176 88 25;
1699614 176 88 0;
1699614 144 67 1;
1704145 128 67 64;
1704348 176 88 0;
1704348 176 88 97;
1704348 144 67 17;
1705630 128 67 64;
1705906 176 88 97;
1705906 176 88 79;
1705906 144 67 25;
1706588 128 67 64;
1706635 176 88 79;
1706635 176 88 41;
1706635 144 68 18;
1707145 128 68 64;
1707177 176 88 41;
1707177 176 88 114;
1707177 144 67 26;
1707317 128 67 64;
1707322 176 88 114;
1707322 176 88 40;
1707322 144 68 28;
1707838 128 68 64;
1707838 176 88 40;
1707838 176 88 22;
1707838 144 70 26;
1708453 128 70 64;
1708989 176 88 22;
1708989 176 88 14;
1708989 144 72 22;
1710791 176 88 14;
1710791 176 88 5;
1710791 144 70 22;
1710812 128 72 64;
1711651 128 70 64;
1711687 176 88 5;
1711687 176 88 115;
1711687 144 72 25;
1712000 128 72 64;
1712020 176 88 115;
1712020 176 88 54;
1712020 144 68 15;
1712317 128 68 64;
1712354 176 88 54;
1712354 176 88 15;
1712354 144 67 25;
1713166 128 67 64;
1713182 176 88 15;
1713182 176 88 113;
1713182 144 72 29;
1713916 128 72 64;
1713937 176 88 113;
1713937 176 88 10;
1713937 144 74 34;
1718432 128 74 64;
1718432 176 88 10;
1718432 176 88 86;
1718432 144 72 19;
1720171 128 72 64;
1720473 176 88 86;
1720473 176 88 47;
1720473 144 75 30;
1720552 176 88 47;
1720552 176 88 39;
1720552 144 72 18;
1721354 128 72 64;
1721927 128 75 64;
1724687 176 88 39;
1724687 176 88 0;
1724687 144 75 17;
1724994 128 75 64;
1727239 176 88 0;
1727239 176 88 41;
1727239 144 75 23;
1727677 128 75 64;
1727703 176 88 41;
1727703 176 88 5;
1727703 144 74 25;
1731682 128 74 64;
1732229 176 88 5;
1732229 176 88 91;
1732229 144 74 14;
1733161 128 74 64;
1733208 176 88 91;
1733208 176 88 67;
1733208 144 72 19;
1733963 128 72 64;
1734203 176 88 67;
1734203 176 88 73;
1734203 144 72 24;
1735052 128 72 64;
1735229 176 88 73;
1735229 176 88 5;
1735229 144 70 24;
1735921 128 70 64;
1736354 176 88 5;
1736354 176 88 24;
1736354 144 72 33;
1737015 128 72 64;
1737453 176 88 24;
1737453 176 88 94;
1737453 144 72 18;
1738250 128 72 64;
1738473 176 88 94;
1738473 176 88 112;
1738473 144 72 18;
1739312 128 72 64;
1739541 176 88 112;
1739541 176 88 111;
1739541 144 72 17;
1740708 176 88 111;
1740708 176 88 13;
1740708 128 72 64;
1740708 144 72 12;
1741380 128 72 64;
1741630 176 88 13;
1741630 176 88 115;
1741630 144 72 19;
1742562 128 72 64;
1742697 176 88 115;
1742697 176 88 70;
1742697 144 72 28;
1743484 128 72 64;
1743734 176 88 70;
1743734 176 88 72;
1743734 144 72 25;
1744583 128 72 64;
1744890 176 88 72;
1744890 176 88 88;
1744890 144 72 18;
1744927 176 88 88;
1744927 176 88 112;
1744927 144 69 14;
1745432 128 69 64;
1745703 128 72 64;
1745942 176 88 112;
1745942 176 88 49;
1745942 144 72 25;
1746781 128 72 64;
1747078 176 88 49;
1747078 176 88 0;
1747078 144 72 17;
1747875 128 72 64;
1748072 176 88 0;
1748072 176 88 96;
1748072 144 72 22;
1748781 128 72 64;
1749140 176 88 96;
1749140 176 88 118;
1749140 144 72 28;
1749156 176 88 118;
1749156 176 88 69;
1749156 144 71 30;
1749755 128 71 64;
1749958 128 72 64;
1750182 176 88 69;
1750182 176 88 91;
1750182 144 72 27;
1751031 128 72 64;
1751265 176 88 91;
1751265 176 88 34;
1751265 144 72 42;
1752098 128 72 64;
1752432 176 88 34;
1752432 176 88 38;
1752432 144 72 22;
1753161 128 72 64;
1753510 176 88 38;
1753510 176 88 59;
1753510 144 72 29;
1753520 176 88 59;
1753520 176 88 80;
1753520 144 67 33;
1754145 128 72 64;
1754177 128 67 64;
1754567 176 88 80;
1754567 176 88 54;
1754567 144 72 23;
1755312 128 72 64;
1755645 176 88 54;
1755645 176 88 49;
1755645 144 72 26;
1756312 128 72 64;
1756692 176 88 49;
1756692 176 88 126;
1756692 144 72 33;
1757572 128 72 64;
1757817 176 88 126;
1757817 176 88 16;
1757817 144 66 36;
1757828 176 88 16;
1757828 176 88 27;
1757828 144 72 38;
1758536 128 72 64;
1758614 128 66 64;
1758885 176 88 27;
1758885 176 88 15;
1758885 144 72 29;
1759677 128 72 64;
1760062 176 88 15;
1760062 176 88 22;
1760062 144 72 41;
1760677 128 72 64;
1761010 176 88 22;
1761010 176 88 97;
1761010 144 72 39;
1761015 176 88 97;
1761015 176 88 14;
1761015 144 62 33;
1761619 128 62 64;
1761755 128 72 64;
1762005 176 88 14;
1762005 176 88 18;
1762005 144 72 40;
1762723 128 72 64;
1763062 176 88 18;
1763062 176 88 123;
1763062 144 72 42;
1763067 176 88 123;
1763067 176 88 72;
1763067 144 67 39;
1763708 128 67 64;
1763812 128 72 64;
1764104 176 88 72;
1764104 176 88 46;
1764104 144 72 45;
1764140 176 88 46;
1764140 176 88 43;
1764140 144 66 39;
1764796 128 66 64;
1764822 128 72 64;
1765104 176 88 43;
1765104 176 88 93;
1765104 144 72 20;
1765125 176 88 93;
1765125 176 88 125;
1765125 144 64 37;
1765786 128 64 64;
1765880 128 72 64;
1766166 176 88 125;
1766166 176 88 98;
1766166 144 67 32;
1766182 176 88 98;
1766182 176 88 86;
1766182 144 72 36;
1767244 128 67 64;
1767286 176 88 86;
1767286 176 88 56;
1767286 144 65 22;
1767885 128 65 64;
1767911 128 72 64;
1768364 176 88 56;
1768364 176 88 97;
1768364 144 72 39;
1768395 176 88 97;
1768395 176 88 77;
1768395 144 66 36;
1769026 128 72 64;
1769135 128 66 64;
1769442 176 88 77;
1769442 176 88 63;
1769442 144 72 20;
1769458 144 62 32;
1769994 128 62 64;
1770260 128 72 64;
1770619 176 88 63;
1770619 176 88 15;
1770619 144 72 28;
1771614 176 88 15;
1771614 176 88 123;
1771614 144 69 24;
1771979 128 72 64;
1772520 128 69 64;
1772723 176 88 123;
1772723 176 88 109;
1772723 144 72 41;
1773463 128 72 64;

1773817 176 88 109;
1773817 176 88 108;
1773817 144 72 30;
1773822 176 88 108;
1773822 176 88 43;
1773822 144 67 34;
1774598 128 67 64;
1774609 128 72 64;
1774890 176 88 43;
1774890 176 88 107;
1774890 144 68 28;
1774906 176 88 107;
1774906 176 88 123;
1774906 144 72 29;
1775609 128 68 64;
1775682 128 72 64;
1775885 176 88 123;
1775885 176 88 80;
1775885 144 72 18;
1775932 176 88 80;
1775932 176 88 86;
1775932 144 69 32;
1776677 128 69 64;
1776682 128 72 64;
1777026 176 88 86;
1777026 176 88 41;
1777026 144 72 29;
1777078 176 88 41;
1777078 176 88 103;
1777078 144 68 30;
1777692 128 68 64;
1777723 128 72 64;
1778161 176 88 103;
1778161 176 88 80;
1778161 144 72 10;
1778182 176 88 80;
1778182 176 88 43;
1778182 144 67 28;
1780911 128 67 64;
1781135 128 72 64;
1781567 176 88 43;
1781567 176 88 6;
1781567 144 72 15;
1783421 128 72 64;
1783723 176 88 6;
1783723 176 88 33;
1783723 144 72 17;
1785770 128 72 64;
1785989 176 88 33;
1785989 176 88 82;
1785989 144 72 22;
1787005 128 72 64;
1787250 176 88 82;
1787250 176 88 66;
1787250 144 73 31;
1787276 176 88 66;
1787276 176 88 29;
1787276 144 72 16;
1788005 128 73 64;
1788041 128 72 64;
1788348 176 88 29;
1788348 176 88 32;
1788348 144 72 15;
1790338 128 72 64;
1790630 176 88 32;
1790630 176 88 18;
1790630 144 72 26;
1792614 128 72 64;
1792963 176 88 18;
1792963 176 88 112;
1792963 144 72 14;
1793796 128 72 64;
1795015 176 88 112;
1795015 176 88 123;
1795015 144 72 23;
1795843 128 72 64;
1796630 176 88 123;
1796630 176 88 75;
1796630 144 72 21;
1796786 128 72 64;
1797166 176 88 75;
1797166 176 88 33;
1797171 144 72 20;
1797192 176 88 33;
1797192 176 88 36;
1797192 144 73 15;
1797447 128 72 64;
1797578 128 73 64;
1797697 176 88 36;
1797697 176 88 121;
1797697 144 72 26;
1798093 128 72 64;
1798296 176 88 121;
1798296 176 88 12;
1798296 144 72 14;
1798348 176 88 12;
1798348 176 88 50;
1798348 144 73 15;
1798546 128 72 64;
1798802 176 88 50;
1798802 176 88 67;
1798802 144 72 29;
1798812 128 73 64;
1800192 128 72 64;
1800562 176 88 67;
1800562 176 88 83;
1800562 144 73 33;
1800640 176 88 83;
1800640 176 88 73;
1800640 144 72 14;
1800750 128 72 64;
1801390 128 73 64;
1802875 176 88 73;
1802875 176 88 32;
1802875 144 73 27;
1804343 128 73 64;
1804598 176 88 32;
1804598 176 88 101;
1804598 144 72 23;
1806072 128 72 64;
1806369 176 88 101;
1806369 176 88 18;
1806369 144 73 31;
1807531 176 88 18;
1807531 176 88 115;
1807531 144 72 33;
1807567 128 73 64;
1808692 176 88 115;
1808692 176 88 4;
1808692 144 84 47;
1809541 128 72 64;
1809796 176 88 4;
1809796 176 88 106;
1809796 144 72 27;
1810421 128 72 64;
1810817 176 88 106;
1810817 176 88 0;
1810817 144 72 35;
1810890 128 84 64;
1812994 176 88 0;
1812994 176 88 64;
1812994 144 73 40;
1813026 128 72 64;
1815250 144 72 48;
1815265 128 73 64;
1817489 128 72 64;
1817520 176 88 64;
1817520 176 88 106;
1817520 144 74 30;
1818562 128 74 64;
1818614 176 88 106;
1818614 176 88 74;
1818614 144 73 23;
1819697 128 73 64;
1819713 176 88 74;
1819713 176 88 15;
1819713 144 72 28;
1826432 128 72 64;
1826729 176 88 15;
1826729 176 88 1;
1826729 144 72 20;
1828963 128 72 64;
1829171 176 88 1;
1829171 176 88 14;
1829171 144 72 14;
1831515 128 72 64;
1831796 176 88 14;
1831796 176 88 67;
1831796 144 72 24;
1833651 128 72 64;
1834135 176 88 67;
1834135 176 88 23;
1834135 144 72 32;
1834859 128 72 64;
1834890 176 88 23;
1834890 176 88 24;
1834890 144 68 16;
1835552 128 68 64;
1835557 176 88 24;
1835557 176 88 28;
1835557 144 72 22;
1836552 128 72 64;
1836760 176 88 28;
1836760 176 88 56;
1836760 144 72 27;
1837609 128 72 64;
1837953 176 88 56;
1837953 176 88 84;
1837953 144 72 15;
1838718 128 72 64;
1839171 176 88 84;
1839171 176 88 123;
1839171 144 72 23;
1843796 128 72 64;
1844203 176 88 123;
1844203 176 88 26;
1844203 144 72 19;
1844848 176 88 26;
1844848 176 88 44;
1844848 144 65 18;
1845432 176 88 44;
1845432 176 88 21;
1845432 144 69 16;
1845645 128 69 64;
1846083 128 65 64;
1846244 176 88 21;
1846244 176 88 3;
1846244 144 68 10;
1846572 128 68 64;
1849098 128 72 64;
1849453 176 88 3;
1849453 176 88 73;
1849453 144 72 13;
1852109 128 72 64;
1852354 176 88 73;
1852354 176 88 71;
1852354 144 72 13;
1855619 176 88 71;
1855619 176 88 25;
1855619 144 70 17;
1857296 128 72 64;
1857869 176 88 25;
1857869 176 88 6;
1857869 144 72 18;
1859427 128 70 64;
1860546 176 88 6;
1860546 176 88 28;
1860546 144 70 16;
1862380 128 70 64;
1865125 128 72 64;
1867255 176 88 28;
1867255 176 88 95;
1867255 144 70 10;
1871494 128 70 64;
1871932 176 88 95;
1871932 176 88 87;
1871932 144 70 12;
1877000 128 70 64;
1877364 176 88 87;
1877364 176 88 31;
1877364 144 70 13;
1879041 128 70 64;
1879072 176 88 31;
1879072 176 88 41;
1879072 144 72 14;
1882859 176 88 41;
1882859 176 88 58;
1882859 144 70 9;
1882994 128 72 64;
1885052 176 88 58;
1885052 176 88 12;
1885052 144 65 13;
1885192 128 70 64;
1889947 128 65 64;
1926291 176 88 12;
1926291 176 88 25;
1926291 144 79 47;
1926312 176 88 25;
1926312 176 88 124;
1926312 144 81 47;
1926458 128 79 64;
1926473 128 81 64;
1928390 176 88 124;
1928390 176 88 105;
1928390 144 76 76;
1928395 176 88 105;
1928395 176 88 108;
1928395 144 77 76;
1928572 128 76 64;
1928583 128 77 64;
1930218 176 88 108;
1930218 176 88 61;
1930218 144 81 79;
1930218 176 88 61;
1930218 176 88 65;
1930218 144 79 77;
1931437 128 79 64;
1931458 128 81 64;
1931713 176 88 65;
1931713 176 88 4;
1931713 144 81 62;
1931718 176 88 4;
1931718 176 88 28;
1931718 144 79 53;
1932833 128 79 64;
1932869 128 81 64;
1933161 176 88 28;
1933161 176 88 61;
1933161 144 81 79;
1933166 176 88 61;
1933166 176 88 79;
1933166 144 79 69;
1934281 128 79 64;
1934328 128 81 64;
1934583 176 88 79;
1934583 176 88 47;
1934583 144 79 71;
1934583 176 88 47;
1934583 176 88 60;
1934583 144 81 66;
1934786 128 79 64;
1934786 128 81 64;
1935010 176 88 60;
1935010 176 88 24;
1935010 144 79 69;
1935010 176 88 24;
1935010 176 88 3;
1935010 144 77 57;
1935015 176 88 3;
1935015 176 88 124;
1935015 144 76 56;
1935203 128 76 64;
1935208 128 77 64;
1935208 128 79 64;
1937619 176 88 124;
1937619 176 88 25;
1937619 144 76 46;
1937645 176 88 25;
1937645 176 88 127;
1937645 144 74 45;
1938536 128 74 64;
1938604 128 76 64;
1938927 176 88 127;
1938927 176 88 88;
1938927 144 74 52;
1938973 176 88 88;
1938973 176 88 65;
1938973 144 76 55;
1940572 128 74 64;
1940729 128 76 64;
1941604 176 88 65;
1941604 176 88 36;
1941604 144 81 44;
1941656 176 88 36;
1941656 176 88 33;
1941656 144 83 46;
1943645 128 81 64;
1943671 128 83 64;
1946255 176 88 33;
1946255 176 88 93;
1946255 144 72 29;
1946276 176 88 93;

1946276 176 88 46;
1946276 144 76 36;
1946364 128 72 64;
1946395 128 76 64;
1947338 176 88 46;
1947338 176 88 53;
1947338 144 76 38;
1947348 176 88 53;
1947348 176 88 62;
1947348 144 72 28;
1947401 128 76 64;
1947421 128 72 64;
1947489 176 88 62;
1947489 176 88 54;
1947489 144 76 42;
1947500 176 88 54;
1947500 176 88 91;
1947500 144 72 53;
1947572 128 72 64;
1947604 128 76 64;
1950067 176 88 91;
1950067 176 88 112;
1950067 144 74 31;
1950177 128 74 64;
1953078 176 88 112;
1953078 176 88 92;
1953078 144 77 54;
1953151 128 77 64;
1956718 176 88 92;
1956718 176 88 57;
1956718 144 81 52;
1956744 176 88 57;
1956744 176 88 44;
1956744 144 79 33;
1956812 128 81 64;
1956817 128 79 64;
1957046 176 88 44;
1957046 176 88 47;
1957046 144 79 47;
1957057 176 88 47;
1957057 176 88 58;
1957057 144 77 38;
1957135 128 79 64;
1957156 128 77 64;
1958854 176 88 58;
1958854 176 88 25;
1958854 144 81 78;
1958859 176 88 25;
1958859 176 88 51;
1958859 144 83 74;
1958947 128 83 64;
1958968 128 81 64;
1959078 176 88 51;
1959078 176 88 84;
1959078 144 79 63;
1959083 176 88 84;
1959083 176 88 5;
1959083 144 81 45;
1959177 128 81 64;
1959250 128 79 64;
1963296 176 88 5;
1963296 176 88 16;
1963296 144 80 46;
1963307 176 88 16;
1963307 176 88 36;
1963307 144 78 38;
1963359 128 80 64;
1963364 128 78 64;
1965468 176 88 36;
1965468 176 88 126;
1965468 144 80 46;
1965468 176 88 126;
1965468 176 88 36;
1965468 144 78 48;
1965531 128 78 64;
1965531 128 80 64;
1966046 176 88 36;
1966046 176 88 42;
1966046 144 78 55;
1966072 176 88 42;
1966072 176 88 107;
1966072 144 76 35;
1966145 128 76 64;
1966177 128 78 64;
1968218 176 88 107;
1968218 176 88 16;
1968218 144 76 62;
1968218 176 88 16;
1968218 176 88 87;
1968218 144 77 57;
1968291 128 76 64;
1968296 128 77 64;
1968656 176 88 87;
1968656 176 88 123;
1968656 144 75 48;
1968671 176 88 123;
1968671 176 88 119;
1968671 144 76 65;
1968791 128 75 64;
1968802 128 76 64;
1970520 176 88 119;
1970520 176 88 123;
1970520 144 74 67;
1970541 176 88 123;
1970541 176 88 108;
1970541 144 71 44;
1970614 128 71 64;
1970640 128 74 64;
1972390 144 73 62;
1972395 176 88 108;
1972395 176 88 91;
1972395 144 72 53;
1972463 128 72 64;
1972473 128 73 64;
1973421 176 88 91;
1973421 176 88 27;
1973421 144 71 63;
1973432 176 88 27;
1973432 176 88 4;
1973432 144 72 62;
1973505 128 71 64;
1973510 128 72 64;
1975713 176 88 4;
1975713 176 88 39;
1975713 144 72 50;
1975713 176 88 39;
1975713 176 88 52;
1975713 144 71 49;
1975796 128 72 64;
1975796 128 71 64;
1976437 176 88 52;
1976437 176 88 76;
1976437 144 67 51;
1976437 176 88 76;
1976437 176 88 29;
1976437 144 69 49;
1976536 128 67 64;
1976541 128 69 64;
1977255 176 88 29;
1977255 176 88 34;
1977255 144 62 51;
1977265 176 88 34;
1977265 176 88 52;
1977265 144 64 49;
1977348 128 64 64;
1977348 128 62 64;
1978703 176 88 52;
1978703 176 88 116;
1978703 144 74 52;
1978708 176 88 116;
1978708 176 88 10;
1978708 144 76 56;
1978828 128 76 64;
1978833 128 74 64;
1981875 176 88 10;
1981875 176 88 116;
1981875 144 74 40;
1981880 176 88 116;
1981880 176 88 54;
1981880 144 76 41;
1981947 128 74 64;
1981947 128 76 64;
1982260 176 88 54;
1982260 176 88 108;
1982260 144 76 48;
1982260 176 88 108;
1982260 176 88 85;
1982260 144 74 45;
1982333 128 76 64;
1982333 128 74 64;
1982635 176 88 85;
1982635 176 88 8;
1982635 144 71 50;
1982635 144 72 51;
1982718 128 71 64;
1982718 128 72 64;
1983010 176 88 8;
1983010 176 88 79;
1983010 144 71 46;
1983010 176 88 79;
1983010 176 88 26;
1983010 144 72 49;
1983093 128 72 64;
1983098 128 71 64;
1983395 176 88 26;
1983395 176 88 85;
1983395 144 67 56;
1983401 176 88 85;
1983401 176 88 89;
1983401 144 69 51;
1983515 128 67 64;
1983520 128 69 64;
1988166 176 88 89;
1988166 176 88 16;
1988166 144 78 51;
1988182 176 88 16;
1988182 176 88 104;
1988182 144 77 41;
1988250 128 77 64;
1988286 128 78 64;
1990625 176 88 104;
1990625 176 88 65;
1990625 144 75 34;
1990630 176 88 65;
1990630 176 88 74;
1990630 144 76 51;
1990687 128 76 64;
1990692 128 75 64;
1991031 176 88 74;
1991031 176 88 92;
1991031 144 75 41;
1991031 144 76 54;
1991098 128 76 64;
1991098 128 75 64;
1991447 176 88 92;
1991447 176 88 112;
1991447 144 75 46;
1991500 176 88 112;
1991500 176 88 2;
1991500 144 74 19;
1991562 128 75 64;
1991572 128 74 64;
1993848 176 88 2;
1993848 176 88 8;
1993848 144 81 53;
1993859 176 88 8;
1993859 176 88 55;
1993859 144 83 52;
1993932 128 83 64;
1993937 128 81 64;
1995036 176 88 55;
1995036 176 88 9;
1995036 144 83 45;
1995062 176 88 9;
1995062 176 88 18;
1995062 144 81 23;
1995098 128 83 64;
1995119 128 81 64;
1995203 176 88 18;
1995203 176 88 112;
1995203 144 79 56;
1995270 128 79 64;
1995406 176 88 112;
1995406 176 88 55;
1995406 144 81 62;
1995562 128 81 64;
2003104 176 88 55;
2003104 176 88 39;
2003104 144 74 31;
2003197 128 74 64;
2003947 176 88 39;
2003947 176 88 84;
2003947 144 74 46;
2004026 128 74 64;
2006057 176 88 84;
2006057 176 88 16;
2006057 144 74 53;
2006098 176 88 16;
2006098 176 88 123;
2006098 144 77 53;
2006130 128 74 64;
2006229 128 77 64;
2008208 176 88 123;
2008208 176 88 4;
2008208 144 76 49;
2008270 128 76 64;
2010505 176 88 4;
2010505 176 88 122;
2010505 144 74 48;
2010578 176 88 122;
2010578 176 88 39;
2010578 144 77 50;
2010598 128 74 64;
2010671 176 88 39;
2010671 176 88 127;
2010671 144 76 51;
2010723 128 77 64;
2010869 128 76 64;
2011036 176 88 127;
2011036 176 88 83;
2011036 144 75 64;
2011192 128 75 64;
2016411 176 88 83;
2016411 176 88 17;
2016411 144 74 49;
2016500 128 74 64;
2018302 176 88 17;
2018302 176 88 126;
2018302 144 74 70;
2018364 128 74 64;
2018515 176 88 126;
2018515 176 88 30;
2018515 144 72 61;
2018583 128 72 64;
2019093 176 88 30;
2019093 176 88 9;
2019093 144 76 79;
2019208 128 76 64;
2021078 176 88 9;
2021078 176 88 33;
2021078 144 77 69;
2021151 128 77 64;
2022687 176 88 33;
2022687 176 88 101;
2022687 144 76 55;
2022708 176 88 101;
2022708 176 88 59;
2022708 144 77 86;
2022734 128 76 64;
2022807 176 88 59;
2022807 176 88 96;
2022807 144 76 73;
2022859 128 77 64;
2022932 128 76 64;
2026541 176 88 96;
2026541 176 88 19;
2026541 144 77 90;
2026552 176 88 19;
2026552 176 88 33;
2026552 144 76 34;
2026609 128 76 64;
2026635 176 88 33;
2026635 176 88 72;
2026635 144 79 73;
2026687 128 77 64;
2026781 128 79 64;
2027203 176 88 72;
2027203 176 88 10;
2027203 144 79 64;
2027343 128 79 64;
2027604 176 88 10;
2027604 176 88 96;
2027604 144 79 60;
2027645 176 88 96;
2027645 176 88 33;
2027645 144 77 83;
2027734 176 88 33;
2027734 176 88 22;
2027734 128 79 64;
2027734 144 76 51;
2027807 128 77 64;
2027880 128 76 64;

2028031 176 88 22;
2028031 176 88 99;
2028031 144 75 56;
2028182 128 75 64;
2028286 176 88 99;
2028286 176 88 47;
2028286 144 74 68;
2028447 128 74 64;
2033739 176 88 47;
2033739 176 88 27;
2033739 144 77 39;
2033750 176 88 27;
2033750 176 88 75;
2033750 144 74 42;
2033833 128 74 64;
2033848 128 77 64;
2035458 176 88 75;
2035458 176 88 34;
2035458 144 74 54;
2035463 176 88 34;
2035463 176 88 50;
2035463 144 79 54;
2035526 128 79 64;
2035531 128 74 64;
2035562 176 88 50;
2035562 176 88 108;
2035562 144 77 76;
2035708 128 77 64;
2035916 176 88 108;
2035916 176 88 92;
2035916 144 77 58;
2036000 128 77 64;
2036239 176 88 92;
2036239 176 88 46;
2036239 144 76 69;
2036348 128 76 64;
2036526 176 88 46;
2036526 176 88 114;
2036526 144 81 76;
2036666 128 81 64;
2036843 176 88 114;
2036843 176 88 8;
2036843 144 79 77;
2036963 128 79 64;
2038911 176 88 8;
2038911 176 88 125;
2038911 144 79 81;
2038932 176 88 125;
2038932 176 88 98;
2038932 144 81 69;
2039026 128 79 64;
2039046 128 81 64;
2040312 176 88 98;
2040312 176 88 113;
2040312 144 77 81;
2040317 176 88 113;
2040317 176 88 28;
2040317 144 79 84;
2040437 128 77 64;
2040442 128 79 64;
2041291 176 88 28;
2041291 176 88 90;
2041291 144 79 88;
2041312 176 88 90;
2041312 176 88 49;
2041312 144 81 74;
2041416 128 79 64;
2041442 128 81 64;
2042145 176 88 49;
2042145 176 88 80;
2042145 144 81 84;
2042151 176 88 80;
2042151 176 88 8;
2042151 144 79 77;
2042197 128 79 64;
2042197 128 81 64;
2042260 176 88 8;
2042260 176 88 98;
2042260 144 79 83;
2042270 176 88 98;
2042270 176 88 61;
2042270 144 81 82;
2042369 128 79 64;
2042385 128 81 64;
2043625 176 88 61;
2043625 176 88 107;
2043625 144 81 62;
2043640 176 88 107;
2043640 176 88 46;
2043640 144 79 52;
2043692 128 79 64;
2043692 128 81 64;
2043781 176 88 46;
2043781 176 88 6;
2043781 144 79 35;
2043791 176 88 6;
2043791 176 88 25;
2043791 144 81 37;
2043854 128 79 64;
2043864 128 81 64;
2044062 144 81 78;
2044067 176 88 25;
2044067 176 88 96;
2044067 144 83 75;
2044135 128 83 64;
2044135 128 81 64;
2044432 176 88 96;
2044432 176 88 62;
2044432 144 81 85;
2044437 176 88 62;
2044437 176 88 8;
2044437 144 79 70;
2044494 128 79 64;
2044520 128 81 64;
2048130 176 88 8;
2048130 176 88 84;
2048130 144 79 63;
2048151 176 88 84;
2048151 176 88 126;
2048151 144 81 43;
2048192 128 79 64;
2048208 128 81 64;
2048322 176 88 126;
2048322 176 88 65;
2048322 144 79 46;
2048322 176 88 65;
2048322 176 88 25;
2048322 144 81 37;
2048385 128 79 64;
2048395 128 81 64;
2048500 176 88 25;
2048500 176 88 99;
2048500 144 79 52;
2048500 144 81 54;
2048588 128 79 64;
2048598 128 81 64;
2048822 176 88 99;
2048822 176 88 57;
2048822 144 84 80;
2048828 176 88 57;
2048828 176 88 98;
2048828 144 86 71;
2048911 128 86 64;
2048932 128 84 64;
2049958 176 88 98;
2049958 176 88 115;
2049958 144 77 64;
2049958 144 79 73;
2050026 128 77 64;
2050036 128 79 64;
2054078 176 88 115;
2054078 176 88 30;
2054078 144 79 56;
2054078 144 81 61;
2054151 128 79 64;
2054151 128 81 64;
2054750 176 88 30;
2054750 176 88 88;
2054750 144 83 67;
2054760 176 88 88;
2054760 176 88 92;
2054760 144 79 50;
2054817 128 79 64;
2054817 128 83 64;
2054911 176 88 92;
2054911 176 88 46;
2054911 144 79 52;
2054947 176 88 46;
2054947 176 88 109;
2054947 144 81 48;
2055005 128 79 64;
2055005 128 81 64;
2055322 176 88 109;
2055322 176 88 121;
2055322 144 79 59;
2055343 176 88 121;
2055343 176 88 81;
2055343 144 81 47;
2055406 128 81 64;
2055416 128 79 64;
2055718 176 88 81;
2055718 176 88 63;
2055718 144 79 64;
2055744 176 88 63;
2055744 176 88 11;
2055744 144 83 55;
2055817 128 83 64;
2055822 128 79 64;
2056125 176 88 11;
2056125 176 88 119;
2056125 144 79 70;
2056130 176 88 119;
2056130 176 88 91;
2056130 144 84 66;
2056244 128 79 64;
2056250 128 84 64;
2056520 176 88 91;
2056520 176 88 101;
2056520 144 79 38;
2056520 144 81 54;
2056572 176 88 101;
2056572 176 88 42;
2056572 144 77 23;
2056630 128 77 64;
2056651 128 79 64;
2056651 128 81 64;
2060770 176 88 42;
2060770 176 88 116;
2060770 144 79 54;
2060786 176 88 116;
2060786 176 88 60;
2060786 144 81 46;
2060838 128 79 64;
2060848 128 81 64;
2060963 176 88 60;
2060963 176 88 24;
2060963 144 79 66;
2060979 176 88 24;
2060979 176 88 59;
2060979 144 77 64;
2061494 128 79 64;
2061505 128 77 64;
2065546 176 88 59;
2065546 176 88 77;
2065546 144 77 79;
2065546 144 79 81;
2065729 128 77 64;
2065750 128 79 64;
2067255 176 88 77;
2067255 176 88 44;
2067255 144 72 83;
2067260 176 88 44;
2067260 176 88 88;
2067260 144 71 79;
2068078 128 71 64;
2068083 128 72 64;
2076786 176 88 88;
2076786 176 88 69;
2076786 144 69 33;
2076901 128 69 64;
2076968 176 88 69;
2076968 176 88 18;
2076968 144 71 28;
2077046 176 88 18;
2077046 176 88 3;
2077046 144 69 37;
2077052 128 71 64;
2077145 128 69 64;
2077156 176 88 3;
2077156 176 88 117;
2077156 144 71 41;
2077260 128 71 64;
2077265 176 88 117;
2077265 176 88 76;
2077265 144 69 49;
2077380 176 88 76;
2077380 176 88 5;
2077380 128 69 64;
2077380 144 71 49;
2077473 176 88 5;
2077473 176 88 127;
2077473 144 69 54;
2077479 128 71 64;
2077588 176 88 127;
2077588 176 88 119;
2077588 144 71 57;
2077609 128 69 64;
2077703 176 88 119;
2077703 176 88 64;
2077703 144 69 58;
2077729 128 71 64;
2077817 176 88 64;
2077817 176 88 0;
2077817 144 71 62;
2077859 128 69 64;
2077932 176 88 0;
2077932 176 88 64;
2077932 144 69 58;
2077968 128 71 64;
2078119 176 88 64;
2078119 176 88 52;
2078119 144 67 67;
2078140 128 69 64;
2078255 176 88 52;
2078255 176 88 38;
2078255 144 69 46;
2078265 128 67 64;
2078447 176 88 38;
2078447 176 88 116;
2078447 144 71 66;
2078473 128 69 64;
2078557 176 88 116;
2078557 176 88 68;
2078557 144 69 50;
2078588 128 71 64;
2078708 176 88 68;
2078708 176 88 56;
2078708 144 71 35;
2078734 128 69 64;
2078796 176 88 56;
2078796 176 88 52;
2078796 144 67 53;
2078812 128 71 64;
2078973 176 88 52;
2078973 176 88 43;
2078973 144 69 48;
2078979 128 67 64;
2079369 176 88 43;
2079369 176 88 57;
2079369 144 71 46;
2079395 128 69 64;
2079864 176 88 57;
2079864 176 88 35;
2079864 144 69 34;
2079890 128 71 64;
2081838 128 69 64;
2082557 176 88 35;
2082557 176 88 57;
2082557 144 69 30;
2082562 176 88 57;
2082562 176 88 25;
2082562 144 71 32;
2083656 128 69 64;
2083781 128 71 64;
2084140 176 88 25;
2084140 176 88 16;
2084140 144 71 33;
2085494 128 71 64;
2088218 176 88 16;
2088218 176 88 39;
2088218 144 70 18;
2089567 128 70 64;
2089583 176 88 39;
2089583 176 88 31;
2089583 144 73 14;
2090890 176 88 31;
2090890 176 88 104;
2090890 144 75 24;
2090953 128 73 64;
2092213 128 75 64;
2092270 176 88 104;
2092270 176 88 97;

2092270 144 73 19;
2092630 128 73 64;
2092687 176 88 97;
2092687 176 88 45;
2092687 144 70 12;
2093729 176 88 45;
2093729 176 88 9;
2093729 144 69 24;
2093781 128 70 64;
2094390 128 69 64;
2095395 176 88 9;
2095395 176 88 88;
2095395 144 69 31;
2095447 176 88 88;
2095447 176 88 102;
2095447 144 72 19;
2095541 128 69 64;
2095572 128 72 64;
2100098 176 88 102;
2100098 176 88 123;
2100098 144 72 29;
2100364 176 88 123;
2100364 176 88 77;
2100364 144 69 31;
2100395 128 72 64;
2100500 128 69 64;
2100942 176 88 77;
2100942 176 88 57;
2100942 144 69 30;
2101203 128 69 64;
2101203 176 88 57;
2101203 176 88 99;
2101203 144 72 35;
2101296 128 72 64;
2105072 176 88 99;
2105072 176 88 75;
2105072 144 72 23;
2105364 176 88 75;
2105364 176 88 76;
2105364 144 69 26;
2105442 128 72 64;
2105489 128 69 64;
2105994 176 88 76;
2105994 176 88 3;
2105994 144 69 29;
2106270 176 88 3;
2106270 176 88 40;
2106270 144 74 37;
2106307 128 69 64;
2106369 128 74 64;
2109817 176 88 40;
2109817 176 88 95;
2109817 144 74 29;
2109921 128 74 64;
2110296 176 88 95;
2110296 176 88 10;
2110296 144 74 34;
2110380 128 74 64;
2110750 176 88 10;
2110750 176 88 66;
2110750 144 72 32;
2110859 128 72 64;
2111213 176 88 66;
2111213 176 88 58;
2111213 144 69 34;
2111343 128 69 64;
2114697 176 88 58;
2114697 176 88 49;
2114697 144 69 15;
2114994 128 69 64;
2115411 176 88 49;
2115411 176 88 38;
2115411 144 74 32;
2115552 128 74 64;
2116041 176 88 38;
2116041 176 88 57;
2116041 144 69 28;
2116317 128 69 64;
2116317 176 88 57;
2116317 176 88 45;
2116317 144 72 34;
2116411 128 72 64;
2116812 176 88 45;
2116812 176 88 80;
2116812 144 72 34;
2116937 128 72 64;
2117765 176 88 80;
2117765 176 88 14;
2117765 144 69 35;
2117890 128 69 64;
2118250 176 88 14;
2118250 176 88 28;
2118250 144 71 41;
2118343 128 71 64;
2118697 176 88 28;
2118697 176 88 30;
2118697 144 74 44;
2118786 128 74 64;
2119468 176 88 30;
2119468 176 88 118;
2119468 144 69 42;
2119572 128 69 64;
2119786 176 88 118;
2119786 176 88 46;
2119786 144 67 45;
2119859 128 67 64;
2127562 176 88 46;
2127562 176 88 47;
2127562 144 69 33;
2127661 128 69 64;
2127776 176 88 47;
2127776 176 88 96;
2127776 144 71 33;
2127911 128 71 64;
2128067 176 88 96;
2128067 176 88 1;
2128067 144 69 30;
2128197 128 69 64;
2128562 176 88 1;
2128562 176 88 29;
2128562 144 74 40;
2128692 128 74 64;
2129572 176 88 29;
2129572 176 88 108;
2129572 144 67 36;
2129723 128 67 64;
2130515 176 88 108;
2130515 176 88 106;
2130515 144 69 39;
2130677 128 69 64;
2131473 176 88 106;
2131473 176 88 23;
2131473 144 65 45;
2131635 128 65 64;
2134348 176 88 23;
2134348 176 88 10;
2134348 144 69 40;
2134437 128 69 64;
2134583 176 88 10;
2134583 176 88 39;
2134583 144 71 31;
2134677 128 71 64;
2134822 176 88 39;
2134822 176 88 74;
2134822 144 72 41;
2134911 128 72 64;
2135281 176 88 74;
2135281 176 88 110;
2135281 144 69 40;
2135406 128 69 64;
2135713 176 88 110;
2135713 176 88 63;
2135713 144 74 46;
2135807 128 74 64;
2136166 176 88 63;
2136166 176 88 66;
2136166 144 69 28;
2136286 128 69 64;
2136718 176 88 66;
2136718 176 88 8;
2136718 144 71 23;
2136848 128 71 64;
2137208 176 88 8;
2137208 176 88 60;
2137208 144 67 48;
2137333 128 67 64;
2142005 176 88 60;
2142005 176 88 125;
2142005 144 71 37;
2142125 128 71 64;
2142968 176 88 125;
2142968 176 88 90;
2142968 144 69 36;
2143078 128 69 64;
2146984 176 88 90;
2146984 176 88 30;
2146984 144 67 36;
2147078 128 67 64;
2147552 176 88 30;
2147552 176 88 19;
2147552 144 67 24;
2147625 128 67 64;
2147718 176 88 19;
2147718 176 88 14;
2147718 144 67 47;
2147802 128 67 64;
2148494 176 88 14;
2148494 176 88 57;
2148494 144 67 44;
2148598 128 67 64;
2150239 176 88 57;
2150239 176 88 103;
2150239 144 65 23;
2150755 128 65 64;
2158322 176 88 103;
2158322 176 88 7;
2158322 144 69 10;
2159010 128 69 64;
2160276 176 88 7;
2160276 176 88 64;
2160276 144 69 55;
2160328 128 69 64;
2160406 176 88 64;
2160406 176 88 84;
2160406 144 69 60;
2160505 128 69 64;
2166197 176 88 84;
2166197 176 88 35;
2166197 144 65 42;
2166203 176 88 35;
2166203 176 88 119;
2166203 144 69 29;
2166322 128 69 64;
2166364 128 65 64;
2166927 176 88 119;
2166927 176 88 52;
2166927 144 69 35;
2166942 176 88 52;
2166942 176 88 15;
2166942 144 65 28;
2167036 128 69 64;
2167041 128 65 64;
2167104 176 88 15;
2167104 176 88 68;
2167104 144 67 33;
2167192 128 67 64;
2167604 176 88 68;
2167604 176 88 39;
2167604 144 67 40;
2167692 128 67 64;
2168046 176 88 39;
2168046 176 88 122;
2168046 144 65 28;
2168046 176 88 122;
2168046 176 88 69;
2168046 144 69 33;
2168166 128 65 64;
2168171 128 69 64;
2173869 176 88 69;
2173869 176 88 108;
2173869 144 57 33;
2174036 128 57 64;
2174692 176 88 108;
2174692 176 88 29;
2174692 144 57 26;
2174786 128 57 64;
2174911 176 88 29;
2174911 176 88 30;
2174911 144 59 33;
2174994 128 59 64;
2175437 176 88 30;
2175437 176 88 88;
2175437 144 57 40;
2175546 128 57 64;
2176031 176 88 88;
2176031 176 88 7;
2176031 144 55 44;
2176687 128 55 64;
2184713 176 88 7;
2184713 176 88 21;
2184713 144 56 54;
2185614 128 56 64;
2185968 176 88 21;
2185968 176 88 87;
2185968 144 57 38;
2186302 128 57 64;
2186781 176 88 87;
2186781 176 88 9;
2186781 144 55 51;
2186958 128 55 64;
2204812 176 88 9;
2204812 176 88 66;
2204812 144 65 26;
2206505 176 88 66;
2206505 176 88 21;
2206505 144 64 20;
2206578 128 65 64;
2208307 176 88 21;
2208307 176 88 67;
2208307 144 62 20;
2208432 128 64 64;
2210531 176 88 67;
2210531 176 88 0;
2210531 144 57 25;
2210713 128 62 64;
2212598 176 88 0;
2212598 176 88 38;
2212598 144 65 24;
2215197 128 65 64;
2215364 128 57 64;
2216078 176 88 38;
2216078 176 88 72;
2216078 144 65 28;
2216083 176 88 72;
2216083 176 88 79;
2216083 144 57 30;
2219468 176 88 79;
2219468 176 88 98;
2219468 144 60 21;
2221854 128 57 64;
2222109 128 60 64;
2222385 128 65 64;
2222437 176 88 98;
2222437 176 88 29;
2222437 144 57 20;
2223437 176 88 29;
2223437 176 88 21;
2223437 144 61 23;
2223468 176 88 21;
2223468 176 88 80;
2223468 144 66 25;
2225437 128 57 64;
2229864 128 66 64;
2229885 128 61 64;
2287031 176 64 0;
2287031 176 64 127;
2287776 176 64 127;
2287776 176 64 0;
2462369 176 64 0;
2462369 176 64 127;
2463041 176 64 127;
2463041 176 64 0;
2472390 176 64 0;
2472390 176 64 127;
2473473 176 64 127;
2473473 176 64 0;
2479687 176 64 0;
2479687 176 64 127;
2480442 176 64 127;
2480442 176 64 0;
2591963 176 88 80;
2591963 176 88 89;
2591963 144 50 30;
2592479 176 88 89;
2592479 176 88 59;
2592479 144 53 36;
2592526 128 50 64;
2592697 128 53 64;
2593500 176 88 59;
2593500 176 88 124;
2593500 144 53 33;
2593947 128 53 64;

2593979 176 88 124;
2593979 176 88 38;
2593979 144 57 29;
2594119 128 57 64;
2594817 176 88 38;
2594817 176 88 44;
2594817 144 52 37;
2595182 128 52 64;
2595213 176 88 44;
2595213 176 88 112;
2595213 144 55 35;
2595359 128 55 64;
2596020 176 88 112;
2596020 176 88 42;
2596020 144 55 31;
2596390 128 55 64;
2596437 176 88 42;
2596437 176 88 119;
2596437 144 59 29;
2596578 128 59 64;
2597859 176 88 119;
2597859 176 88 67;
2597859 144 50 24;
2598104 128 50 64;
2598708 176 88 67;
2598708 176 88 118;
2598708 144 53 14;
2598979 128 53 64;
2599572 176 88 118;
2599572 176 88 35;
2599572 144 53 28;
2599697 128 53 64;
2599937 176 88 35;
2599937 176 88 66;
2599937 144 57 28;
2600166 128 57 64;
2600729 176 88 66;
2600729 176 88 39;
2600729 144 52 14;
2601088 128 52 64;
2601390 176 88 39;
2601390 176 88 83;
2601390 144 55 19;
2601890 128 55 64;
2602348 176 88 83;
2602348 176 88 58;
2602348 144 55 22;
2602619 128 55 64;
2602890 176 88 58;
2602890 176 88 116;
2602890 144 59 21;
2603281 128 59 64;
2606989 176 88 116;
2606989 176 88 60;
2606989 144 38 28;
2609265 176 88 60;
2609265 176 88 28;
2609265 144 62 22;
2609796 128 38 64;
2611125 176 88 28;
2611125 176 88 2;
2611125 144 50 17;
2611286 128 62 64;
2612901 176 88 2;
2612901 176 88 97;
2612901 144 81 23;
2613151 128 50 64;
2614953 176 88 97;
2614953 176 88 101;
2614953 144 38 16;
2615296 128 81 64;
2616625 176 88 101;
2616625 176 88 68;
2616625 144 77 24;
2617708 128 38 64;
2618437 176 88 68;
2618437 176 88 21;
2618437 144 33 24;
2619302 128 77 64;
2620307 176 88 21;
2620307 176 88 32;
2620307 144 74 13;
2621359 128 33 64;
2622338 176 88 32;
2622338 176 88 116;
2622338 144 38 23;
2622666 128 74 64;
2624031 176 88 116;
2624031 176 88 81;
2624031 144 83 16;
2625812 176 88 81;
2625812 176 88 82;
2625812 144 31 23;
2625880 128 38 64;
2626046 128 83 64;
2627484 176 88 82;
2627484 176 88 115;
2627484 144 88 15;
2628510 128 31 64;
2629500 176 88 115;
2629500 176 88 46;
2629500 144 45 21;
2629854 128 88 64;
2631286 176 88 46;
2631286 176 88 76;
2631286 144 77 16;
2632687 128 45 64;
2633276 176 88 76;
2633276 176 88 77;
2633276 144 47 19;
2633947 128 77 64;
2635072 176 88 77;
2635072 176 88 42;
2635072 144 74 15;
2636062 128 47 64;
2637015 176 88 42;
2637015 176 88 88;
2637015 144 53 13;
2637369 128 74 64;
2638911 176 88 88;
2638911 176 88 53;
2638911 144 69 8;
2639583 128 53 64;
2640911 176 88 53;
2640911 176 88 86;
2640911 144 38 23;
2641130 128 69 64;
2641338 128 38 64;
2641364 176 88 86;
2641364 176 88 111;
2641364 144 41 24;
2643114 128 41 64;
2643218 176 88 111;
2643218 176 88 1;
2643218 144 91 32;
2645046 176 88 1;
2645046 176 88 113;
2645046 144 52 13;
2645218 128 91 64;
2646776 176 88 113;
2646776 176 88 121;
2646776 144 79 5;
2648005 128 79 64;
2648593 176 88 121;
2648593 176 88 34;
2648593 144 76 20;
2648609 144 55 29;
2648687 128 52 64;
2650197 176 88 34;
2650197 176 88 102;
2650197 144 72 22;
2650223 176 88 102;
2650223 176 88 100;
2650223 128 55 64;
2650223 144 60 20;
2650296 128 76 64;
2651906 176 88 100;
2651906 176 88 3;
2651906 144 48 17;
2652067 128 60 64;
2652135 128 72 64;
2653697 176 88 3;
2653697 176 88 55;
2653697 144 91 11;
2654703 128 48 64;
2655463 176 88 55;
2655463 176 88 54;
2655463 144 45 20;
2655802 128 91 64;
2657223 176 88 54;
2657223 176 88 95;
2657223 144 85 15;
2657885 128 45 64;
2659312 176 88 95;
2659312 176 88 125;
2659312 144 40 17;
2659703 128 85 64;
2661109 176 88 125;
2661109 176 88 17;
2661109 144 86 11;
2662619 128 40 64;
2662963 176 88 17;
2662963 176 88 82;
2662963 144 38 3;
2664109 128 86 64;
2664739 176 88 82;
2664739 176 88 17;
2664739 144 89 12;
2666192 128 38 64;
2666604 128 89 64;
2666718 176 88 17;
2666718 176 88 0;
2666718 144 43 1;
2668333 144 79 1;
2669526 176 88 0;
2669526 176 88 9;
2669526 144 47 12;
2669552 128 43 64;
2670411 176 88 9;
2670411 176 88 116;
2670411 144 76 14;
2670468 128 79 64;
2670979 176 88 116;
2670979 176 88 36;
2670979 144 50 34;
2671046 128 47 64;
2671593 128 76 64;
2671604 176 88 36;
2671604 176 88 25;
2671604 144 71 20;
2672255 176 88 25;
2672255 176 88 51;
2672255 144 55 13;
2672270 128 50 64;
2672963 176 88 51;
2672963 176 88 85;
2672963 144 67 14;
2673057 128 71 64;
2674390 128 55 64;
2674442 176 88 85;
2674442 176 88 4;
2674442 144 60 3;
2674817 128 67 64;
2675328 176 88 4;
2675328 176 88 119;
2675328 144 69 17;
2676177 176 88 119;
2676177 176 88 55;
2676177 144 57 26;
2676307 128 60 64;
2676307 128 69 64;
2676911 176 88 55;
2676911 176 88 61;
2676911 144 72 10;
2677635 176 88 61;
2677635 176 88 60;
2677635 144 55 23;
2677645 128 57 64;
2677776 128 72 64;
2678359 176 88 60;
2678359 176 88 11;
2678359 144 74 24;
2679072 128 55 64;
2679098 176 88 11;
2679098 176 88 13;
2679098 144 52 27;
2679322 128 74 64;
2679916 176 88 13;
2679916 176 88 8;
2679916 144 79 27;
2680546 128 52 64;
2681557 128 79 64;
2681609 176 88 8;
2681609 176 88 52;
2681609 144 40 7;
2682265 176 88 52;
2682265 176 88 78;
2682265 144 88 20;
2683125 128 40 64;
2683145 176 88 78;
2683145 176 88 124;
2683145 144 45 27;
2683276 128 88 64;
2683755 176 88 124;
2683755 176 88 15;
2683755 144 84 26;
2684661 128 84 64;
2684713 176 88 15;
2684713 176 88 51;
2684713 144 50 27;
2684770 128 45 64;
2685270 176 88 51;
2685270 176 88 101;
2685270 144 79 16;
2688145 128 50 64;
2688192 176 88 101;
2688192 176 88 72;
2688192 144 47 18;
2688197 176 88 72;
2688197 176 88 14;
2688197 144 83 21;
2688244 128 79 64;
2690427 128 83 64;
2690463 176 88 14;
2690463 176 88 64;
2690463 144 43 28;
2690531 128 47 64;
2691119 176 88 64;
2691119 176 88 112;
2691119 144 81 18;
2691921 176 88 112;
2691921 176 88 94;
2691921 144 41 29;
2691947 128 81 64;
2692020 128 43 64;
2692708 176 88 94;
2692708 176 88 47;
2692708 144 84 23;
2693395 128 41 64;
2693463 176 88 47;
2693463 176 88 86;
2693463 144 40 35;
2693468 128 84 64;
2694380 176 88 86;
2694380 176 88 23;
2694380 144 88 26;
2695703 176 88 23;
2695703 176 88 84;
2695703 144 36 18;
2695708 176 88 84;
2695708 176 88 36;
2695708 144 91 34;
2695781 128 40 64;
2695796 128 88 64;
2700713 128 91 64;
2700750 128 36 64;
2701041 176 88 36;
2701041 176 88 35;
2701041 144 36 19;
2701979 176 88 35;
2701979 176 88 93;
2701979 144 79 8;
2702500 128 36 64;
2703682 176 88 93;
2703682 176 88 117;
2703682 144 43 20;
2703817 128 79 64;
2704630 176 88 117;
2704630 176 88 114;
2704630 144 86 13;
2705796 128 43 64;
2706307 176 88 114;
2706307 176 88 76;
2706307 144 41 22;
2706369 128 86 64;
2707229 176 88 76;
2707229 176 88 43;
2707229 144 84 17;
2708161 128 41 64;
2709114 176 88 43;
2709114 176 88 126;
2709114 144 48 18;

2709130 128 84 64;
2709927 176 88 126;
2709927 176 88 120;
2709927 144 79 18;
2710958 128 48 64;
2711932 176 88 120;
2711932 176 88 87;
2711932 144 45 27;
2712125 128 79 64;
2712692 176 88 87;
2712692 176 88 121;
2712692 144 76 20;
2714265 176 88 121;
2714265 176 88 99;
2714265 144 50 19;
2714296 128 76 64;
2714380 128 45 64;
2714947 176 88 99;
2714947 176 88 68;
2714947 144 69 17;
2715583 128 50 64;
2716682 176 88 68;
2716682 176 88 63;
2716682 144 47 10;
2716817 128 69 64;
2717359 176 88 63;
2717359 176 88 67;
2717359 144 66 21;
2717937 128 47 64;
2718979 176 88 67;
2718979 176 88 45;
2718979 144 53 24;
2719088 128 66 64;
2719635 176 88 45;
2719635 176 88 9;
2719635 144 72 22;
2720697 128 53 64;
2721390 176 88 9;
2721390 176 88 65;
2721390 144 50 22;
2721567 128 72 64;
2722166 176 88 65;
2722166 176 88 114;
2722166 144 69 19;
2724203 176 88 114;
2724203 176 88 95;
2724203 144 47 11;
2724348 128 50 64;
2724401 128 69 64;
2724973 176 88 95;
2724973 176 88 59;
2724973 144 66 20;
2725661 128 47 64;
2727109 176 88 59;
2727109 176 88 67;
2727109 144 45 21;
2727317 128 66 64;
2727838 176 88 67;
2727838 176 88 26;
2727838 144 65 13;
2729796 128 45 64;
2730125 128 65 64;
2730156 176 88 26;
2730156 176 88 19;
2730156 144 45 11;
2730234 176 88 19;
2730234 176 88 80;
2730234 144 64 21;
2732661 176 88 80;
2732661 176 88 88;
2732661 144 40 14;
2732786 128 64 64;
2732791 128 45 64;
2735187 176 88 88;
2735187 176 88 117;
2735187 144 76 7;
2736406 128 76 64;
2736989 176 88 117;
2736989 176 88 42;
2736989 144 74 20;
2737010 176 88 42;
2737010 176 88 16;
2737010 144 43 32;
2737036 128 40 64;
2739281 128 74 64;
2739729 128 43 64;
2739760 176 88 16;
2739760 176 88 103;
2739760 144 41 32;
2739802 176 88 103;
2739802 176 88 67;
2739802 144 72 18;
2741380 128 72 64;
2741947 176 88 67;
2741947 176 88 36;
2741947 144 76 20;
2742026 176 88 36;
2742026 176 88 62;
2742026 144 45 34;
2742104 128 41 64;
2744364 128 76 64;
2744854 176 88 62;
2744854 176 88 58;
2744854 144 48 28;
2744885 176 88 58;
2744885 176 88 7;
2744885 144 69 13;
2744895 128 45 64;
2748322 176 88 7;
2748322 176 88 11;
2748322 144 52 21;
2748328 128 48 64;
2748333 176 88 11;
2748333 176 88 25;
2748333 144 67 24;
2748380 128 69 64;
2751166 176 88 25;
2751166 176 88 95;
2751166 128 52 64;
2751166 144 65 17;
2751171 176 88 95;
2751171 176 88 113;
2751171 144 50 20;
2751291 128 67 64;
2753312 176 88 113;
2753312 176 88 47;
2753312 144 69 15;
2753333 128 65 64;
2753369 176 88 47;
2753369 176 88 121;
2753369 144 53 7;
2753484 128 50 64;
2755223 176 88 121;
2755223 176 88 116;
2755223 144 52 28;
2755250 176 88 116;
2755250 176 88 21;
2755250 144 67 21;
2755260 128 53 64;
2755359 128 69 64;
2757031 176 88 21;
2757031 176 88 111;
2757031 128 52 64;
2757031 144 52 2;
2757677 176 88 111;
2757677 176 88 23;
2757677 144 64 23;
2757755 176 88 23;
2757755 176 88 49;
2757755 144 48 20;
2757796 128 52 64;
2757937 128 67 64;
2760250 128 48 64;
2760265 176 88 49;
2760265 176 88 87;
2760265 144 65 20;
2760312 176 88 87;
2760312 176 88 74;
2760312 144 50 25;
2760406 128 64 64;
2762927 176 88 74;
2762927 176 88 19;
2762927 144 43 27;
2762932 176 88 19;
2762932 176 88 36;
2762932 144 71 22;
2763052 128 50 64;
2763140 128 65 64;
2767473 176 88 36;
2767473 176 88 65;
2767473 144 36 25;
2767489 176 88 65;
2767489 176 88 112;
2767489 144 72 19;
2767583 128 43 64;
2767734 128 71 64;
2770635 128 72 64;
2770635 128 36 64;
